# An appraisal of peer-reviewed published literature on Influenza, 2000–2021 from countries in South-East Asia Region

**DOI:** 10.3389/fpubh.2023.1127891

**Published:** 2023-04-17

**Authors:** Pushpa Ranjan Wijesinghe, Divita Sharma, Bharathi Vaishnav, Ritika Mukherjee, Priyanka Pawar, Archisman Mohapatra, Nilesh Buddha, Edwin Ceniza Salvador, Manish Kakkar

**Affiliations:** ^1^World Health Organization, Regional Office for South-East Asia, World Health House, New Delhi, India; ^2^Executive Office, Generating Research Insights for Development Council (GRID Council), Noida, Uttar Pradesh, India

**Keywords:** influenza, SEAR, pandemic, zoonotic, bibliometric

## Abstract

**Background:**

Influenza poses a major public health challenge in South-East Asia Region (SEAR). To address the challenge, there is a need to generate contextual evidence that could inform policy makers and program managers for response preparedness and impact mitigation. The World Health Organization has identified priority areas across five streams for research evidence generation at a global level (WHO Public Health Research Agenda). Stream 1 focuses on research for reducing the risk of emergence, Stream 2 on limiting the spread, Stream 3 on minimizing the impact, Stream 4 on optimizing the treatment and Stream 5 on promoting public health tools and technologies for Influenza. However, evidence generation from SEAR has been arguably low and needs a relook for alignment with priorities. This study aimed to undertake a bibliometric analysis of medical literature on Influenza over the past 21 years to identify gaps in research evidence and for identifying major areas for focusing with a view to provide recommendations to member states and SEAR office for prioritizing avenues for future research.

**Methods:**

We searched Scopus, PubMed, Embase, and Cochrane databases in August 2021. We identified studies on influenza published from the 11 countries in WHO SEAR in the date range of 1 January 2000–31 December 2021. Data was retrieved, tagged and analyzed based on the WHO priority streams for Influenza, member states, study design and type of research. Bibliometric analysis was done on Vosviewer.

**Findings:**

We included a total of 1,641 articles (Stream 1: *n* = 307; Stream 2: *n* = 516; Stream 3: *n* = 470; Stream 4: *n* = 309; Stream 5: *n* = 227). Maximum number of publications were seen in Stream 2, i.e., limiting the spread of pandemic, zoonotic, and seasonal epidemic influenza which majorly included transmission, spread of virus at global and local levels and public health measures to limit the transmission. The highest number of publications was from India (*n* = 524) followed by Thailand (*n* = 407), Indonesia (*n* = 214) and Bangladesh (*n* = 158). Bhutan (*n* = 10), Maldives (*n* = 1), Democratic People’s Republic of Korea (*n* = 1), and Timor-Leste (*n* = 3) had the least contribution in Influenza research. The top-most journal was PloS One which had the maximum number of influenza articles (*n* = 94) published from SEAR countries. Research that generated actionable evidence, i.e., implementation and intervention related topics were less common. Similarly, research on pharmaceutical interventions and on innovations was low. SEAR member states had inconsistent output across the five priority research streams, and there was a much higher scope and need for collaborative research. Basic science research showed declining trends and needed reprioritization.

**Interpretation:**

While a priority research agenda has been set for influenza at the global level through the WHO Global Influenza Program since 2009, and subsequently revisited in 2011 and again in 2016–2017, a structured contextualized approach to guide actionable evidence generation activities in SEAR has been lacking. In the backset of the Global Influenza Strategy 2019–2030 and the COVID-19 pandemic, attuning research endeavors in SEAR could help in improved pandemic influenza preparedness planning. There is a need to prioritize contextually relevant research themes within priority streams. Member states must inculcate a culture of within and inter-country collaboration to produce evidence that has regional as well as global value.

## Introduction

Influenza poses as a major public health challenge in South East Asia Region (SEAR). The SEAR context is uniquely vulnerable for influenza outbreaks (1). SEAR member states (MS) face typical low- and middle-income country challenges– complex demographic and epidemiological transitions along with inequitable resource constraints (2). The continuous spread of High Pathogenic Avian Influenza (H5N1) in wild and domestic poultry in the region has posed serious threat of human pandemic risk for decades (3). Situation analyses have found that countries are often ill prepared to respond to such influenza related public health emergencies (4).

The World Health Organization (WHO) recognizes that by pursuing a priority research agenda, actionable evidence could be generated to inform strategic policy making and program designing against influenza. In 2009, the WHO Global Influenza Program held a consultation among different investigators, health policy makers, public health experts and funding agencies (5). The consultation developed a research agenda and proposed recommendations that would assist in meeting gaps in scientific understanding for preparedness and response to the emergence, transmission, mitigation and management of influenza. The recommendations were for five high priority research streams: Stream 1: Reducing the risk of emergence of Pandemic Influenza; Stream 2: Limiting the spread of pandemic, zoonotic and seasonal epidemic influenza; Stream 3: Minimizing the impact of pandemic, zoonotic and seasonal epidemic influenza; Stream 4: Optimizing the treatment of patients; and Stream 5: Promoting the development and application of modern public health tools (6).

Evaluation of the research agenda was done in 2011 to understand the major achievements, identify unmet public health needs and major knowledge gaps in order to reprioritize the areas for research. The evaluation identified several objectives to facilitate progress in influenza research (7). In August 2016, technical working groups were established for the five research streams to provide an updated agenda on priorities in influenza research for the next 5–10 years. Various sub-streams identified under each priority stream were released in 2017 (Box 1). Later in 2019, WHO proposed the *Global influenza strategy 2019–2030* as a global guidance tool to prevent, control and prepare for influenza pandemic. It identified two high level outcomes for 2030: (1) better global tools: a focused, consensus-driven plan leads to greater research, innovation and availability of new and improved tools for the prevention, detection, control and treatment of influenza; and (2) stronger country capacities: every country has a prioritized influenza program that is evidence-based, is optimized to fit the country’s needs, and contributes to national and global preparedness, response and health security (8).

BOX 1 Priority research streams identified by the WHO Global Influenza Program, 2009.

Stream 1: Reducing the risk of emergence of Pandemic Influenza.

1.1Improved surveillance and detection of emergent Influenza A virus (IAV) with zoonotic or pandemic potential for risk assessment and response.

1.2Identification of virus, host and environmental determinants for infectivity, susceptibility, transmission and pathogenesis of potentially zoonotic IAVs.

1.3Management or a modification of animal production and marketing systems for mitigation of the risk of zoonotic IAV emergence, geographic spread and transmission to humans.

1.4Improving vaccines and their application in the animal host populations to reduce human exposure to zoonotic IAV.

Stream 2: Limiting the spread of pandemic, zoonotic and seasonal epidemic influenza.

2.1Factors affecting person-to-person transmission.

2.2Dynamics of virus spread at global and local levels.

2.3Public health measures to limit transmission.

Stream 3: Minimizing the impact of pandemic, zoonotic and seasonal epidemic influenza.

3.1Determining disease burden and social impact.

3.2Improve immunogenicity, availability and delivery of influenza vaccines.

3.3Public health policies to reduce the impact of disease.

Stream 4: Optimizing the treatment of patients.

4.1Factors associated with pathogenesis and clinical severity.

4.2Improve clinical management of patients.

4.3Health care capacity and response.

Stream 5: Promoting the development and application of modern public health tools.

5.1Next-generation sequencing and other emerging technologies.

5.2Role of modeling in public health decision-making.

5.3Strategic communication.

Research output from the SEAR MS has been notably low in terms of alignment with priority areas and subsequent knowledge translation (9, 10). There is a need to appraise the body of evidence accrued from member states in SEAR to inform future efforts at prioritizing research for efficient and effective influenza preparedness and response. Considering that strategic evidence generation for public health action against influenza is a pressing need in SEAR, we have undertaken a bibliometric analysis of medical literature on Influenza and related diseases, its control, prevention and pandemic preparedness and response published from/on SEAR. We have focused on literature available through, approximately, the past 21 years (1 January 2000–31 March 2021) to map existing evidence to priority research streams and identify gaps in research evidence. The current analysis aims to demonstrate growth and trends of publications and contribution of individual authors, their institutions and representative country as well as the extent of collaborations between them. Our broader goal was to identify priority avenues for future research on Influenza in SEAR.

## Methodology

### About bibliometric analysis

Bibliometric analysis has been widely used in academic explorations of trends in literature available and contributions of countries or regions, researchers and journals in this regard. Application of bibliometric analysis in evidence mapping can identify scientific relationships between research members, institutions and countries for specific health related domains. Components of bibliometric analysis such as co-publications and co-authorships, can highlight main contributors in research and provide a description of scientific network. It provides an opportunity to various researchers and stakeholders, to gain an understanding of the field of research and promote cross-disciplinary collaboration while identifying gaps in knowledge areas (11, 12).

### Literature search and selection strategy

We performed a systematic search of Scopus, PubMed, Embase and Cochrane databases for publications on influenza from the 11 countries in WHO SEAR including that from WHO SEAR Office. We searched for literature published in volumes/issues of various journals dated 1st January 2000 till 31st December 2021; we conducted the literature search in August 2021. The key words for the search were identified after initial review of search strategies used in recent systematic reviews on influenza and other reviews as deemed relevant for this study.

The search was carried out at two levels:

- Focus population: The keywords used were “SEAR,” “South Asia,” “South-East Asia,” “Southeast Asia,” “South-East Asia Region” OR “Southeast Asia Region” and the name of each MS in SEAR.
- Focus Condition: The keywords identified were “Influenza,” “parainfluenza,” “flu” and other search terms related to influenza, e.g., Hemagglutinin, Neuraminidase, etc.

The above-mentioned keywords were incorporated in the search strategy. Medical sub-heading (MeSH) terms and possible synonyms were included. For the scientific and research databases, appropriate Boolean operators and search modifiers [date range (1st January 2000 till 31 December 2021 (to capture ahead of print articles)), English] were used. Articles that did not have primary focus on influenza, editorials, announcements, and presentations, and articles not written in English language were excluded.

The detailed search strategy has been provided in Supplementary File Table S1-1.

### Data extraction and management

Articles from the four databases were imported into Rayyan online software.^1^ Two review authors independently screened the titles and abstracts identified as a result of the searches to select potentially eligible studies. Full text study reports/publications were retrieved and two review authors independently screened the full texts and identified studies for inclusion, and also identified and recorded reasons for exclusion of the ineligible studies. Any conflicts or disagreements were resolved through discussion and consensus with the third reviewer (senior researcher) in alignment with preset decision rules. The study was included in the analysis if answer to both of the following was “yes”: “Is the study related to SEAR or any of the SEAR countries? Is the study related to Influenza?”

All the included articles were tagged based on the five streams developed by WHO Public Health Research Agenda for Influenza, country/geographic scope of the article, type of study design and method of research. A structured data extraction form was developed in Microsoft Excel worksheet to collect data elements of each study. These data elements included information on the five WHO priority research streams, country of study focus [Bangladesh, Bhutan, India, Indonesia, Maldives, Myanmar, Nepal, Democratic People’s Republic of Korea (DPRK), Sri Lanka, Thailand, Timor-Leste, SEAR, Asia, Global], study design (“bench research” included empirical research designs), “observational” (included descriptive, non-interventional analytical designs, and data modeling), “interventional” (included experimental designs, e.g., trials), and “review” (including narrative and systematic reviews and guidelines), method of research [“basic science” (fundamental and -omics research), “clinical” (articles on all diagnostic elements such as patient signs and symptoms and radiologic or laboratory findings, including molecular diagnosis), “socio-behavioral and public health” (articles on public health, health systems and policy research)], authors, journal, year of publication, abstract, objectives and findings.

Bibliography was managed using Zotero software.

### Data analysis

Descriptive statistics (frequency and proportion) of articles according to stream, study design, type of research and country of study was computed for the decades 2000–2010 and 2011–2021, and overall, i.e., 2000–2021. Further, to summarize the landscape of influenza research, tables were generated by cross-tabulation of article topics with article types, primary focus, and country of study in Microsoft Excel. To understand the trend and research gap, appropriate graphs were generated.

Publication characteristics were tabulated, including top authors, journal sources, affiliations of authors and countries or regions to which the authors belong. VOSviewer (version 1∙6∙17) software was used to create network visualization maps for the authors that had contributed most to the Influenza research in the SEAR MS. The strength of research collaboration was presented as Total Link Strength (TLS), which was automatically given by VOSviewer upon mapping research activity of selected authors. The TLS is proportional to the extent of research collaboration; a higher TLS value indicates more frequent collaboration. VOSviewer obtained clusters *via* analyzing the links between different authors appearing within the different publications. Different nodes in the map represented the authors. The size of the nodes reflected the number of publications or frequency; larger the node, greater the number of publications or frequency. The links between nodes represent relationships of collaboration among different authors. Each cluster is represented by its color for the nodes and lines. Each cluster represents the connectivity of authors within that cluster. The distance between two authors in the visualization approximately indicates the relatedness of the authors in terms of co-citation links. In general, the closer two authors are located to each other, the stronger their relatedness.

Journals were arranged in descending order of number of influenza related articles published from SEAR MS. The top 10 journals were identified as per their sequence in the order thus generated. Top authors were presented globally and for Bangladesh, India, Indonesia, Nepal and Thailand in descending order of their TLS. Publications and journals were assessed using h-index. The h-index for articles was taken from Scopus database while the h-index for journals was taken from Scimago. Impact factor, Scimago journal rank, Source normalized impact per paper and Cite Score for the top 10 journals were also enlisted.

For identifying areas of research within the priority streams, frequency of keywords was counted. Based on the frequency of these keywords, we reflected on areas of research that were less frequently visited in the publications along with a deep-dive to see if a gap in research was indeed palpable in the context of SEAR MS and with due consideration to the research trends identified by this exercise. This was an iterative, a qualitative assessment undertaken by the team of authors of this manuscript in consensus. (The authors have more than 30 years of cumulative experience of working on influenza and infectious diseases as researchers and public health program managers in SEAR.)

## Results

### Volume of articles

Out of 7,368 articles, 1,641 (22∙3%) were unique articles that were included in the final analysis, detailed bibliography of which is given in Additional File. Detailed flow diagram is given in the Supplementary File #4, Supplementary Figure S4-1. Of these, 410 (25%) had been published between 2000 and 2010, and 1,231 (75%) between 2011 and 2021. Thus, the volume of literature on influenza produced from SEAR MS had tripled between 2011 and 2021 as compared to that in the previous decade.

### Stream of research

The maximum number of publications, overall, between 2000 and 2021, were in Stream 2, i.e., limiting the spread of pandemic, zoonotic, and seasonal epidemic influenza which majorly included transmission, spread of virus at global and local levels and public health measures to limit the transmission (Table 1). However, maximum decadal growth rate (DGR = 3.1) was seen Stream 3, i.e., minimizing the impact of pandemic, zoonotic and seasonal epidemic influenza which included influenza disease burden and social impact, influenza vaccines and public health policies.

**Table 1:** Distribution of articles included in the review according to WHO Influenza Streams.

S.no	Stream	Decade		Total n^ψ^(%)	Decadal growth rate
		2000–2010 n*(%)	2011–2021 *n*^ϕ^(%)		
1	Reducing the risk of emergence of a pandemic influenza	85 (20.7)	222 (18.0)	307 (18.7)	1.6
2	Limiting the spread of pandemic, zoonotic, and seasonal epidemic influenza	154 (37.6)	362 (29.4)	516 (31.4)	1.3
3	Minimizing the impact of pandemic, zoonotic and seasonal epidemic influenza	93 (22.7)	377 (30.6)	470 (28.6)	3.1
4	Optimizing the treatment of patients	80 (19.5)	229 (18.6)	309 (18.8)	1.9
5	Promoting the development and application of new public health tools	60 (14.6)	167 (13.6)	227 (13.8)	1.8

n* = 410, *n*^ϕ^ = 1,231, *n*^Ψ^ = 1,641.

### Performance of SEAR countries

**Figure 1:**
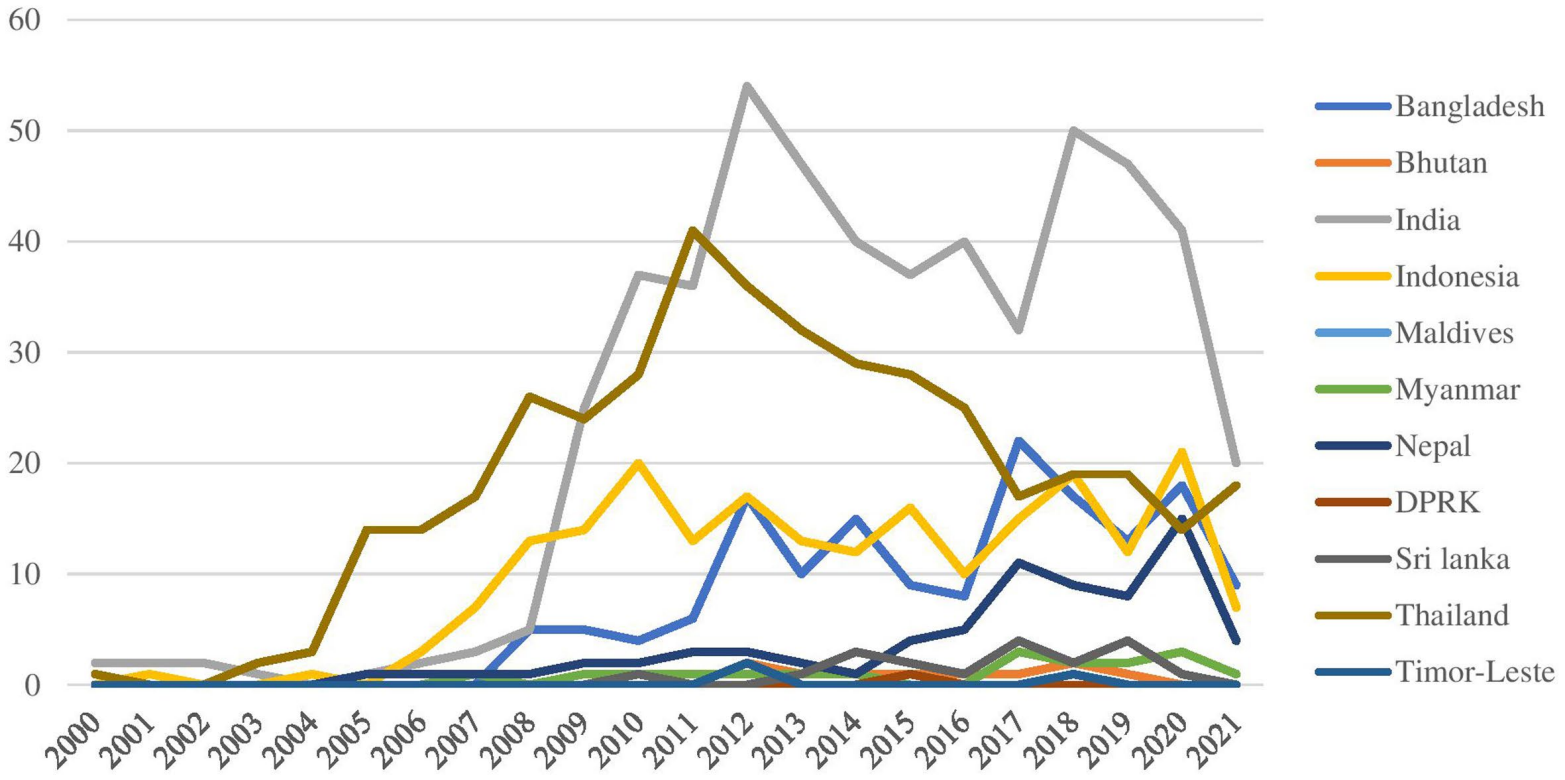
Trend in the number of publications from SEAR member states, 2000–2021.

We observed variations in the influenza research trends in SEAR countries between 2000 and 2021 with noticeable trend-change in most countries around late 2000s and thereafter (Figure 1). This was also around the time when the H1N1/09 flu pandemic (2009–2010) happened and the influenza research agenda setting consultations were undertaken leading to the development of the research agenda on Influenza by WHO Global Influenza Program (7). There were lesser number of articles seen during years 2014–2016 which later increased from 2017 during which WHO updated the influenza research agenda.

Among SEAR countries, India had produced the maximum number of papers (*n* = 524; DGR = 4.5) followed by Thailand (*n* = 407; DGR = 1.1), Indonesia (*n* = 214; DGR = 1.6) and Bangladesh (*n* = 158; DGR = 9.3; Table 2; Supplementary File Table S1-2). These countries had produced papers across all 5 streams. While Bangladesh and Indonesia focused majorly on Stream 1, 2, and 3, India focused on Streams 2, 3, and 4, and Thailand on Streams 2 and 3. Stream 5 was a relatively new and upcoming stream for SEAR countries.

**Table 2:** Distribution of articles included in the review according to both stream and South East Asian Region countries.

S.no	Country	Reducing the risk of emergence of a pandemic influenza		Limiting the spread of pandemic, zoonotic, and seasonal epidemic influenza		Minimizing the impact of pandemic, zoonotic and seasonal epidemic influenza		Optimizing the treatment of patients		Promoting the development and application of new public health tools		Overall	
		Total *N* = 307 (%)	DGR	Total *N* = 516 (%)	DGR	Total *N* = 470 (%)	DGR	Total *N* = 309 (%)	DGR	Total *N* = 227 (%)	DGR	Total *N* = 1,641 (%)	DGR
1	Bangladesh	61 (19.9)	18.3	46 (8.9)	5.7	37 (7.9)	7.3	14 (4.5)	5	17 (7.5)	6.5	158 (9.6)	9.3
2	Bhutan	4 (1.3)	–	3 (0.6)	–	3 (0.6)	–	1 (0.3)	–	–	–	10 (0.8)	–
3	India	44 (14.3)	2.9	156 (30.2)	3.2	168 (35.7)	6.4	135 (43.7)	5.1	87 (38.3)	3.4	524 (31.9)	4.5
4	Indonesia	52 (16.9)	2.3	72 (14.0)	1.4	44 (9.4)	2.0	32 (10.4)	0.9	31 (13.7)	1.4	214 (13.0)	1.6
5	Maldives	–	–	1 (0.2)	–	–	–	–	–	–	–	1 (0.06)	–
6	Myanmar	5 (1.6)	3	4 (0.8)	–	2 (0.4)	–	5 (1.6)	0.5	2 (0.9)	–	18 (1.1)	4
7	Nepal	13 (4.2)	1.7	21 (4.1)	19	24 (5.1)	10.0	19 (6.1)	7.5	5 (2.2)	–	73 (4.4)	7.1
8	DPR Korea	–	–	–	–	1 (0.2)	–	–	–	–	–	1 (0.06)	–
9	Sri Lanka	–	–	7 (1.4)	–	7 (1.5)	5.0	3 (1.0)	–	2 (0.9)	–	19 (1.1)	17
10	Thailand	72 (23.5)	1.1	146 (28.3)	0.5	111 (23.6)	2.4	72 (23.3)	0.9	58 (25.6)	1.2	407 (24.8)	1.1
11	Timor-Leste	–	–	1 (0.2)	–	1 (0.2)	–	–	–	1 (0.4)	–	3 (0.2)	–
12	SEAR	31 (10.1)	−0.6	41 (7.9)	−0.5	28 (6.0)	−0.6	17 (5.5)	−0.7	13 (5.7)	−0.4	116 (7.1)	−0.5
13	Asia^*^	5 (1.6)	3	6 (1.2)	−0.8	6 (1.3)	–	4 (1.3)	2.0	5 (2.2)	0.5	23 (1.4)	1.3
14	Global^#^	23 (7.5)	0.1	27 (5.2)	1.3	47 (10.0)	2.7	15 (4.9)	0.1	10 (4.4)	0	109 (6.6)	1.2

* Articles which were not exclusive to SEAR but included other Asian countries.

# Articles which were not exclusive to SEAR but included other countries globally.

DPR Korea (*n* = 1), Maldives (*n* = 1) and Timor Leste (*n* = 3) had the least output of research papers on influenza between 2000 and 2021.

### Common research areas within streams

Based on keywords, under stream 1, publications commonly related to surveillance and vaccines. In stream 2, infection, outbreak surveillance and transmission were commonly used keywords. For stream 3, vaccines, burden and epidemiological indicators dominated. Treatment, infections and antiviral drugs and therapies were the common key words in stream 4. Stream 5 majorly focused on developing newer methods for genetic analysis and monitoring. Thus, there were inequities within the streams of priority research (Table 3).

**Table 3:** Percentage^*^ of key words used in different streams.

Stream 1		Stream 2		Stream 3		Stream 4		Stream 5	
Key words	%	Key words	%	Key words	%	Key words	%	Key words	%
Surveillance^#^	48.1	Infection	39.3	Vaccine	74.1	Treatment	46.5	Analysis	44.9
Vaccine	18.1	Outbreak	36.8	Burden	20.6	Infections	26.6	Genetic	18.1
Biosecurity	8.8	Surveillance^^^	35.8	Incidence	14.7	Antiviral	20.5	Monitoring	7.8
Pathogenesis	6.3	Transmission	31.4	Hospitalization	10.1	Therapy	10.0	GIS Mapping	2.1
One health	5.9	Detection	14.2	Prevalence	9.7	Severity	8.2	Risk communication	0.0
Farming	5.0	Interventions	7.2	Epidemiology	9.5	Interventions	2.1	Community engagement	0.0
Diagnostic	2.2	Screening	5.1	Policy	11.3	Cost-effectiveness	1.2		
				Pandemic Preparedness	9.1				

* % here means proportion of articles that included the keyword within the respective stream.

# Stream 1 articles, i.e., animal related surveillance.

^ Stream 2 articles, i.e., human related surveillance.

Stream 1: Reducing the Risk of Emergence of Pandemic Influenza; Stream 2: Limiting the spread of pandemic, zoonotic and seasonal epidemic influenza; Stream 3: Minimizing the impact of pandemic, zoonotic and seasonal epidemic influenza; Stream 4: Optimizing the treatment of patients; and Stream 5: Promoting the development and application of modern public health tools.

**Figure 2:**
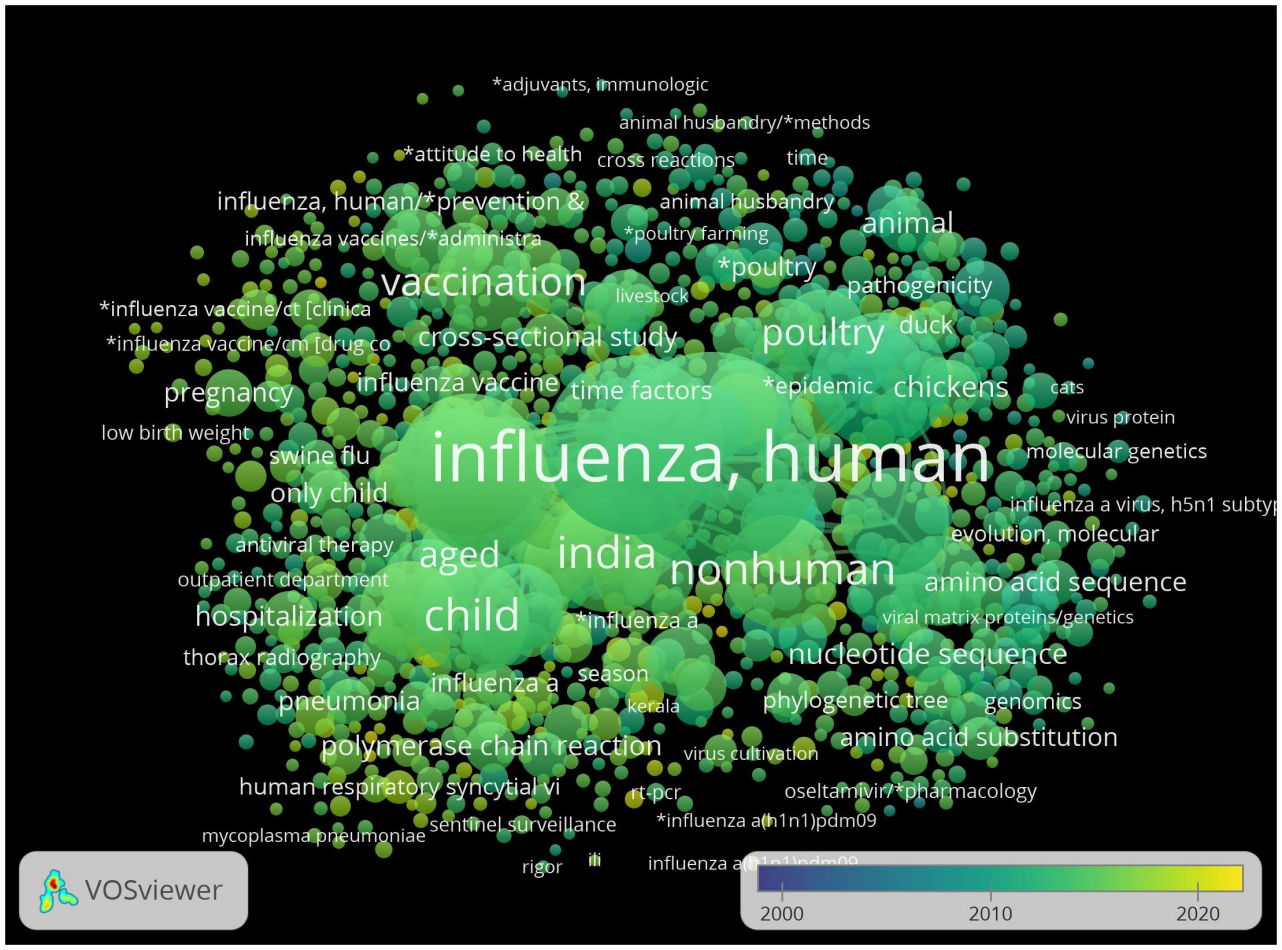
Overlay visualization of co-occurrence of keywords.

The network visualization of keywords generated by VOSviewer suggested that the commonly used keywords emerged around and after 2010. Thus, the scope of research on influenza in SEAR seems to have expanded since 2010 with new keywords coming into vogue (Figure 2).

### Types of research and study designs

**Figure 3:**
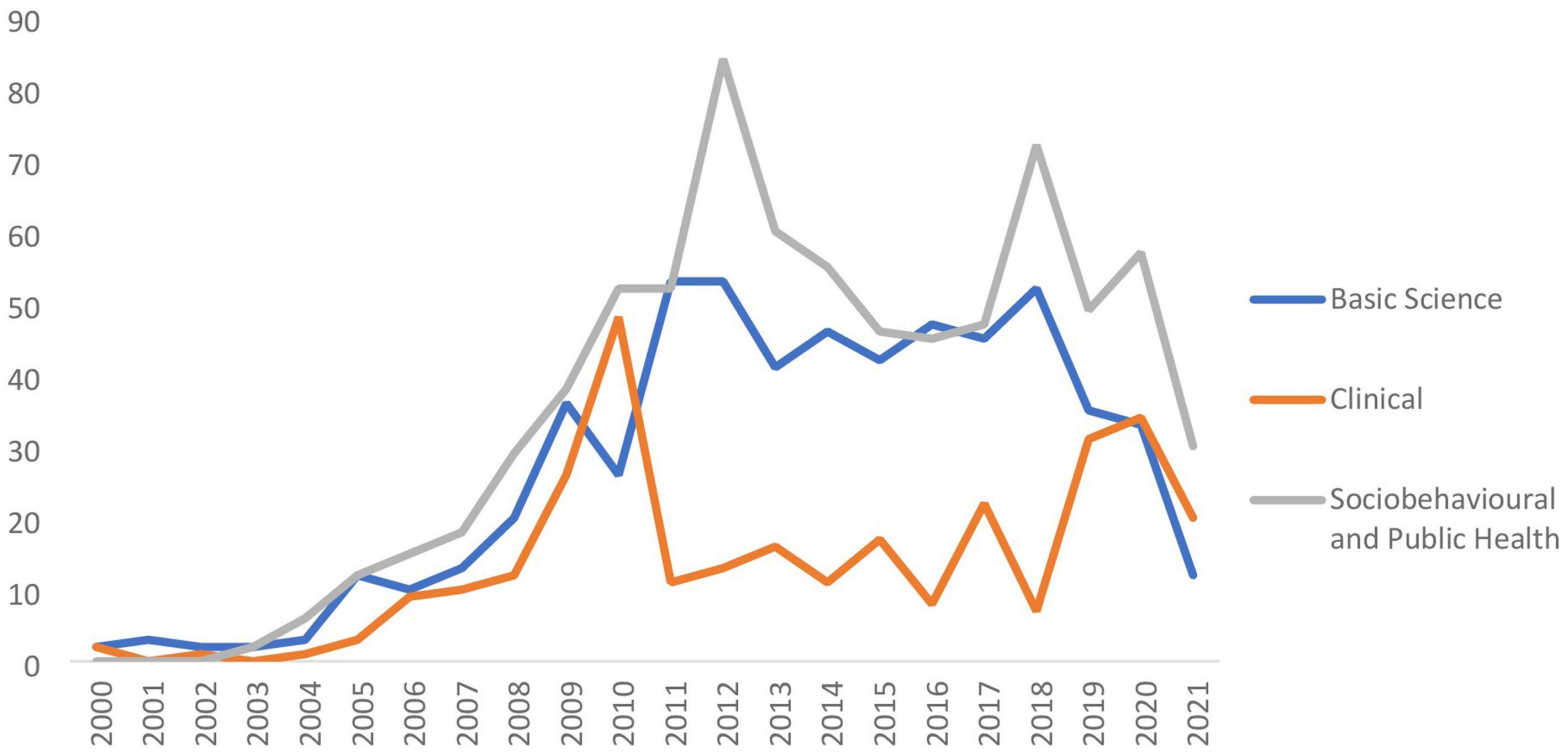
Trend of number of publications included in the review based on type of research.

The most common type of research was from the socio-behavioral and public health domain (46∙9% of all papers). Of the 410 articles published between 2000 and 2010, and 1,641 articles between 2011 and 2021, clinical research continued to lag behind and had shown the lowest decadal growth (2000–2010: n-112; 2011–2021: n-190; DGR: 0.7) as compared to the research from basic science (2000–2010: n-129; 2011–2021: n-459; DGR: 2.5), and socio-behavioral and public health themes (2000–2010: n-172; 2011–2021: n-597; DGR: 2.5; Figure 3). However, of late, since 2018, clinical research seemed to be catching up from the SEAR MS and basic science, and socio-behavioral and public health research declining in trend. There were very few articles (n-16) that combined more than one type of research (Supplementary File Table S1-3).

Based on the type of study design, observational studies (2000–2010: n-254; 2011–2021: n-713; DGR: 1.8) accounted for the highest number (n-967; 58∙9%) of papers while interventional designs (2000–2010: n-27; 2011–2021: n-66; DGR: 1.4) were used in only 93 (5∙7%) of the papers. Interventional studies also showed the lowest decadal growth rate (DGR = 1∙4) while bench research (2000–2010: n-129; 2011–2021: n-459) showed the maximal rate of growth (DGR = 2.5). There was minimal increase in the number of review articles produced from SEAR (2000–2010: n-78; 2011–2021: n-109; DGR = 0.4; Supplementary File Table S1-4).

### Journals and authors

Table 4 enlists the top 10 journals based on the number of articles published on influenza from SEAR. These journals had published 442 articles, i.e., 26∙9% of the total number of publications (n-1,641) from the Region. Most of the leading journals publishing influenza related research from SEAR were specialized journals focusing on specific fields of interest, e.g., on vaccines, infectious diseases, and animal health.

**Table 4:** List of top 10 Journals where Influenza-related research from South East Asia Region was published between 2000 and 2021.

S.no.	Journal	Total no. of influenza related publications included (*N* = 1,641)	h-index	Impact factor	SJR	SNIP	CiteScore
1	PloS one	94 (5.7)	367	3.75	0.85	1.37	5.6
2	Vaccine	62 (3.8)	191	4.17	1.39	1.30	6.7
3	Influenza and Other Respiratory Viruses	58 (3.5)	64	2.95	1.63	1.48	7.8
4	Emerging Infectious Diseases	48 (3.0)	240	16.13	3.67	2.36	9.8
5	International Journal of Infectious Diseases	43 (2.6)	104	3.62	2.43	2.49	10.8
6	American Journal of Tropical Medicine and Hygiene	39 (2.4)	157	3.70	1.01	1.10	4.0
7	Preventive veterinary medicine	29 (1.8)	100	3.42	0.67	1.32	4.1
8	Transboundary and Emerging Diseases	25 (1.5)	70	5.00	0.95	1.69	8.6
9	Southeast Asian Journal of Tropical Medicine and Public Health	24 (1.5)	55	0.26	0.13	0.26	0.8
10	Avian Diseases	20 (1.2)	83	1.73	0.48	0.89	2.4

h-index: The h-index attempts to measure the productivity of a researcher/journal and the citation impact of their publications. Impact factor: It is the ratio of number of citations in 1 year to content published in the previous 2 years to the number of articles and reviews published within the previous 2 years. SJR: SCImago Journal Rank aims to capture the effect the subject field, quality, and reputation of a journal has on a citation. It looks at the prestige of a journal by considering the sources of citations to it, rather than counting all citations equally. Each citation received by a journal is assigned a weight based on the SJR of the citing journal. SNIP: Source Normalized Impact per Paper measures citations received relative to citations expected for the subject field. CiteScore: CiteScore measures average citations received per document published. It is a ratio of citations to research published, and looks at all content published in a journal (not just articles and reviews).

* All the journal metrics are as of 2021 collated from different sources on the internet.

PLoS One, a multi-disciplinary open-access journal catering to a wider profile of readers accounted for the maximum number of papers from the Region, which also had the highest h-index among all the journals indicating a higher citation impact. Overall, from the given journal metrics, highest number of publications were published in journals having h-index ranging between 55 and 367 and impact factor ranging between 0.26 and 16.13. Taking all the metrics into consideration, “Emerging Infectious Diseases” was among the top, however, it was the 4th journal when number of influenza related publications were considered. Among the journals publishing influenza related research from SEAR, “International Journal of Infectious Diseases,” “Emerging Infectious Diseases,” and “Transboundary and Emerging Diseases” topped the list according to their CiteScores.

The top 20 authors in the influenza research based on the total link strength are presented in Table 5. Of the 20 authors in this list, only 10 were from SEAR MS. These were from Bangladesh (n-4), India (n-4), and Thailand (n-2). These also featured in the major groups collaborating for research on Influenza from SEAR. The other 10 authors in the top 20 list, 09 were from United States of America (United States) and 01 from Denmark. Majority of these researchers were affiliated to state-supported dedicated research laboratories, e.g., International Centre for Diarrheal Disease Research (Bangladesh), National Institute of Virology (India), and the Centers for Disease Control and Prevention (CDC United States). The list of top authors and network visualization of their cluster for Bangladesh, India, Indonesia, Nepal, Thailand was also captured and presented in Supplementary File #2.

**Table 5:** List of top 20 authors contributing to South East Asian Region related influenza research between 2000 and 2021, based on their total link strength.

S.no.	Author	h-index^#^	Total link strength^*^	Affiliated institution	Country	No. of influenza related publications
1	Rahman, M.	63	249	International Centre for Diarrheal Disease Research	Bangladesh	40
2	Chadha, M.S.	38	201	National Institute of Virology	India	50
3	Widdowson, M.-A.	53	198	Centers for Disease Control and Prevention (CDC), Atlanta, Georgia	United States	25
4	Azziz-Baumgartner, E.	40	190	International Centre for Diarrheal Disease Research	Bangladesh	27
5	Amonsin, A.	32	182	Faculty of Veterinary Science, Chulalongkorn University	Thailand	44
6	Katz, J.M.	87	162	Centers for Disease Control and Prevention (CDC), Atlanta, Georgia	United States	23
7	Dawood, F.S.	24	156	Centers for Disease Control and Prevention, Atlanta, Georgia	United States	30
8	Chittaganpitch, M.	30	149	National Institutes of Health	Thailand	42
9	Luby, S.P.	68	146	Stanford University	United States	20
10	Saha, S.	19	145	Centers for Disease Control and Prevention, Atlanta, Georgia	United States	19
11	Bresee, J.	90	144	Centers for Disease Control and Prevention, Atlanta, Georgia	United States	17
12	Olsen, S.J.	53	143	WHO Regional Office for Europe	Denmark	22
13	Webster, R. G.	144	143	St. Jude Children’s Research Hospital	United States	29
14	Sturm-Ramirez, K.	25	140	International Centre for Diarrheal Disease Research	Bangladesh	17
15	Broor, S.	37	133	Shree Guru Gobind Singh Tricentenary University	India	30
16	Lal, R.B.	45	130	Centers for Disease Control and Prevention, Atlanta, Georgia	United States	20
17	Krishnan, A.	35	129	All India Institute of Medical Sciences, New Delhi	India	24
18	Leclerq, S.C.	7	120	Nepal Nutrition Intervention Project-Sarlahi	India	19
19	Englund, J.A.	74	119	Seattle Children’s Research Institute	United States	20
20	Feeroz, M.M.	19	114	Jahangirnagar University	Bangladesh	16

# Source: Scopus.

* Total link strength: Links attribute indicates the number of co-authorship links of a given researcher with other researchers. Total link strength indicates the total strength of the co-authorship links of a given researcher with other researchers.

**Figure 4:**
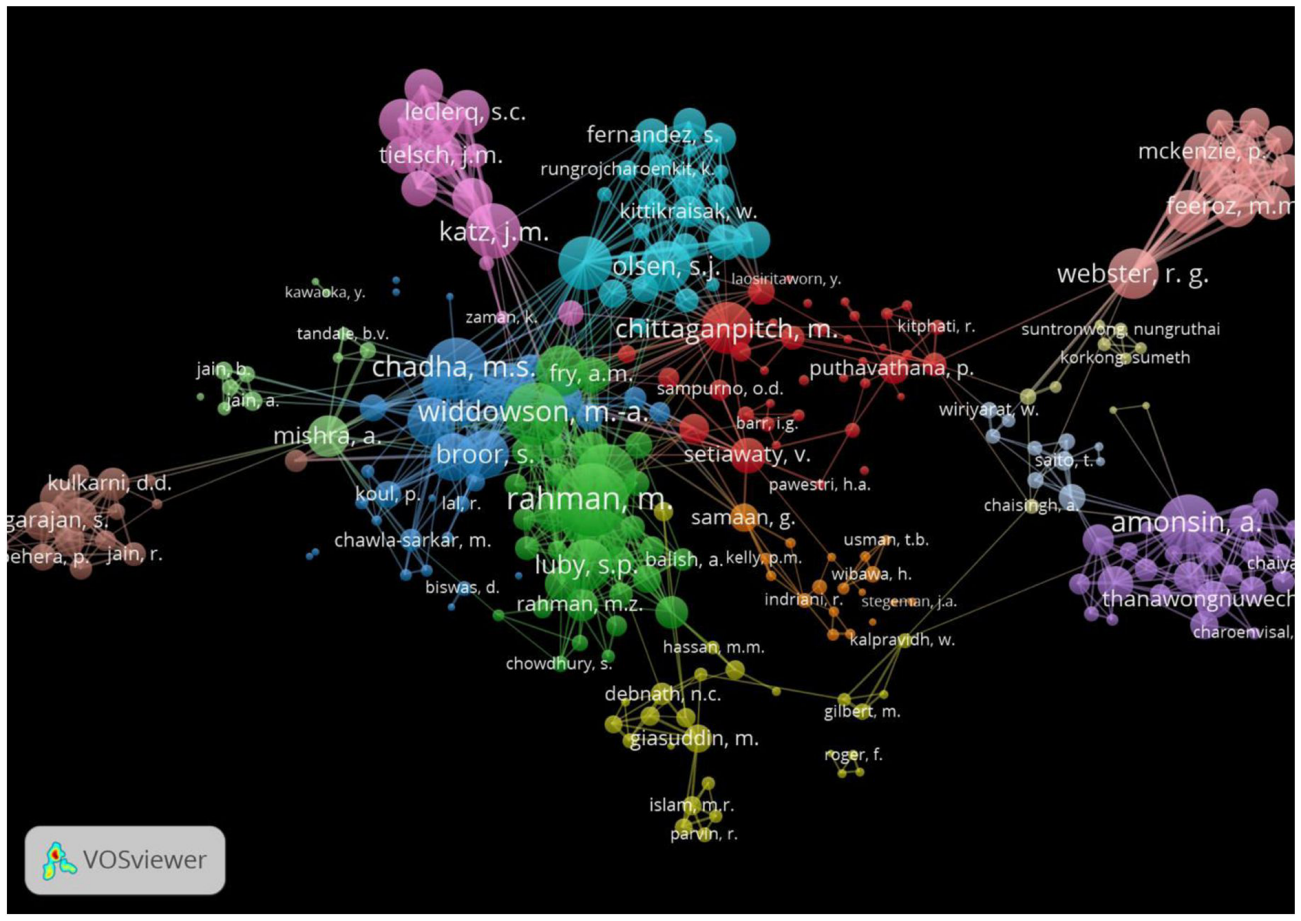
Network visualization of connectivity of authors (Global).

The network visualization generated by VOSviewer suggested that there were about 8–9 cluster of authors that accounted for most of the Influenza related research output from SEAR. About 50% of these clusters worked in close collaboration with one another. These included clusters from India, Bangladesh, Thailand, United States, and Denmark (Figure 4).

Publication trend in different SEAR countries represented in diagram and stacked plots have been presented in Supplementary File #3.

## Discussion

We conducted a bibliometric analysis of a comprehensive compendium of literature on influenza from SEAR MS to detect the trends in research outputs between 2000 and 2021, its alignment with WHO research priority streams, and to unravel gaps and inequities therein. Research output on influenza from SEAR MS had increased exponentially over the years. Notwithstanding these increases, one area that WHO needs to discuss in its annual regional policy and strategic dialog with MS is the noted inequities in research output between SEAR MS, and within and across the five priority streams identified by WHO.

### Research output

It is encouraging to note that India and Thailand in SEAR had sustained research output for influenza, while Bangladesh, Nepal and Sri Lanka showed promising decadal growth in research output. However, in the context of rolling out the Global Influenza Strategy (2019–2030) in SEAR, in particular, one of its key objectives namely promoting research and innovation to address unmet public health needs, the finding that, six SEAR countries, i.e., Myanmar, Sri Lanka, Bhutan, Timor-Leste, Maldives, DPR Korea lagged behind the others in research output for influenza is of paramount importance. It calls for WHO SEAR Office to understand the reasons for and work out strategies jointly with these MS to advance the research agenda.

Another policy implication of our analysis is that a considerable amount of research on influenza from SEAR was conducted through ‘foreign’ researchers and institutions either as exclusive or collaborative outputs. For example, for Nepal, Bangladesh and India, much of the research output for influenza came from collaboration with researchers from CDC, Atlanta and NIH, United States. Paradoxically, presence of these collaborative partners was not prominent in the six SEAR MS enlisted above for lower research output. Thus, there is a conspicuous inequity in terms of collaborative opportunity/ capacity for research on Influenza among the SEAR MS. It leads to an action point for WHO as the secretariat to see how WHO can facilitate collaborations between centers of excellence and MS to scale up joint research in these countries.

SEARO has to leverage the global experience that international collaborations are likely to produce more literature in low-middle income countries (LMICs) settings by mobilizing funds and institutional capacity for research from high income countries (HICs), by identifying novel questions and designing innovative research studies, and by complementing/ building capacity among researchers in LMICs (13). However, it has also to be cognizant of the fact that HIC-LMIC collaborations have been criticized for diverging from local research priorities and for not being able to build equitable capacities among the collaborating groups in LMICs (14). Developing a framework of priority research for the region and countries within the WHO influenza research priority framework will help mitigating the above stated risk. The other important policy implication is underscoring the need to review the impact of these strategic partnerships on building indigenous research capacity in SEAR MS and also establishing mechanisms for strengthening within-region collaborations for influenza research (15).

Regional consultations with MS, targeted allocation of funds for priority research areas in major donor supported funding proposals and advocating for Influenza specific research in the biennium work plan of Influenza teams at SEARO and WHO country offices (WCOs) under influenza research agenda are critical requirements. They enable SEARO and WCOs to develop country priority research implementation plans for encouraging collaborative research capacity within the region. It also encourages pursuing a context-driven research agenda in the region guided by the WHO’s Influenza priority research streams (16–19). Lessons can be learned from implementation of the regional priority research agenda in relation to COVID-19 in SEAR and other WHO regions (20–22). Emphasizing South–South Co-operation, countries like India, Bangladesh and Thailand could hand-hold the MS that lag behind in this regard. Since dedicated research laboratories are the major producers of evidence on influenza from the region, facilitating establishment of such centers in the MS with institutional mentorship support and scientist exchange programs along with funding support to young researchers to facilitate them build career tracks in influenza research could be one strategic action that could be taken up with MS in WHO’s Regional consultations.

Lessons learned from COVID-19 pandemic response indicated the need for more focus on clinical management aspects in preparedness phase for future pandemics due to respiratory borne pathogens with enhanced future efforts on intensive care of patients (23–25). In this context, the increasing trends in publications from clinical research and reviews on influenza from SEAR since 2015 are encouraging and useful for country focused infrastructure and capacity development. This was most evident for India, Indonesia, Bangladesh and Thailand. The trend was also obvious when the 6 low-research-output countries were aggregated (Supplementary File #3). We also observed that basic science related research output from SEAR was on a downtrend and required attention.

WHO SEAR Office would require reviews in SEAR MS for exploring reasons for such emerging trends to gauge future directions and inform strategic planning. The five-year project implemented by SEARO titled “Surveillance and Response to Seasonal and Pandemic Influenza” in SEAR could be an appropriate platform (26). From a policy perspective, the finding of an upsurgence in clinical research and reviews could indicate an emerging appreciation for evidence-based practice. Continued focus on clinical research needs encouragement in all SEAR MS. Reviews are operationally feasible in all MS given that they are also relatively less resource intensive as compared to field or basic science laboratory-based research, and hence more “doable” (27).

### Tracking research output from SEAR

Beyond a quarter of the publications on Influenza from SEAR got published in 10 journals suggesting that access to these journals was key to dissemination and development of science on Influenza from the region. We also noted that substantial papers were attributable to a handful of authors and author groups. This finding is operationally useful for WHO’s country support plans given the fact that WHO is involved in regional capacity enhancement and building cohorts of regional experts for cross-country support. Thus, the progress in scientific knowhow on influenza in SEAR could be significantly influenced by these authors and author groups and tracked by following outputs from their laboratories. Given that a major proportion of literature was published in specialized journals, there is a need to identify and leverage platforms for inter-disciplinary communication of evidence.

### Priority research streams vs. conducted research

Countries seem to have inconsistent capacity to pursue research across all five priority streams. The found gaps in research outputs notable within the streams are appropriate for WHO to advocate for filling them and where relevant, to collaborate through their biennium workplans with MS. In stream 1, the limited research focus on biosecurity, flu pathogenesis, safe farming practices, one health, veterinary diagnostic kits underscores the need for accelerating research in these areas to strengthen evidence informed interventions pertinent to one health including those in the human animal interface. Simultaneously, the limited focus on research related to effective non-pharmaceutical interventions and mass screening strategies (Stream 2) and policy and planning for pandemic preparedness (Stream 3) could be an indication for planning research for selecting evidence informed actions for preparedness for pandemics and large outbreaks. These research questions could benefit from research involving above highlighted strategies implemented for responding to the COVID-19 pandemic in SEAR MS. In stream 4, research on pharmacological interventions across disease severity profiles and on cost-effectiveness of the same, needed impetus. Stream 5 (promoting the development and application of new public health tools), in particular, needed attention as these could provide contextual and actionable guidance for data monitoring, geo-epidemiological mapping, and risk communication and community engagement. As the region is aggressively pursuing implementation of the Global Influenza Strategy, strengthening stream 5 will contribute to better influenza tools which is one of the two core outcomes of the global strategy (28).

The disparity in the performance of the MS in terms of research output between and within priority streams coincided with relatively low number of articles produced by most MS; thus, trends elicited may not be consistent enough for these countries. Also, the current public health situation for priorities in influenza research in SEAR MS, especially in the aftermath of the COVID-19 pandemic waves, is undecided. Nevertheless, most SEAR MS needed to increase the number of papers that they produce while ensuring that these addressed critical knowledge gaps within and across the priority streams. Where possible, integrating with current research on COVID-19 pandemic will ease implementation of influenza research while COVID-19 is still in progress overwhelming scarce human resources in health.

The fact that much of the research evidence was, possibly, of lower ‘strength on the hierarchy of evidence’ since only a few studies utilized experimental design is an area for improvement in the region; however, we must admit that we have not assessed the quality of the articles. Very few articles combined basic science, clinical and socio-behavioral and public health research.

One major challenge for MS as well as WHO is to make strategic efforts for sector-wide engagement. For example, inclusion of departments of animal health, environment and agriculture is an essential One Health approach for generating critical evidence. This, however, would require commitment, highest level support in countries for sector wide coordination and differentiated investments in research capacity building across these sectors while working toward inclusive designing of priority research agenda.

### Closing the research-policy gap

There has been encouraging progress in SEAR for influenza sentinel surveillance over the past years (29, 30). Global Influenza Surveillance and Response System (GISRS) is well established in SEAR, and virological data are collected, analyzed and influenza viruses are shared to determine recommendations for vaccine composition. Eight of the 11 SEAR MS have WHO-recognized national influenza centers (NICs). A WHO reference laboratory for influenza A(H5) has also been established in the region (NIV, Pune India). Moreover, considering the increasing emerging need, animal surveillance for Highly Pathogenic Avian Influenza among wild birds, poultry, pigs and other animals has also evolved on national scales (31). The landscape of technical and financial support MS has also changed over time (32, 33).

In SEAR MS, these recent progress in surveillance and laboratory capacity have produced valuable surveillance data and consequent publications (34, 35). However, with limited evidence from interventional (including implementation) designs, it is unclear how academia and program managers are converging their efforts for effective program designing. While it has been argued that pandemic influenza preparedness plans must include strategic virological and epidemiological uncertainty and cannot entirely rely on evidence-based policy making, a consideration of local vulnerabilities, resources, attributes of health system and public perception of risk and management strategies, is also important for effective pandemic influenza risk management (PIRM). This contextualization, however, could compromise the inter-operability of national preparedness plans (36).

Based on the findings of the current research and also interventions and learnings from the COVID-19 pandemic, there is a need to revisit influenza research priorities in SEAR. Some of the emergent research questions in contemporary times include research for efficient health financing, preparedness planning amidst geo-political volatility, exploration of partnerships with existing and new stakeholders at sub-national, national and international levels, and building surge capacity (37). Information management is yet another theme that has emerged as a challenge during the pandemic. Leveraging the existent regional research forum, WHO Collaborating Centers and disease networks among MS, should be some of the considerations for designing and implementing a coherent priority research agenda for influenza in SEAR.

## Limitations

Our study has several limitations. We reviewed only peer-reviewed literature published in English. We understand that manuscripts are not the only (and perhaps, not the most common either) vehicle for knowledge exchange and translation. Policy and technical briefs, consultation meetings, and conference proceedings are popular methods for academic dissemination and evidence communication. SEAR MS could also be producing literature, both peer-reviewed and gray, in local languages. These have been excluded from our focus. Having a central repository of research evidence could help in cross-learning and utilization of evidence by the MS so that policy making and program designing could be well-informed and harmonized. The COVID-19 pandemic seems to have had an impact on the volume of literature produced by research teams world-wide. We have not examined the trends during the pandemic.

The ongoing COVID-19 pandemic underscores that countries must evolve indigenous capacity for pandemic preparedness and response against infectious diseases and especially for those presenting with influenza-like illness. It is also likely that the pandemic could have disrupted the influenza-research ecosystem in ways that are yet to be examined – some research avenues could have become obsolete while new opportunities of integrated research might have come up. There is a need to reconsider which areas of research must be prioritized in the aftermath of the pandemic and how these could be best supported. Nevertheless, member states must inculcate a culture of within and inter-country collaboration to produce evidence that has regional as well as global value.

## Author contributions

PR, MK, and AM: conceptualization, resources, and validation. DS, BV, RM, PP, and AM: data curation and writing—original draft. AM, DS, BV, MK, and PR: formal analysis. PR and MK: fund acquisition. AM, MK, PR, NB, EC, DS, BV, RM, and PP: investigation and writing—review and editing. MK and AM: methodology, project administration, and supervision. DS and BV: software. DS, BV, RM, and PP: visualization. All authors contributed to the article and approved the submitted version.

## Funding

This study was supported from the PIP Partnership contributions funds under the WHP PIP work plan of the 2020–21. Under the HLIP-II, WHO SEARO is managing the PIP PC project in the region and this activity was conducted under the approved work plan (2020–2021) by Partnership Contribution Independent Technical Expert Mechanism (PCITEM).

## Conflict of interest

The authors declare that the research was conducted in the absence of any commercial or financial relationships that could be construed as a potential conflict of interest.

## Publisher’s note

All claims expressed in this article are solely those of the authors and do not necessarily represent those of their affiliated organizations, or those of the publisher, the editors and the reviewers. Any product that may be evaluated in this article, or claim that may be made by its manufacturer, is not guaranteed or endorsed by the publisher.

## Footnotes

^1^ https://www.rayyan.ai/
