# Supplementary material for: An appraisal of peer-reviewed published literature on Influenza, 2000–2021 from countries in South-East Asia Region
Source: Front Public Health. 2023 Apr 17;11:1127891. doi: 10.3389/fpubh.2023.1127891 (PMC10149947; doi:10.3389/fpubh.2023.1127891)
Supplement: Supplementary file 1 [file Data_Sheet_1.pdf]

Table of Contents

Supplementary File #1 4
Table S1-1. Search Strategy 4
Table S1-2. Distribution of articles included in the review according to SEAR member countries 5
Table S1-3. Distribution of articles included in the review according to type of research 6
Table S1-4. Distribution of articles included in the review according to study design 7
Figure S1-1: Trend of number of publications included in the review based on study design 7
Supplementary File #2 8
Figure S2-1: Network visualization of connectivity of authors (Bangladesh) 8
Table S2-1. List of top authors contributing to Influenza research in Bangladesh 8
Figure S2-2: Network visualization of connectivity of authors (India) 9
Table S2-2. List of top authors contributing to Influenza research in India 9
Figure S2-3: Network visualization of connectivity of authors (Indonesia) 10
Table S2-3. List of top authors contributing to Influenza research in Indonesia 11
Figure S2-4: Network visualization of connectivity of authors (Nepal) 12
Table S2-4. List of top authors contributing to Influenza research in Nepal 12
Figure S2-5: Network visualization of connectivity of authors (Thailand) 13
Figure S2-6: Network visualization of connectivity of authors (Thailand) 14
Table S2-5. List of top authors contributing to Influenza research in Thailand 14
Supplementary File #3 15
Figure S3-1: Trend of number of publications of Bangladesh based on WHO influenza streams 15
Figure S3-2: Stacked plot of number of publications of Bangladesh based on WHO influenza streams 15
Figure S3-3: Trend of number of publications of Bangladesh based on type of research 16
Figure S3-4: Trend of number of publications of Bangladesh based on study design 16
Figure S3-5: Trend of number of publications of Bhutan based on WHO influenza streams 17
Figure S3-6: Stacked plot of number of publications of Bhutan based on WHO influenza streams 17
Figure S3-7: Trend of number of publications of Bhutan based on type of research 18
Figure S3-8: Trend of number of publications of Bhutan based on study design 18
Figure S3-9: Trend of number of publications of DPR Korea based on WHO influenza streams 19

Figure S3-10: Stacked plot of number of publications of DPR Korea based on WHO influenza streams 19
Figure S3-11: Trend of number of publications of DPR Korea based on type of research .20
Figure S3-12: Trend of number of publications of DPR Korea based on study design 20
Figure S3-13: Trend of number of publications of India based on WHO influenza streams 21
Figure S3-17: Trend of number of publications of Indonesia based on WHO influenza streams 23
Figure S3-18: Stacked plot of number of publications of Indonesia based on WHO influenza streams 23
Figure S3-19: Trend of number of publications of Indonesia based on type of research....24
Figure S3-20: Trend of number of publications of Indonesia based on study design 24
Figure S3-21: Trend of number of publications of Maldives based on WHO influenza streams 25
Figure S3-22: Stacked plot of number of publications of Maldives based on WHO influenza streams 25
Figure S3-23: Trend of number of publications of Maldives based on type of research.....26
Figure S3-24: Trend of number of publications of Bangladesh based on study design 26
Figure S3-25: Trend of number of publications of Myanmar based on WHO influenza streams 27
Figure S3-26: Stacked plot of number of publications of Myanmar based on WHO influenza streams 27
Figure S3-27: Trend of number of publications of Myanmar based on type of research....28
Figure S3-28: Trend of number of publications of Myanmar based on study design 28
Figure S3-29: Trend of number of publications of Nepal based on WHO influenza streams 29
Figure S3-30: Stacked plot of number of publications of Nepal based on WHO influenza streams 29
Figure S3-31: Trend of number of publications of Nepal based on type of research..........30
Figure S3-32: Trend of number of publications of Nepal based on study design 30
Figure S3-33: Trend of number of publications of Sri Lanka based on WHO influenza streams 31
Figure S3-34: Stacked plot of number of publications of Sri Lanka based on WHO influenza streams 31
Figure S3-35: Trend of number of publications of Sri Lanka based on type of research....32
Figure S3-36: Trend of number of publications of Sri Lanka based on study design 32
Figure S3-37: Trend of number of publications of Thailand based on WHO influenza streams 33

Figure S3-38: Stacked plot of number of publications of Thailand based on WHO influenza streams 33
Figure S3-39: Trend of number of publications of Thailand based on type of research 34
Figure S3-40: Trend of number of publications of Thailand based on study design...........34
Figure S3-41: Trend of number of publications of Timor-Leste based on WHO influenza streams 35
Figure S3-42: Stacked plot of number of publications of Timor-Leste based on WHO influenza streams 35
Figure S3-43: Trend of number of publications of Timor-Leste based on type of research36
Figure S3-44: Trend of number of publications of Timor-Leste based on study design.....36
Figure S3-45: Trend of number of publications of 6 SEAR countries which are below 50th percentile based on WHO influenza streams 37
Figure S3-46: Stacked Plot of number of publications of 6 SEAR countries which are below 50th percentile based on WHO influenza streams 37
Figure S3-47: Trend of number of publications of 6 SEAR countries which are below 50th percentile based on type of research 38
Figure S3-48: Trend of number of publications of 6 SEAR countries, which are below 50th percentile based on study design..38
Supplementary File #4..39
Figure S4-1: Flow diagram showing the selection and inclusion of the studies.................39
File S4-2: Data extraction form 40

Supplementary File #1

Table S1-1. Search Strategy

PUBMED
("South Asia"[Title/Abstract] OR "South-East Asia"[Title/Abstract] OR "Southeast Asia"[Title/Abstract] OR "South-East Asia Region"[Title/Abstract] OR "Southeast Asia Region"[Title/Abstract] OR "SEAR"[Title/Abstract] OR Bangladesh[Title/Abstract] OR India[Title/Abstract] OR Indonesia[Title/Abstract] OR Nepal[Title/Abstract] OR "Sri Lanka"[Title/Abstract] OR Thailand[Title/Abstract] OR Bhutan[Title/Abstract] OR "DPR Korea"[Title/Abstract] OR "North Korea"[Title/Abstract] OR Myanmar[Title/Abstract] OR Burma[Title/Abstract] OR Maldives[Title/Abstract] OR "Timor-Leste"[Title/Abstract] OR "Timor Leste"[Title/Abstract]) AND (Influenza OR parainfluenza OR flu OR Neuraminidase OR Haemagglutinin OR Hemagglutinin OR (Haemagglutinin[MeSH Terms]) OR (Neuraminidase[MeSH Terms]) OR orthomyxoviridae [MeSH] OR "Orthomyxoviridae Infections"[Mesh] NOT (isavirus[MeSH Terms]) NOT (thogotovirus[MeSH Terms])) Filters: English, from 2000/1/1 - 2021/12/31
Result: 2965

SCOPUS
TITLE-ABS-KEY ("South Asia" OR "South-East Asia" OR "Southeast Asia" OR "South-East Asia Region" OR "Southeast Asia Region" OR "SEAR" OR bangladesh OR india OR indonesia OR nepal OR "Sri Lanka" OR thailand OR bhutan OR "DPR Korea" OR "North Korea" OR myanmar OR burma OR maldives OR "Timor-Leste" OR "Timor Leste") AND ALL (influenza OR parainfluenza OR flu OR haemagglutinin OR hemagglutinin OR neuraminidase OR "Orthomyxoviridae Infections" OR orthomyxoviridae) AND NOT (isavirus AND thogotovirus)
(LIMIT-TO (PUBYEAR , 2021) OR LIMIT- TO (PUBYEAR , 2020) OR LIMIT-TO (PUBYEAR , 2019) OR LIMIT-TO (PUBYEAR , 2018) OR LIMIT-TO (PUBYEAR , 2017) OR LIMIT-TO (PUBYEAR , 2016) OR LIMIT-TO (PUBYEAR , 2015) OR LIMIT-TO (PUBYEAR , 2014) OR LIMIT-TO (PUBYEAR , 2013) OR LIMIT-TO (PUBYEAR , 2012) OR LIMIT-TO (PUBYEAR , 2011) OR LIMIT-TO (PUBYEAR , 2010) OR LIMIT-TO (PUBYEAR , 2009) OR LIMIT-TO (PUBYEAR , 2008) OR LIMIT-TO (PUBYEAR , 2007) OR LIMIT-TO (PUBYEAR , 2004) OR LIMIT-TO (PUBYEAR , 2003) OR LIMIT-TO (PUBYEAR , 2002) OR LIMIT-TO (PUBYEAR , 2001) OR LIMIT-TO (PUBYEAR , 2000)) AND (LIMIT-TO (LANGUAGE , "English")) AND (LIMIT-TO (SUBJAREA , "MEDI") OR LIMIT-TO (SUBJAREA , "BIOC") OR LIMIT-TO (SUBJAREA , "VETE") OR LIMIT-TO (SUBJAREA , "ENVI") OR LIMIT-TO (SUBJAREA , "NURS") OR LIMIT-TO (SUBJAREA , "HEAL") OR LIMIT-TO (SUBJAREA , "DENT"))
Result: 5724

EMBASE
South Asia.m_titl. OR South-East Asia.m_titl.OR Southeast Asia.m_titl. OR South-East Asia Region.m_titl. OR Southeast Asia Region.m_titl. OR SEAR.m_titl. OR Bangladesh.m_titl. OR India.m_titl. OR Indonesia.m_titl. OR Nepal.m_titl. OR Sri Lanka.m_titl. OR Thailand.m_titl. OR Bhutan.m_titl. OR DPR Korea.m_titl. OR North Korea.m_titl. OR Myanmar.m_titl. OR Burma.m_titl. OR Maldives.m_titl. OR Timor-Leste.m_titl. OR Timor Leste.m_titl. AND influenza.mp OR parainfluenza.mp. OR flu.mp OR Hemagglutinin.mp OR Haemagglutinin.mp. or exp hemagglutinin/ OR Neuraminidase.mp. OR exp orthomyxoviridae/ or exp influenza virus/limit to (english language and yr="2000 - 2021")
Result: 1462

COCHRANE
(“South Asia”):ti,ab,kw OR ("South-East Asia"):ti,ab,kw OR (“Southeast Asia”):ti,ab,kw OR (“South-East Asia Region”):ti,ab,kw OR (“Southeast Asia Region”):ti,ab,kw OR (“SEAR”):ti,ab,kw OR (Bangladesh):ti,ab,kw OR (India):ti,ab,kw OR (Indonesia):ti,ab,kw OR (Nepal):ti,ab,kw OR (“Sri Lanka”):ti,ab,kw OR (Thailand):ti,ab,kw OR (Bhutan):ti,ab,kw OR (“DPR Korea”):ti,ab,kw OR (“North Korea”):ti,ab,kw OR (Myanmar):ti,ab,kw OR (Burma):ti,ab,kw OR (Maldives):ti,ab,kw OR (“Timor-Leste”):ti,ab,kw OR (“Timor Leste”):ti,ab,kw
AND (Influenza) OR (parainfluenza) OR (flu) OR (Haemagglutinin) OR (Hemagglutinin) OR (Neuraminidase) OR MeSH descriptor: [Neuraminidase] this term only OR MeSH descriptor: [Orthomyxoviridae] explode all trees OR MeSH descriptor: [Orthomyxoviridae Infections] this term only
Date limiter: Jan 2000 to Dec 2021
Result: 304

Table S1-2. Distribution of articles included in the review according to SEAR member countries

S.no	Region	Reducing the risk of emergence of a pandemic influenza				Limiting the spread of pandemic, zoonotic, and seasonal epidemic influenza				Minimizing the impact of pandemic, zoonotic and seasonal epidemic influenza				Optimizing the treatment of patients				Promoting the development and application of new public health tools				Overall			
		2000-2010 N=85 (%)	2011-2021 N=222 (%)	**Total N=307 (%)**	**DGR**	2000-2010 N=154 (%)	2011-2021 N=362 (%)	**Total N=516 (%)**	**DGR**	2000-2010 N=97 (%)	2011-2021 N=377 (%)	**Total N=470 (%)**	**DGR**	2000-2010 N=80 (%)	2011-2021 N=229 (%)	**Total N=309(%)**	**DGR**	2000-2010 N=60 (%)	2011-2021 N=167 (%)	**Total N=227 (%)**	**DGR**	2000-2010 N=410 (%)	2011-2021 N=1231 (%)	**Total N=1641 (%)**	**DGR**
1	Bangladesh	3 (3·5)	58 (26·1)	**61 (19·9)**	**18·3**	6 (3·9)	40 (11·0)	**46 (8·9)**	**5·7**	4 (4·1)	33 (8·8)	**37 (7·9)**	**7·3**	2 (2·5)	12 (5·2)	**14 (4·5)**	**5**	2 (3·3)	15 (9·0)	**17 (7·5)**	**6·5**	14 (3·4)	144 (11·7)	**158 (9·6)**	**9·3**
2	Bhutan	-	4 (1·8)	**4 (1·3)**	-	-	3 (0·8)	**3 (0·6)**	-	-	3 (0·8)	**3 (0·6)**	-	-	1 (0·4)	**1 (0·3)**	-	-	-	-	-	-	10 (0·8)	**10 (0·8)**	-
3	India	9 (10·6)	35 (15·8)	**44 (14·3)**	**2·9**	30 (19·5)	126 (34·8)	**156 (30·2)**	**3·2**	20 (20·6)	148 (39·3)	**168 (35·7)**	**6·4**	19 (23·8)	116 (50·7)	**135 (43·7)**	**5·1**	16 (26·7)	71 (42·5)	**87 (38·3)**	**3·4**	80 (19·5)	444 (36·1)	**524 (31·9)**	**4·5**
4	Indonesia	12 (14·1)	40 (18·0)	**52 (16·9)**	**2·3**	21 (13·6)	51 (14·1)	**72 (14·0)**	**1·4**	11 (11·3)	33 (8·8)	**44 (9·4)**	**2·0**	11 (13·8)	21 (9·2)	**32 (10·4)**	**0·9**	9 (15·0)	22 (13·2)	**31 (13·7)**	**1·4**	59 (14·4)	155 (12·6)	**214 (13·0)**	**1·6**
5	Maldives	-	-	-	-	-	1 (0·3)	**1 (0·2)**	-	-	-	-	-	-	-	-	-	-	-	-	-	-	1 (0·1)	**1 (0·06)**	-
6	Myanmar	1 (1·2)	4 (1·8)	**5 (1·6)**	**3**	-	4 (1·1)	**4 (0·8)**	-	-	2 (0·5)	**2 (0·4)**	-	2 (2·5)	3 (1·3)	**5 (1·6)**	**0·5**	-	2 (1·2)	**2 (0·9)**	-	3 (0·7)	15 (1·2)	**18 (1·1)**	**4**
7	Nepal	3 (3·5)	8 (3·6)	**13 (4·2)**	**1·7**	1 (0·6)	20 (5·5)	**21 (4·1)**	**19**	2 (2·1)	22 (5·8)	**24 (5·1)**	**10·0**	2 (2·5)	17 (7·4)	**19 (6·1)**	**7·5**	-	5 (3·0)	**5 (2·2)**	-	8 (2·0)	65 (1·3)	**73 (4·4)**	**7·1**
8	DPR Korea	-	-	-	-	-	-	-	-	-	1 (0·3)	**1 (0·2)**	-	-	-	-	-	-	-	-	-	-	1 (0·1)	**1 (0·06)**	**0**
9	Sri Lanka	-	-	-	-	-	7 (1·9)	**7 (1·4)**	-	1 (1·0)	6 (1·6)	**7 (1·5)**	**5·0**	-	3 (1·3)	**3 (1·0)**	-	-	2 (1·2)	**2 (0·9)**	-	1 (0·2)	18 (1·5)	**19 (1·1)**	**17**
10	Thailand	23 (27·1)	49 (22·1)	**72 (23·5)**	**1·1**	58 (37·7)	88 (24·3)	**146 (28·3)**	**0·5**	25 (25·8)	86 (22·8)	**111 (23·6)**	**2·4**	25 (31·3)	47 (20·5)	**72 (23·3)**	**0·9**	18 (30·0)	40 (24·0)	**58 (25·6)**	**1·2**	129 (31·5)	278 (22·6)	**407 (24·8)**	**1·1**
11	Timor-Leste	-	-	-	-	-	1 (0·3)	**1 (0·2)**	-	-	1 (0·3)	**1 (0·2)**	-	-	-	-	-	-	1 (0·6)	**1 (0·4)**	-	-	3 (0·2)	**3 (0·2)**	-
12	SEAR	22 (25·9)	9 (4·1)	**31 (10·1)**	**-0·6**	28 (18·2)	13 (3·6)	**41 (7·9)**	**-0·5**	20 (20·6)	8 (2·1)	**28 (6·0)**	**-0·6**	13 (16·3)	4 (1·7)	**17 (5·5)**	**-0·7**	8 (13·3)	5 (3·0)	**13 (5·7)**	**-0·4**	80 (19·5)	36 (2·9)	**116 (7·1)**	**-0·5**
13	Asia *	1 (1·2)	4 (1·8)	**5 (1·6)**	**3**	5 (3·2)	1 (0·3)	**6 (1·2)**	**-0·8**	-	6 (1·6)	**6 (1·3)**	-	1 (1·3)	3 (1·3)	**4 (1·3)**	**2·0**	2 (3·3)	3 (1·8)	**5 (2·2)**	**0·5**	4 (1·7)	16 (1·3)	**23 (1·4)**	**1·3**
14	Global #	11 (12·9)	12 (5·4)	**23 (7·5)**	**0·1**	8 (5·2)	19 (5·2)	**27 (5·2)**	**1·3**	10 (10·3)	37 (9·8)	**47 (10·0)**	**2·7**	7 (8·8)	8 (3·5)	**15 (4·9)**	**0·1**	5 (8·3)	5 (3·0)	**10 (4·4)**	**0**	37 (8·3)	75 (6·1)	**109 (6·6)**	**1·2**

Table S1-3. Distribution of articles included in the review according to type of research

S.no	Type of research	Decade		Total n^{Ψ}(%)	Decadal Growth Rate
		2000-2010 n*(%)	2011-2021 n^{ϕ}(%)		
1	Basic Science	129 (31·5)	459 (37·3)	588 (35·7)	2·5
2	Clinical	112 (27·3)	190 (15·4)	302 (18·4)	0·7
3	Socio-behavioral and Public Health	172 (42·0)	597 (48·5)	769 (46·9)	2·5

n*=410, n^{ϕ}=1231, n^{Ψ} =1641

Table S1-4. Distribution of articles included in the review according to study design

S.no	Study Design	Decade		Total nΨ(%)	Decadal Growth Rate
		2000-2010 n*(%)	2011-2021 nϕ(%)		
1	Bench Research	129 (31·5)	459 (37·3)	588 (35·8)	2·5
2	Observational	254 (61·9)	713 (57·9)	967 (58·9)	1·8
3	Interventional	27 (6·6)	66 (5·4)	93 (5·7)	1·4
4	Review	78 (19·0)	109 (8·8)	187 (11·4)	0·4

n*=410, nϕ =1231, nΨ=1641

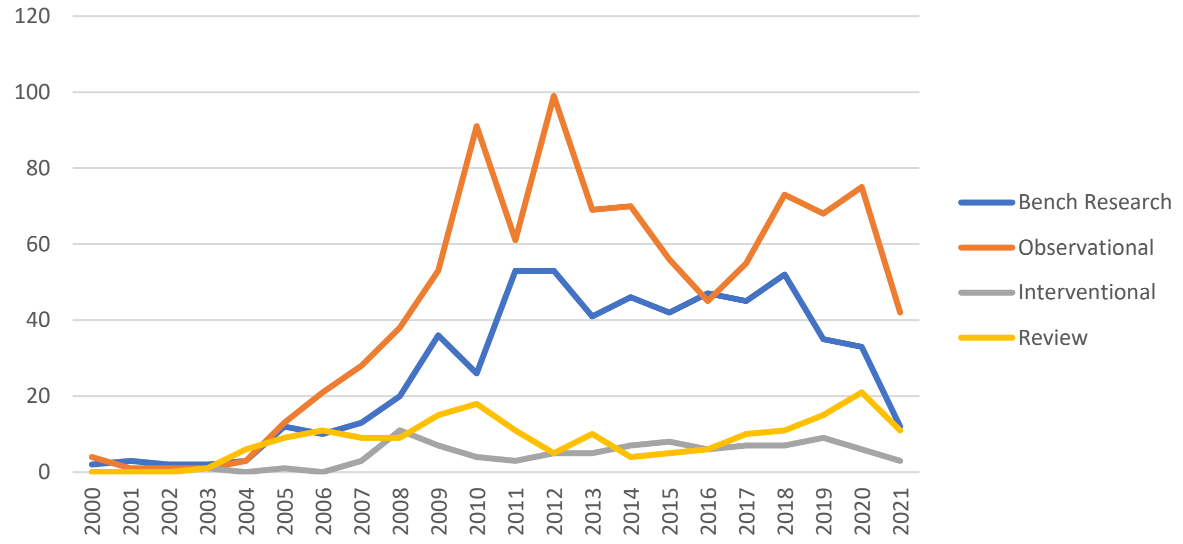

**data for 2021 (ongoing year) is as of August 2021*

Figure S1-1: Trend of number of publications included in the review based on study design

Supplementary File #2

Bangladesh

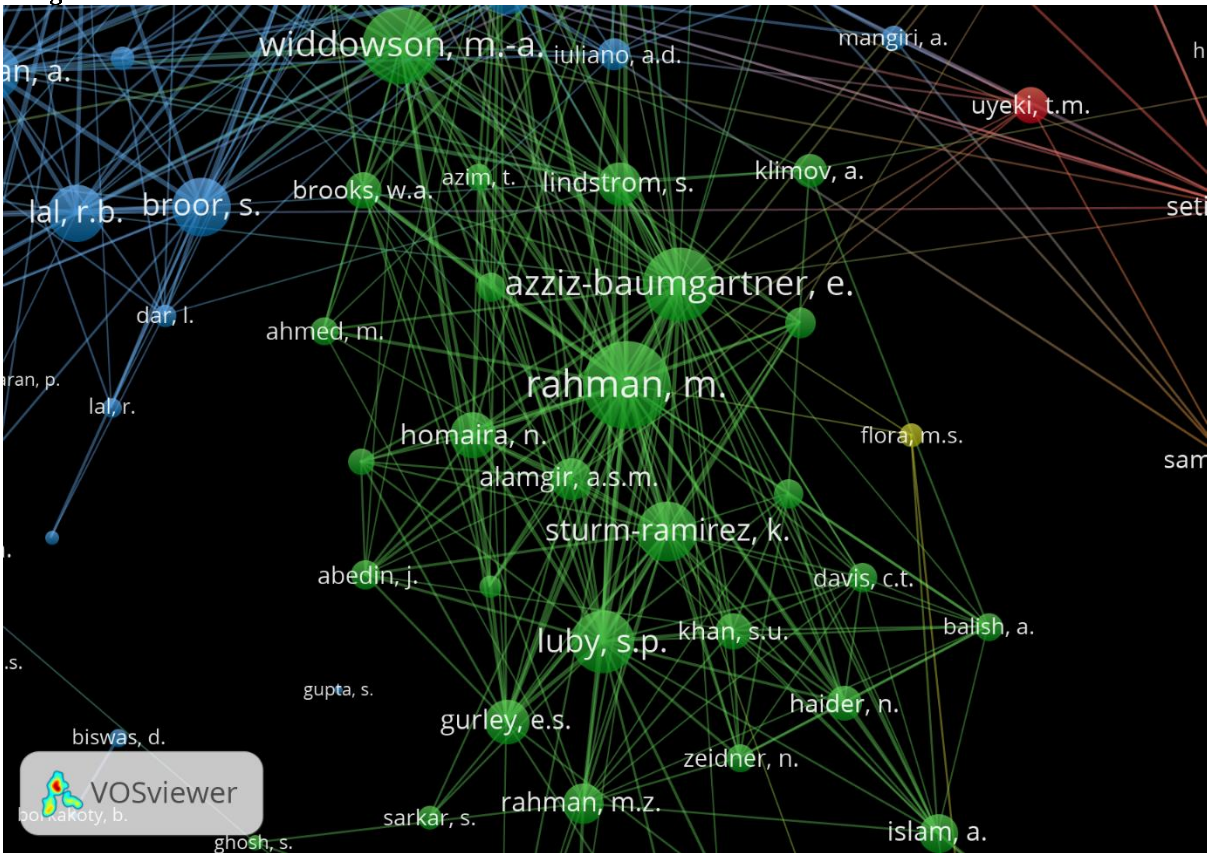

Figure S2-1: Network visualization of connectivity of authors (Bangladesh)

Table S2-1. List of top authors contributing to Influenza research in Bangladesh

S.no.	Author	H-Index#	Total link strength*	Affiliated Institution	No. of influenza related publications
1	Rahman, M.	48	249	International Centre for Diarrheal Disease Research	40
2	Azziz-Baumgartner, E.	34	190	International Centre for Diarrheal Disease Research	27
3	Sturm-Ramirez, K.	23	140	International Centre for Diarrheal Disease Research	17
4	Feeroz, M.M.	16	114	Jahangirnagar University	16
5	Alamgir, A.S.M.	15	78	Institute of Epidemiology, Disease Control and Research (IEDCR)	9
6	Rahman, M.Z.	6	75	Jahangirnagar University	14
7	Brooks W. Abdullah	39	65	International Centre for Diarrhoeal Disease Research Bangladesh	17
8	Hasan, M.K.	19	63	Jahangirnagar University	7

9	Khan, S.U.	11	63	International Centre for Diarrhoeal Disease Research Bangladesh	6
10	Giasuddin, M.	13	59	Bangladesh Livestock Research Institute	19

#Source: Scopus
*Total link strength: Links attribute indicates the number of co-authorship links of a given researcher with other researchers. Total link strength indicates the total strength of the co-authorship links of a given researcher with other researchers.

3. India

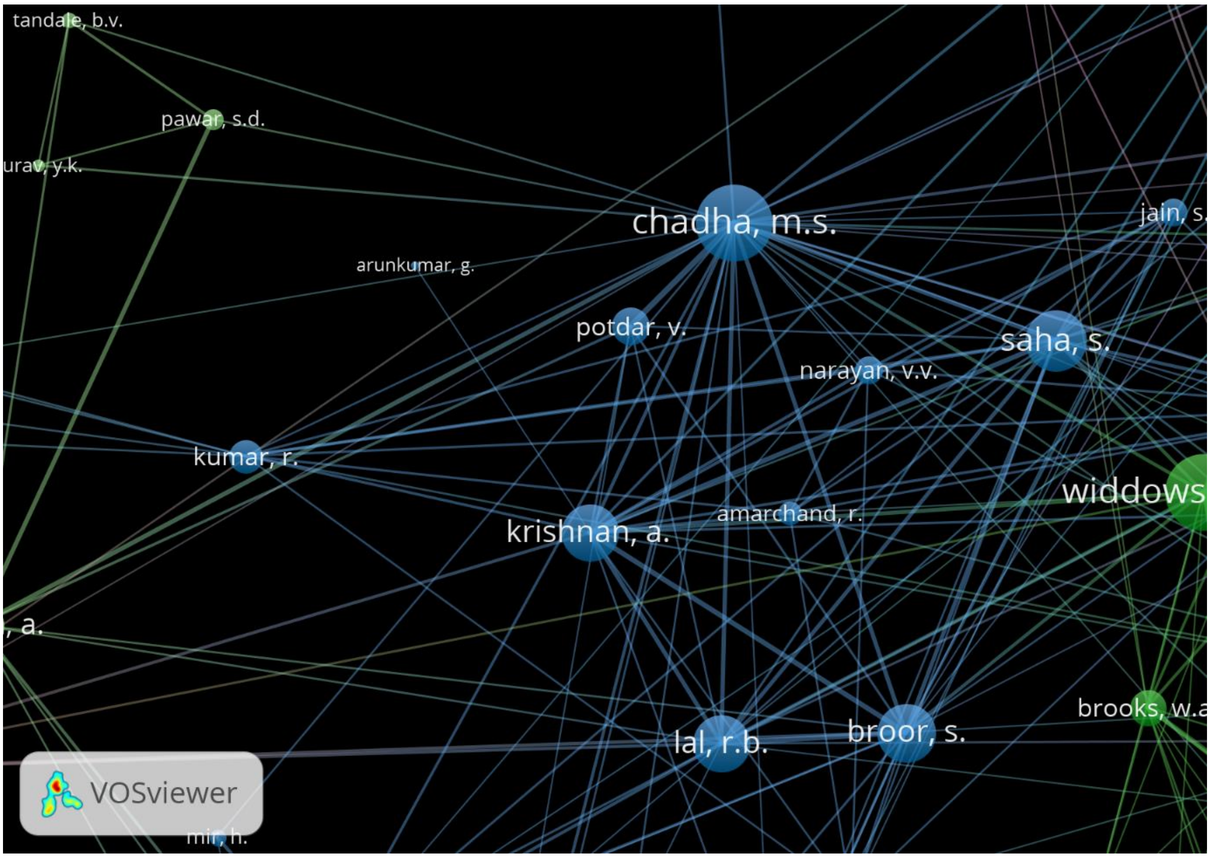

Figure S2-2: Network visualization of connectivity of authors (India)

Table S2-2. List of top authors contributing to Influenza research in India

S.no.	Author	H-Index#	Total link strength*	Affiliated Institution	No. of influenza related publications
1	Chadha, M.S.	37	201	National Institute of Virology, Pune	50
2	Broor, S.	36	133	Shree Guru Gobind Singh Tricentenary University	30
3	Krishnan, A.	34	129	All India Institute of Medical Sciences, New Delhi	24
4	Mishra, A.	42	105	Bharati Vidyapeeth, Pune	32
5	Nagarajan, S.	15	94	ICAR - National Institute of High Security Animal Diseases, Bhopal	19
6	Tosh, C.	22	92	ICAR - National Institute of High Security Animal Diseases, Bhopal	16

7	Murugkar, H.V.	13	78	ICAR - National Institute of High Security Animal Diseases, Bhopal	14
8	Kulkarni, D.D.	16	73	ICAR - National Institute of High Security Animal Diseases, Bhopal	14
9	Potdar, V.	19	69	National Institute of Virology, Pune	21
10	Dubey, S.C.		63	ICAR - National Institute of High Security Animal Diseases, Bhopal	10
#Source: Scopus<br>*Total link strength: Links attribute indicates the number of co-authorship links of a given researcher with other researchers. Total link strength indicates the total strength of the co-authorship links of a given researcher with other researchers.					

4. Indonesia

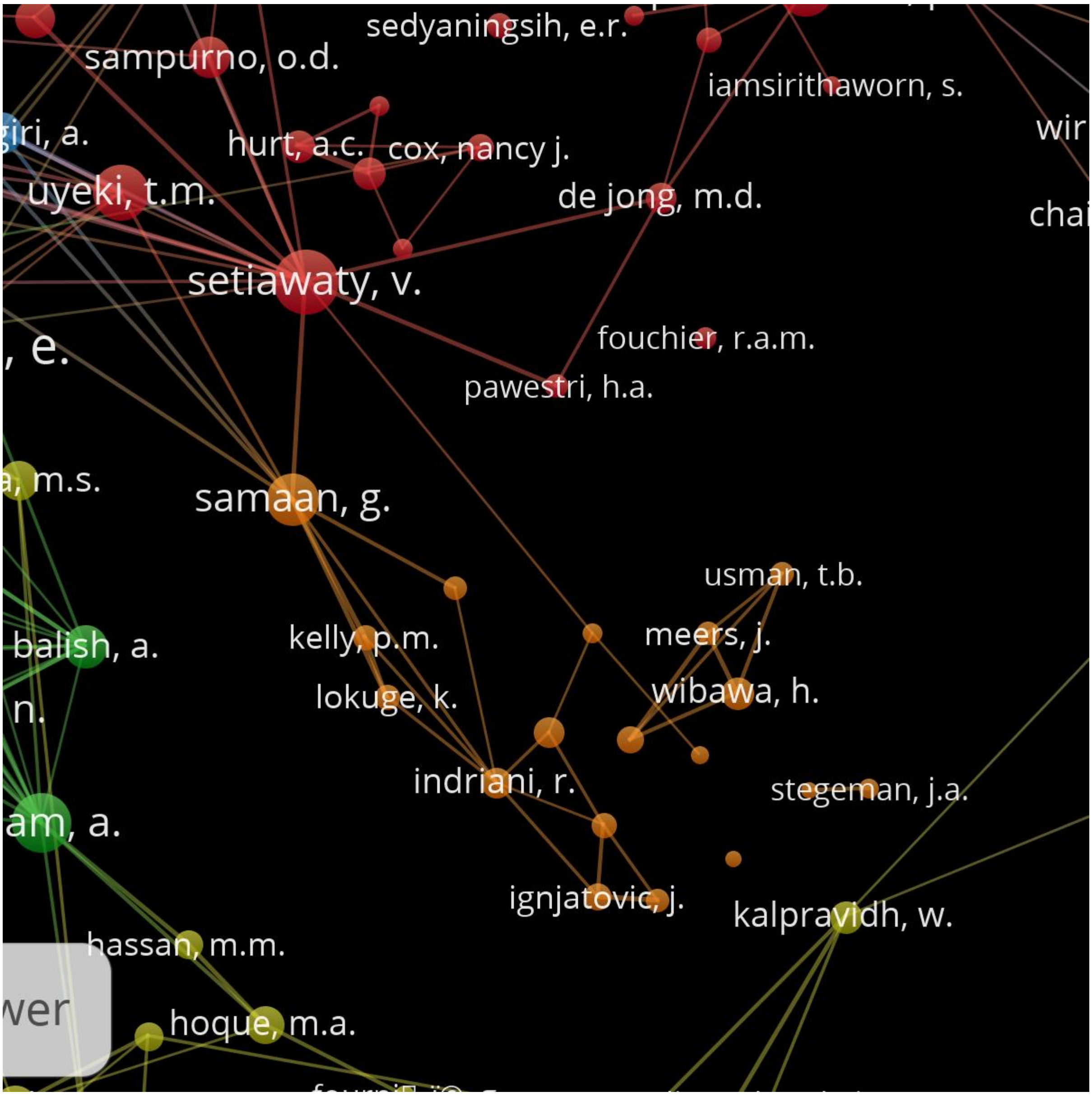

Figure S2-3: Network visualization of connectivity of authors (Indonesia)

Table S2-3. List of top authors contributing to Influenza research in Indonesia

S.no.	Author	H-Index#	Total link strength*	Affiliated Institution	No. of influenza related publications
1	Setiawaty, Vivi	10	85	Nepal Nutrition Intervention Project-Sarlahi	21
2	Sampurno, O.D.	6	37	Health Research and Development Agency, Ministry of Health of the Republic of Indonesia	7
3	Pangesti, K.N.A.	6	36	Health Research and Development Agency, Ministry of Health of the Republic of Indonesia	5
4	Wibawa, H.	9	25	Yogyakarta Animal Disease Investigation Centre	14
5	Indriani, R.	7	23	Ministry of Agriculture, Jakarta	8
6	Sedyaningsih, E.R.	18	15	Health Research and Development Agency, Ministry of Health of the Republic of Indonesia	6
7	Dharmayanti, N.L.P.I.	6	14	Ministry of Agriculture, Jakarta	6
8	Tarigan, S.	5	14	Indonesian Research Centre for Veterinary Science	6
9	Pawestri, H.A.	3	13	Health Research and Development Agency, Ministry of Health of the Republic of Indonesia	5
10	Usman, T.B.	6	13	Disease Investigation Centre Regional IV Wates	5

#Source: Scopus
*Total link strength: Links attribute indicates the number of co-authorship links of a given researcher with other researchers. Total link strength indicates the total strength of the co-authorship links of a given researcher with other researchers.

5. Nepal

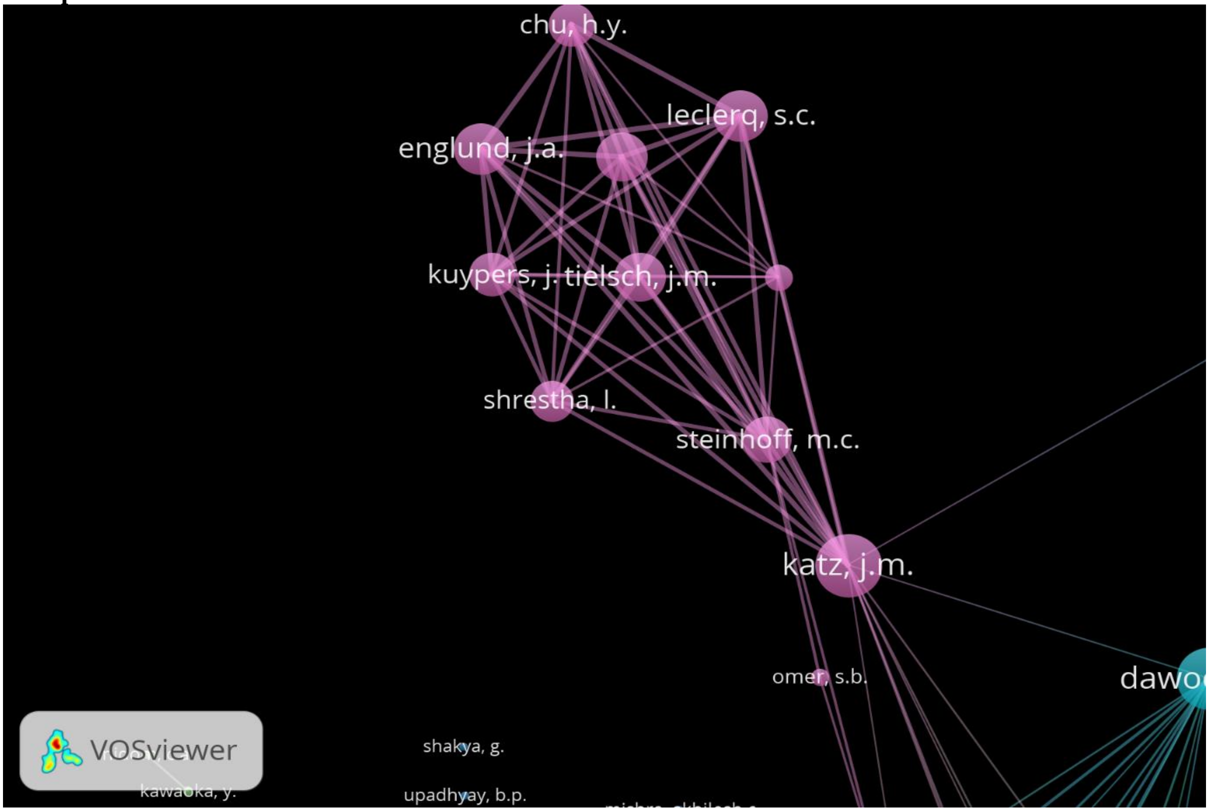

Figure S2-4: Network visualization of connectivity of authors (Nepal)

Table S2-4. List of top authors contributing to Influenza research in Nepal

S.no.	Author	H-Index#	Total link strength*	Affiliated Institution	No. of influenza related publications
1	Leclerq, s.c.	41	120	Nepal Nutrition Intervention Project-Sarlahi	19
2	Khatry, s.k.	46	110	Nepal Nutrition Intervention Project-Sarlahi	17
3	Shrestha, l.	13	86	Tribhuvan University	12
4	shakya, g.	11	5	Minister for Health and Population Kathmandu	5
5	upadhyay, b.p.	7	4	National Public Health Laboratory Kathmandu	7

#Source: Scopus
*Total link strength: Links attribute indicates the number of co-authorship links of a given researcher with other researchers. Total link strength indicates the total strength of the co-authorship links of a given researcher with other researchers.

6. Thailand

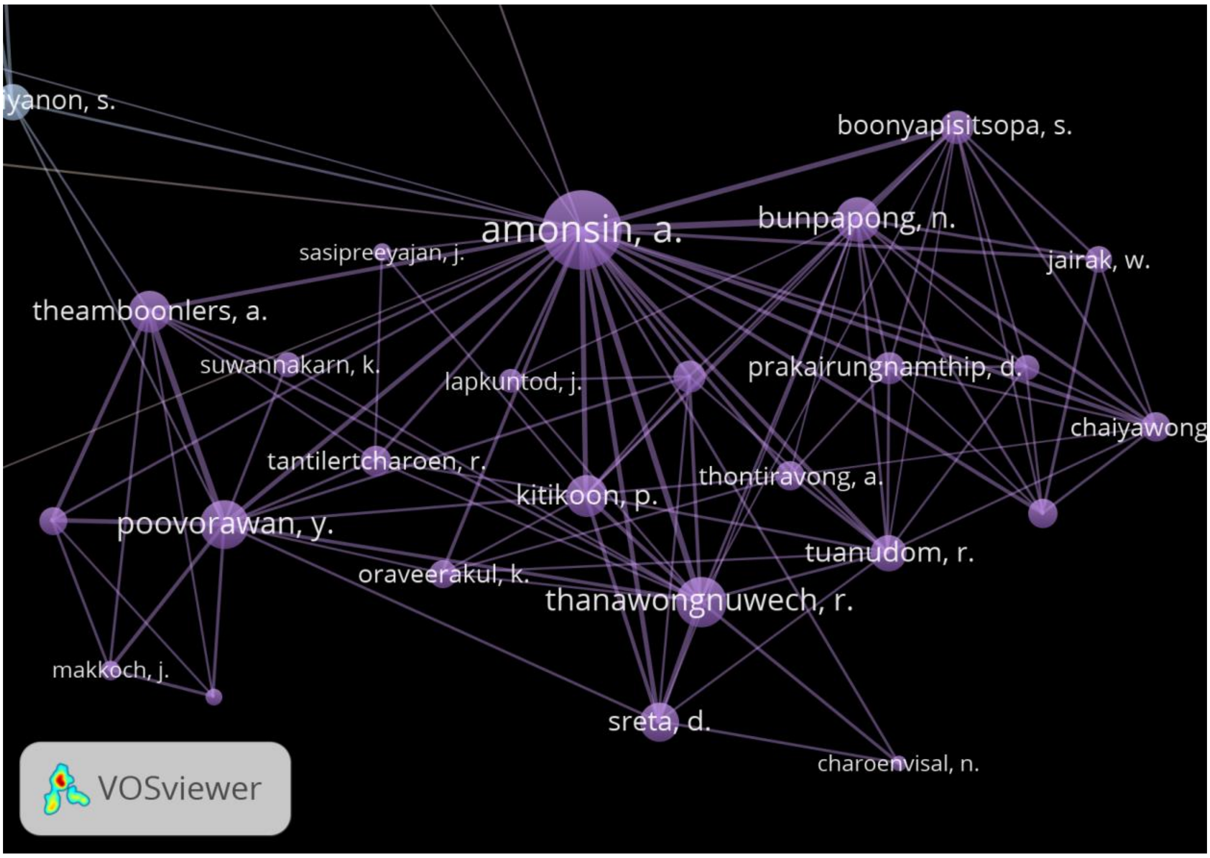

Figure S2-5: Network visualization of connectivity of authors (Thailand)

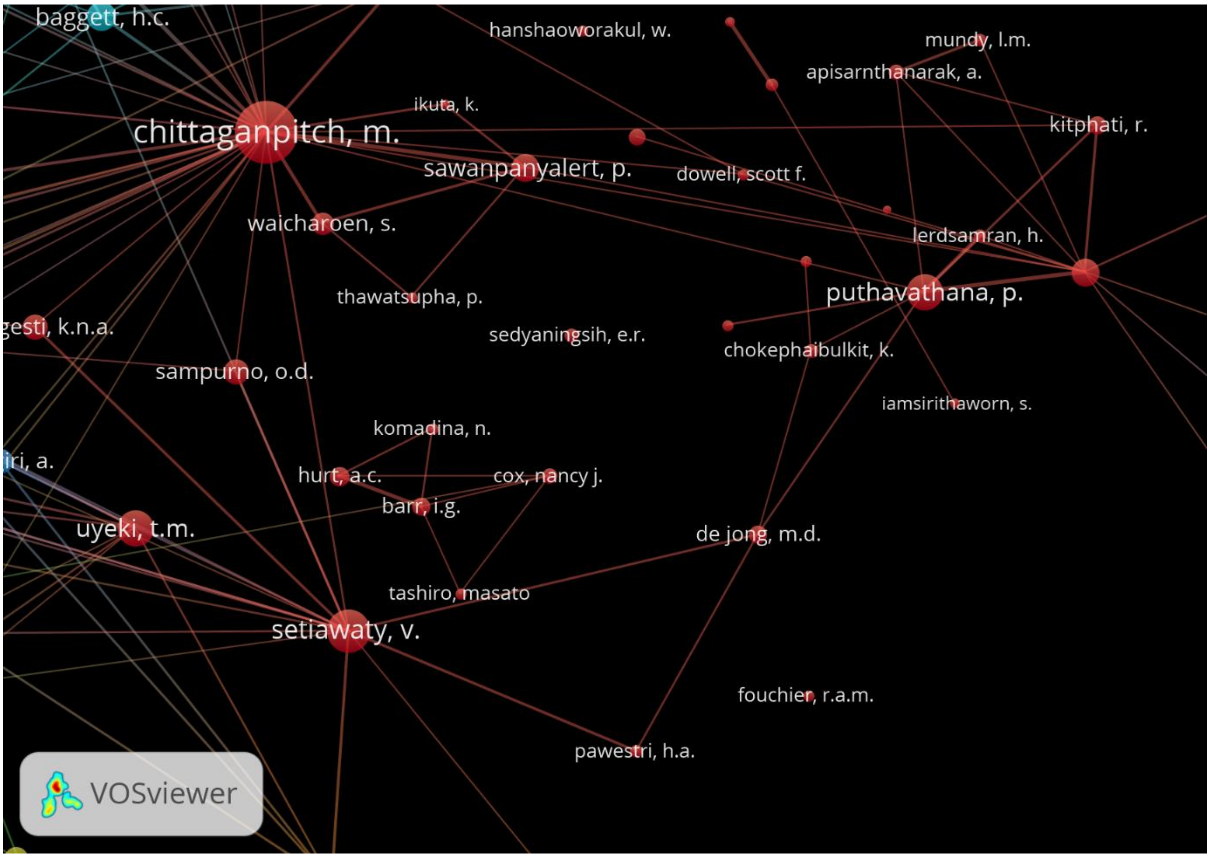

Figure S2-6: Network visualization of connectivity of authors (Thailand)

Table S2-5. List of top authors contributing to Influenza research in Thailand

S.no.	Author	H-Index#	Total link strength*	Affiliated Institution	No. of influenza related publications
1	Amonsin, A.	31	182	Faculty of Veterinary Science, Chulalongkorn University	44
2	Chittaganpitch, M.	29	149	National Institute of Health	42
3	Suntarattiwon, P.	13	88	Queen Sirikit National Institute of Child Health	17
4	Thanawongnuwech, R.	31	88	Chulalongkorn University	16
5	Poovorawan, Y.	52	87	Center of Excellence in Clinical Virology, Faculty of Medicine, Chulalongkorn University	22
6	Chotpitayasunondh, T.	31	85	Queen Sirikit National Institute of Child Health	18
7	Stefan Fernandez	24	82	Armed Forces Research Institute of Medical Sciences, Thailand	18
8	Bunpapong, N.	10	73	Chulalongkorn University	16
9	Klungthong, C.	23	73	Armed Forces Research Institute of Medical Sciences	19
10	Kittikraisak,W.	11	70	Centers for Disease Control (CDC), Thailand Field Station	11

#Source: Scopus
*Total link strength: Links attribute indicates the number of co-authorship links of a given researcher with other researchers. Total link strength indicates the total strength of the co-authorship links of a given researcher with other researchers.

Supplementary File #3

Publication Trends

1. Bangladesh

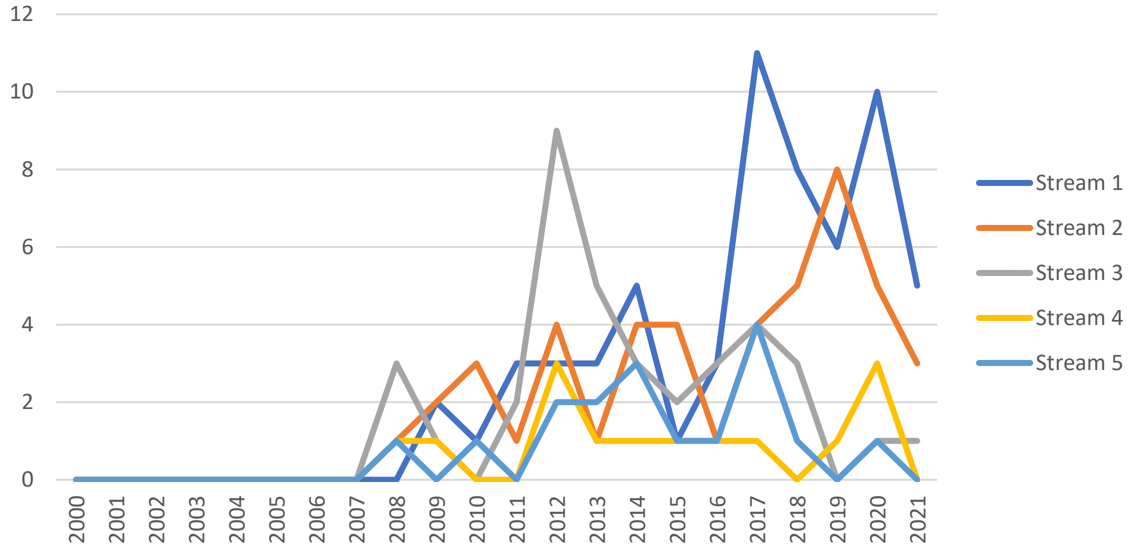

Figure S3-1: Trend of number of publications of Bangladesh based on WHO influenza streams
**data for 2021 (ongoing year) is as of August 2021*
Streams: 1-Reducing the risk of emergence of a pandemic influenza; 2-Limiting the spread of pandemic, zoonotic, and seasonal epidemic influenza; 3-Minimizing the impact of pandemic, zoonotic and seasonal epidemic influenza; 4-Optimizing the treatment of patients; 5-Promoting the development and application of new public health tools

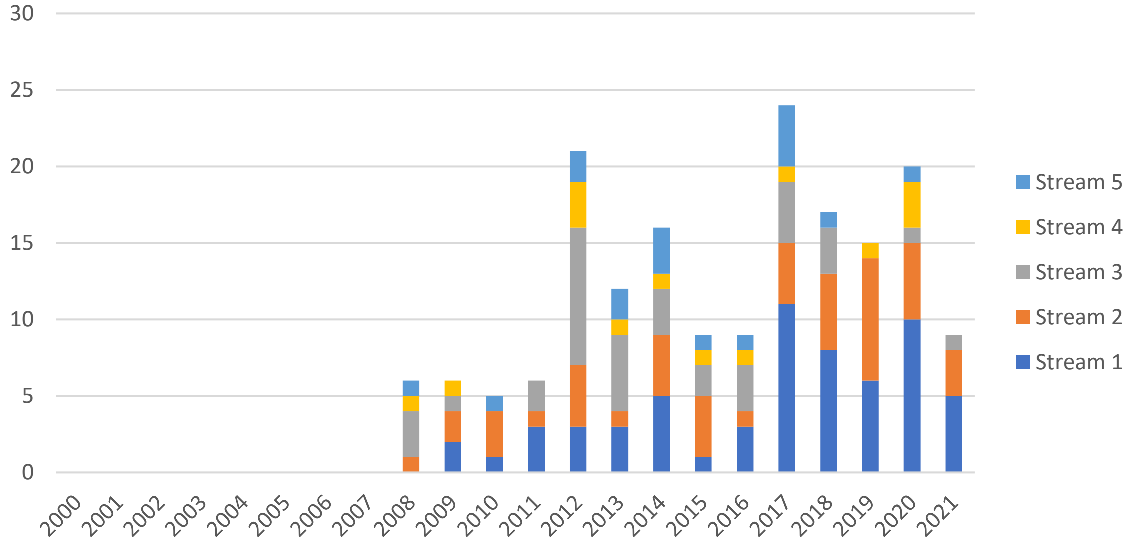

Figure S3-2: Stacked plot of number of publications of Bangladesh based on WHO influenza streams
**data for 2021 (ongoing year) is as of August 2021*
Streams: 1-Reducing the risk of emergence of a pandemic influenza; 2-Limiting the spread of pandemic, zoonotic, and seasonal epidemic influenza; 3-Minimizing the impact of pandemic, zoonotic and seasonal epidemic influenza; 4-Optimizing the treatment of patients; 5-Promoting the development and application of new public health tools

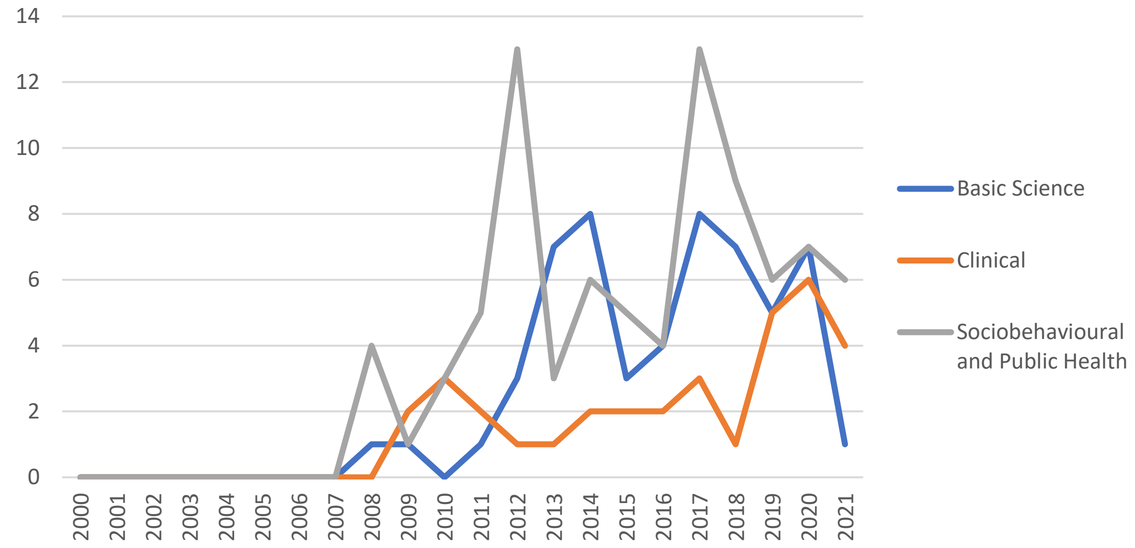

**data for 2021 (ongoing year) is as of August 2021*

Figure S3-3: Trend of number of publications of Bangladesh based on type of research

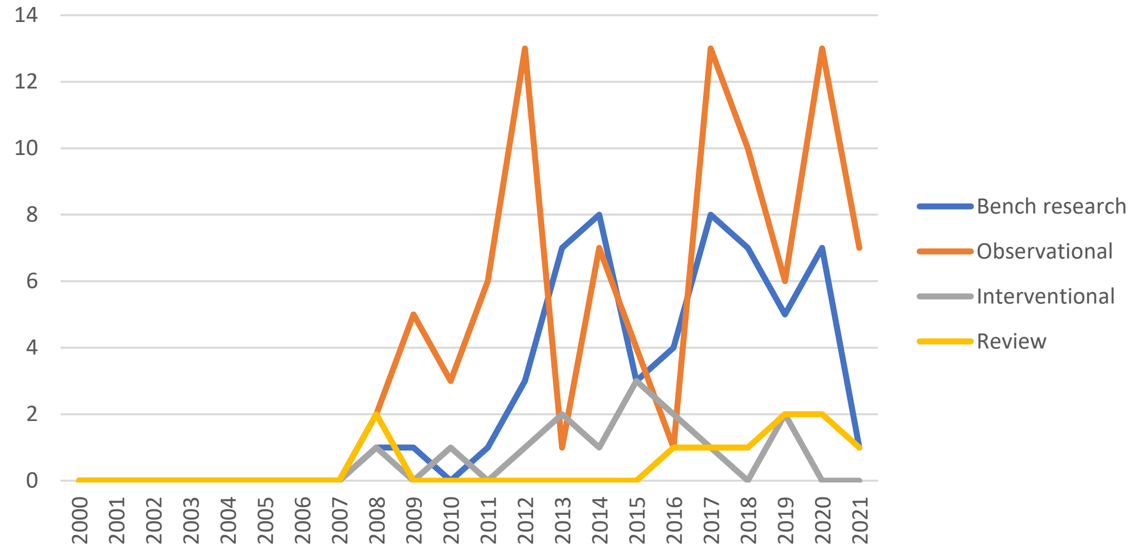

**data for 2021 (ongoing year) is as of August 2021*

Figure S3-4: Trend of number of publications of Bangladesh based on study design

2. Bhutan

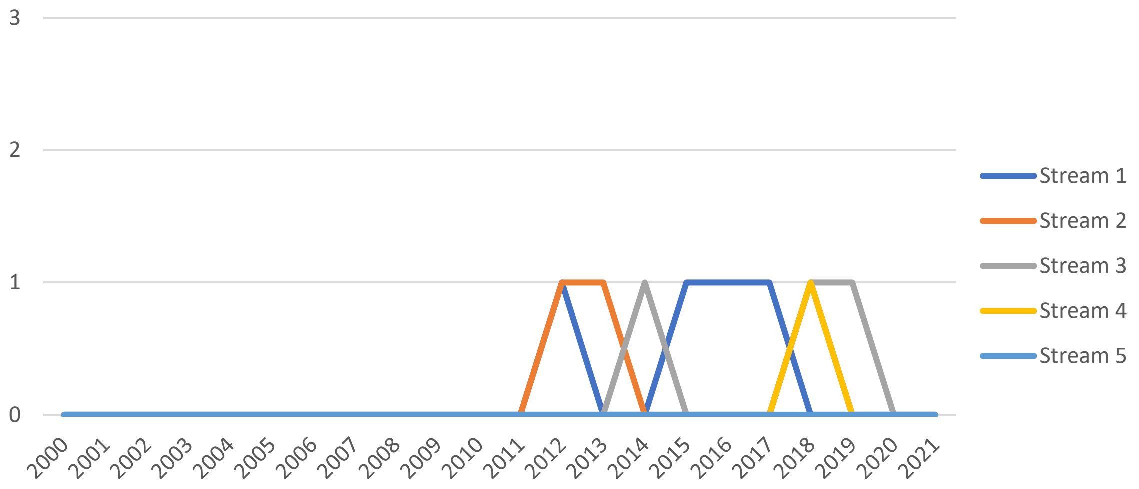

Figure S3-5: Trend of number of publications of Bhutan based on WHO influenza streams
**data for 2021 (ongoing year) is as of August 2021*
Streams: 1-Reducing the risk of emergence of a pandemic influenza; 2-Limiting the spread of pandemic, zoonotic, and seasonal epidemic influenza; 3-Minimizing the impact of pandemic, zoonotic and seasonal epidemic influenza; 4-Optimizing the treatment of patients; 5-Promoting the development and application of new public health tools

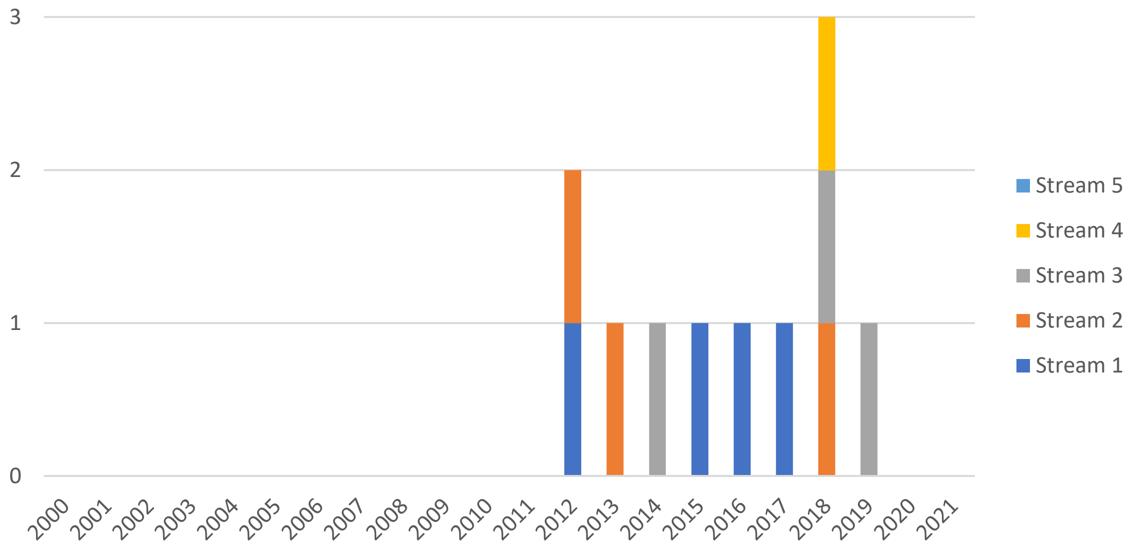

Figure S3-6: Stacked plot of number of publications of Bhutan based on WHO influenza streams
**data for 2021 (ongoing year) is as of August 2021*
Streams: 1-Reducing the risk of emergence of a pandemic influenza; 2-Limiting the spread of pandemic, zoonotic, and seasonal epidemic influenza; 3-Minimizing the impact of pandemic, zoonotic and seasonal epidemic influenza; 4-Optimizing the treatment of patients; 5-Promoting the development and application of new public health tools

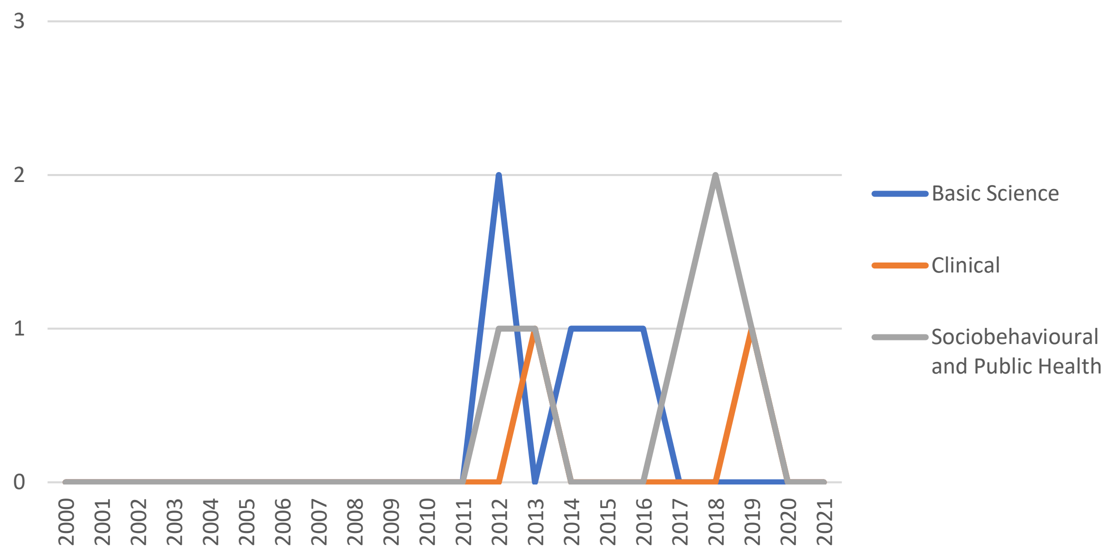

**data for 2021 (ongoing year) is as of August 2021*

Figure S3-7: Trend of number of publications of Bhutan based on type of research

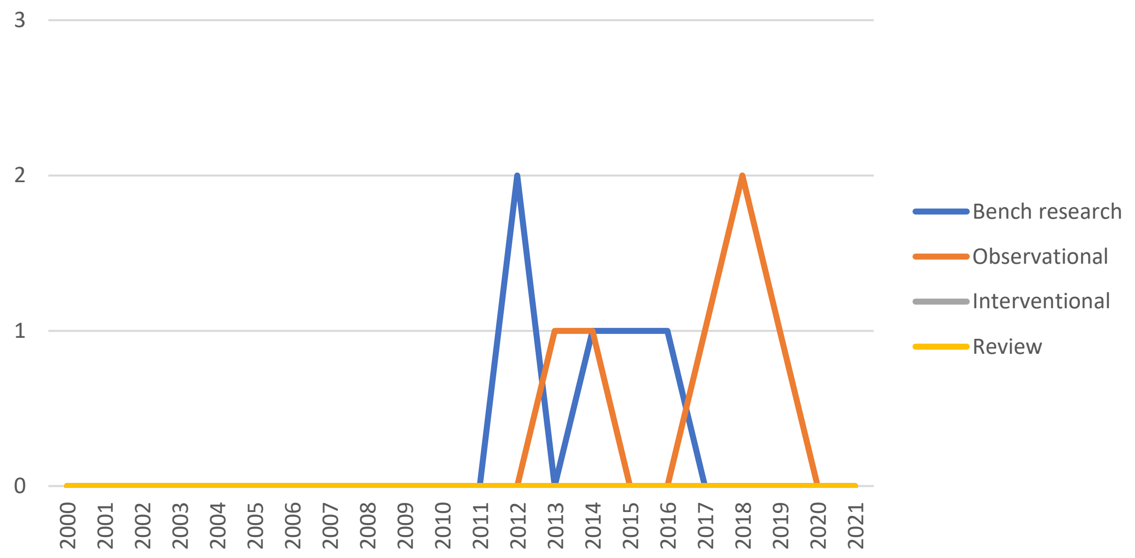

**data for 2021 (ongoing year) is as of August 2021*

Figure S3-8: Trend of number of publications of Bhutan based on study design

3. DPR Korea

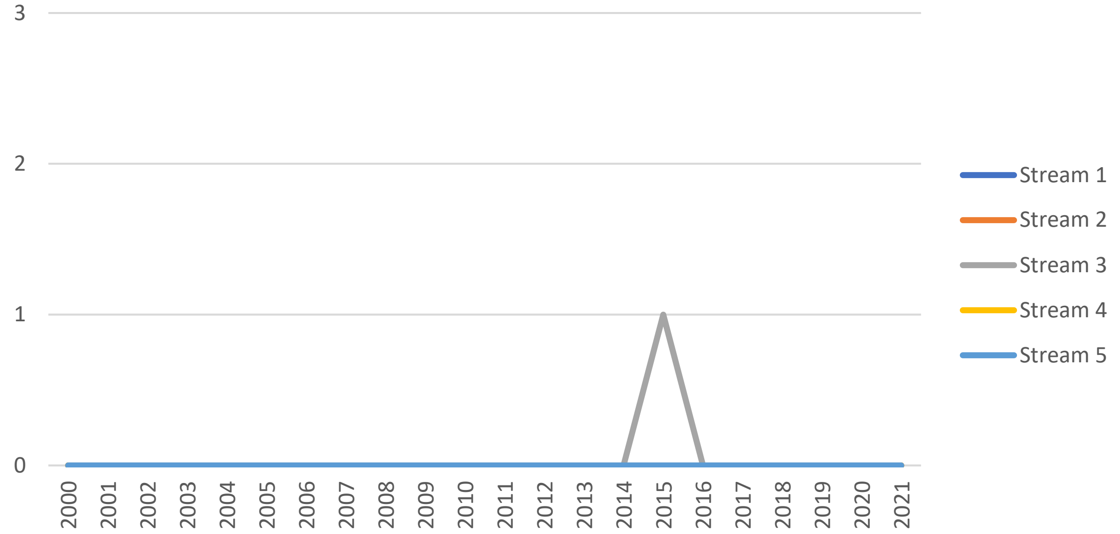

Figure S3-9: Trend of number of publications of DPR Korea based on WHO influenza streams

***data for 2021 (ongoing year) is as of August 2021*

Streams: 1-Reducing the risk of emergence of a pandemic influenza; 2-Limiting the spread of pandemic, zoonotic, and seasonal epidemic influenza; 3-Minimizing the impact of pandemic, zoonotic and seasonal epidemic influenza; 4-Optimizing the treatment of patients; 5-Promoting the development and application of new public health tools

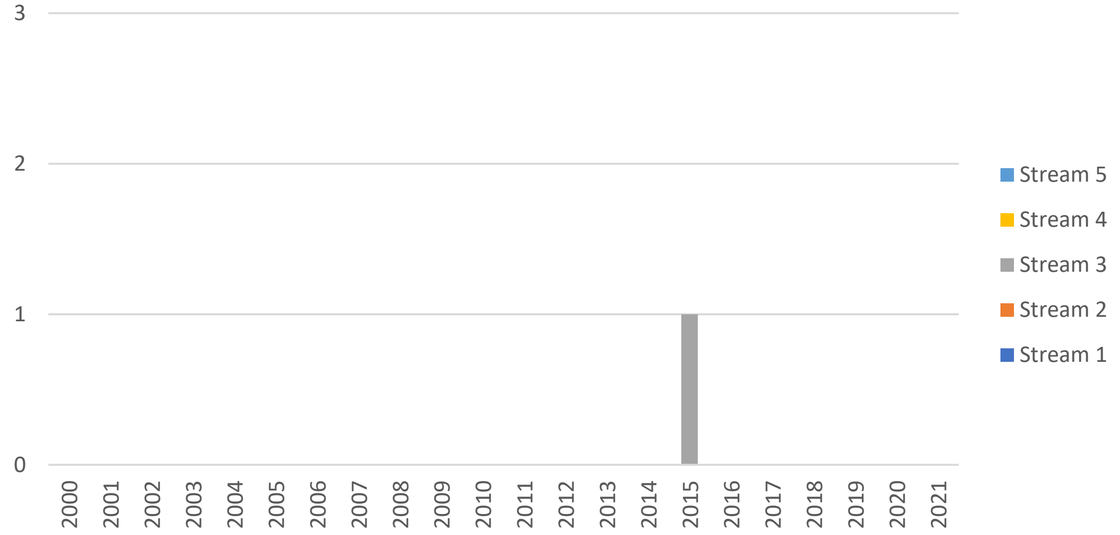

Figure S3-10: Stacked plot of number of publications of DPR Korea based on WHO influenza streams

***data for 2021 (ongoing year) is as of August 2021*

Streams: 1-Reducing the risk of emergence of a pandemic influenza; 2-Limiting the spread of pandemic, zoonotic, and seasonal epidemic influenza; 3-Minimizing the impact of pandemic, zoonotic and seasonal epidemic influenza; 4-Optimizing the treatment of patients; 5-Promoting the development and application of new public health tools

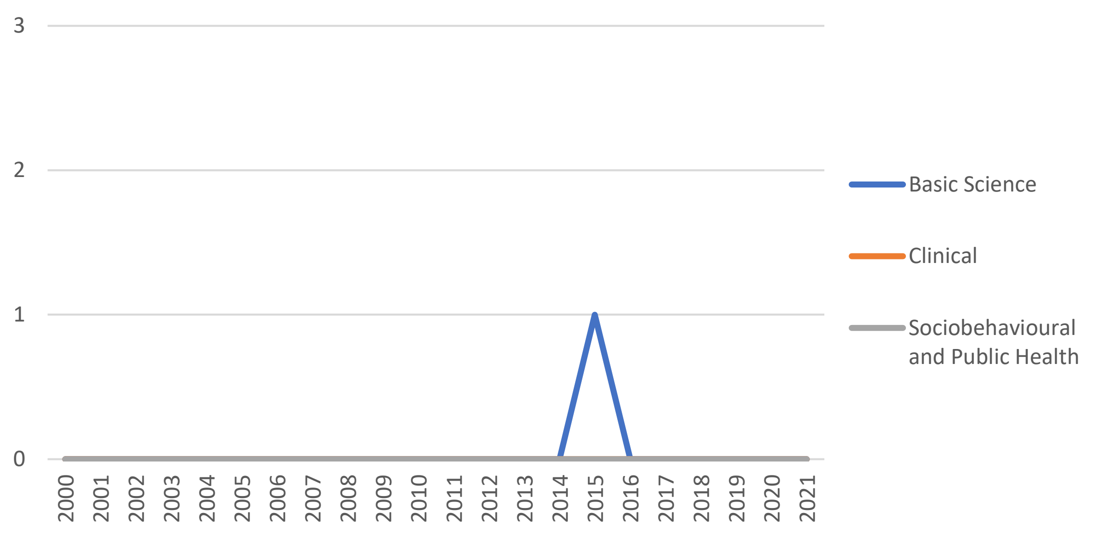

**data for 2021 (ongoing year) is as of August 2021*

Figure S3-11: Trend of number of publications of DPR Korea based on type of research

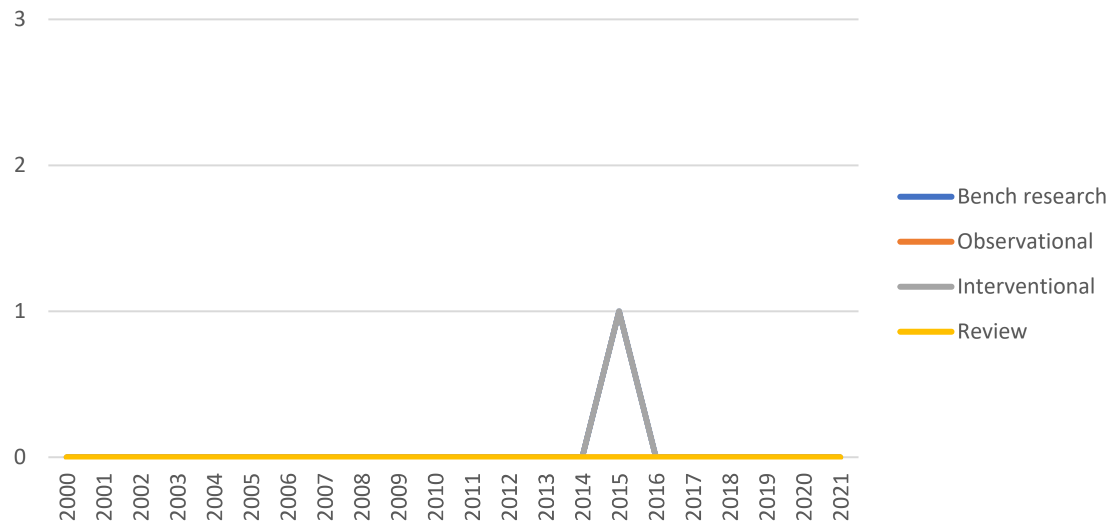

**data for 2021 (ongoing year) is as of August 2021*

Figure S3-12: Trend of number of publications of DPR Korea based on study design

4. India

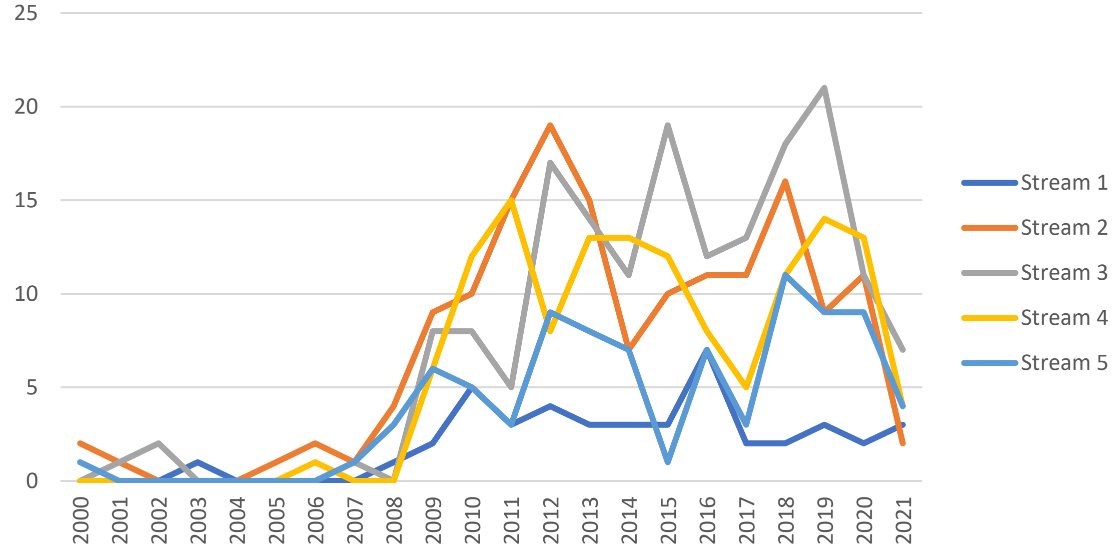

Figure S3-13: Trend of number of publications of India based on WHO influenza streams
**data for 2021 (ongoing year) is as of August 2021*
Streams: 1-Reducing the risk of emergence of a pandemic influenza; 2-Limiting the spread of pandemic, zoonotic, and seasonal epidemic influenza; 3-Minimizing the impact of pandemic, zoonotic and seasonal epidemic influenza; 4-Optimizing the treatment of patients; 5-Promoting the development and application of new public health tools

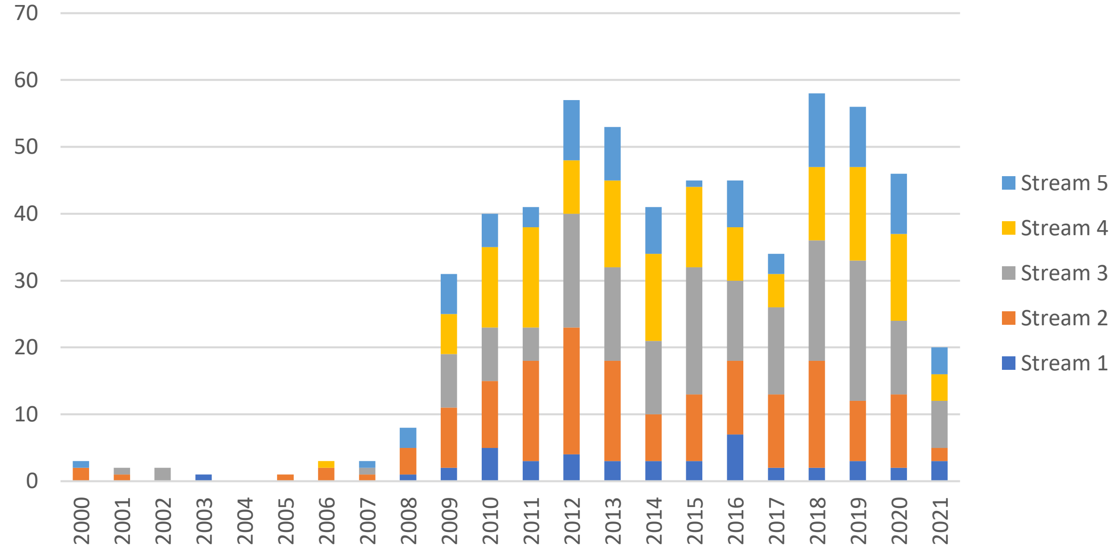

Figure S3-14: Stacked plot of number of publications of India based on WHO influenza streams

**data for 2021 (ongoing year) is as of August 2021*
Streams: 1-Reducing the risk of emergence of a pandemic influenza; 2-Limiting the spread of pandemic, zoonotic, and seasonal epidemic influenza; 3-Minimizing the impact of pandemic, zoonotic and seasonal epidemic influenza; 4-Optimizing the treatment of patients; 5-Promoting the development and application of new public health tools

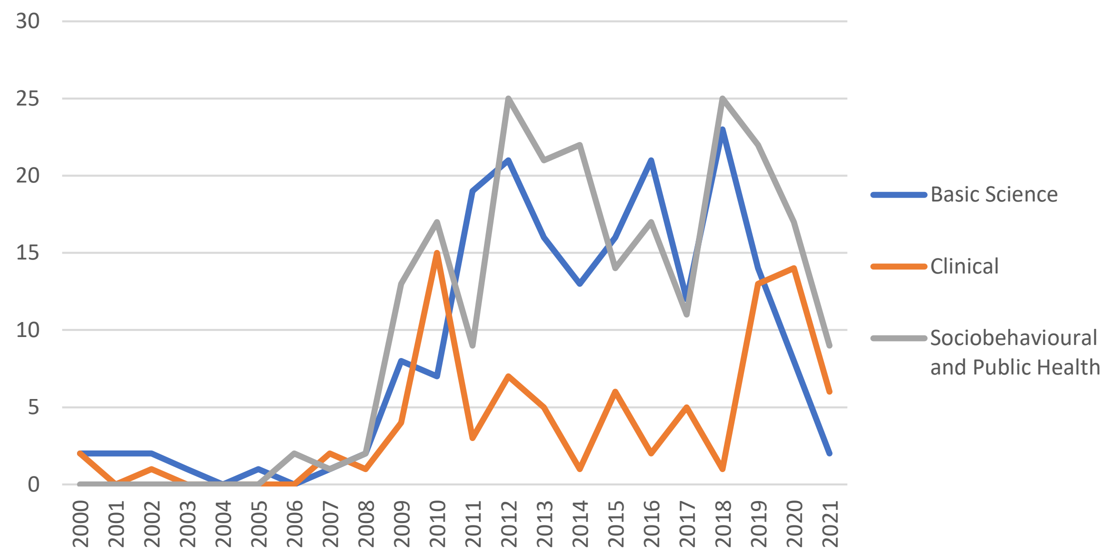

data for 2021 (ongoing year) is as of August 2021

Figure S3-15: Trend of number of publications of India based on type of research

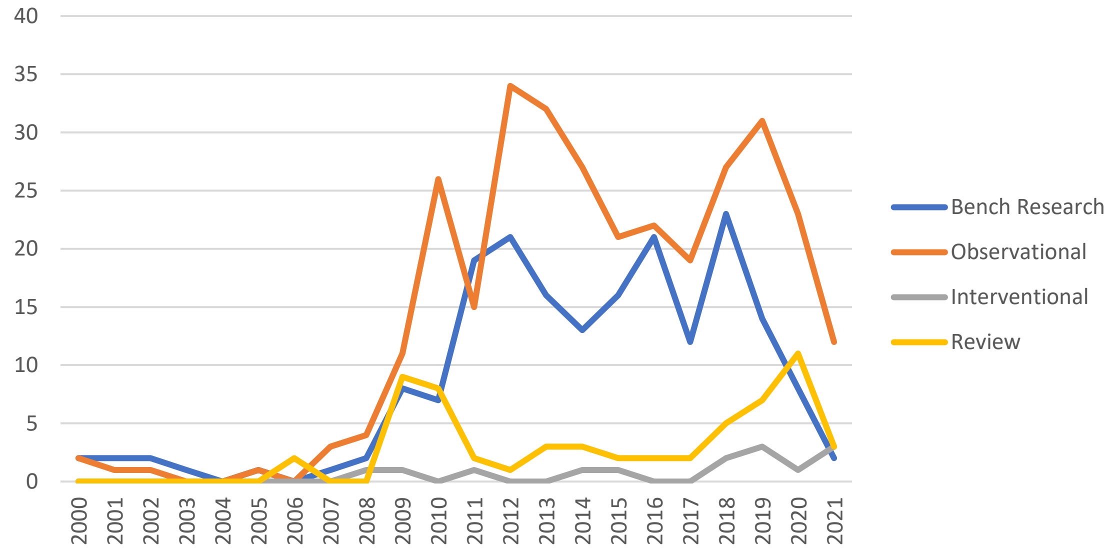

data for 2021 (ongoing year) is as of August 2021

Figure S3-16: Trend of number of publications of India based on study design

5. Indonesia

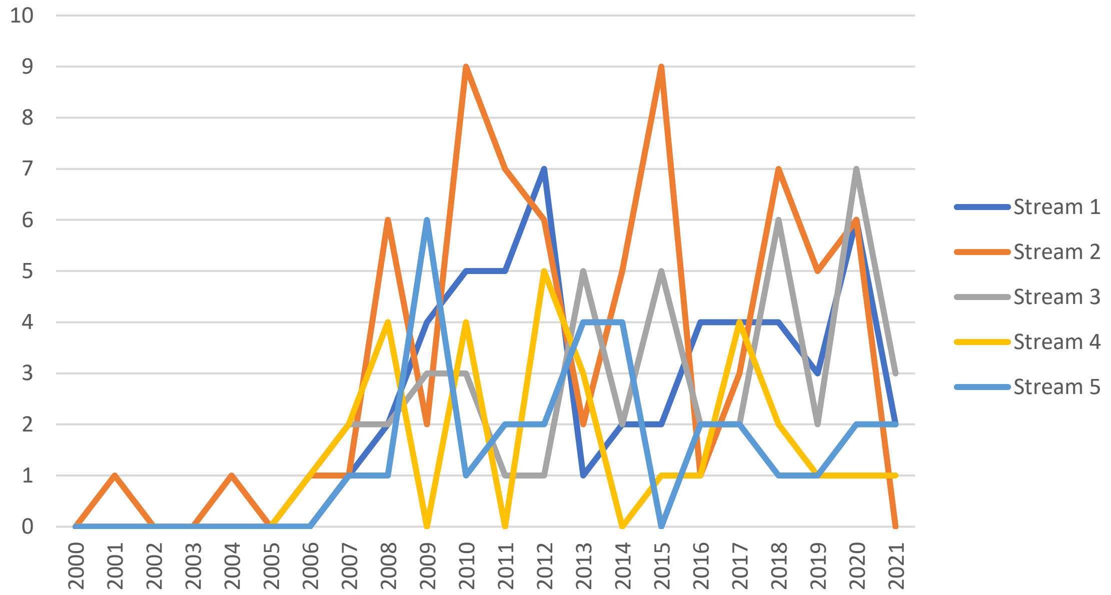

Figure S3-17: Trend of number of publications of Indonesia based on WHO influenza streams
**data for 2021 (ongoing year) is as of August 2021*
Streams: 1-Reducing the risk of emergence of a pandemic influenza; 2-Limiting the spread of pandemic, zoonotic, and seasonal epidemic influenza; 3-Minimizing the impact of pandemic, zoonotic and seasonal epidemic influenza; 4-Optimizing the treatment of patients; 5-Promoting the development and application of new public health tools

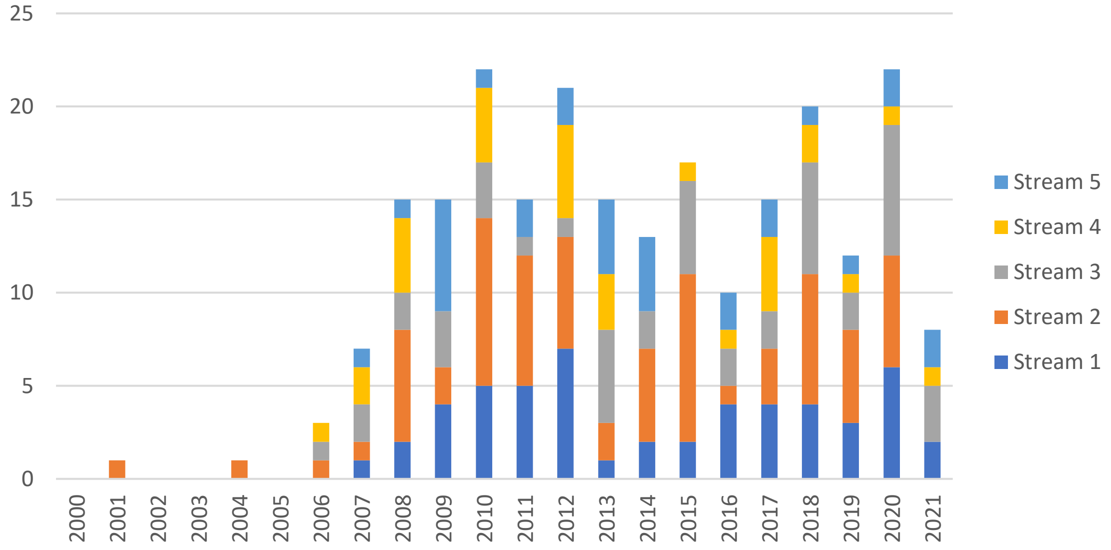

Figure S3-18: Stacked plot of number of publications of Indonesia based on WHO influenza streams
**data for 2021 (ongoing year) is as of August 2021*
Streams: 1-Reducing the risk of emergence of a pandemic influenza; 2-Limiting the spread of pandemic, zoonotic, and seasonal epidemic influenza; 3-Minimizing the impact of pandemic, zoonotic and seasonal epidemic influenza; 4-Optimizing the treatment of patients; 5-Promoting the development and application of new public health tools

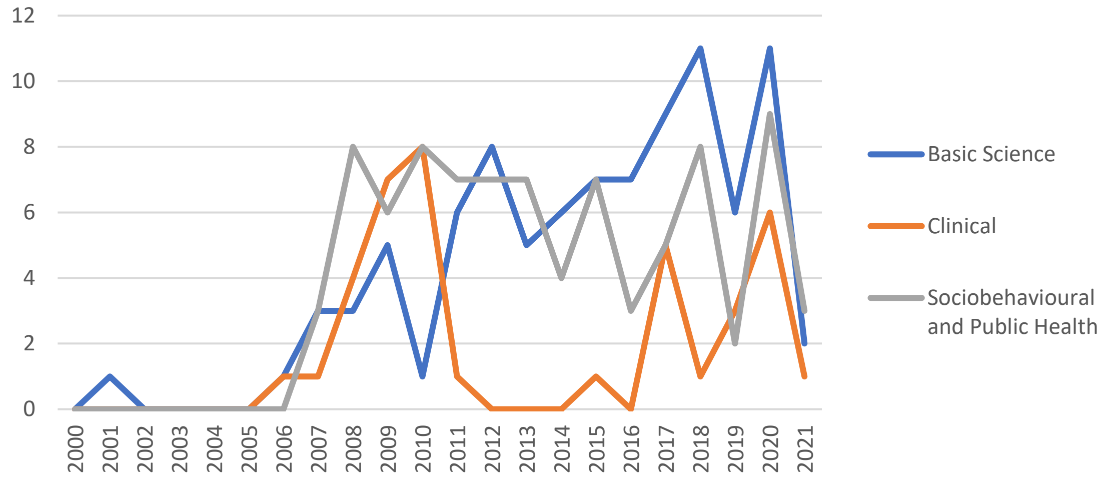

**data for 2021 (ongoing year) is as of August 2021*

Figure S3-19: Trend of number of publications of Indonesia based on type of research

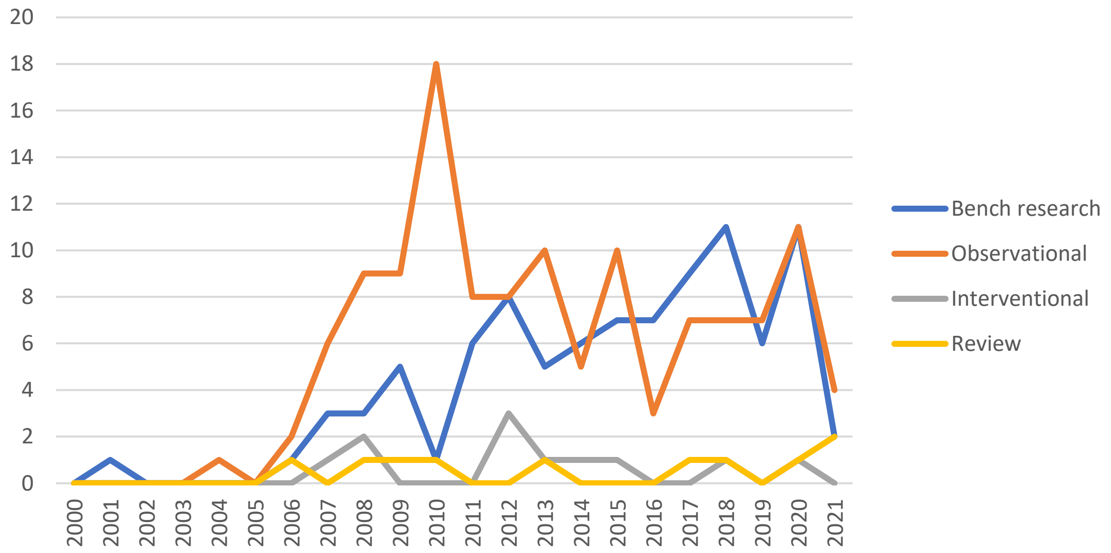

**data for 2021 (ongoing year) is as of August 2021*

Figure S3-20: Trend of number of publications of Indonesia based on study design

6. Maldives

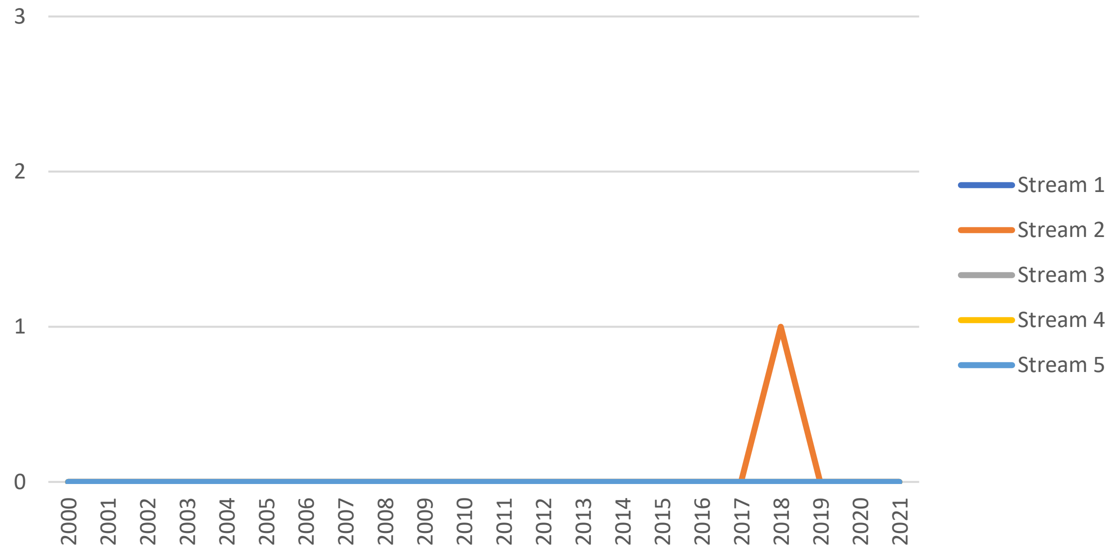

Figure S3-21: Trend of number of publications of Maldives based on WHO influenza streams
**data for 2021 (ongoing year) is as of August 2021*
Streams: 1-Reducing the risk of emergence of a pandemic influenza; 2-Limiting the spread of pandemic, zoonotic, and seasonal epidemic influenza; 3-Minimizing the impact of pandemic, zoonotic and seasonal epidemic influenza; 4-Optimizing the treatment of patients; 5-Promoting the development and application of new public health tools

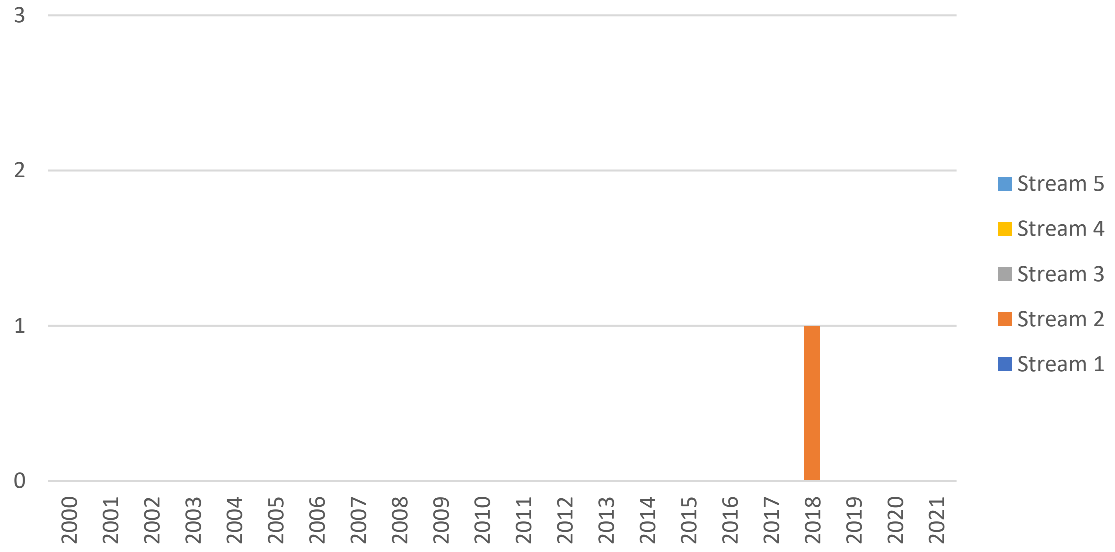

Figure S3-22: Stacked plot of number of publications of Maldives based on WHO influenza streams
**data for 2021 (ongoing year) is as of August 2021*
Streams: 1-Reducing the risk of emergence of a pandemic influenza; 2-Limiting the spread of pandemic, zoonotic, and seasonal epidemic influenza; 3-Minimizing the impact of pandemic, zoonotic and seasonal epidemic influenza; 4-Optimizing the treatment of patients; 5-Promoting the development and application of new public health tools

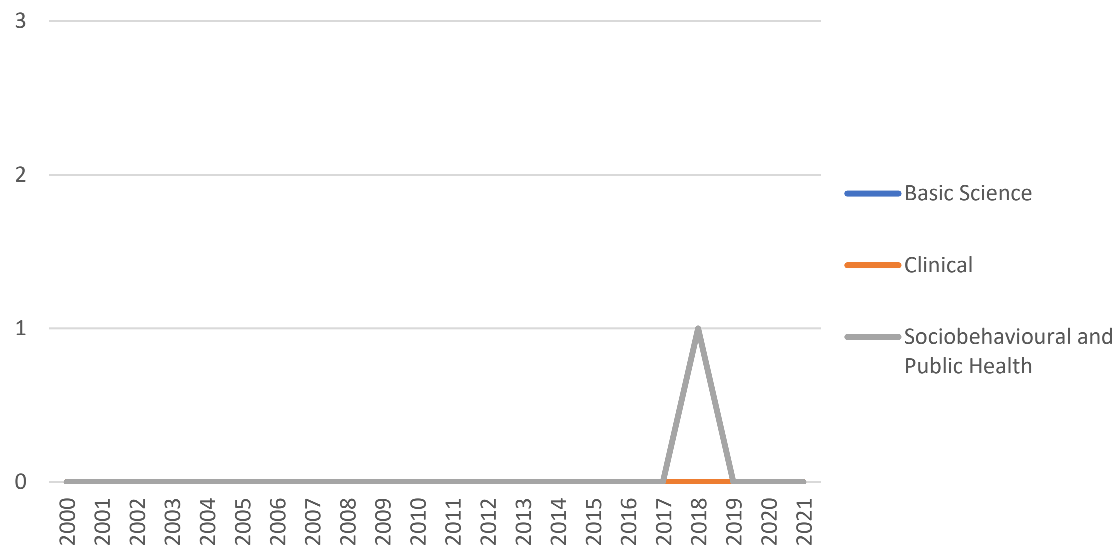

**data for 2021 (ongoing year) is as of August 2021*

Figure S3-23: Trend of number of publications of Maldives based on type of research

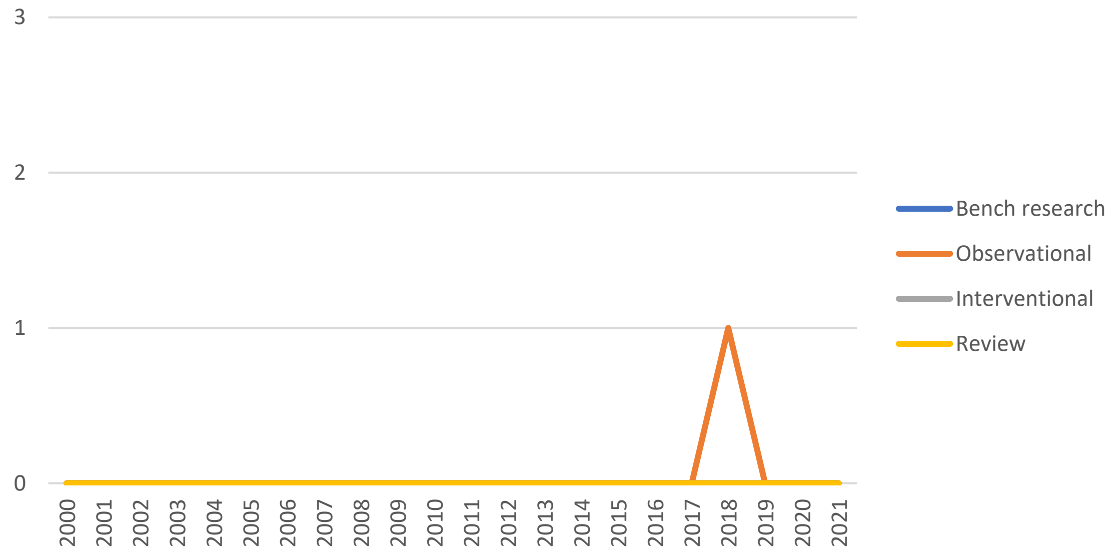

**data for 2021 (ongoing year) is as of August 2021*

Figure S3-24: Trend of number of publications of Bangladesh based on study design

7. Myanmar

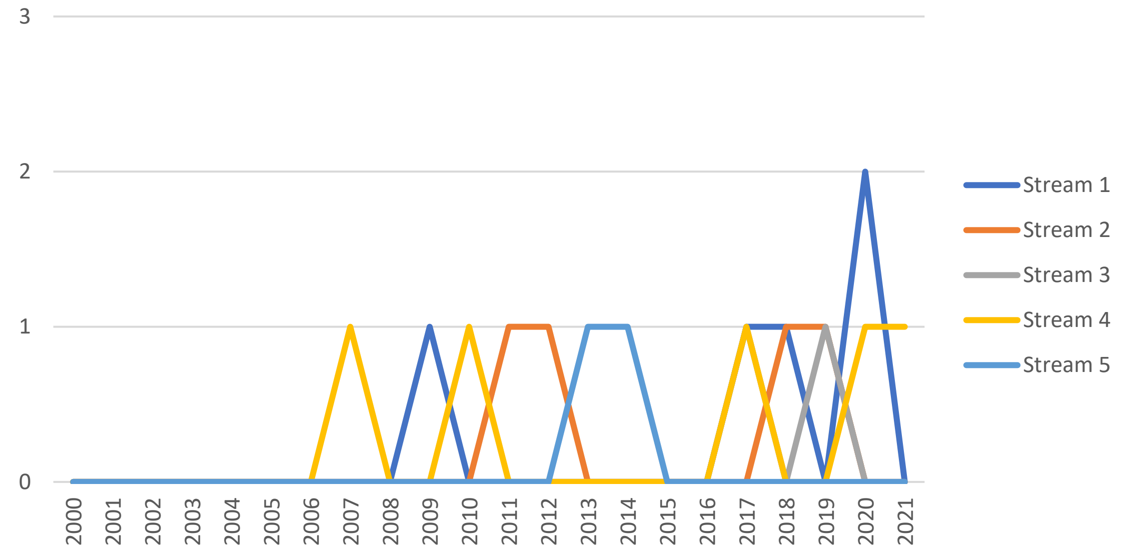

Figure S3-25: Trend of number of publications of Myanmar based on WHO influenza streams
**data for 2021 (ongoing year) is as of August 2021*
Streams: 1-Reducing the risk of emergence of a pandemic influenza; 2-Limiting the spread of pandemic, zoonotic, and seasonal epidemic influenza; 3-Minimizing the impact of pandemic, zoonotic and seasonal epidemic influenza; 4-Optimizing the treatment of patients; 5-Promoting the development and application of new public health tools

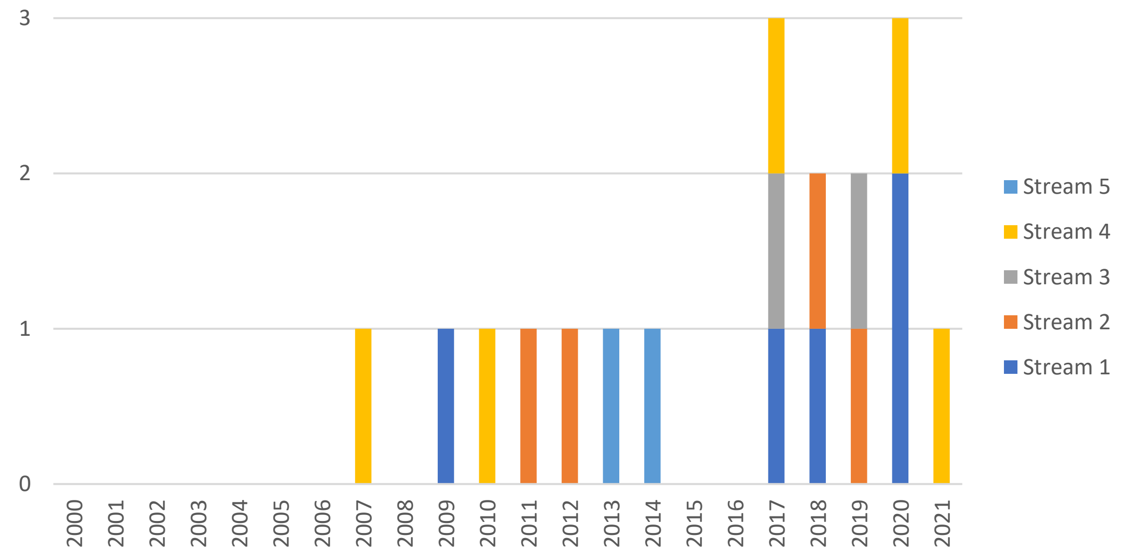

Figure S3-26: Stacked plot of number of publications of Myanmar based on WHO influenza streams
**data for 2021 (ongoing year) is as of August 2021*
Streams: 1-Reducing the risk of emergence of a pandemic influenza; 2-Limiting the spread of pandemic, zoonotic, and seasonal epidemic influenza; 3-Minimizing the impact of pandemic, zoonotic and seasonal epidemic influenza; 4-Optimizing the treatment of patients; 5-Promoting the development and application of new public health tools

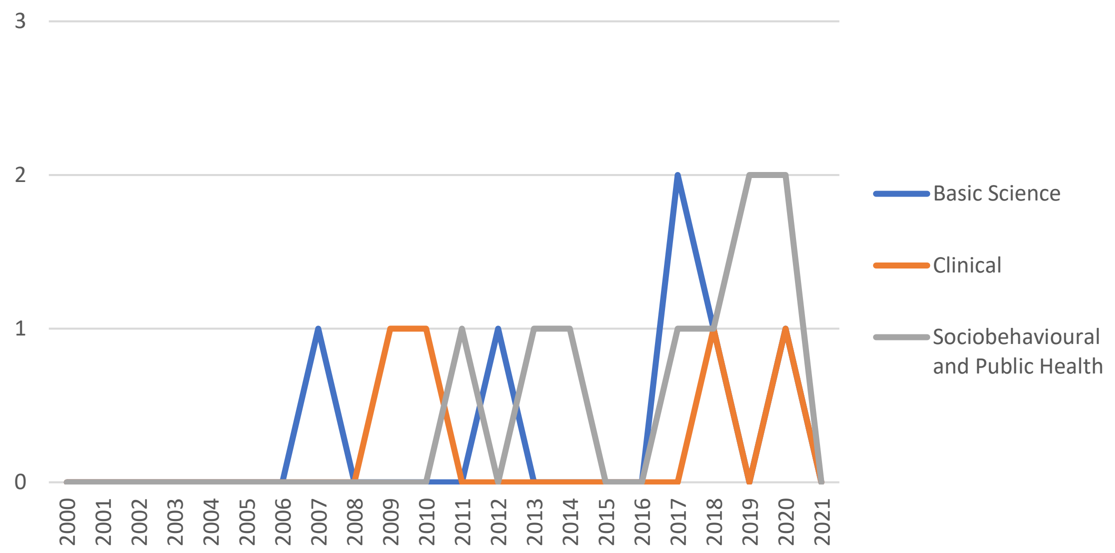

**data for 2021 (ongoing year) is as of August 2021*

Figure S3-27: Trend of number of publications of Myanmar based on type of research

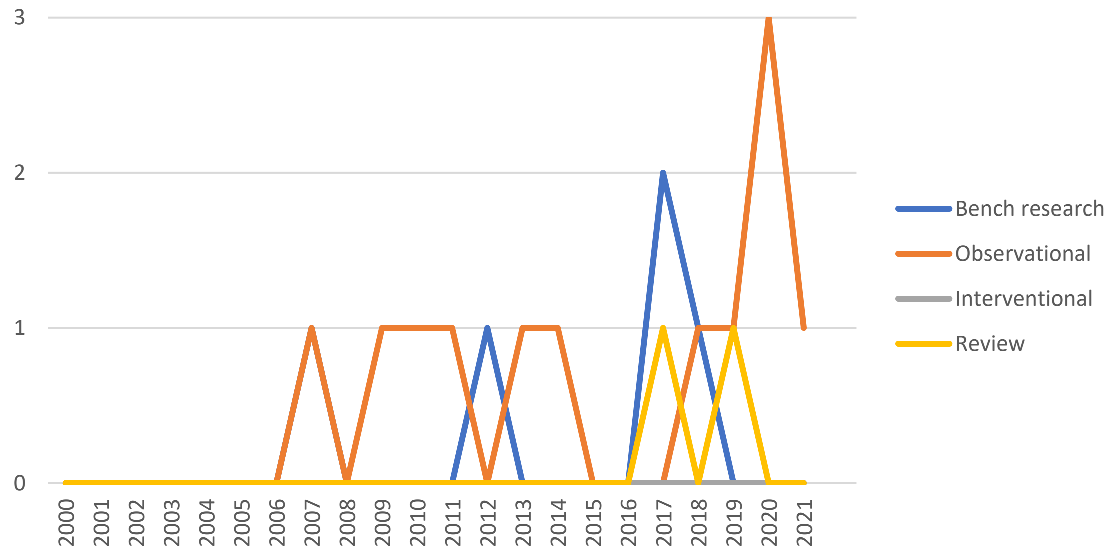

**data for 2021 (ongoing year) is as of August 2021*

Figure S3-28: Trend of number of publications of Myanmar based on study design

8. Nepal

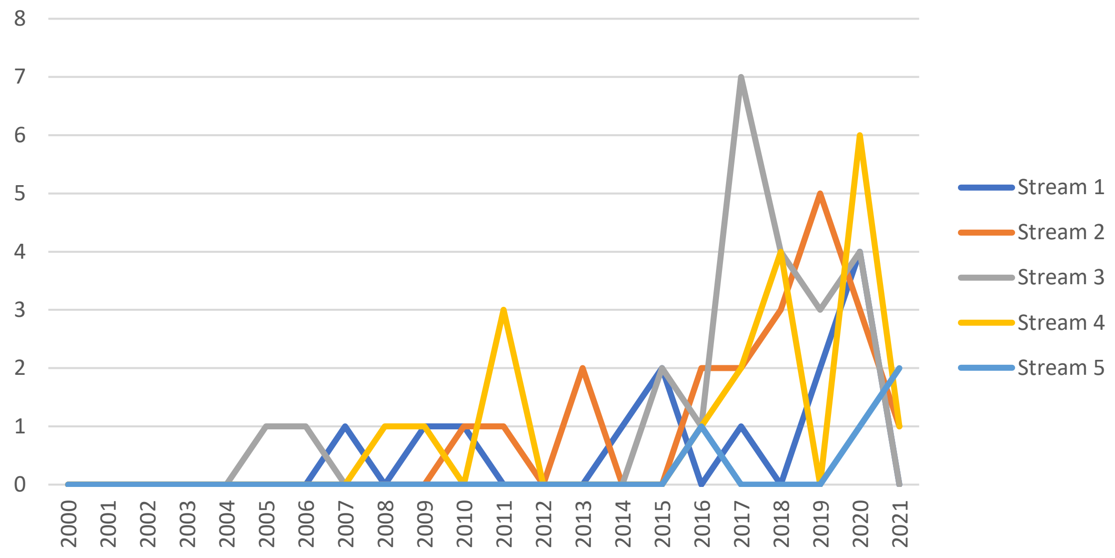

Figure S3-29: Trend of number of publications of Nepal based on WHO influenza streams
**data for 2021 (ongoing year) is as of August 2021*
Streams: 1-Reducing the risk of emergence of a pandemic influenza; 2-Limiting the spread of pandemic, zoonotic, and seasonal epidemic influenza; 3-Minimizing the impact of pandemic, zoonotic and seasonal epidemic influenza; 4-Optimizing the treatment of patients; 5-Promoting the development and application of new public health tools

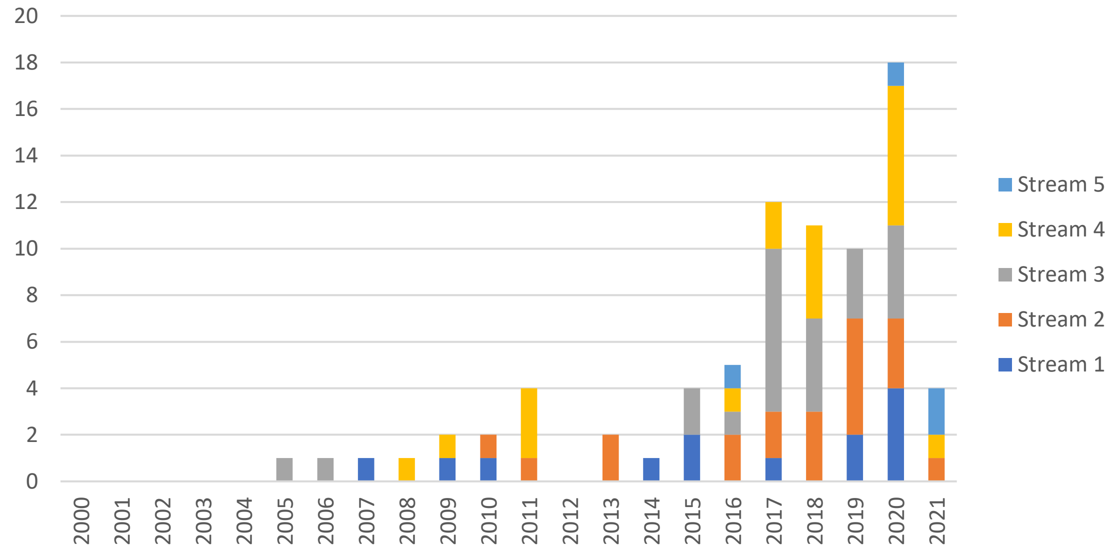

Figure S3-30: Stacked plot of number of publications of Nepal based on WHO influenza streams
**data for 2021 (ongoing year) is as of August 2021*
Streams: 1-Reducing the risk of emergence of a pandemic influenza; 2-Limiting the spread of pandemic, zoonotic, and seasonal epidemic influenza; 3-Minimizing the impact of pandemic, zoonotic and seasonal epidemic influenza; 4-Optimizing the treatment of patients; 5-Promoting the development and application of new public health tools

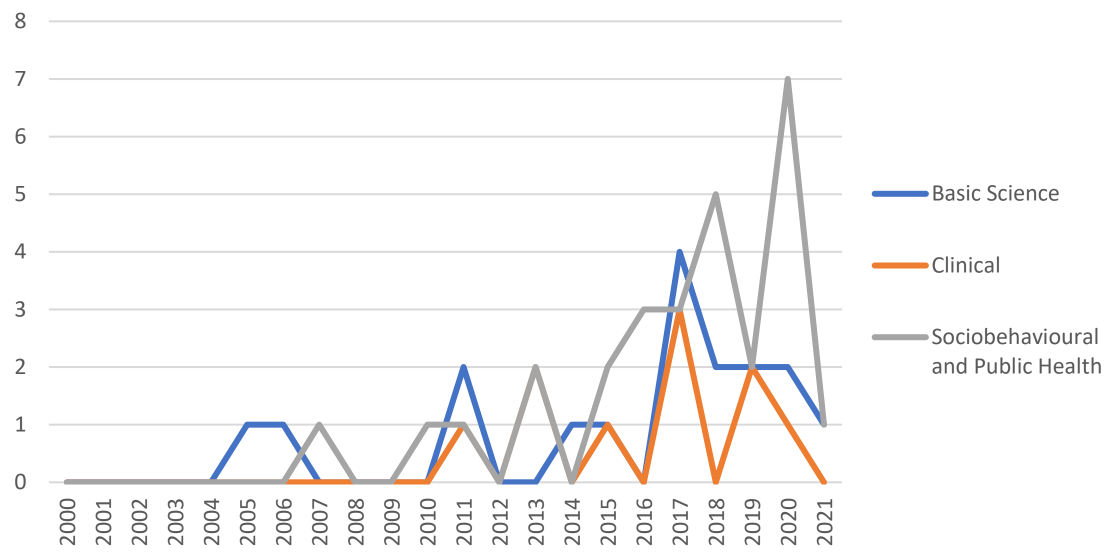

**data for 2021 (ongoing year) is as of August 2021*

Figure S3-31: Trend of number of publications of Nepal based on type of research

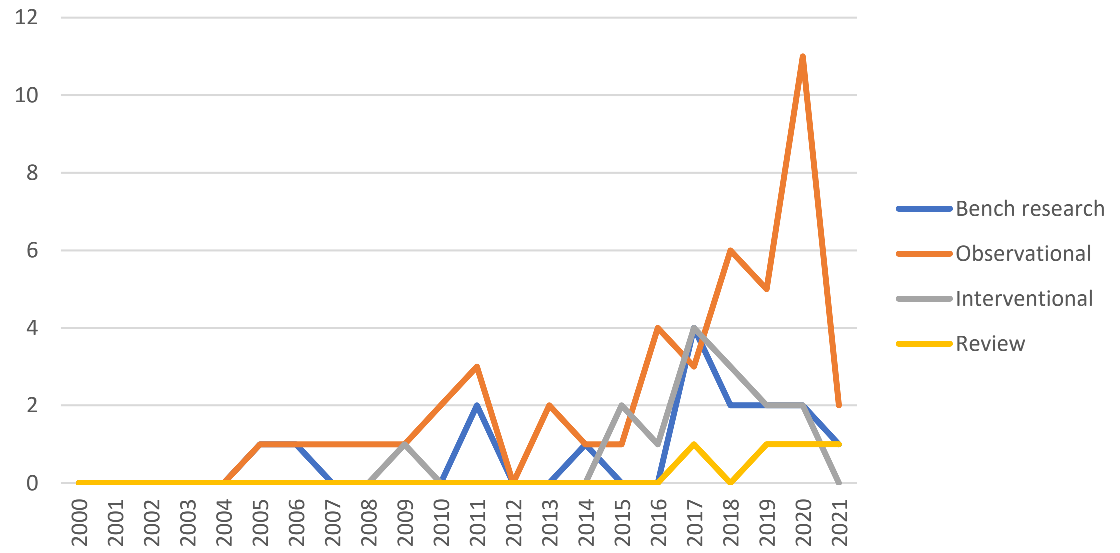

**data for 2021 (ongoing year) is as of August 2021*

Figure S3-32: Trend of number of publications of Nepal based on study design

9. Sri Lanka

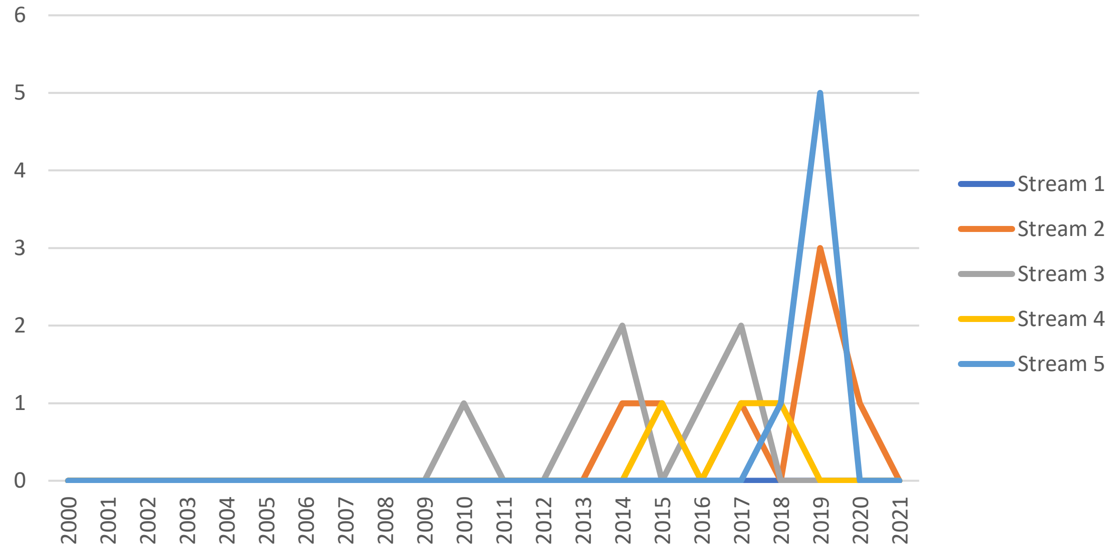

Figure S3-33: Trend of number of publications of Sri Lanka based on WHO influenza streams
**data for 2021 (ongoing year) is as of August 2021*
Streams: 1-Reducing the risk of emergence of a pandemic influenza; 2-Limiting the spread of pandemic, zoonotic, and seasonal epidemic influenza; 3-Minimizing the impact of pandemic, zoonotic and seasonal epidemic influenza; 4-Optimizing the treatment of patients; 5-Promoting the development and application of new public health tools

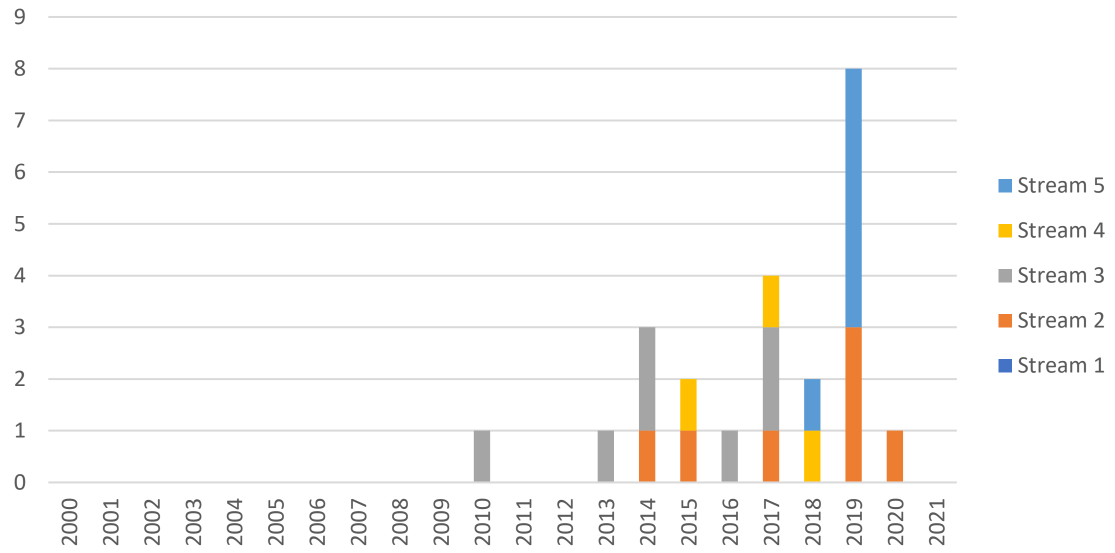

Figure S3-34: Stacked plot of number of publications of Sri Lanka based on WHO influenza streams
**data for 2021 (ongoing year) is as of August 2021*
Streams: 1-Reducing the risk of emergence of a pandemic influenza; 2-Limiting the spread of pandemic, zoonotic, and seasonal epidemic influenza; 3-Minimizing the impact of pandemic, zoonotic and seasonal epidemic influenza; 4-Optimizing the treatment of patients; 5-Promoting the development and application of new public health tools

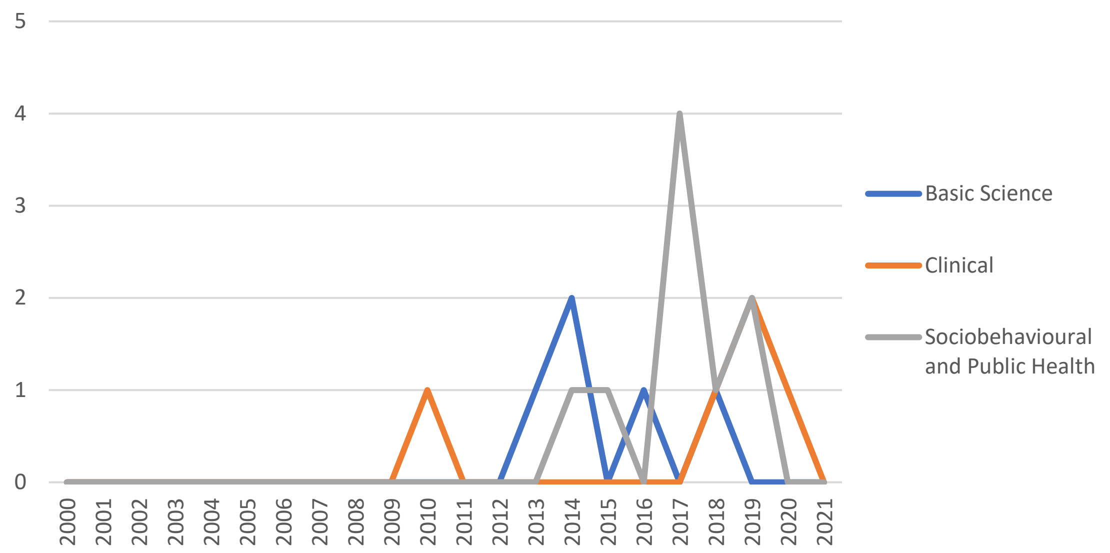

**data for 2021 (ongoing year) is as of August 2021*

Figure S3-35: Trend of number of publications of Sri Lanka based on type of research

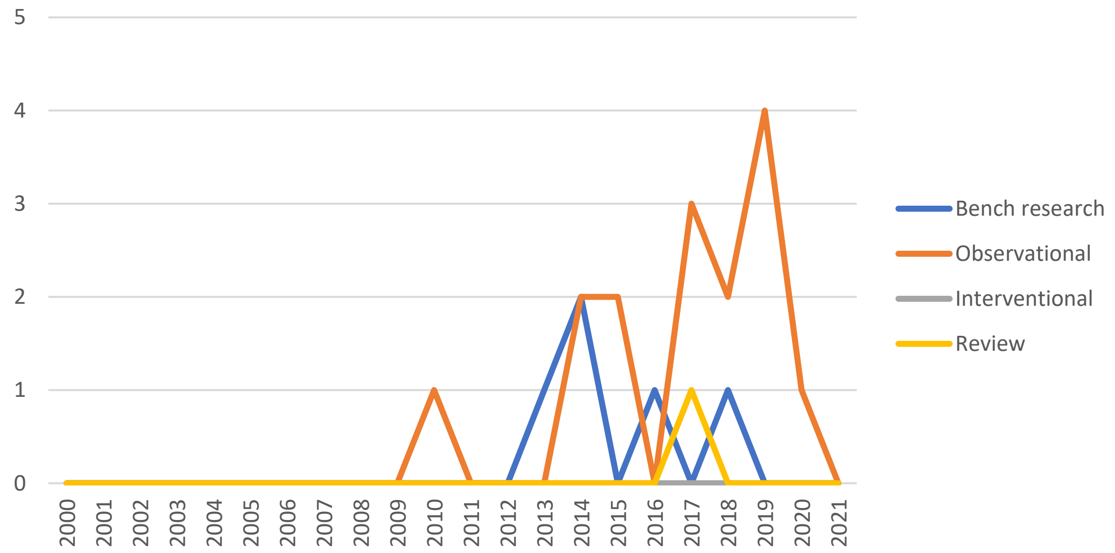

**data for 2021 (ongoing year) is as of August 2021*

Figure S3-36: Trend of number of publications of Sri Lanka based on study design

10. Thailand

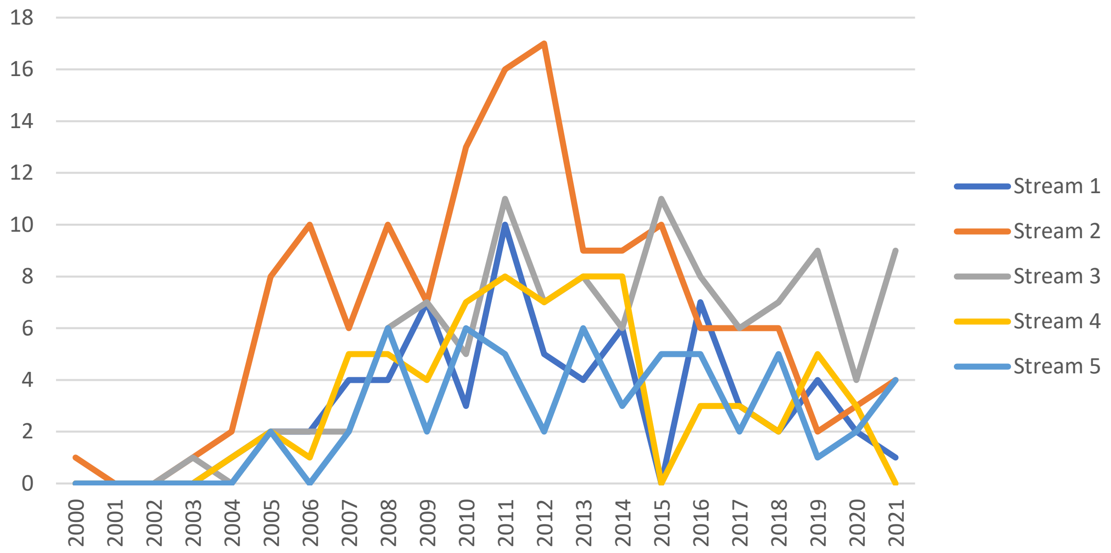

Figure S3-37: Trend of number of publications of Thailand based on WHO influenza streams
**data for 2021 (ongoing year) is as of August 2021*
Streams: 1-Reducing the risk of emergence of a pandemic influenza; 2-Limiting the spread of pandemic, zoonotic, and seasonal epidemic influenza; 3-Minimizing the impact of pandemic, zoonotic and seasonal epidemic influenza; 4-Optimizing the treatment of patients; 5-Promoting the development and application of new public health tools

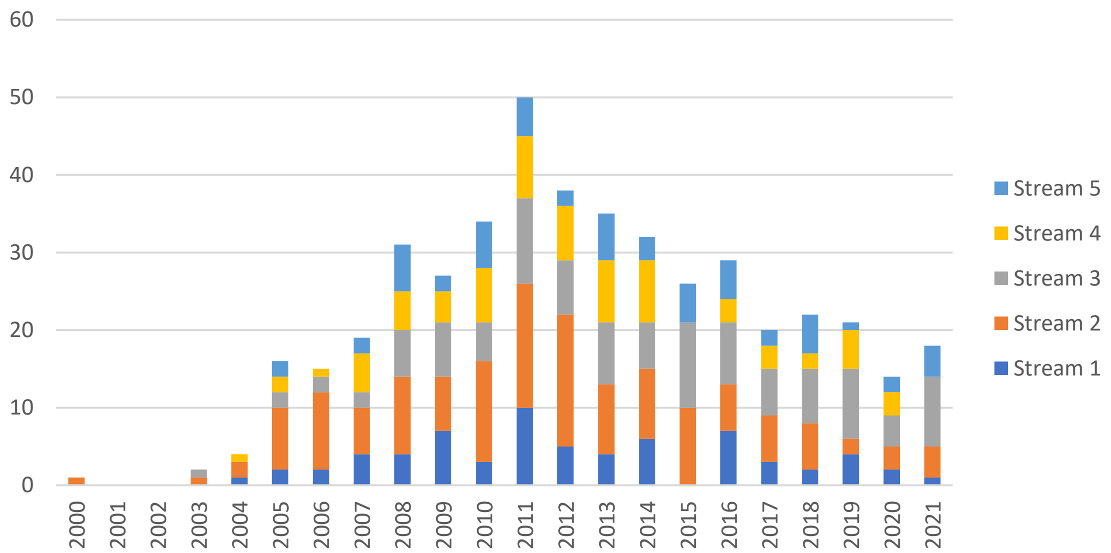

Figure S3-38: Stacked plot of number of publications of Thailand based on WHO influenza streams
**data for 2021 (ongoing year) is as of August 2021*
Streams: 1-Reducing the risk of emergence of a pandemic influenza; 2-Limiting the spread of pandemic, zoonotic, and seasonal epidemic influenza; 3-Minimizing the impact of pandemic, zoonotic and seasonal epidemic influenza; 4-Optimizing the treatment of patients; 5-Promoting the development and application of new public health tools

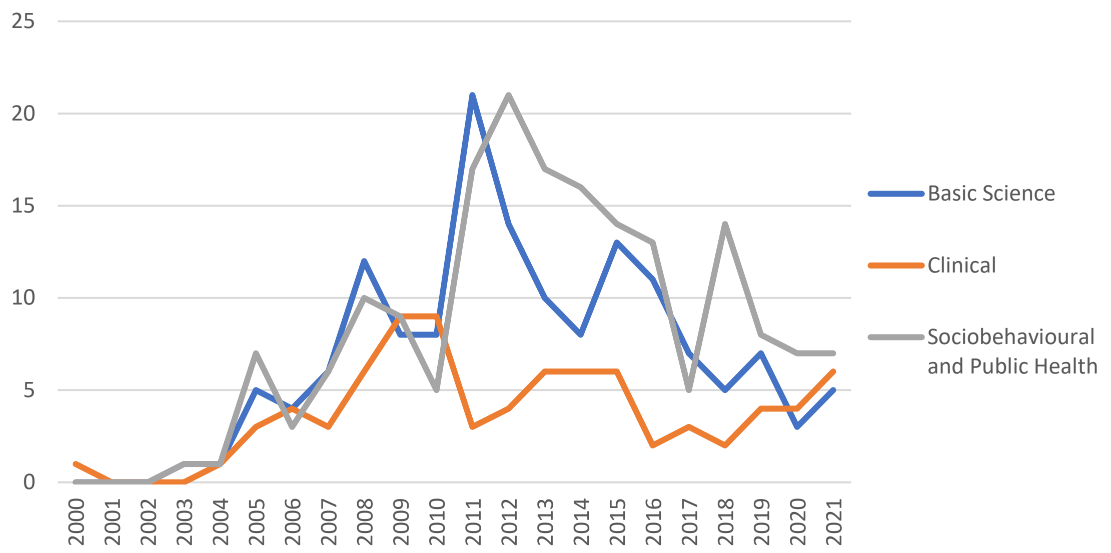

**data for 2021 (ongoing year) is as of August 2021*

Figure S3-39: Trend of number of publications of Thailand based on type of research

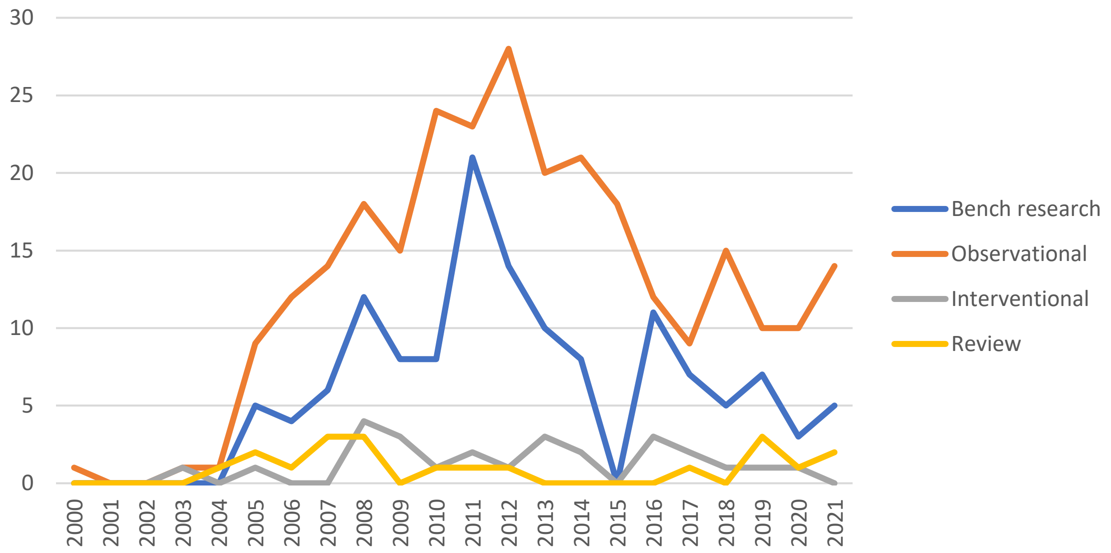

**data for 2021 (ongoing year) is as of August 2021*

Figure S3-40: Trend of number of publications of Thailand based on study design

11. Timor-Leste

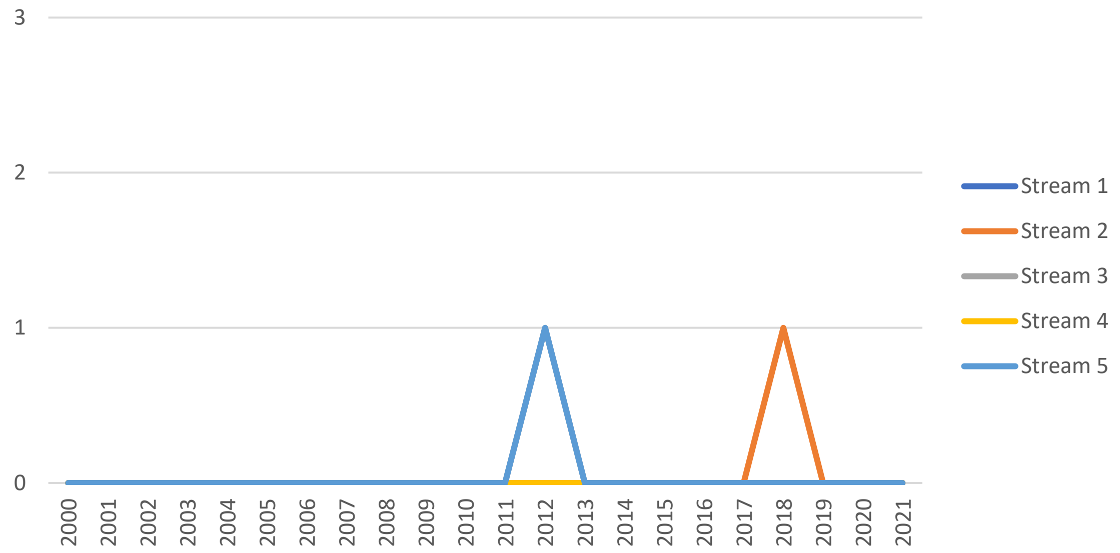

Figure S3-41: Trend of number of publications of Timor-Leste based on WHO influenza streams
**data for 2021 (ongoing year) is as of August 2021*
Streams: 1-Reducing the risk of emergence of a pandemic influenza; 2-Limiting the spread of pandemic, zoonotic, and seasonal epidemic influenza; 3-Minimizing the impact of pandemic, zoonotic and seasonal epidemic influenza; 4-Optimizing the treatment of patients; 5-Promoting the development and application of new public health tools

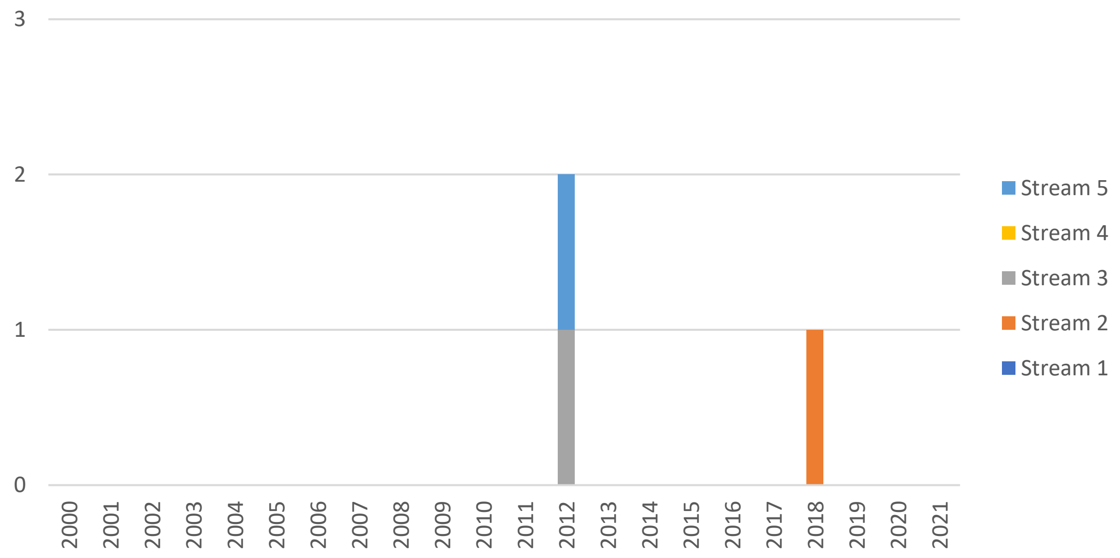

Figure S3-42: Stacked plot of number of publications of Timor-Leste based on WHO influenza streams
**data for 2021 (ongoing year) is as of August 2021*
Streams: 1-Reducing the risk of emergence of a pandemic influenza; 2-Limiting the spread of pandemic, zoonotic, and seasonal epidemic influenza; 3-Minimizing the impact of pandemic, zoonotic and seasonal epidemic influenza; 4-Optimizing the treatment of patients; 5-Promoting the development and application of new public health tools

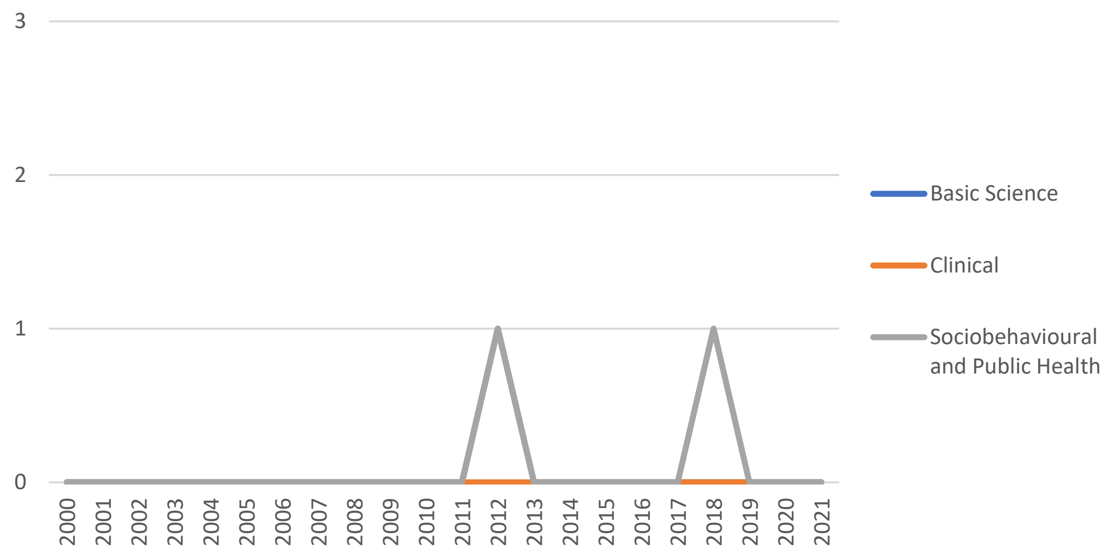

**data for 2021 (ongoing year) is as of August 2021*

Figure S3-43: Trend of number of publications of Timor-Leste based on type of research

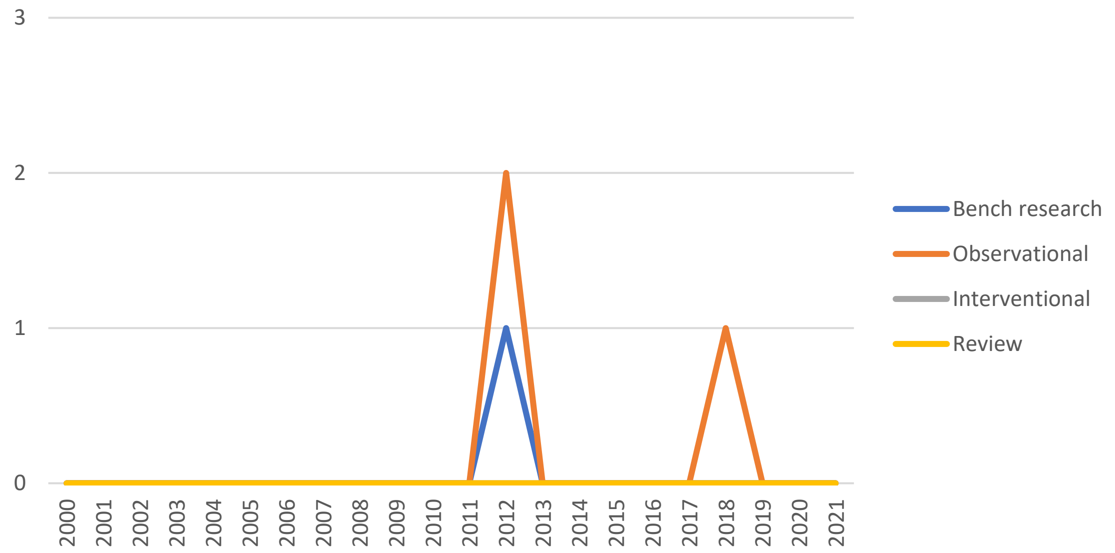

**data for 2021 (ongoing year) is as of August 2021*

Figure S3-44: Trend of number of publications of Timor-Leste based on study design

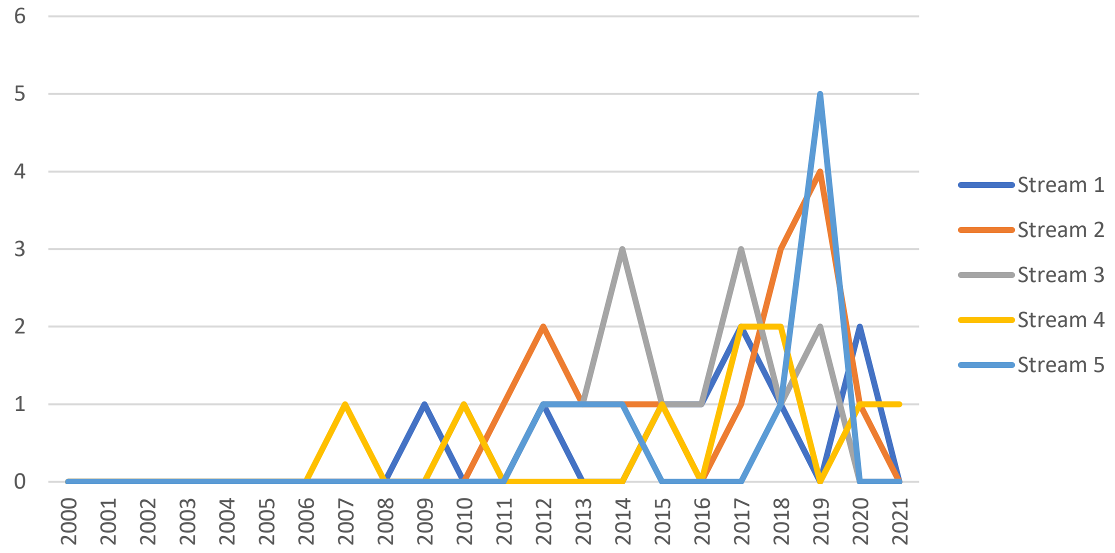

Figure S3-45: Trend of number of publications of 6 SEAR countries which are below 50th percentile based on WHO influenza streams

*6 SEAR countries – Myanmar, Sri Lanka, Bhutan, Timor-Leste, Maldives, DPR Korea.

**data for 2021 (ongoing year) is as of August 2021*

Streams: 1-Reducing the risk of emergence of a pandemic influenza; 2-Limiting the spread of pandemic, zoonotic, and seasonal epidemic influenza; 3-Minimizing the impact of pandemic, zoonotic and seasonal epidemic influenza; 4-Optimizing the treatment of patients; 5-Promoting the development and application of new public health tools

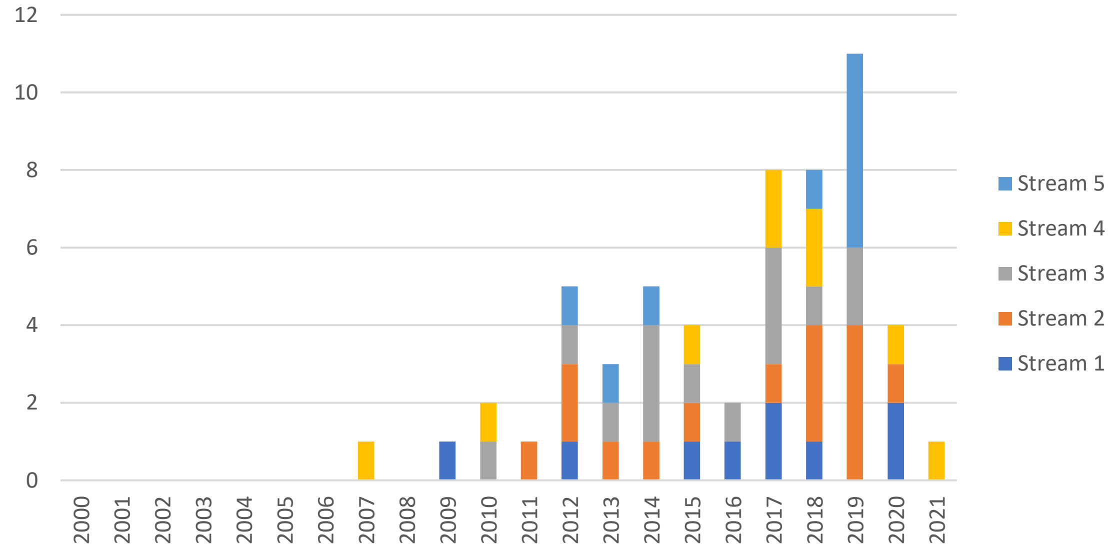

Figure S3-46: Stacked Plot of number of publications of 6 SEAR countries which are below 50th percentile based on WHO influenza streams

*6 SEAR countries – Myanmar, Sri Lanka, Bhutan, Timor-Leste, Maldives, DPR Korea.

**data for 2021 (ongoing year) is as of August 2021*

Streams: 1-Reducing the risk of emergence of a pandemic influenza; 2-Limiting the spread of pandemic, zoonotic, and seasonal epidemic influenza; 3-Minimizing the impact of pandemic, zoonotic and seasonal epidemic influenza; 4-Optimizing the treatment of patients; 5-Promoting the development and application of new public health tools

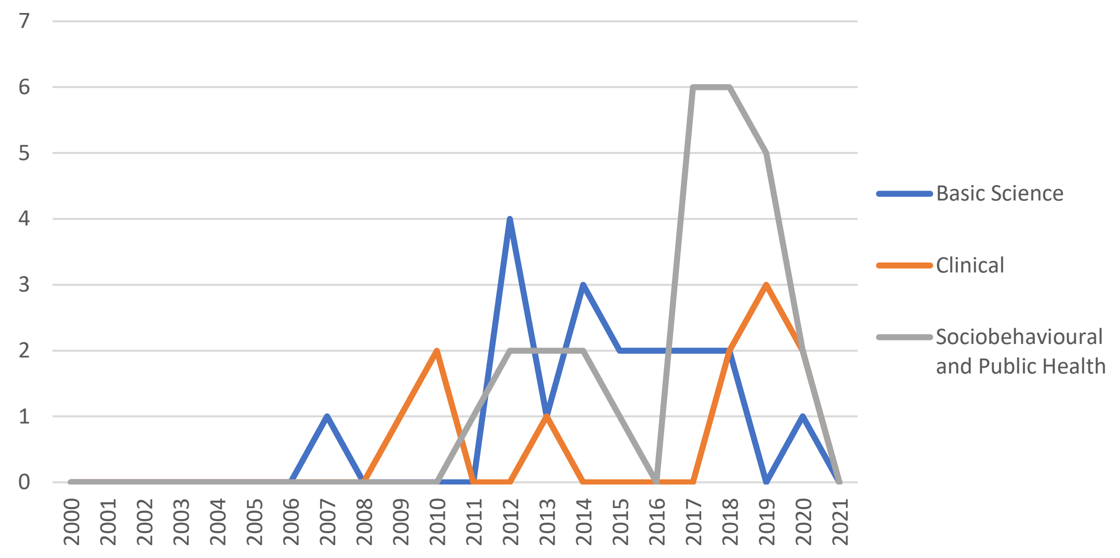

Figure S3-47: Trend of number of publications of 6 SEAR countries which are below 50th percentile based on type of research

*6 SEAR countries – Myanmar, Sri Lanka, Bhutan, Timor-Leste, Maldives, DPR Korea

**data for 2021 (ongoing year) is as of August 2021*

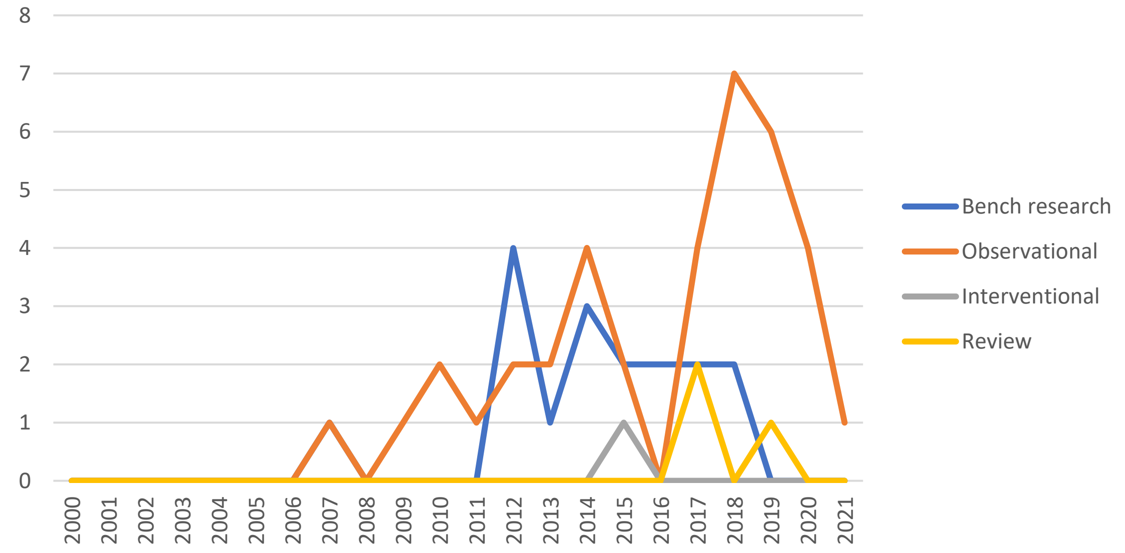

Figure S3-48: Trend of number of publications of 6 SEAR countries, which are below 50th percentile based on study design

*6 SEAR countries – Myanmar, Sri Lanka, Bhutan, Timor-Leste, Maldives, DPR Korea

**data for 2021 (ongoing year) is as of August 2021*

Supplementary File #4

Flow diagram

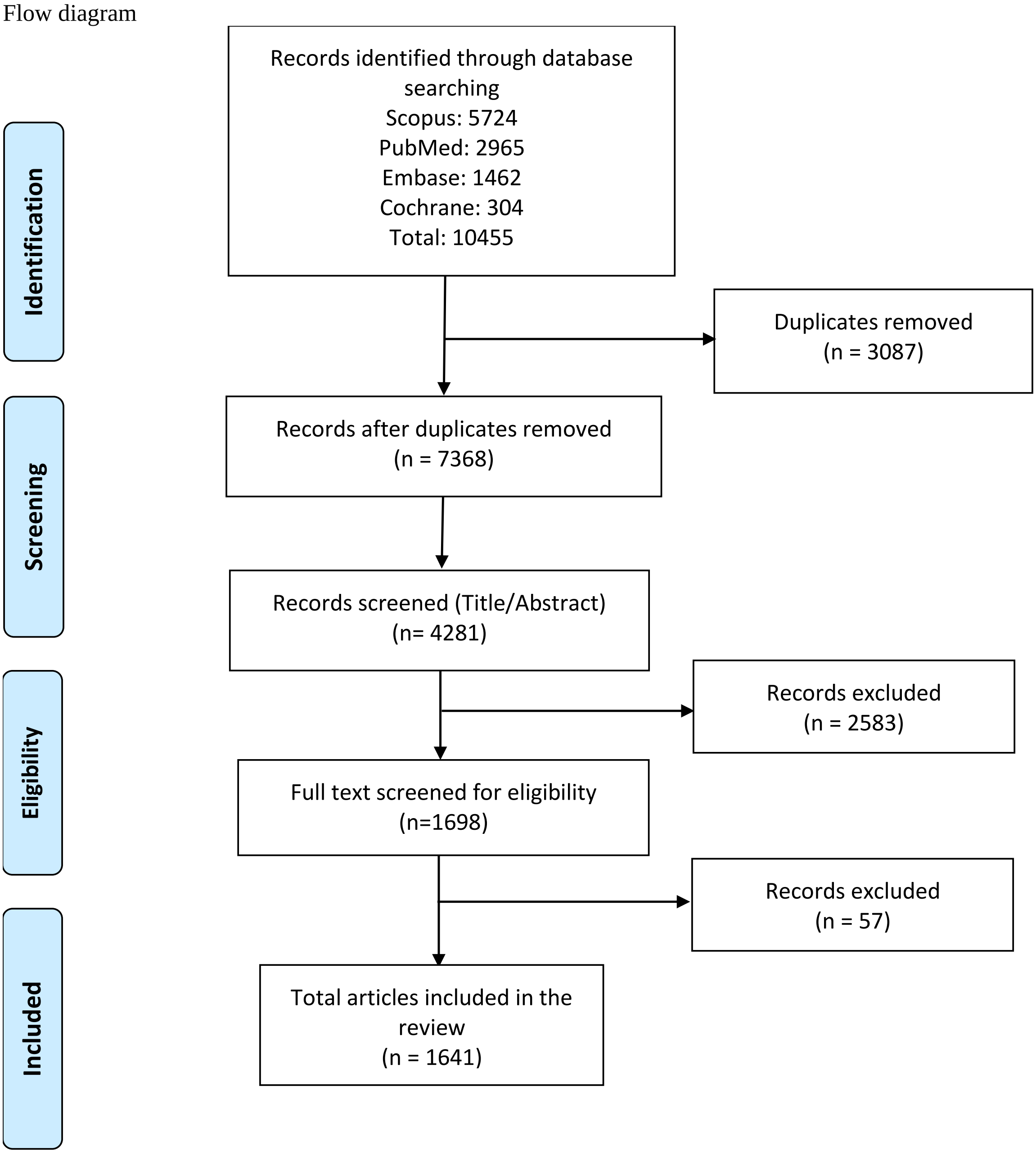

Figure S4-1: Flow diagram showing the selection and inclusion of the studies

File S4-2: Data extraction form

The file attached below is a data extraction form which was used to extract data for included articles (n=1641)

Data%20extraction%
20form.xlsx
