# Supplementary material for: An appraisal of peer-reviewed published literature on Influenza, 2000–2021 from countries in South-East Asia Region
Source: Front Public Health. 2023 Apr 17;11:1127891. doi: 10.3389/fpubh.2023.1127891 (PMC10149947; doi:10.3389/fpubh.2023.1127891)
Supplement: Supplementary file 2 [file Data_Sheet_2.docx]

**Bibliography of the included literature (N=1641)**

1. John TJ, Moorthy M. 2009 Pandemic influenza in India. Indian Pediatrics. 2010;47(1):25–31.

2. Kingsford C, Nagarajan N, Salzberg SL. 2009 Swine-origin influenza A (H1N1) resembles previous influenza isolates. PLoS One. 2009;4(7):e6402.

3. Mishra B. 2015 resurgence of influenza a (H1N1) 09: Smoldering pandemic in India? J Global Infect Dis. 2015;7(2):56–9.

4. Kadi AS, Avaradi SR. A bayesian inferential approach to quantify the transmission intensity of disease outbreak. Computational and Mathematical Methods in Medicine [Internet]. 2015;2015. Available from: https://www.scopus.com/inward/record.uri?eid=2-s2.0-84924263935&doi=10.1155%2f2015%2f256319&partnerID=40&md5=737a9a61b484da0210ae67d26a56b784

5. Bhardwaj SD, Potdar VA, Yadav PD, Chaudhary ML, Chadha MS, Mourya D. A case report of the enterovirus-D68 associated severe acute respiratory illness in a pediatric case from India. Journal of Infection and Public Health. 2019;12(6):900–3.

6. Ehrlich HJ, Müller M, Fritsch S, Zeitlinger M, Berezuk G, Löw-Baselli A, et al. A cell culture (Vero)-derived H5N1 whole-virus vaccine induces cross-reactive memory responses. J Infect Dis. 2009;200(7):1113–8.

7. Chokephaibulkit K, Uiprasertkul M, Puthavathana P, Chearskul P, Auewarakul P, Dowell SF, et al. A child with avian influenza A (H5N1) infection. Pediatr Infect Dis J. 2005;24(2):162–6.

8. Gupta BD, Purohit A. A clinical study of hospitalized H1N1 infected children in western Rajasthan. Journal of Tropical Pediatrics. 2011;57(2):87–90.

9. Kubavat AH, Mittal R, Patel PM, Jarsaniya DH, Pawar PR. A clinical trial to assess the immunogenicity and safety of Inactivated Influenza Vaccine (Whole Virion) IP (Pandemic Influenza (H1N1) 2009 Monovalent Vaccine; VaxiFlu-S ) in healthy Indian adult population. Journal of Postgraduate Medicine. 2011;57(2):102–8.

10. Thiansukhon E, Paitoonpong L, Suwanpimolkul G, Bhattarakosol P, Suankratay C. A comparative study between 2009 H1N1 influenza A virus and seasonal influenza virus infections at a Bangkok hospital, Thailand. Clin Microbiol Infect. 2010;16:S304.

11. Simmerman JM, Fry AM, Shinde V, Hanshaoworakul W, Narueponjirakul U, Areechokechai D, et al. A comparison of clinical and epidemiological characteristics of fatal human infections with H5N1 and human influenza viruses in Thailand, 2004-2006. PLoS ONE. 2011;6(4):e14809.

12. Rao R, Dhingra MS, Bavdekar S, Behera N, Daga SR, Dutta AK, et al. A comparison of immunogenicity and safety of indigenously developed liquid (DTwPHB-Hib) pentavalent combination vaccine (Shan 5) with Easyfive (liq) and TritanrixHB + Hiberix (lyo) in Indian infants administered according to the EPI schedule. Human Vaccines. 2009;5(6):425–9.

13. Takano R, Nidom CA, Kiso M, Muramoto Y, Yamada S, Shinya K, et al. A comparison of the pathogenicity of avian and swine H5N1 influenza viruses in Indonesia. Archives of Virology. 2009;154(4):677–81.

14. C T, Dv S, A S, V K, A K. A Concurrent Comparison of the Epidemiology and Clinical Presentation of Patients Hospitalized with Pandemic 2009 (H1N1) Influenza and Seasonal Influenza-A in Sub-himalayan Region of Himachal Pradesh. J Assoc Physicians India. 2019;67(3):70–4.

15. Rimi NA, Hassan MdZ, Chowdhury S, Rahman M, Sultana R, Biswas PK, et al. A decade of avian influenza in Bangladesh: Where are we now? Tropical Medicine and Infectious Disease [Internet]. 2019;4(3). Available from: https://www.scopus.com/inward/record.uri?eid=2-s2.0-85073028064&doi=10.3390%2ftropicalmed4030119&partnerID=40&md5=13fdd05d6a2816509315463bfc994c9e

16. Ravivarman L, Sugunan A, Kar SS, Devika S, Ganeshkumar P, Srividya V. A decade with climatic factors and seasonal activity of influenza A H1N1, Puducherry, India, 2009-2019. Int J Infect Dis. 2020;101:265–6.

17. Chandra S, Kassens-Noor E, Kuljanin G, Vertalka J. A geographic analysis of population density thresholds in the influenza pandemic of 1918-19. Int J Health Geogr. 2013;12:9.

18. Alexander V, Sam Paul G, Zachariah A, Mathuram A. A hospital-based nonconcurrent cohort study on factors associated with in-hospital mortality in patients with laboratory confirmed influenza. Journal of Global Infectious Diseases. 2020;12(4):208–13.

19. Clague B, Wannachaiwong Y, Olsen SJ, Chamany S, Burapat C, Simmerman JM, et al. A household survey to assess the burden of influenza in rural Thailand. Southeast Asian J Trop Med Public Health. 2006;37(3):488–93.

20. Moyen N, Ahmed G, Gupta S, Tenzin T, Khan R, Khan T, et al. A large-scale study of a poultry trading network in Bangladesh: Implications for control and surveillance of avian influenza viruses. BMC Veterinary Research [Internet]. 2018;14(1). Available from: https://www.scopus.com/inward/record.uri?eid=2-s2.0-85040446788&doi=10.1186%2fs12917-018-1331-5&partnerID=40&md5=b3b9d9a0ffb5bfa82effafa17984b691

21. Poltep K, Ketchim N, Paungpin W, Prompiram P, Sedwisai P, Chamsai T, et al. A long-term serosurvey of avian influenza H5 among wild birds in Nakhon Sawan Province, Thailand. Journal of Zoo and Wildlife Medicine. 2018;49(2):464–9.

22. Dawood FS, Fry AM, Muangchana C, Sanasuttipun W, Baggett HC, Chunsuttiwat S, et al. A method for estimating vaccine-preventable pediatric influenza pneumonia hospitalizations in developing countries: Thailand as a case study. Vaccine. 2011;29(26):4416–21.

23. Noordeen F, Pitchai FNN, Kudagammana ST, Rafeek RAM. A mini outbreak of human metapneumovirus infection with severe acute respiratory symptoms in a selected group of children presented to a teaching hospital in Sri Lanka. VirusDisease. 2019;30(2):307–10.

24. Gibbons RV, Nisalak A, Yoon IK, Tannitisupawong D, Rungsimunpaiboon K, Vaughn DW, et al. A model international partnership for community-based research on vaccine-preventable diseases: The Kamphaeng Phet-AFRIMS Virology Research Unit (KAVRU). Vaccine. 2013;31(41):4487–500.

25. Wibawa H, Henning J, Wong F, Selleck P, Junaidi A, Bingham J, et al. A molecular and antigenic survey of H5N1 highly pathogenic avian influenza virus isolates from smallholder duck farms in Central Java, Indonesia during 2007-2008. Virology Journal [Internet]. 2011;8. Available from: https://www.scopus.com/inward/record.uri?eid=2-s2.0-80052560138&doi=10.1186%2f1743-422X-8-425&partnerID=40&md5=cba6d4bbb38171697d570cc0b9690301

26. Velasco JM, Shrestha S, Valderama MT, Shrestha J, Shrestha B, Diones PC, et al. A multi-country field validation of the FluChip-8G Insight Assay. Journal of Virological Methods [Internet]. 2021;289. Available from: https://www.scopus.com/inward/record.uri?eid=2-s2.0-85097523440&doi=10.1016%2fj.jviromet.2020.114029&partnerID=40&md5=e575fc23e8cb44a5c8934cf75354e5dd

27. Kitphati R, Apisarnthanarak A, Chittaganpitch M, Tawatsupha P, Auwanit W, Puthavathana P, et al. A nationally coordinated laboratory system for human avian influenza A (H5N1) in Thailand: Program design, analysis, and evaluation. Clinical Infectious Diseases. 2008;46(9):1394–400.

28. Nooruzzaman M, Mumu TT, Hasnat A, Akter MN, Rasel MSU, Rahman MM, et al. A new reassortant clade 2.3.2.1a H5N1 highly pathogenic avian influenza virus causing recent outbreaks in ducks, geese, chickens and turkeys in Bangladesh. Transboundary and Emerging Diseases. 2019;66(5):2120–33.

29. Kode SS, Pawar SD, Tare DS, Keng SS, Hurt AC, Mullick J. A novel I117T substitution in neuraminidase of highly pathogenic avian influenza H5N1 virus conferring reduced susceptibility to oseltamivir and zanamivir. Veterinary Microbiology. 2019;235:21–4.

30. Chaiwarith R, Prommee N, Liwsrisakun C, Oberdorfer P, Nuntachit N, Pothirat C. A novel influenza A H1N1 clinical manifestations in patients at Chiang Mai university hospital. Journal of the Medical Association of Thailand. 2011;94(8):908–15.

31. Dhere R, Yeolekar L, Kulkarni P, Menon R, Vaidya V, Ganguly M, et al. A pandemic influenza vaccine in India: From strain to sale within 12 months. Vaccine. 2011;29:A16–21.

32. Yano T, Phornwisetsirikun S, Susumpow P, Visrutaratna S, Chanachai K, Phetra P, et al. A participatory system for preventing pandemics of animal origins: Pilot study of the participatory one health disease detection (podd) system. JMIR Public Health and Surveillance [Internet]. 2018;20(3). Available from: https://www.scopus.com/inward/record.uri?eid=2-s2.0-85047731343&doi=10.2196%2fpublichealth.7375&partnerID=40&md5=0f34cc1f46a8f96b4991b90a8fcf12ee

33. Michael JS, Sridharan G. A pilot study of seroprevalence of influenza virus type A in Vellore, south India. Indian J Med Res. 2002;115:173–5.

34. Kulkarni PS, Raut SK, Dhere RM. A post-marketing surveillance study of a human live-virus pandemic influenza A (H1N1) vaccine (Nasovac®) in India. Human Vaccines and Immunotherapeutics. 2013;9(1):122–4.

35. de Silva UC, Warachit J, Waicharoen S, Chittaganpitch M. A preliminary analysis of the epidemiology of influenza A(H1N1)v virus infection in Thailand from early outbreak data, June-July 2009. Euro surveillance : bulletin européen sur les maladies transmissibles = European communicable disease bulletin [Internet]. 2009;14(31). Available from: https://www.scopus.com/inward/record.uri?eid=2-s2.0-70349331581&doi=10.2807%2fese.14.31.19292-en&partnerID=40&md5=ec2beb86977855e407c8814ce07000fe

36. Broor S, Parveen S, Bharaj P, Prasad VS, Srinivasulu KN, Sumanth KM, et al. A prospective three-year cohort study of the epidemiology and virology of acute respiratory infections of children in rural India. PLoS ONE. 2007;2(6):e491.

37. Chattopadhyay K, Fournie G, Pfeiffer DU, Abul Kalam M, Biswas PK, Hoque A, et al. A Qualitative Stakeholder Analysis of Avian Influenza Policy in Bangladesh. Ecohealth. 2018;15(1):63–71.

38. Ahankari AS, Myles PR, Tsang S, Khan F, Atre S, Langley T, et al. A qualitative study exploring factors influencing clinical decision-making for influenza-like illness in Solapur city, Maharashtra, India. Anthropology and Medicine. 2019;26(1):65–86.

39. Kongpatanakul S, Chatsiricharoenkul S, Panich U, Sathirakul K, Pongnarin P, Sangvanich P. A randomized, open-label, 2-period, crossover bioequivalence study of two oral formulations of 75 mg oseltamivir in healthy Thai volunteers. International Journal of Clinical Pharmacology and Therapeutics. 2008;46(12):654–62.

40. Lu H, Khurana S, Verma N, Manischewitz J, King L, Beigel JH, et al. A rapid Flp-In system for expression of secreted H5N1 influenza hemagglutinin vaccine immunogen in mammalian cells. PLoS One. 2011;6(2):e17297.

41. Arunorat J, Charoenvisal N, Keawcharoen J, Sreta D, Amonsin A, Thanawongnuwech R. A reassortant virus of A Thai Swine Influenza Virus (SIV) and the pandemic H1N1 of pig origin did not induce severe disease in experimental ducks. Thai Journal of Veterinary Medicine. 2014;44(3):335–43.

42. Pagadala, Sindu H. A Retrospective Study of Swine Flu Patients Presenting to the Emergency Department in a Tertiary Center in South India. J Emerg Med. 2020;58(4):715.

43. Jegin P, Dinesh Kumar M, Dayana J, Elakiya E, Subbulakshmi S. A retrospective study to compute the prevalence of pneumonia and its associated risk factors among children in selected tertiary care hospital, Kelambakkam, Kancheepuram, Tamilnadu, India. Indian J Public Health Res Dev. 2020;11(3):136–8.

44. Pitisuttithum P, Wirachwong P. A review of epidemic preparedness for influenza through local vaccine production: national security for Thailand. Human Vaccines and Immunotherapeutics. 2019;15(10):2440–5.

45. Amber R, Adnan M, Tariq A, Mussarat S. A review on antiviral activity of the Himalayan medicinal plants traditionally used to treat bronchitis and related symptoms. J Pharm Pharmacol. 2017;69(2):109–22.

46. Leung VKY, Deng YM, Todd A, Peck H, Buettner I, Zakis T, et al. A second external quality assessment of isolation and identification of influenza viruses in cell culture in the Asia Pacific region highlights improved performance by participating laboratories. Journal of Clinical Virology [Internet]. 2021;142. Available from: https://www.scopus.com/inward/record.uri?eid=2-s2.0-85110193812&doi=10.1016%2fj.jcv.2021.104907&partnerID=40&md5=6cafc80593acac1db5b0e7bdbc2d3c4d

47. Raj NW, Bhuvaneshwari R, Shenbagapraba N. A study “to assess the level of knowledge on swine flu among adults in selected area at Poonchery, Kanchipuram district, Tamilnadu. Medico-Legal Update. 2020;20(1):135–7.

48. Agrawal A, Agarwal S, Kumar V, Nawal CL, Mital P, Chejara R. A study of an influenza A (H1N1)pdm09 outbreak in pregnant women in Rajasthan, India. International Journal of Gynecology and Obstetrics. 2016;132(2):146–50.

49. Mudhigeti N, Racherla R, Mahalakshmi P, Pamireddy M, Nallapireddy U, Kante M, et al. A study of influenza 2017-2018 outbreak in Andhra Pradesh, India. Indian Journal of Medical Microbiology. 2018;36(4):526–31.

50. Kumar S. A study of outbreak of swine flu (H1N1) in north - West zone of Rajasthan (current status - 2015). Journal of Association of Physicians of India. 2016;64:46–9.

51. Abinaya S, Gaspar BL, Benjamin AT. A study on aetiology and outcomes of viral lower respiratory tract infections in hospitalized children from South India. Sri Lanka Journal of Child Health. 2020;49(3):218–22.

52. Shilpa K, Praveen Kumar BA, Yogesh Kumar S, Ugargol AR, Naik VA, Mallapur MD. A study on awareness regarding swine flu (influenza A H1N1) pandemic in an urban community of Karnataka. Medical Journal of Dr DY Patil University. 2014;7(6):732–7.

53. Datta S, Sen S, Sengupta B. A study on knowledge and practice related to bird flu in a rural community of Hooghly District of West Bengal. Indian J Public Health. 2010;54(4):216–8.

54. Limaye D, Limaye V, Fortwengel G. A study to assess the vaccination coverage of university students in Mumbai, India. Int J Pharm Sci Res. 2017;8(6):2667–76.

55. Hanafusa S, Muhadir A, Santoso H, Tanaka K, Anwar M, Sulistyo ET, et al. A surveillance model for human avian influenza with a comprehensive surveillance system for local-priority communicable diseases in South Sulawesi, Indonesia. Tropical Medicine and Health. 2012;40(4):141–7.

56. Pramuwidyatama MG, Hogeveen H, Saatkamp HW. A systematic evaluation of measures against highly pathogenic avian influenza (HPAI) in Indonesia. Front Vet Sci. 2019;6:00033.

57. Chada KE, Forshee R, Golding H, Anderson S, Yang H. A systematic review and meta-analysis of cross-reactivity of antibodies induced by oil-in-water emulsion adjuvanted influenza H5N1 virus monovalent vaccines. Vaccine. 2017;35(24):3162–70.

58. Halton K, Sarna M, Barnett A, Graves N, Leonardo L. A systematic review of community-based interventions for emerging zoonotic infectious diseases in Southeast Asia. JBI Database Syst Rev Implement Rep. 2013;11(2):1–235.

59. Farooq QUA, Shaukat Z, Aiman S, Zhou T, Li C. A systems biology-driven approach to construct a comprehensive protein interaction network of influenza A virus with its host. BMC Infect Dis. 2020;20(1):480.

60. Bulu PM, Robertson ID, Geong M. A targeted investigation to demonstrate the freedom of West Timor from HPAI H5N1. Preventive Veterinary Medicine. 2018;150:47–51.

61. Chudasama R, Patel U, Verma P, Banerjee A, Buch P, Patel P. A Two Wave Analysis of Hospitalizations and Mortality from Seasonal and Pandemic 2009 A (H1N1) Influenza in Saurashtra, India: 2009-2011. Ann Med Health Sci Res. 2013;3(3):334–40.

62. Cherian SS, Chakrabarti AK, Pawar SD, Jadhav SM, Pal B, Raut S, et al. A unique influenza A (H5N1) virus causing a focal poultry outbreak in 2007 in Manipur, India. Virol J. 2009;6:26.

63. Gutiérrez RA, Naughtin MJ, Horm SV, San S, Buchy P. A(H5N1) Virus Evolution in South East Asia. Viruses. 2009;1(3):335–61.

64. Buddhari D, Gibbons RV, Yoon IK, Love CS, Heil GL, Rothman AL, et al. Absence of neutralizing antibodies against influenza A/H5N1 virus among children in Kamphaeng Phet, Thailand. J Clin Virol. 2015;69:78–80.

65. Chotpitayasunondh C, Patrasuwan S, Prontri M, Poiynok S. Acceptance of pandemic influenza A (H1N1) 2009 vaccine among health care workers, Thailand. BMC Proc. 2011;5.

66. Kabiraj CK, Mumu TT, Chowdhury EH, Islam MR, Parvin R, Beer M, et al. Active virological surveillance in backyard ducks in Bangladesh: detection of avian influenza and gammacoronaviruses. Avian Pathol. 2020;49(4):361–8.

67. Purwitasari N, Agil M, Studiawan H. Activity of ethyl acetate fraction of merremia mammosa hall as anti-influenza a (H1N1). Indian Journal of Forensic Medicine and Toxicology. 2020;14(3):2070–3.

68. Tillekeratne LG, Simmons R, Vanderburg S, Nicholson BP, Park LP, Ostbye T, et al. Acute respiratory viral infections: An important cause of admissions for acute febrile illness in the southern province, Sri Lanka. Am J Trop Med Hyg. 2018;99(4):411.

69. Deng YM, Iannello P, Komadina N, Barr IG, Hurt AC. Adamantane resistance in influenza A(H1) viruses increased in 2007 in South East Asia but decreased in Australia and some other countries. Antiviral Res. 2008;80(2):200–5.

70. Sommanustweechai A, Iamsirithaworn S, Patcharanarumol W, Kalpravidh W, Tangcharoensathien V. Adoption of One Health in Thailand’s National strategic plan for emerging infectious diseases. Journal of Public Health Policy. 2017;38(1):121–36.

71. Jairaj A, Shirisha P, Abdul MSM, Fatima U, Tiwari RVC, Moothedath M. Adult Immunization - Need of the Hour. J Int Soc Prev Community Dent. 2018;8(6):475–81.

72. Midha D, Kumar A, Vasudev P, Iqbal ZA, Mandal AK. Adult influenza A (H1N1) related encephalitis: A case report. Indian Journal of Critical Care Medicine. 2018;22(5):384–7.

73. Berlanda Scorza F. Advancing new vaccines against pandemic influenza in low-resource countries. Vaccine. 2017;35(40):5397–402.

74. Anovadiya AP, Barvaliya MJ, Shah RA, Ghori VM, Sanmukhani JJ, Patel TK, et al. Adverse drug reaction profile of oseltamivir in Indian population: A prospective observational study. Indian Journal of Pharmacology. 2011;43(3):258–61.

75. Gasparini R, Amicizia D, Lai PL, Panatto D. Aflunov(®): a prepandemic influenza vaccine. Expert Rev Vaccines. 2012;11(2):145–57.

76. Dudley JP, Mackay IM. Age-specific and sex-specific morbidity and mortality from avian influenza A(H7N9). J Clin Virol. 2013;58(3):568–70.

77. Peyre M, Chevalier V, Paul MC, Gilbert M, Desvaux S, Andriamanivo HR, et al. Agro-environmental determinants of avian influenza circulation: A multisite study in Thailand, Vietnam and Madagascar. PLoS ONE. 2014;9(7):e101958.

78. Jacob A, Sood R, Chanu KV, Bhatia S, Khandia R, Pateriya AK, et al. Amantadine resistance among highly pathogenic avian influenza viruses (H5N1) isolated from India. Microbial Pathogenesis. 2016;91:35–40.

79. Bai GR, Kanai Y, Li YG, Ikuta K, Chittaganpitch M, Auwanit W, et al. Amantadine- and oseltamivir-resistant variants of influenza A viruses in Thailand. Biochem Biophys Res Commun. 2009;390(3):897–901.

80. Kosoltanapiwat N, Boonyuen U, Pooruk P, Iamsirithaworn S, Mungaomklang A, Chokephaibulkit K, et al. Amino acid substitutions in hemagglutinin of the 2009 pandemic influenza A(H1N1) viruses that might affect the viral antigenicity. BMC Res Notes. 2014;7:951.

81. Levie K, Leroux-Roels I, Hoppenbrouwers K, Kervyn AD, Vandermeulen C, Forgus S, et al. An adjuvanted, low-dose, pandemic influenza A (H5N1) vaccine candidate is safe, immunogenic, and induces cross-reactive immune responses in healthy adults. J Infect Dis. 2008;198(5):642–9.

82. Hinjoy S, Smithsuwan P, Wongkumma A. An analytical study of behavioral risks and illness among camel keeper and non-camel keeper at zoo parks in Thailand 2014. Int J Infect Dis. 2016;45:245.

83. Suenaga E, Kumar PKR. An aptamer that binds efficiently to the hemagglutinins of highly pathogenic avian influenza viruses (H5N1 and H7N7) and inhibits hemagglutinin-glycan interactions. Acta Biomater. 2014;10(3):1314–23.

84. Srinivas P, Bhattacharyya D, Chakkaravarthy DM. An artificial intelligent based system for efficient swine flu prediction using naive bayesian classifier. International Journal of Current Research and Review. 2020;12(15):134–9.

85. Islam K, Murshidul Ahsan M, Chakma S, Penjor K, Barua M, Jalal MS, et al. An assessment on potential risk pathways for the incursion of highly pathogenic avian influenza virus in backyard poultry farm in Bangladesh. Veterinary World. 2020;13(10):2104–11.

86. Hollenbeck JE. An Avian Connection as a Catalyst to the 1918-1919 Influenza Pandemic. Int J Med Sci. 2005;2(2):87–90.

87. Pawar S, Chakrabarti A, Cherian S, Pande S, Nanaware M, Raut S, et al. An avian influenza A(H11N1) virus from a wild aquatic bird revealing a unique Eurasian-American genetic reassortment. Virus Genes. 2010;41(1):14–22.

88. Sarkar S, Khan SU, Mikolon A, Rahman MZ, Abedin J, Zeidner N, et al. An epidemiological study of avian influenza A (H5) virus in nomadic ducks and their raising practices in northeastern Bangladesh, 2011-2012. Influenza and other Respiratory Viruses. 2017;11(3):275–82.

89. Rahman MAW, Nidom CA, Budhi S. An exploratory study in the indonesian archipelago: Are there influenza b virus (b-victoria sub-type) in the bat’s respiratory organs? Indian Journal of Public Health Research and Development. 2019;10(9):1564–8.

90. Poetranto ED, Yamaoka M, Nastri AM, Krisna LAW, Rahman MH, Wulandari L, et al. An H5N1 highly pathogenic avian influenza virus isolated from a local tree sparrow in Indonesia. Microbiology and Immunology. 2011;55(9):666–72.

91. Poetri ON, Bouma A, Murtini S, Claassen I, Koch G, Soejoedono RD, et al. An inactivated H5N2 vaccine reduces transmission of highly pathogenic H5N1 avian influenza virus among native chickens. Vaccine. 2009;27(21):2864–9.

92. Chiu D. An informatics and epidemiological evaluation of infectious disease surveillance and reporting practices in Thailand: a case study of Suphanburi province and Avian Influenza. AMIA Annu Symp Proc. 2008;904.

93. Dandagi GL, Byahatti SM. An insight into the swine-influenza A (H1N1) virus infection in humans. Lung India. 2011;28(1):34–8.

94. Kaewpongsri S, Sukasem C, Srichunrusami C, Pasomsub E, Zwang J, Pairoj W, et al. An integrated bioinformatics approach to the characterization of influenza A/H5N1 viral sequences by microarray data: Implication for monitoring H5N1 emerging strains and designing appropriate influenza vaccines. Mol Cell Probes. 2010;24(6):387–95.

95. Wijayaratne WMDGB, Devasiri V, Nagahawatte A, Bodinayake CK, Gunasena S, Weerasinghe NP, et al. An outbreak of adenovirus causing severe respiratory illness in Southern Sri Lanka, 2018. Am J Trop Med Hyg. 2019;101(5):175.

96. Apidechkul T. An outbreak of Influenza A (H1N1) 2009 at Mae Fah Luang University, Chiang Rai Province, northern Thailand. Retrovirology. 2010;7:P187.

97. Tangkanakul W, Thawatsupha P, Lertmongkol J, Tharmaphornpilas P, Laolukpong P. An outbreak of influenza A virus in a Hilltribe Village of Mae Hong Son Province Thailand, 1997. Journal of the Medical Association of Thailand. 2000;83(9):1005–10.

98. Peter S, Balakrishnan A, Potdar VA, Chadha MS, Jadhav SM. An outbreak of influenza A(H3N2) in Alappuzha district, Kerala, India, in 2011. Journal of Infection in Developing Countries. 2015;9(4):362–7.

99. Amatya B, Pandey P, Shrestha SK. An outbreak of influenza among trekkers in the Everest region of Nepal. J Travel Med. 2021;27(6):1–2.

100. Khan UH, Ahmad F, Hussain Mir M, Koul PA, Mir MA, Bali NK, et al. An outbreak of influenza B in an isolated nomadic community in Jammu & Kashmir, India. Indian J Med Res. 2013;138:1012–5.

101. Biswas DK, Kaur P, Murhekar M, Bhunia R. An outbreak of pandemic influenza A (H1N1) in Kolkata, West Bengal, India, 2010. Indian J Med Res. 2012;135(4):529–33.

102. Tillekeratne LG, Bodinayake CK, Nagahawatte A, Vidanagama D, Devasiri V, Arachchi WK, et al. An under-recognized influenza epidemic identified by rapid influenza testing, Southern Sri Lanka, 2013. American Journal of Tropical Medicine and Hygiene. 2015;92(5):1023–9.

103. Su Y, Yang HY, Zhang BJ, Jia HL, Tien P. Analysis of a point mutation in H5N1 avian influenza virus hemagglutinin in relation to virus entry into live mammalian cells. Arch Virol. 2008;153(12):2253–61.

104. Putri K, Wibowo MH, Tarigan S, Wawegama N, Ignjatovic J, Noormohammadi AH. Analysis of antibody response to an epitope in the haemagglutinin subunit 2 of avian influenza virus H5N1 for differentiation of infected and vaccinated chickens. Avian Pathology. 2020;49(2):161–70.

105. Zhang J, Lei F. Analysis of human infectious avian influenza virus: hemagglutinin genetic characteristics in Asia and Africa from 2004 to 2009. Integr Zool. 2010;5(3):264–71.

106. Danishuddin, Khan AU. Analysis of PB2 protein from H9N2 and H5N1 avian flu virus. Bioinformation. 2008;3(1):41–6.

107. Vijayakumar P, Mishra A, Ranaware PB, Kolte AP, Kulkarni DD, Burt DW, et al. Analysis of the crow lung transcriptome in response to infection with highly pathogenic H5N1 avian influenza virus. Gene. 2015;559(1):77–85.

108. Kamal RP, Tosh C, Pattnaik B, Behera P, Nagarajan S, Gounalan S, et al. Analysis of the PB2 gene reveals that Indian H5N1 influenza virus belongs to a mixed-migratory bird sub-lineage possessing the amino acid lysine at position 627 of the PB2 protein. Arch Virol. 2007;152(9):1637–44.

109. Arbat S, Dave M, Niranjane V, Rahman I, Arbat A. Analyzing the clinical profile of swine flu/influenza A H1N1 infection in central India: a retrospective study. VirusDisease. 2017;28(1):33–8.

110. Giduthuri JG, Purohit V, Kudale A, Utzinger J, Schindler C, Weiss MG. Antenatal influenza vaccination in urban Pune, India: clinician and community stakeholders’ awareness, priorities, and practices. Human Vaccines and Immunotherapeutics. 2021;17(4):1211–22.

111. Paul M, Tavornpanich S, Abrial D, Gasqui P, Charras-Garrido M, Thanapongtharm W, et al. Anthropogenic factors and the risk of highly pathogenic avian influenza H5N1: Prospects from a spatial-based model. Veterinary Research [Internet]. 2010;41(3). Available from: https://www.scopus.com/inward/record.uri?eid=2-s2.0-77649115925&doi=10.1051%2fvetres%2f2009076&partnerID=40&md5=c503020207c86fd04b1ee5ee97ff9338

112. Enkhtaivan G, Maria John KM, Ayyanar M, Sekar T, Jin KJ, Kim DH. Anti-influenza (H1N1) potential of leaf and stem bark extracts of selected medicinal plants of South India. Saudi J Biol Sci. 2015;22(5):532–8.

113. Shoji M, Takahashi E, Hatakeyama D, Iwai Y, Morita Y, Shirayama R, et al. Anti-influenza activity of c60 fullerene derivatives. PLoS One. 2013;8(6):e66337.

114. Iwai Y, Murakami K, Gomi Y, Hashimoto T, Asakawa Y, Okuno Y, et al. Anti-influenza activity of marchantins, macrocyclic bisbibenzyls contained in liverworts. PLoS One. 2011;6(5):e19825.

115. Davey RT Jr, Fernández-Cruz E, Markowitz N, Pett S, Babiker AG, Wentworth D, et al. Anti-influenza hyperimmune intravenous immunoglobulin for adults with influenza A or B infection (FLU-IVIG): a double-blind, randomised, placebo-controlled trial. The Lancet Respiratory Medicine. 2019;7(11):951–63.

116. Shoji M, Woo SY, Masuda A, Win NN, Ngwe H, Takahashi E, et al. Anti-influenza virus activity of extracts from the stems of Jatropha multifida Linn.collected in Myanmar. BMC Complementary and Alternative Medicine [Internet]. 2017;17(1). Available from: https://www.scopus.com/inward/record.uri?eid=2-s2.0-85011805114&doi=10.1186%2fs12906-017-1612-8&partnerID=40&md5=e8f223f586522f6c11471527ea51b75d

117. Tillekeratne LG, Bodinayake CK, Dabrera T, Nagahawatte A, Arachchi WK, Sooriyaarachchi A, et al. Antibiotic overuse for acute respiratory tract infections in Sri Lanka: a qualitative study of outpatients and their physicians. BMC Family Practice [Internet]. 2017;18(1). Available from: https://www.scopus.com/inward/record.uri?eid=2-s2.0-85015254454&doi=10.1186%2fs12875-017-0619-z&partnerID=40&md5=060dea799ae4f0c8c41fdf58f9bcee63

118. Tandale BV, Pawar SD, Gurav YK, Parkhi SS, Mishra AC. Antibody persistence after Pandemic H1N1 2009 influenza vaccination among healthcare workers in Pune, India. Human Vaccines and Immunotherapeutics. 2013;9(1):125–7.

119. McLaws M, Priyono W, Bett B, Al-Qamar S, Claassen I, Widiastuti T, et al. Antibody response and risk factors for seropositivity in backyard poultry following mass vaccination against highly pathogenic avian influenza and Newcastle disease in Indonesia. Epidemiology and Infection. 2016;143(8):1632–42.

120. Chittaganpitch M, Puthavathana P, Praphasiri P, Waicharoen S, Shrestha M, Mott JA, et al. Antibody responses against influenza B lineages among community-dwelling individuals 65 years of age or older having received trivalent inactivated influenza vaccine during two consecutive seasons in Thailand. Southeast Asian Journal of Tropical Medicine and Public Health. 2019;50(3):500–13.

121. Nakphooki S, Patumanond J, Shresthai M, Prasert K, Chittaganpitch M, Praphasiri P, et al. Antibody responses induced by trivalent inactivated influenza vaccine among pregnant and non-pregnant women in Thailand: A matched cohort study. PLoS ONE. 2021;16(6):e0253028.

122. Swayne DE, Suarez DL, Spackman E, Jadhao S, Dauphin G, Kim-Torchetti M, et al. Antibody titer has positive predictive value for vaccine protection against challenge with natural antigenic-drift variants of H5N1 high-pathogenicity avian influenza viruses from Indonesia. Journal of Virology. 2015;89(7):3746–62.

123. Leroux-Roels I, Borkowski A, Vanwolleghem T, Dramé M, Clement F, Hons E, et al. Antigen sparing and cross-reactive immunity with an adjuvanted rH5N1 prototype pandemic influenza vaccine: a randomised controlled trial. Lancet. 2007;370(9587):580–9.

124. Islam MA riful, Sultana N, Ahmed F, Rahman MM, Rahman SR ezwana. ANTIGENIC AND GENETIC CHARACTERIZATION OF INFLUENZA B VIRUSES IN 2012 FROM SLUMS, DHAKA, BANGLADESH. Southeast Asian J Trop Med Public Health. 2015;46(4):611–5.

125. Shanmuganatham K, Feeroz MM, Jones-Engel L, Smith GJD, Fourment M, Walker D, et al. Antigenic and molecular characterization of avian influenza A(H9N2) viruses, Bangladesh. Emerging Infectious Diseases. 2013;19(9):1393–402.

126. Bhat S, Nagarajan S, Kumar M, Murugkar HV, Kalaiyarasu S, Venkatesh G, et al. Antigenic characterization of H5N1 highly pathogenic avian influenza viruses isolated from poultry in India, 2006-2015. Archives of Virology. 2017;162(2):487–94.

127. Setiawaty V, Pratiwi E, Pawestri HA, Ibrahim F, Soebandrio A. Antigenic variation in H5N1 clade 2.1 viruses in Indonesia From 2005 to 2011 submit a paper. Virology: Research and Treatment. 2013;4:27–34.

128. Koel BF, van der Vliet S, Burke DF, Bestebroer TM, Bharoto EE, Yasa IWW, et al. Antigenic variation of clade 2.1 H5N1 virus is determined by a few amino acid substitutions immediately adjacent to the receptor binding site. mBio [Internet]. 2014;5(3). Available from: https://www.scopus.com/inward/record.uri?eid=2-s2.0-84904015625&doi=10.1128%2fmBio.01070-14&partnerID=40&md5=603e8e7e2c066a333c5b2c369e9654d4

129. Nayak MK, Agrawal AS, Bose S, Naskar S, Bhowmick R, Chakrabarti S, et al. Antiviral activity of baicalin against influenza virus H1N1-pdm09 is due to modulation of NS1-mediated cellular innate immune responses. Journal of Antimicrobial Chemotherapy. 2014;69(5):1298–310.

130. Rajbhandari M, Mentel R, Jha PK, Chaudhary RP, Bhattarai S, Gewali MB, et al. Antiviral activity of some plants used in Nepalese traditional medicine. Evid Based Complement Alternat Med. 2009;6(4):517–22.

131. Potdar VA, Dakhave MR, Kulkarni PB, Tikhe SA, Patil KN, Kadam AA, et al. Antiviral drug profile of human influenza A & B viruses circulating in india: 2004-2011. Indian J Med Res. 2014;140:244–51.

132. Pongthanapisith V, Ikuta K, Puthavathana P, Leelamanit W. Antiviral protein of Momordica charantia L. inhibits different subtypes of influenza A. Evidence-based Complementary and Alternative Medicine [Internet]. 2013;2013. Available from: https://www.scopus.com/inward/record.uri?eid=2-s2.0-84880891334&doi=10.1155%2f2013%2f729081&partnerID=40&md5=d4c3c65f0b28e7835eee77c4bef9c103

133. Apisarnthanarak A, Mundy LM. Antiviral therapy for avian influenza virus (H5N1) infection at 2 Thai medical centers: Survey findings and implications for pandemic preparedness. Infection Control and Hospital Epidemiology. 2008;29(12):1185–8.

134. McCullers JA. Antiviral therapy of influenza. Expert Opin Investig Drugs. 2005;14(3):305–12.

135. Samaan G, Hendrawati F, Taylor T, Pitona T, Marmansari D, Rahman R, et al. Application of a healthy food markets guide to two indonesian markets to reduce transmission of “avian flu.” Bulletin of the World Health Organization. 2012;90(4):295–300.

136. Susilarini NK, Sitorus M, Praptaningsih CY, Sampurno OD, Bratasena A, Mulyadi E, et al. Application of WHO’s guideline for the selection of sentinel sites for hospital-based influenza surveillance in Indonesia. BMC Health Services Research [Internet]. 2014;14(1). Available from: https://www.scopus.com/inward/record.uri?eid=2-s2.0-84907910919&doi=10.1186%2f1472-6963-14-424&partnerID=40&md5=5f5cc9d611329152797af42c0abccc1f

137. Pandejpong D, Danchaivijitr S, Vanprapa N, Pandejpong T, Cook EF. Appropriate time-interval application of alcohol hand gel on reducing influenza-like illness among preschool children: A randomized, controlled trial. American Journal of Infection Control. 2012;40(6):507–11.

138. Hassan MM, Hoque MA, Debnath NC, Yamage M, Klaassen M. Are Poultry or Wild Birds the Main Reservoirs for Avian Influenza in Bangladesh? Ecohealth. 2017;14(3):490–500.

139. Tewawong N, Prachayangprecha S, Vichiwattana P, Korkong S, Klinfueng S, Vongpunsawad S, et al. Assessing Antigenic Drift of Seasonal Influenza A(H3N2) and A(H1N1)pdm09 Viruses. PLoS One. 2015;10(10):e0139958.

140. Netrabukkana P, Robertson ID, Kasemsuwan S, Wongsathapornchai K, Fenwick S. Assessing Potential Risks of Influenza A Virus Transmission at the Pig-Human Interface in Thai Small Pig Farms Using a Questionnaire Survey. Transboundary and Emerging Diseases. 2016;63(1):e135–9.

141. Dang A, Sharma J. Assessing the Low Influenza Vaccination Coverage Rate Among Healthcare Personnel in India: A Review of Obstacles, Beliefs, and Strategies. Value in Health Regional Issues. 2020;21:100–4.

142. Roche SE, Cogger N, Garner MG, Putra AAG, Toribio JALML. Assessing the risk of highly pathogenic avian influenza H5N1 transmission through poultry movements in Bali, Indonesia. Preventive Veterinary Medicine. 2014;113(4):599–607.

143. Tare DS, Kode SS, Hurt AC, Pawar SD. Assessing the susceptibility of highly pathogenic avian influenza H5N1 viruses to oseltamivir using embryonated chicken eggs. Indian J Med Res. 2019;150(5):486–91.

144. Carter MJ, Gurung P, Jones C, Rajkarnikar S, Kandasamy R, Gurung M, et al. Assessment of an Antibody-in-Lymphocyte Supernatant Assay for the Etiological Diagnosis of Pneumococcal Pneumonia in Children. Frontiers in Cellular and Infection Microbiology [Internet]. 2020;9. Available from: https://www.scopus.com/inward/record.uri?eid=2-s2.0-85078843141&doi=10.3389%2ffcimb.2019.00459&partnerID=40&md5=b9887665b919a44498ffde3ebaade4d3

145. Sayeed MA, Smallwood C, Imam T, Mahmud R, Hasan RB, Hasan M, et al. Assessment of hygienic conditions of live bird markets on avian influenza in Chittagong metro, Bangladesh. Preventive Veterinary Medicine. 2017;142:7–15.

146. Newman KL, Stewart LM, Scott EM, Tielsch JM, Englund JA, Khatry SK, et al. Assessment of indirect protection from maternal influenza immunization among non-vaccinated household family members in a randomized controlled trial in Sarlahi, Nepal. Vaccine. 2020;38(43):6826–31.

147. Kumari R, Gupta R, Langer B, Verma A. Assessment of knowledge and practices towards swine flu: A cross-sectional study among rural housewives. Indian Journal of Community Health. 2016;28(1):21–7.

148. Swayne DE, Pavade G, Hamilton K, Vallat B, Miyagishima K. Assessment of national strategies for control of high-pathogenicity avian influenza and low-pathogenicity notifiable avian influenza in poultry, with emphasis on vaccines and vaccination. Rev Sci Tech. 2011;30(3):839–70.

149. Sebastian J, Mathews RM, Gopal H, Jose A. Assessment of parents knowledge attitude and practice towards vaccines that are not covered under expanded program on immunization in India. Pharmacoepidemiol Drug Saf. 2020;29:448–9.

150. Shankar BP, Gowda RNS, Manjunath Prabhu BH, Pattnaik B, Nagarajan S, Patil SS, et al. Assessment of pathogenic potential of two indian h5n1 highly pathogenic avian influenza virus isolates by intravenous pathogenicity index test. International Journal of Poultry Science. 2009;8(3):283–90.

151. Kittikraisak W, Chittaganpitch M, Gregory CJ, Laosiritaworn Y, Thantithaveewat T, Dawood FS, et al. Assessment of potential public health impact of a quadrivalent inactivated influenza vaccine in Thailand. Influenza and other Respiratory Viruses. 2016;10(3):211–9.

152. Unsematham S, Ketbumrongporn W, Boonthongtho K. Associated factors for diagnosis of influenza A (H1N1) 2009 in patients presenting to Rajavithi Hospital. Journal of the Medical Association of Thailand = Chotmaihet thangphaet. 2012;95:S16-21.

153. Upadhyay AK, Srivastava S. Association between Haemophilus influenza type B (Hib) vaccination and child anthropometric outcomes in Andhra Pradesh (India): Evidence from the Young Lives Study. Journal of Public Health (Germany). 2017;25(6):581–9.

154. Hadakshi RK, Patel DM, Patel MV, Patel MM, Patel PJ, Patel MV, et al. Association between socioeconomic status and influenza-like illness: A study from Western part of India. J Family Med Prim Care. 2020;9(9):4587–91.

155. Chowdhury S, Azziz-Baumgartner E, Kile JC, Hoque MA, Rahman MZ, Hossain ME, et al. Association of biosecurity and hygiene practices with environmental contamination with influenza a viruses in live bird markets, bangladesh. Emerging Infectious Diseases. 2020;26(9):2087–96.

156. Choudhary ML, Alagarasu K, Chaudhary U, Kawale S, Malasane P, Gurav YK, et al. Association of single nucleotide polymorphisms in TNFA and IL10 genes with disease severity in influenza A/H1N1pdm09 virus infections: A study from Western India. Viral Immunology. 2018;31(10):683–8.

157. Dharmayanti NLPI, Thor SW, Zanders N, Hartawan R, Ratnawati A, Jang Y, et al. Attenuation of highly pathogenic avian influenza A(H5N1) viruses in Indonesia following the reassortment and acquisition of genes from low pathogenicity avian influenza A virus progenitors. Emerging Microbes and Infections [Internet]. 2018;7(1). Available from: https://www.scopus.com/inward/record.uri?eid=2-s2.0-85051957775&doi=10.1038%2fs41426-018-0147-5&partnerID=40&md5=e4180a065452f00cb04cb1aa3aa3c9df

158. Bala D. Attitudes, beliefs, and self-use of Kabasura Kudineer among urban and rural population in Tamil Nadu, India: A comparative cross-sectional study. J Family Med Prim Care. 2021;10(1):158–66.

159. Pongcharoensuk P, Adisasmito W, Sat LM, Silkavute P, Muchlisoh L, Cong Hoat P, et al. Avian and pandemic human influenza policy in South-East Asia: The interface between economic and public health imperatives. Health Policy and Planning. 2012;27(5):374–83.

160. Lahariya C, Sharma AK, Pradhan SK. Avian flu and possible human pandemic. Indian Pediatr. 2006;43(4):317–25.

161. Rimi NA, Sultana R, Ahmed KI, Hasin M, Rahman MdZ, Roza AK, et al. Avian flu: Piloting an intervention to reduce the risk of transmission to backyard poultry-raising families in rural Bangladesh. Am J Trop Med Hyg. 2010;83(5):103.

162. Taylor WRJ, Burhan E, Wertheim H, Soepandi PZ, Horby P, Fox A, et al. Avian influenza - A review for doctors in travel medicine. Travel Medicine and Infectious Disease. 2010;8(1):1–12.

163. Nagarajan S, Tosh C, Murugkar HV, Sridevi R, Kumar M, Katare M, et al. Avian influenza (H5N1) virus of clade 2.3.2 in domestic poultry in India. PLoS ONE. 2012;7(2):e31844.

164. Santhia K, Ramy A, Jayaningsih P, Samaan G, Putra AAG, Dibia N, et al. Avian influenza A H5N1 infections in Bali province, Indonesia: A behavioral, virological and seroepidemiological study. Influenza and other Respiratory Viruses. 2009;3(3):81–9.

165. Setiawaty V, Dharmayanti NLPI, Misriyah, Pawestri HA, Azhar M, Tallis G, et al. Avian Influenza A(H5N1) Virus Outbreak Investigation: Application of the FAO-OIE-WHO Four-way Linking Framework in Indonesia. Zoonoses and Public Health. 2015;62(5):381–7.

166. Koh GCH, Abikusno N, Kwing CS, Yee WT, Kusumaratna R, Sundram M, et al. Avian influenza and south Jakarta primary healthcare workers: A controlled mixed-method study. Tropical Medicine and International Health. 2009;14(7):817–29.

167. Cristalli A, Morini M, Comin A, Capello K, Sunn K, Martini M. Avian influenza epidemiology in semi-intensive free ranging duck flocks of the Moyingyi Wetland in Bago East District, Myanmar. Tropical Animal Health and Production. 2018;50(2):251–7.

168. Ruamsap N, Khantapura P, Gonwong S, Khemnu N, Chuenchitra T, Islam D, et al. Avian influenza exposure in young thai males from suphanburi province of thailand. Am J Trop Med Hyg. 2017;97(5):50–1.

169. Songserm T, Amonsin A, Jam-on R, Sae-Heng N, Meemak N, Pariyothorn N, et al. Avian influenza H5N1 in naturally infected domestic cat. Emerg Infect Dis. 2006;12(4):681–3.

170. Aditama TY, Kusriastuti R, Purba W, Misriyah, Santoso H, Bratasena A, et al. Avian influenza H5N1 transmission in households, Indonesia. PLoS ONE. 2012;7(1):e29971.

171. Pawar SD, Tandale BV, Raut CG, Parkhi SS, Barde TD, Gurav YK, et al. Avian influenza H9N2 seroprevalence among poultry workers in Pune, India, 2010. PLoS ONE. 2012;7(5):e36374.

172. van Reeth K. Avian influenza in swine: a threat for the human population? Verh K Acad Geneeskd Belg. 2006;68(2):81–101.

173. Suttie A, Karlsson EA, Deng YM, Hurt AC, Greenhill AR, Barr IG, et al. Avian influenza in the Greater Mekong Subregion, 2003-2018. Infect Genet Evol. 2019;74:103920.

174. Ahmed SSU, Barua H, Das A, Rahman MH, Ahad A, Faruque R, et al. Avian influenza outbreaks in chickens, Bangladesh. Emerg Infect Dis. 2008;14(12):1909–12.

175. Maton T, Fungladda W, Kaewkangwal J, Butraporn P. Avian influenza protection knowledge, awareness, and behaviors in a high-risk population in Suphan Buri Province, Thailand. Southeast Asian J Trop Med Public Health. 2007;38(3):560–8.

176. Gurley ES, Rahman MZ, Rahman M, Haider N, Chowdhury S, Zaman RU, et al. Avian influenza surveillance in domestic waterfowl and environment of live bird markets in Bangladesh, 2007-2012. Sci Rep. 2018;8(1):9396.

177. Pawar SD, Kale SD, Rawankar AS, Koratkar SS, Raut CG, Pande SA, et al. Avian influenza surveillance reveals presence of low pathogenic avian influenza viruses in poultry during 2009-2011 in the West Bengal State, India. Virology Journal [Internet]. 2012;9. Available from: https://www.scopus.com/inward/record.uri?eid=2-s2.0-84864771999&doi=10.1186%2f1743-422X-9-151&partnerID=40&md5=c101d8dcb42290fa5bc2287c336e5f69

178. Sultana R, Islam MS, Rahman M, Nahar K, Goswami D, Nahar S, et al. Avian influenza virus A (H5N1), detected through routine surveillance, in child, Bangladesh. Emerg Infect Dis. 2009;15(8):1311–3.

179. Alexander DJ. Avian influenza viruses and human health. Dev Biol (Basel). 2006;124:77–84.

180. Gilbert M, Xiao X, Chaitaweesub P, Kalpravidh W, Premashthira S, Boles S, et al. Avian influenza, domestic ducks and rice agriculture in Thailand. Agric Ecosyst Environ. 2007;119:409–15.

181. Trampuz A, Prabhu RM, Smith TF, Baddour LM. Avian influenza: a new pandemic threat? Mayo Clin Proc. 2004;79(4):523–30; quiz 530.

182. Steensels M, Van Borm S, Van den Berg TP. Avian influenza: mini-review, European control measures and current situation in Asia. Verh K Acad Geneeskd Belg. 2006;68(2):103–20.

183. Louie C. Avian influenza: Myth or mass murder? Can J Infect Dis Med Microbiol. 2005;16(3):197–201.

184. Lazarus R, Lim PL. Avian influenza: recent epidemiology, travel-related risk, and management. Curr Infect Dis Rep. 2015;17(1):456.

185. Adams S, Sandrock C. Avian influenza: update. Med Princ Pract. 2010;19(6):421–32.

186. Zeitlin GA, Maslow MJ. Avian Influenza. Curr Infect Dis Rep. 2005;7(3):193–9.

187. Gowthaman V, Singh SD, Dhama K, Barathidasan R, Anjaneya, Bhatt P. Avian pathogenic E. Coli (APEC) associated with respiratory disease complications in poultry. Veterinary Practitioner. 2013;14(2):430–1.

188. Subiakto Y. Aviation medicine capacity on facing biological threat in Indonesia airports. Infectious Disease Reports [Internet]. 2020;12. Available from: https://www.scopus.com/inward/record.uri?eid=2-s2.0-85090662685&doi=10.4081%2fidr.2020.8738&partnerID=40&md5=44be1234a93f3468b64c4ef0adce6ec2

189. Hadaye R, Manapurath R, Gadapani B. Awareness and acceptance of H1N1 vaccination among physicians: Experience of 2017 vaccination campaign. Journal of Education and Health Promotion [Internet]. 2019;8(1). Available from: https://www.scopus.com/inward/record.uri?eid=2-s2.0-85085558386&doi=10.4103%2fjehp.jehp_356_18&partnerID=40&md5=7052e9249cb758f7e4c0add4dd0dedf7

190. James PT, Kunoor A, Rakesh PS. Awareness of health care workers, patients and visitors regarding air borne infection control – A descriptive study from a Tertiary Care Centre in Kerala, southern India. Indian Journal of Tuberculosis. 2018;65(2):168–71.

191. Zareen U, Surya Durga Prasad M. Awareness of swine flu (Influenza h1n1) among the rural population of shamirpet mandal,telangana. Indian Journal of Public Health Research and Development. 2018;9(10):80–4.

192. Manandhar K, Chataut J, Khanal K, Shrestha A, Shrestha S, Shrestha S. Awareness regarding preventive measures of avian influenza among the adult people of Thimi Municipality, Nepal. Kathmandu University Medical Journal. 2013;11(41):45–9.

193. Chaudhary V, Singh RK, Agrawal VK, Agarwal A, Kumar R, Sharma M. Awareness, perception and myths towards swine flu in school children of Bareilly, Uttar Pradesh. Indian J Public Health. 2010;54(3):161–4.

194. Talwar S, Sood S, Kumar J, Chauhan R, Sharma M, Tuli HS. Ayurveda and Allopathic Therapeutic Strategies in Coronavirus Pandemic Treatment 2020. Curr Pharmacol Rep. 2020;1–10.

195. Gundeti MS, Bhurke LW, Mundada PS, Murudkar S, Surve A, Sharma R, et al. AYUSH 64, a polyherbal Ayurvedic formulation in Influenza-like illness - Results of a pilot study. Journal of Ayurveda and Integrative Medicine [Internet]. 2021; Available from: https://www.scopus.com/inward/record.uri?eid=2-s2.0-85089968195&doi=10.1016%2fj.jaim.2020.05.010&partnerID=40&md5=2ec7c8a5724654586353ff097d5d376b

196. Sultana R, Nahar N, Rimi NA, Azad S, Islam MS, Gurley ES, et al. Backyard poultry raising in Bangladesh: A valued resource for the villagers and a setting for zoonotic transmission of avian influenza. A qualitative study. Rural and Remote Health [Internet]. 2012;12(3). Available from: https://www.scopus.com/inward/record.uri?eid=2-s2.0-84871704614&partnerID=40&md5=d3c454eb479ef4c398e0eb5389c3e098

197. Biswal B, Dwibedi B, Hansa J, Kar SK. Bacterial and viral pathogen spectra of ARI among the children below 5 years age group in tribal and coastal regions of Odisha. Indian Journal of Public Health Research and Development. 2018;9(1):366–72.

198. Kongre VA, Pol SS, Bharadwaj RS, Gurav YK, Chadha MS, Tandale BV, et al. Bacteriological Study Among Influenza-like Illness Cases in a Community Setting in Pune, India. Cureus. 2018;10(11):e3601.

199. Sultana R, Rimi NA, Azad S, Saiful Islam M, Salah Uddin Khan M, Gurley ES, et al. Bangladeshi backyard poultry raisers’ perceptions and practices related to zoonotic transmission of avian influenza. Journal of Infection in Developing Countries. 2012;6(2):156–65.

200. Safira L, Siregar AS. Baseline KAP survey: Avian influenza in the incidence areas of Tangerang, banten province, Indonesia. Am J Trop Med Hyg. 2009;81(5):289.

201. Jamieson DJ, Kissin DM, Bridges CB, Rasmussen SA. Benefits of influenza vaccination during pregnancy for pregnant women. Am J Obstet Gynecol. 2012;207(3):S17-20.

202. Lemoine CH, Nidom RV, Ventura R, Indrasari S, Normalina I, Santoso KP, et al. Better pandemic influenza preparedness through adjuvant technology transfer: Challenges and lessons learned. Vaccines [Internet]. 2021;9(5). Available from: https://www.scopus.com/inward/record.uri?eid=2-s2.0-85105935254&doi=10.3390%2fvaccines9050461&partnerID=40&md5=480ea01877f19c8fd119b30642001c8a

203. Roshier DA, Heinsohn R, Adcock GJ, Beerli P, Joseph L. Biogeographic models of gene flow in two waterfowl of the Australo-Papuan tropics. Ecol Evol. 2012;2(11):2803–14.

204. Khanna M, Akther N, Srivastava V, Kumar P, Vijayan VK. Biological and epidemiological aspects of influenza virus H5N1 in context of India. Indian J Exp Biol. 2006;44(4):265–78.

205. Noisumdaeng P, Pooruk P, Kongchanagul A, Assanasen S, Kitphati R, Auewarakul P, et al. Biological properties of H5 hemagglutinin expressed by vaccinia virus vector and its immunological reactivity with human sera. Viral Immunology. 2013;26(1):49–59.

206. Biswas PK, Giasuddin M, Nath BK, Islam MZ, Debnath NC, Yamage M. Biosecurity and Circulation of Influenza A (H5N1) Virus in Live-Bird Markets in Bangladesh, 2012. Transboundary and Emerging Diseases. 2017;64(3):883–91.

207. Rimi NA, Sultana R, Muhsina M, Uddin B, Nahar N, Haider N, et al. Biosecurity Conditions in Small Commercial Chicken Farms, Bangladesh 2011-2012. Ecohealth. 2017;14(2):244–58.

208. Flowra MT, Asaduzzaman M. Biosecurity practices in backyard poultry in rural Bangladesh: A major contributing factor to the incursion of novel subtype of avian influenza and its human spill over in the community. Am J Trop Med Hyg. 2018;99(4):182.

209. Tenzin T, Wangdi C, Rai PB. Biosecurity survey in relation to the risk of HPAI outbreaks in backyard poultry holdings in Thimphu city area, Bhutan. BMC Veterinary Research [Internet]. 2017;13(1). Available from: https://www.scopus.com/inward/record.uri?eid=2-s2.0-85018518936&doi=10.1186%2fs12917-017-1033-4&partnerID=40&md5=15e9c0097b258f56676ccf02dc5b4465

210. Sellwood C, Asgari-Jirhandeh N, Salimee S. Bird flu: if or when? Planning for the next pandemic. Postgrad Med J. 2007;83(981):445–50.

211. Bridge ES, Kelly JF, Xiao X, Takekawa JY, Hill NJ, Yamage M, et al. Bird Migration and Avian Influenza: A Comparison of Hydrogen Stable Isotopes and Satellite Tracking Methods. Ecol Indic. 2014;45:266–73.

212. Wagh K, Bhatia A, Greenbaum BD, Bhanot G. Bird to human transmission biases and vaccine escape mutants in H5N1 infections. PLoS One. 2014;9(7):e100754.

213. Rashid H, Khandaker G, Muhit MA, Booy R. Bridging the 10/90 gap: Can Bangladesh provide a developing world model for influenza and pneumonia research? Journal of Pediatric Infectious Diseases. 2012;7(2):49–53.

214. Kitikoon P, Sreta D, Nuntawan Na Ayudhya S, Wongphatcharachai M, Lapkuntod J, Prakairungnamthip D, et al. Brief report: Molecular characterization of a novel reassorted pandemic H1N1 2009 in Thai pigs. Virus Genes. 2011;43(1):1–5.

215. Rasul CH, Bakar MA, Mamun AA, Siraz MS, Zaman RU. Burden and outcome of human influenza in a tertiary care hospital of Bangladesh. Asian Pacific Journal of Tropical Medicine. 2011;4(6):478–81.

216. Rasul CH, Siraz MS, Bakar MA, Mamun AA, Zaman RU. Burden and outcome of human influenza in a tertiary care hospital of Bangladesh. Asian Pac J Trop Med. 2011;4(6):478–81.

217. Shapiro D, Bodinayake CK, Nagahawatte A, Devasiri V, Kurukulasooriya R, Hsiang J, et al. Burden and seasonality of viral acute respiratory tract infections among outpatients in Southern Sri Lanka. American Journal of Tropical Medicine and Hygiene. 2017;97(1):88–96.

218. Reechaipichitkul W, Thavornpitak Y, Sutra S. Burden of adult Pneumonia in Thailand: A nationwide hospital admission data 2010. Journal of the Medical Association of Thailand. 2014;97(3):283–92.

219. Chatterjee P, Seth B, Biswas T, Bera K. Burden of H1N1 influenza in India (2010-2017): Identifying hotspots and policy directions. Am J Respir Crit Care Med [Internet]. 2018;197. Available from: http://www.atsjournals.org/doi/abs/10.1164/ajrccm-conference.2018.197.1_MeetingAbstracts.A4948

220. Narayan VV, Iuliano AD, Roguski K, Bhardwaj R, Chadha M, Saha S, et al. Burden of influenza-associated respiratory and circulatory mortality in India, 2010-2013. Journal of Global Health [Internet]. 2020;10(1). Available from: https://www.scopus.com/inward/record.uri?eid=2-s2.0-85084328340&doi=10.7189%2fjogh.10.010402&partnerID=40&md5=d38d33bd87df781e1517e73b2c784233

221. Mazumdar J, Chawla-Sarkar M, Rajendran K, Ganguly A, Sarkar UK, Ghosh S, et al. Burden of respiratory tract infections among paediatric in and out-patient units during 2010-11. European Review for Medical and Pharmacological Sciences. 2013;17(6):802–8.

222. Chadha MS, Mishra AC, Hirve S, Lele P, Deoshatwar A, Sambhudas S, et al. Burden of Seasonal and Pandemic Influenza-Associated Hospitalization during and after 2009 A(H1N1)pdm09 Pandemic in a Rural Community in India. PLoS ONE. 2013;8(5):e55918.

223. Putthasri W, Lertiendumrong J, Chompook P, Tangcharoensathien V, Coker R. Capacity of Thailand to contain an emerging influenza pandemic. Emerging Infectious Diseases. 2009;15(3):423–32.

224. Boonarkart C, Champunot R, Uiprasertkul M, Bunthi C, Kiatboobsri S, Rochanawutanon M, et al. Case report: Increased viral receptor expression associated with high viral load and severe pneumonia in a young patient infected with 2009 H1N1 influenza A with no pre-existing conditions. Journal of Medical Virology. 2012;84(3):380–5.

225. Puvanalingam A, Rajendiran C, Sivasubramanian K, Ragunanthanan S, Suresh S, Gopalakrishnan S. Case series study of the clinical profile of H1N1 swine flu influenza. Journal of Association of Physicians of India. 2011;59(1):14–8.

226. Pratheepamornkull T, Ratanakorn W, Samransamruajkit R, Poovorawan Y. CAUSATIVE AGENTS OF SEVERE COMMUNITY ACQUIRED VIRAL PNEUMONIA AMONG CHILDREN IN EASTERN THAILAND. Southeast Asian J Trop Med Public Health. 2015;46(4):650–6.

227. Suttinont C, Losuwanaluk K, Niwatayakul K, Hoontrakul S, Intaranongpai W, Silpasakorn S, et al. Causes of acute, undifferentiated, febrile illness in rural Thailand: Results of a prospective observational study. Ann Trop Med Parasitol. 2006;100(4):363–70.

228. Haider M, Ahamed SN, Leslie T. Challenges for Bangladesh to conquer avian influenza. International Journal of Pharmaceutical and Healthcare Marketing. 2008;2(4):273–83.

229. Kumar R, Amarchand R, Narayan VV, Saha S, Lafond KE, Kapoor SK, et al. Challenges in conducting a community-based influenza vaccine trial in a rural community in northern India. Human Vaccines and Immunotherapeutics. 2018;14(8):1909–13.

230. Stockman LJ, Anderson LJ, Brooks WA, Streatfield PK, Rahman M, Goswami D, et al. Challenges to Evaluating Respiratory Syncytial Virus Mortality in Bangladesh, 2004-2008. PLoS ONE. 2013;8(1):e53857.

231. Thepmalee C, Sanguansermsri P, Suwanankhon N, Chamnanpood C, Chamnanpood P, Pongcharoen S, et al. Changes in the NS1 gene of avian influenza viruses isolated in Thailand affect expression of type i interferon in primary chicken embryonic fibroblast cells. Indian Journal of Virology. 2013;24(3):365–72.

232. Alfelali M, Barasheed O, Tashani M, Azeem MI, El Bashir H, Memish ZA, et al. Changes in the prevalence of influenza-like illness and influenza vaccine uptake among Hajj pilgrims: A 10-year retrospective analysis of data. Vaccine. 2015;33(22):2562–9.

233. Rai SK. Changing trend of infectious diseases in Nepal. Adv Exp Med Biol. 2018;1052:19–38.

234. Sak I, Dewi L. Characteristics of children hospitalized with severe acute respiratory infection in west Nusa Tenggara Hospital, Mataram, Indonesia. Paediatr Respir Rev. 2012;13:S74.

235. Chudasama RK, Patel UV, Verma PB, Amin CD, Shah HM, Banerjee A, et al. Characteristics of fatal cases of pandemic influenza A (H1N1) from September 2009 to January 2010 in Saurashtra Region, India. Online Journal of Health and Allied Sciences [Internet]. 2010;9(4). Available from: https://www.scopus.com/inward/record.uri?eid=2-s2.0-79956352581&partnerID=40&md5=6b3f34a415fc208fe931ea4ce282e727

236. Chudasama RK, Patel UV, Verma PB. Characteristics of Hospitalized Patients with Severe and Non-Severe Pandemic Influenza A (H1N1) in Saurashtra Region, India (Two Waves Analysis). J Family Med Prim Care. 2013;2(2):182–7.

237. Suksatu A, Sangsawad W, Thitithanyanont A, Smittipat N, Fukuda MM, Ubol S. Characteristics of stork feces-derived H5N1 viruses that are preferentially transmitted to primary human airway epithelial cells. Microbiology and Immunology. 2009;53(12):675–84.

238. Chuaychoo B, Rattanasaengloet K, Banlengchit R, Horthongkham N, Athipanyasilp N, Totanarungroj K, et al. Characteristics, complications and mortality of respiratory syncytial virus compared to influenza infections in hospitalized adult patients in Thailand. Int J Infect Dis. 2021;

239. Fally M, Redlberger-Fritz M, Starzengruber P, Swoboda P, Fuehrer H, Yunus E, et al. Characterization and epidemiology of influenza viruses in patients seeking treatment for influenza-like illnesses in rural Bangladesh. Journal of Postgraduate Medicine. 2012;58(4):242–5.

240. Bhatia V, Gupta AK, Nainawatee HS. Characterization of equine influenza A/Equi-2/Ludhiana/87 (H3 N8) isolate by nucleotide sequencing of its neuraminidase gene. Indian Journal of Biotechnology. 2003;2(2):203–13.

241. Nakamura K, Shirakura M, Fujisaki S, Kishida N, Burke DF, Smith DJ, et al. Characterization of influenza A(H1N1)pdm09 viruses isolated from Nepalese and Indian outbreak patients in early 2015. Influenza and other Respiratory Viruses. 2017;11(5):399–403.

242. Roy S, Dahake R, Patil D, Tawde S, Mukherjee S, Athlekar S, et al. Characterization of influenza virus among influenza like illness cases in Mumbai, India. VirusDisease. 2014;25(3):372–5.

243. Ravindra A, Sharmila FM, Dhodapkar R, Sistla S, Wyawahare M. Characterization of mutations of Haemagglutinin (HA) and Neuraminidase (NA) genes of Influenza A H1N1pdm09 isolated during the 2018 outbreak in India. Int J Infect Dis. 2020;101:493.

244. Wiyatno A, Dewantari AK, Myint KS, Safari D, Zul Febrianti ES, Idris NS. Characterization of rhinovirus c from a 4-year-old boy with acute onset dilated cardiomyopathy in Jakarta, Indonesia. JMM Case Rep. 2018;5(9):1–4.

245. Upadhyay BP, Ghimire P, Tashiro M, Banjara MR. Characterization of seasonal influenza virus type and subtypes isolated from influenza like illness cases of 2012. Kathmandu University Medical Journal. 2017;15(57):56–60.

246. Ray K, Potdar VA, Cherian SS, Pawar SD, Jadhav SM, Waregaonkar SR, et al. Characterization of the complete genome of influenza a (H5N1) virus isolated during the 2006 outbreak in poultry in India. Virus Genes. 2008;36(2):345–53.

247. Keawcharoen J, Amonsin A, Oraveerakul K, Wattanodorn S, Papravasit T, Karnda S, et al. Characterization of the hemagglutinin and neuraminidase genes of recent influenza virus isolates from different avian species in Thailand. Acta Virol. 2005;49(4):277–80.

248. Chakrabarti AK, Pawar SD, Cherian SS, Koratkar SS, Jadhav SM, Pal B, et al. Characterization of the influenza A H5N1 viruses of the 2008-09 outbreaks in India reveals a third introduction and possible endemicity. PLoS ONE. 2009;4(11):e7846.

249. Suntronwong N, Klinfueng S, Korkong S, Vichaiwattana P, Thongmee T, Vongpunsawad S, et al. Characterizing genetic and antigenic divergence from vaccine strain of influenza A and B viruses circulating in Thailand, 2017-2020. Sci Rep. 2021;11(1):735.

250. Kandun IN, Samaan G, Harun S, Purba WH, Sariwati E, Septiawati C, et al. Chicken faeces garden fertilizer: Possible source of human avian influenza H5N1 infection. Zoonoses and Public Health. 2010;57(4):285–90.

251. Jain A, Dangee T, Jain B, Singh KP, Singh A, Dwivedi M, et al. Circulating genotypes of influenza virus in india and their correlation with clinical features and metereological factors. FASEB J [Internet]. 2012;26. Available from: http://www.fasebj.org/cgi/content/meeting_abstract/26/1_MeetingAbstracts/lb501?sid=2b657849-fcd6-4619-8175-950558ef69ee

252. Suntronwong N, Vichaiwattana P, Klinfueng S, Korkong S, Thongmee T, Vongpunsawad S, et al. Climate factors influence seasonal influenza activity in Bangkok, Thailand. PLoS ONE. 2020;15(9):e0239729.

253. Chudasama RK, Patel UV, Verma PB, Agarwal P, Bhalodiya S, Dholakiya D. Clinical and epidemiological characteristics of 2009 pandemic influenza A in hospitalized pediatric patients of the Saurashtra region, India. World Journal of Pediatrics. 2012;8(4):321–7.

254. Suntarattiwong P, Sojisirikul K, Sitaposa P, Pornpatanangkoon A, Chittaganpitch M, Srijuntongsiri S, et al. Clinical and epidemiological characteristics of respiratory syncytial virus and influenza virus associated hospitalization in urban Thai infants. Journal of the Medical Association of Thailand = Chotmaihet thangphaet. 2011;94:S164-171.

255. Agus R, Sulastri S, Murniati D, Darwis F, Wiweka IS, Rusli A, et al. Clinical and epidemiological features of patients with confirmed avian influenza presenting to Sulianti Saroso Infectious Diseases Hospital, Indonesia, 2005-2007. Ann Acad Med Singapore. 2008;37(6):454–7.

256. Anderson KB, Simasathien S, Watanaveeradej V, Weg AL, Ellison DW, Suwanpakdee D, et al. Clinical and laboratory predictors of influenza infection among individuals with influenza-like illness presenting to an urban Thai hospital over a five-year period. PLoS One. 2018;13(3):e0193050.

257. Punpanich W, Chirapanyanon P, Srisarang S. Clinical characteristics and hospital charges among thai children hospitalized with influenza. Southeast Asian Journal of Tropical Medicine and Public Health. 2014;45(1):75–84.

258. Sitlaothaworn C, Anugulruengkitt S, Kowitdamrong E, Panchareon C. Clinical characteristics and outcomes of influenza in hospitalized pediatric patients in king chulalongkorn memorial hospital. Journal of the Medical Association of Thailand. 2020;103(11):1220–9.

259. Saxena S, Singh D, Zia A, Umrao J, Srivastava N, Pandey A, et al. Clinical characterization of influenza A and human respiratory syncytial virus among patients with influenza like illness. Journal of Medical Virology. 2017;89(1):49–54.

260. Soepandi PZ, Burhan E, Mangunnegoro H, Nawas A, Aditama TY, Partakusuma L, et al. Clinical course of avian influenza A(H5N1) in patients at the Persahabatan Hospital, Jakarta, Indonesia, 2005-2008. Chest. 2010;138(3):665–73.

261. Aung K, Hattasingh W, Limkittikul K, Suntarattiwong P, Chotpitayasunondh T. Clinical difference between hospitalized paediatric pandemic H1N1 2009 influenza and other acute respiratory tract infections at queen sirikit national institute of child health, Thailand. Acta Paediatr Int J Paediatr. 2010;99:43.

262. Purakayastha DR, Broor S, Krishnan A, Gupta V, Sullender W, Fowler K, et al. Clinical differences between influenza A (H1N1) pdm09 & influenza B infections identified through active community surveillance in north India. Indian J Med Res. 2013;138:962–8.

263. Phungoen P, Sawanyawisuth K, Engchanil C, Sutra S, Rangsrikajee D, Lulitanond V, et al. Clinical factors predictive of PCR positive in pandemic H1N1 2009 influenza virus infection. Influenza and other Respiratory Viruses. 2011;5(6):e558–62.

264. Kanchana S, Kanchana S, Vijitsopa T, Thammakumpee K, Yamwong S, Sawanyawisuth K. Clinical factors predictive of pneumonia caused by pandemic 2009 H1N1 influenza virus. American Journal of Tropical Medicine and Hygiene. 2013;88(3):461–3.

265. Wulandari L, Palilingan JF, Setiawati L, Setyoningrum RA, Sarjono LA, Retnowati E, et al. Clinical features of Influenza a (H1N1) patients hospitalized in Dr Soetomo Hospital, Surabaya, Indonesia, during the peak of the 2009 flu pandemic. Chest [Internet]. 2010;138(4). Available from: http://chestjournal.chestpubs.org/cgi/content/meeting_abstract/138/4_MeetingAbstracts/602A?sid=562e108c-9e3f-4568-aec7-bc50b4337c63

266. Kosasih H, Karyana M, Lokida D, Alisjahbana B, Tjitra E, Gasem MH, et al. Clinical manifestations, hematology, and chemistry profiles of the six most common etiologies from an observational study of acute febrile illness in Indonesia. Open Forum Infect Dis. 2017;4:S127.

267. Patel KK, Patel AK, Mehta PM, Amin RP, Patel KP, Chuhan PC, et al. Clinical outcome of novel H1N1 (Swine Flu)-infected patients during 2009 pandemic at tertiary referral hospital in western India. Journal of Global Infectious Diseases. 2013;5(3):93–7.

268. Acharya U, Acharya SP. Clinical Outcome of Patients Hospitalized in a Tertiary Care Hospital of Nepal with Confirmed Influenza A/pdm 09(H1N1) in 2018/2019. Journal of Nepal Health Research Council. 2020;18(2):196–200.

269. Suntarattiwong P, Jarman RG, Levy J, Baggett HC, Gibbons RV, Chotpitayasunondh T, et al. Clinical performance of a rapid influenza test and comparison of nasal versus throat swabs to detect 2009 pandemic influenza a (h1n1) infection in thai children. Pediatric Infectious Disease Journal. 2010;29(4):366–7.

270. Dhar R, Ghoshal AG, Guleria R, Sharma S, Kulkarni T, Swarnakar R, et al. Clinical practice guidelines 2019: Indian consensus-based recommendations on influenza vaccination in adults. Lung India. 2020;37(7):S4–18.

271. Mital G, Vegad MM, Soni ST, Patel KJ, Khyati P. Clinical predictors of associated with influenza A (H1N1) during epidemic 2015 at tertiary care hospital, Ahmedabad. Indian Journal of Public Health Research and Development. 2017;8(1):80–5.

272. Midha T, Nath B, Kumari R, Rao YK, Lekhwani S, Vaswani ND, et al. Clinical predictors of influenza a(H1N1) in Kanpur, India. Journal of the Indian Medical Association. 2012;110(1):22–4.

273. Chu HY, Katz J, Tielsch J, Khatry SK, Shrestha L, LeClerq SC, et al. Clinical Presentation and Birth Outcomes Associated with Respiratory Syncytial Virus Infection in Pregnancy. PLoS One. 2016;11(3):e0152015.

274. Lochindarat S, Bunnag T. Clinical presentations of pandemic 2009 influenza A (H1N1) virus infection in hospitalized Thai children. Journal of the Medical Association of Thailand = Chotmaihet thangphaet. 2011;94:S107-112.

275. Singhal S, Sarda N, Arora R, Punia N, Jain A. Clinical profile & outcome of H1N1 infected pregnant women in a tertiary care teaching hospital of northern India. Indian J Med Res. 2014;139:454–8.

276. Mathur S, Dubey T, Kulshrestha M, Agarwal H, Mathur G, Mathur A, et al. Clinical profile and mortality among novel influenza A (H1N1) infected patients: 2009-2010 Jodhpur, Rajasthan pandemic. Journal of Association of Physicians of India. 2013;61(9):627–32.

277. Verma N, Pooniya V, Kumar A. Clinical Profile and Outcome of Influenza A/H1N1 in Pediatric Oncology Patients during the 2015 Outbreak: A Single Center Experience from Northern India. Journal of Pediatric Hematology/Oncology. 2017;39(7):e357–8.

278. Amaravathi KS, Sakuntala P, Sudarsi B, Manohar S, Nagamani R, Rao SR. Clinical profile and outcome of recent outbreak of influenza A H1N1 (swine flu) at a tertiary care center in Hyderabad, Telangana. Annals of Tropical Medicine and Public Health. 2015;8(6):267–71.

279. Kumari S, Suri V, Rao HR, Bhalla A, Singh I, Singh MP, et al. Clinical profile and outcome of scrub typhus-related acute respiratory distress syndrome in adults presenting to a tertiary care Hospital in North India. Open Forum Infect Dis. 2019;6:S616–7.

280. Das RR, Sami A, Lodha R, Jain R, Broor S, Kaushik S, et al. Clinical profile and outcome of swine flu in Indian children. Indian Pediatrics. 2011;48(5):373–8.

281. Gunasekaran K, Das S, Iyyadurai R. Clinical profile and outcomes of inpatients with influenza from a tertiary care center in South India. Trans R Soc Trop Med Hyg. 2019;113:S223–4.

282. Ramakrishna K, Sampath S, Chacko J, Chacko B, Narahari DL, Veerendra HH, et al. Clinical profile and predictors of mortality of severe pandemic (H1N1) 2009 virus infection needing intensive care: A multi-centre prospective study from South India. Journal of Global Infectious Diseases. 2012;4(3):145–52.

283. Singh J, Singh Sudan D, Singh S, Kaur Haher R, Kang M. Clinical profile of H1N1 like outbreak at Bathinda, Punjab, India. Am J Respir Crit Care Med [Internet]. 2014;189. Available from: http://www.atsjournals.org/doi/pdf/10.1164/ajrccm-conference.2014.189.1_MeetingAbstracts.A6244

284. Ratnaparkhe V, Ratnaparkhe R. Clinical profile of patients admitted with swine-origin influenza A (H1N1) virus infection: An experience from a tertiary care hospital. Journal of Communicable Diseases. 2018;50(1):4–8.

285. Saroch A, Ramadoss R, Kumar S, Mathews S, Taneja RS, Ali M, et al. Clinical Profile of Suspected H1N1 Influenza Patients and Predictor of Mortality in H1N1-Positive Patients. Infectious Diseases in Clinical Practice. 2018;26(1):35–8.

286. Sriram P, Kumar M, Renitha R, Mondal N, Bhat VB. Clinical profile of swine flu in children at Puducherry. Indian Journal of Pediatrics. 2010;77(10):1093–5.

287. Aggarwal KK, Pareek KK, Nadkar M, Tiwaskar M, Vora A. Clinical recommendations on the management of seasonal & acute febrile infections. Journal of the Indian Medical Association. 2020;118(3):13–9.

288. Siddharth V, Goyal V, Koushal V. Clinical-epidemiological profile of influenza a h1n1 cases at a tertiary care institute of India. Indian Journal of Community Medicine. 2012;37(4):232–5.

289. Siddharth V, Goyal V, Koushal VK, Gupta SK. Clinical-epidemiological profile of laboratory confirmed cases of influenza a H1N1, at Government Medical College and Hospital (GMCH), Chandigarh, India. J Int Med Sci Acad. 2012;25(4):285–6.

290. Prawira Y, Murniati D, Rusli A, Giriputro S, Setiawaty V, Oswari H, et al. Clinical, laboratory, and radiologic characteristics of confirmed avian influenza (H5N1). Southeast Asian Journal of Tropical Medicine and Public Health. 2012;43(4):877–89.

291. Tjitra E, Made Susila I, Lingga IMGD, Arief M, Pelupessy NM, Parwati I, et al. Clinical, serological and molecular diagnosis of typhoid fever, a significant cause of acute febrile illness among hospitalized patients in Indonesia from 2013-2016. Am J Trop Med Hyg. 2017;97(5):148.

292. Kadam D, Raichur PA, Chandanwale A, Joshi S, Marbaniang I, Mave V, et al. Clinical, social, and meteorological factors associated with dengue and malaria diagnosis in adults in Pune, India. Int J Infect Dis. 2016;45:240–1.

293. Chudasama RK, Patel UV, Verma PB, Amin CD, Savaria D, Ninama R, et al. Clinico-epidemiological features of the hospitalized patients with 2009 pandemic influenza A (H1N1) virus infection in Saurashtra region, India (September, 2009 to February, 2010). Lung India. 2011;28(1):11–5.

294. Mehta VK, Sharma P, Guleria RC, Ganju SA, Singh D, Kanga A. Clinico-epidemiological profile, pandemic influenza a H1N1/2009 and seasonal influenza, August 2009-March 2013, Himachal Pradesh, India. Indian Journal of Community Medicine. 2016;41(1):69–71.

295. Maheshwari M, Maheshwari S. Clinico-radiological profile and outcome of novel H1N1-infected patients during 2009 to 2014 pandemic at tertiary referral hospital in Rajasthan. Journal of Association of Physicians of India. 2015;63:42–5.

296. Borse RT, Kadam DB, Sangle SA, Basavraj A, Prasad HB, Umarji PB, et al. Clinicoradiologic correlation in adult patients diagnosed with novel influenza A (H1N1). Journal of Association of Physicians of India. 2013;61(9):600–7.

297. Charoenkul K, Nasamran C, Amonsin A, Lestari, Wibawa H, Lubis EP, et al. Co-circulation and characterization of HPAI-H5N1 and LPAI-H9N2 recovered from a duck farm, Yogyakarta, Indonesia. Transboundary Emer Dis. 2020;67(2):994–1007.

298. Poen MJ, Venkatesh D, Bestebroer TM, Vuong O, Scheuer RD, Oude Munnink BB, et al. Co-circulation of genetically distinct highly pathogenic avian influenza A clade 2.3.4.4 (H5N6) viruses in wild waterfowl and poultry in Europe and East Asia, 2017-18. Virus Evol. 2019;5(1):vez004.

299. Abe H, Mine J, Parchariyanon S, Takemae N, Boonpornprasert P, Ubonyaem N, et al. Co-infection of influenza A viruses of swine contributes to effective shuffling of gene segments in a naturally reared pig. Virology. 2015;484:203–12.

300. Beer M, Harder T, Parvin R, Begum JA, Chowdhury EH, Islam MR. Co-subsistence of avian influenza virus subtypes of low and high pathogenicity in Bangladesh: Challenges for diagnosis, risk assessment and control. Sci Rep. 2019;9(1):8306.

301. Auewarakul P, Chatsurachai S, Kongchanagul A, Kanrai P, Upala S, Suriyaphol P, et al. Codon volatility of hemagglutinin genes of H5N1 avian influenza viruses from different clades. Virus Genes. 2009;38(3):404–7.

302. Jhuria L, Muthu V, Gupta S, Singh MP, Biswal M, Goyal K, et al. Coinfection of H1N1 influenza and scrub typhus-a review. QJM. 2020;113(7):465–8.

303. Paul MC, Figuié M, Kovitvadhi A, Valeix S, Wongnarkpet S, Poolkhet C, et al. Collective resistance to HPAI H5N1 surveillance in the Thai cockfighting community: Insights from a social anthropology study. Preventive Veterinary Medicine. 2015;120(1):106–14.

304. Chandramoulie S, Ramraj B, Marimuthu A. Combating pandemics in india - a comparison study between the 1918, 2009 and 2019-2020 pandemics. Int J Res Pharm Sci. 2020;11:1762–7.

305. Omer SB, Zaman K, Roy E, Arifeen SE, Raqib R, Noory L, et al. Combined effects of antenatal receipt of influenza vaccine by mothers and pneumococcal conjugate vaccine receipt by infants: Results from a randomized, blinded, controlled trial. Journal of Infectious Diseases. 2013;207(7):1144–7.

306. Liang L, Xu B, Chen Y, Liu Y, Cao W, Fang L, et al. Combining spatial-temporal and phylogenetic analysis approaches for improved understanding on global H5N1 transmission. PLoS One. 2010;5(10):e13575.

307. Sundaram N, Purohit V, Schaetti C, Kudale A, Joseph S, Weiss MG. Community awareness, use and preference for pandemic influenza vaccines in pune, India. Human Vaccines and Immunotherapeutics. 2015;11(10):2376–88.

308. Verma R, Chayal V, Kumar R, Bhalla K, Dhankar M, Dhaka R, et al. Community perception about swine flu in an urban slum of Haryana: A cross-sectional study. J Family Med Prim Care. 2018;7(6):1515–20.

309. Hunter C, Birden HH, Toribio JA, Booy R, Abdurrahman M, Ambarawati AIGAA, et al. Community preparedness for highly pathogenic avian influenza on Bali and Lombok, Indonesia. Rural and Remote Health [Internet]. 2014;14(3). Available from: https://www.scopus.com/inward/record.uri?eid=2-s2.0-84907151260&partnerID=40&md5=19c52ae9cf345917d425f2b96b262219

310. Padmawati S, Nichter M. Community response to avian flu in Central Java, Indonesia. Anthropol Med. 2008;15(1):31–51.

311. Jadhao SJ, Lee CW, Sylte M, Suarez DL. Comparative efficacy of North American and antigenically matched reverse genetics derived H5N9 DIVA marker vaccines against highly pathogenic Asian H5N1 avian influenza viruses in chickens. Vaccine. 2009;27(44):6247–60.

312. Agrawal AS, Sarkar M, Chakrabarti S, Rajendran K, Kaur H, Mishra AC, et al. Comparative evaluation of real-time PCR and conventional RT-PCR during a 2 year surveillance for influenza and respiratory syncytial virus among children with acute respiratory infections in Kolkata, India, reveals a distinct seasonality of infection. Journal of Medical Microbiology. 2009;58(12):1616–22.

313. Cahyani JI, Widyarini S, Wibowo MH. Comparative safety and efficacy of two bivalent vaccines containing Newcastle disease LaSota and avian influenza H9N2 Sidrap isolate formulated with different oil adjuvants. Veterinary World. 2020;13(11):2493–501.

314. Dayakar S, Pillai HR, Thulasi VP, Jayalekshmi D, Nair RR. Comparative Study of Molecular Approaches for the Detection of Influenza Virus from Patient Samples Using Real-time PCR: Prospective Disease Burden Study in Kerala (India) from 2010 to 2016. Current Infectious Disease Reports [Internet]. 2018;20(8). Available from: https://www.scopus.com/inward/record.uri?eid=2-s2.0-85048303380&doi=10.1007%2fs11908-018-0632-y&partnerID=40&md5=2a62190d574eae71d1ab301c9a6d2d33

315. Thontiravong A, Wannaratana S, Tantilertcharoen R, Prakairungnamthip D, Tuanudom R, Sasipreeyajan J, et al. Comparative study of pandemic (H1N1) 2009, swine H1N1, and avian H3N2 influenza viral infections in quails. Journal of Veterinary Science. 2012;13(4):395–403.

316. Farnsworth ML, Hamilton-West C, Fitchett S, Newman SH, de La Rocque S, De Simone L, et al. Comparing national and global data collection systems for reporting, outbreaks of H5N1 HPAI. Prev Vet Med. 2010;95(3):175–85.

317. Sharma A, Ghosh D, Divekar N, Gore M, Gochhait S, Shireshi SS. Comparing the socio-economic implications of the 1918 Spanish flu and the COVID-19 pandemic in India: A systematic review of literature. Int Soc Sci J. 2021;

318. Joob B, Wiwanitkit V. Comparison between adjuvanted and nonadjuvanted influenza vaccination among patients undergoing hemodialysis: A cost-effectiveness analysis from Thailand. Saudi journal of kidney diseases and transplantation : an official publication of the Saudi Center for Organ Transplantation, Saudi Arabia. 2018;29(2):474–5.

319. Nagarajan S, Murugkar HV, Tosh C, Behera P, Khandia R, Jain R, et al. Comparison of a nucleoprotein gene based RT-PCR with real time RT-PCR for diagnosis of avian influenza in clinical specimens. Research in Veterinary Science. 2012;93(1):504–7.

320. Liao Q, Lam WWT, Fielding R, Bich TH, Dang VT. Comparison of behaviors regarding live poultry exposure among rural residents in Vietnam and Thailand. J Infect Dev Ctries. 2014;8(4):526–34.

321. Kittikraisak W, Ditsungnoen D, Suntarattiwong P, Kanjanapattanakul W, Chotpitayasunondh T, Klungthong C, et al. Comparison of incidence and cost of influenza between healthy and high-risk children <60 months old in Thailand, 2011-2015. PLoS ONE. 2018;13(5):e0197207.

322. Kittikraisak W, Phadungkiatwatana P, Ditsungnoen D, Kaoiean S, Macareo L, Rungrojcharoenkit K, et al. Comparison of influenza antibody titers among women who were vaccinated in the 2nd and the 3rd trimesters of pregnancy. Vaccine. 2021;39(1):18–25.

323. Siengsanan J, Chaichoune K, Phonaknguen R, Sariya L, Prompiram P, Kocharin W, et al. Comparison of outbreaks of H5N1 highly pathogenic avian influenza in wild birds and poultry in Thailand. J Wildl Dis. 2009;45(3):740–7.

324. Parvin R, Schinkoethe J, Grund C, Ulrich R, Bönte F, Behr KP, et al. Comparison of pathogenicity of subtype H9 avian influenza wild-type viruses from a wide geographic origin expressing mono-, di-, or tri-basic hemagglutinin cleavage sites. Veterinary Research [Internet]. 2020;51(1). Available from: https://www.scopus.com/inward/record.uri?eid=2-s2.0-85082739151&doi=10.1186%2fs13567-020-00771-3&partnerID=40&md5=c231594b4990b20f10229315e1287165

325. Wibawa H, Henning J, Waluyati DE, Usman TB, Lowther S, Bingham J, et al. Comparison of serological assays for detecting antibodies in ducks exposed to H5 subtype avian influenza virus. BMC Veterinary Research [Internet]. 2012;8. Available from: https://www.scopus.com/inward/record.uri?eid=2-s2.0-84872461893&doi=10.1186%2f1746-6148-8-117&partnerID=40&md5=708cd11795fe7f7c9b5ec37d2e6985b4

326. Amin M, Yudhawati R, Prasetya RR, Dewantari JR, Nastri AM, Rahardjo K, et al. Comparison of virulence and lethality in mice for avian influenza viruses of two A/H5N1 and one A/H3N6 isolated from poultry during year 2013-2014 in Indonesia. Jpn J Infect Dis. 2020;73(5):336–42.

327. Kiertiburanakul S, Morasert T, Sirinavin S, Chantratita W. Comparisons of clinical characteristics between adult patients with 2009 H1N1 influenza and those with seasonal influenza during the 2009 epidemic in Thailand. Japanese Journal of Infectious Diseases. 2014;67(1):33–9.

328. Wongwiwatwaitaya R, Uppala R, Pithak P, Teeratakulpisarn J. Comparisons of the clinical features and outcomes of children presenting with influenza-like illnesses, including a(H1n1) PDM09 and seasonal influenza, in a university hospital, Thailand. Southeast Asian Journal of Tropical Medicine and Public Health. 2014;45(6):1316–25.

329. Høg E, Fournié G, Hoque MA, Mahmud R, Pfeiffer DU, Barnett T. Competing biosecurity and risk rationalities in the Chittagong poultry commodity chain, Bangladesh. BioSocieties. 2019;14(3):368–92.

330. Roth S, Whitehead S, Thamthitiwat S, Chittaganpitch M, Maloney SA, Baggett HC, et al. Concurrent influenza virus infection and tuberculosis in patients hospitalized with respiratory illness in Thailand. Influenza and other Respiratory Viruses. 2013;7(3):244–8.

331. El-Shesheny R, Franks J, Turner J, Seiler P, Walker D, Friedman K, et al. Continued Evolution of H5Nx Avian Influenza Viruses in Bangladeshi Live Poultry Markets: Pathogenic Potential in Poultry and Mammalian Models. Journal of Virology [Internet]. 2020;94(23). Available from: https://www.scopus.com/inward/record.uri?eid=2-s2.0-85095977736&doi=10.1128%2fJVI.01141-20&partnerID=40&md5=a0afdd3108b0306b113227085a253552

332. Barman S, Turner JCM, Hasan MK, Akhtar S, El-Shesheny R, Franks J, et al. Continuing evolution of highly pathogenic H5N1 viruses in Bangladeshi live poultry markets. Emerging Microbes and Infections. 2019;8(1):650–61.

333. Koul PA, Khan UH, Asad R, Yousuf R, Broor S, Lal RB, et al. Contribution of influenza to acute exacerbations of chronic obstructive pulmonary disease in Kashmir, India, 2010-2012. Influenza and other Respiratory Viruses. 2015;9(1):40–2.

334. Jain A, Prakash S, Jain B. Contribution of non- influenza respiratory viruses in causation of Influenza like Illness (ILI) during influenza epidemic: A laboratory based study. Clinical Epidemiology and Global Health. 2017;5(4):173–5.

335. Parvin R, Nooruzzaman M, Kabiraj CK, Begum JA, Chowdhury EH, Islam MR, et al. Controlling avian influenza virus in Bangladesh: Challenges and recommendations. Viruses [Internet]. 2020;12(7). Available from: https://www.scopus.com/inward/record.uri?eid=2-s2.0-85088035087&doi=10.3390%2fv12070751&partnerID=40&md5=483e9139ee5145da06b0069f4ebde001

336. King DB, Kamble S, DeLongis A. Coping with influenza A/H1N1 in India: empathy is associated with increased vaccination and health precautions. International Journal of Health Promotion and Education. 2016;54(6):283–94.

337. Anonymous. Correction: Risk factors for H5 avian influenza virus prevalence on urban live bird markets in Jakarta, Indonesia-Evaluation of long-term environmental surveillance data (PLoS One (2019) 14:5 (e0216984) DOI: 10.1371/journal.pone.0216984). PLoS ONE. 2019;14(8):e0221611.

338. Karmacharya D, Manandhar S, Sharma A, Bhatta T, Adhikari P, Sherchan AM, et al. Correction: Surveillance of influenza A virus and its subtypes in migratory wild birds of Nepal (PLoS One (2015) 10:7 (e0133035) DOI: 10.1371/journal.pone.0133035). PLoS ONE. 2019;14(6):e0218344.

339. Chudasama RK, Verma PB, Amin CD, Gohel B, Savariya D, Ninama R. Correlates of severe disease in patients admitted with 2009 pandemic influenza A (H1N1) infection in Saurashtra region, India. Indian Journal of Critical Care Medicine. 2010;14(3):113–20.

340. Forrest BD, Pride MW, Dunning AJ, Capeding MRZ, Chotpitayasunondh T, Tam JS, et al. Correlation of cellular immune responses with protection against culture-confirmed influenza virus in young children. Clinical and Vaccine Immunology. 2008;15(7):1042–53.

341. Riewpaiboon A. Cost analysis of influenza vaccination for pregnant women in Thailand. Pharmaceutical Sciences Asia. 2021;48(2):99–106.

342. Doung-ngern P, Ploddi K, Suthachana S, Chaifoo W, Suphanchaimat R, Phaiyarom M, et al. Cost effectiveness and budget impact analyses of influenza vaccination for prisoners in thailand: An application of system dynamic modelling. Int J Environ Res Public Health. 2020;17(4):1247.

343. Stanciole AE, Ortegon M, Chisholm D, Lauer JA. Cost effectiveness of strategies to combat chronic obstructive pulmonary disease and asthma in sub-Saharan Africa and South East Asia: mathematical modelling study. BMJ. 2012;344:e608.

344. Koul PA, Nowshehri AA, Khan UH, Jan RA, Shah SU. Cost of severe chronic obstructive pulmonary disease exacerbations in a high burden region in North India. Annals of Global Health [Internet]. 2019;85(1). Available from: https://www.scopus.com/inward/record.uri?eid=2-s2.0-85061273992&doi=10.5334%2faogh.2423&partnerID=40&md5=d2fef86998e12d954402f5ba48d8249f

345. Karki S, Lupiani B, Budke CM, Karki NPS, Rushton J, Ivanek R. Cost-benefit analysis of avian influenza control in Nepal. Rev - Off Int Epizoot. 2015;34(3):813–27.

346. Wiwanitkit V. Cost-effectiveness analysis for basic screening tests for swine flu in a pandemic. Archives of Hellenic Medicine. 2011;28(5):644–6.

347. Kittikraisak W, Suntarattiwong P, Ditsungnoen D, Pallas SE, Abimbola TO, Klungthong C, et al. Cost-effectiveness of inactivated seasonal influenza vaccination in a cohort of Thai children ≤60 months of age. PLoS One. 2017;12(8):e0183391.

348. Choosakulchart P, Kittisopee T, Takdhada S, Lubell Y, Robinson J. Cost-utility evaluation of influenza vaccination in patients with existing coronary heart diseases in Thailand. Asian Biomed. 2013;7(3):425–35.

349. Bhuiyan MU, Luby SP, Alamgir NI, Homaira N, Sturm-Ramirez K, Gurley ES, et al. Costs of hospitalization with respiratory syncytial virus illness among children aged < 5 years and the financial impact on households in Bangladesh, 2010. Journal of Global Health [Internet]. 2017;7(1). Available from: https://www.scopus.com/inward/record.uri?eid=2-s2.0-85023191269&doi=10.7189%2fjogh.07.010412&partnerID=40&md5=3d1b66ba647d5622912c2add937a2fc9

350. Bhuiyan M, Homaira N, Al Mamun A, Khan J, Gurley ES, Uz Zaman R, et al. Costs of severe pneumonia associated with respiratory viruses among children aged <5 years in four tertiary hospitals in Bangladesh, 2010. Int J Infect Dis. 2012;16:e342.

351. Lertsirimunkong J, Thavornwattanayong W, Hirunkanakorn P, Buranapatanapong R, Jermtienchai S, Mingmoree N. Costs per DALYs Averted of Quadrivalent Influenza Vaccine versus Trivalent Influenza Vaccine in Elderly Population in Thailand. Siriraj Medical Journal. 2021;73(4):259–67.

352. Samaan G, Gultom A, Indriani R, Lokuge K, Kelly PM. Critical control points for avian influenza A H5N1 in live bird markets in low resource settings. Preventive Veterinary Medicine. 2011;100(1):71–8.

353. Chacko J, Gagan B, Ashok E, Radha M, Hemanth HV. Critically ill patients with 2009 H1N1 infection in an Indian ICU. Indian Journal of Critical Care Medicine. 2010;14(2):77–82.

354. Stittelaar KJ, Lacombe V, van Lavieren R, van Amerongen G, Simon J, Cozette V, et al. Cross-clade immunity in cats vaccinated with a canarypox-vectored avian influenza vaccine. Vaccine. 2010;28(31):4970–6.

355. Thongcharoen P, Auewarakul P, Hutagalung Y, Ong G, Gillard P, Drame M, et al. Cross-clade immunogenicity and antigen-sparing with an AS03(A)-adjuvanted prepandemic influenza vaccine in a Thai population. J Med Assoc Thai. 2011;94(8):916–26.

356. Kucharski AJ, Edmunds WJ. Cross-immunity and age patterns of influenza A(H5N1) infection. Epidemiol Infect. 2015;143(6):1119–24.

357. Jusuf MD. Cross-immunity to the 2009 pandemic H1N1 influenza virus among adolescents born 1990-1996 in Indonesia. Turk Pediatr Ars. 2013;48:115.

358. Baras B, Stittelaar KJ, Simon JH, Thoolen RJMM, Mossman SP, Pistoor FHM, et al. Cross-protection against lethal H5N1 challenge in ferrets with an adjuvanted pandemic influenza vaccine. PLoS One. 2008;3(1):e1401.

359. Jiang L, Changsom D, Lerdsamran H, Masamae W, Jongkaewwattana A, Iamsirithaworn S, et al. Cross-reactive antibodies against H7N9 and H5N1 avian influenza viruses in Thai population. Asian Pacific Journal of Allergy and Immunology. 2017;35(1):20–6.

360. Nehul S, Kulkarni A, Pawar S, Godbole S, Ghate M, Thakar M. Cross-reactive influenza-specific antibody-dependent cellular cytotoxicity-mediating antibodies in HIV-infected Indian individuals. Infectious Diseases. 2018;50(1):35–43.

361. Stephenson I, Bugarini R, Nicholson KG, Podda A, Wood JM, Zambon MC, et al. Cross-reactivity to highly pathogenic avian influenza H5N1 viruses after vaccination with nonadjuvanted and MF59-adjuvanted influenza A/Duck/Singapore/97 (H5N3) vaccine: a potential priming strategy. J Infect Dis. 2005;191(8):1210–5.

362. Piewbang C, Rungsipipat A, Poovorawan Y, Techangamsuwan S. Cross-sectional investigation and risk factor analysis of community-acquired and hospital-associated canine viral infectious respiratory disease complex. Heliyon. 2019;5(11):e02726.

363. Karki S, Lupiani B, Budke CM, Manandhar S, Ivanek R. Cross-Sectional Serosurvey of Avian Influenza Antibodies Presence in Domestic Ducks of Kathmandu, Nepal. Zoonoses and Public Health. 2014;61(6):442–8.

364. Sundaram N, Schaetti C, Purohit V, Kudale A, Weiss MG. Cultural epidemiology of pandemic influenza in urban and rural Pune, India: A cross-sectional, mixed-methods study. BMJ Open [Internet]. 2014;4(12). Available from: https://www.scopus.com/inward/record.uri?eid=2-s2.0-84920525212&doi=10.1136%2fbmjopen-2014-006350&partnerID=40&md5=0f5d8d9fff09fc4d21edb9809fe7abf6

365. Delabouglise A, Antoine-Moussiaux N, Tatong D, Chumkaeo A, Binot A, Fournié G, et al. Cultural Practices Shaping Zoonotic Diseases Surveillance: The Case of Highly Pathogenic Avian Influenza and Thailand Native Chicken Farmers. Transboundary and Emerging Diseases. 2017;64(4):1294–305.

366. Ansari I, Pokhrel Y. Culture proven bacterial meningitis in children: Agents, clinical profile and outcome. Kathmandu University Medical Journal. 2011;9(33):36–40.

367. Beigel J, Bray M. Current and future antiviral therapy of severe seasonal and avian influenza. Antiviral Res. 2008;78(1):91–102.

368. Swayne DE, Spackman E. Current status and future needs in diagnostics and vaccines for high pathogenicity avian influenza. Dev Biol (Basel). 2013;135:79–94.

369. Khatun MM, Islam MA, Rahman MM. Current status of veterinary public health activities in Bangladesh and its future plans. BMC Veterinary Research [Internet]. 2019;15(1). Available from: https://www.scopus.com/inward/record.uri?eid=2-s2.0-85066491982&doi=10.1186%2fs12917-019-1879-8&partnerID=40&md5=ceb42f3d4fba16ccf5320a107909180a

370. Lawpoolsri S, Kaewkungwal J, Khamsiriwatchara A, Sovann L, Sreng B, Phommasack B, et al. Data quality and timeliness of outbreak reporting system among countries in Greater Mekong subregion: Challenges for international data sharing. PLoS Neglected Tropical Diseases [Internet]. 2018;12(4). Available from: https://www.scopus.com/inward/record.uri?eid=2-s2.0-85046461301&doi=10.1371%2fjournal.pntd.0006425&partnerID=40&md5=7459e004c4aff5652b2835bdcf9ed4d6

371. Rutvisuttinunt W. Deep sequencing as a tool to identify pathogens from pooled respiratory samples from South/ Southeast Asia. Am J Trop Med Hyg. 2014;91(5):256–7.

372. Durr PA, Wibowo MH, Artanto S, Tarigan S, Rosyid MN, Ignjatovic J. Defining “Sector 3” Poultry Layer Farms in Relation to H5N1-HPAI-An Example from Java, Indonesia. Avian Dis. 2016;60(1):183–90.

373. Dapat C, Saito R, Kyaw Y, Myint YY, Oo HN, Oo KY, et al. Delayed emergence of oseltamivir-resistant seasonal influenza A (H1N1) and pandemic influenza A(H1N1)pdm09 viruses in Myanmar. Influenza and other Respiratory Viruses. 2013;7(5):766–71.

374. Root ED, Agustian D, Kartasasmita C, Uyeki TM, Simões EAF. Demographic and ecological risk factors for human influenza A virus infections in rural Indonesia. Influenza and other Respiratory Viruses. 2017;11(5):425–33.

375. Broor S, Sullender W, Fowler K, Gupta V, Widdowson MA, Krishnan A, et al. Demographic shift of influenza A(H1N1)pdm09 during and after pandemic, rural India. Emerging Infectious Diseases. 2012;18(9):1472–5.

376. Liu WC, Lin SC, Yu YL, Chu CL, Wu SC. Dendritic cell activation by recombinant hemagglutinin proteins of H1N1 and H5N1 influenza A viruses. J Virol. 2010;84(22):12011–7.

377. Singh A, Khera K, Agarwal J, Awasthi S, Francis JM, Thunga G, et al. Descriptive analysis of mortality predictors in H1n1 influenza in south Indian patients. Infectious Disorders - Drug Targets. 2017;17(2):106–15.

378. Virmani N, Bera BC, Gulati BR, Karuppusamy S, Singh BK, Kumar Vaid R, et al. Descriptive epidemiology of equine influenza in India (2008-2009): temporal and spatial trends. Vet Ital. 2010;46(4):449–58.

379. Mahajan SL. Descriptive epidemiology of H1N1 cases in District Amritsar from the year 2009 to 2014. Indian Journal of Public Health Research and Development. 2016;7(2):175–81.

380. Allam RR, Murhekar MV, Tadi GP, Udaragudi PR. Descriptive epidemiology of novel influenza A (H1N1), Andhra Pradesh 2009-2010. Indian journal of public health. 2013;57(3):161–5.

381. Sun G, Vinh NQ, Matsuoka A, Miyata K, Chen C, Ueda A, et al. Design an easy-to-use infection screening system for non-contact monitoring of vital-signs to prevent the spread of pandemic diseases. Annu Int Conf IEEE Eng Med Biol Soc. 2014;2014:4811–4.

382. Sullender W, Fowler K, Krishnan A, Gupta V, Moulton LH, Lafond K, et al. Design and initiation of a study to assess the direct and indirect effects of influenza vaccine given to children in rural India. Vaccine. 2012;30(35):5235–9.

383. Wang W, Ren P, Mardi S, Hou L, Tsai C, Chan KH, et al. design of multiplexed detection assays for identification of avian influenza a virus subtypes pathogenic to humans by SmartCycler real-time reverse transcription-PCR. J Clin Microbiol. 2009;47(1):86–92.

384. Tielsch JM, Steinhoff M, Katz J, Englund JA, Kuypers J, Khatry SK, et al. Designs of two randomized, community-based trials to assess the impact of influenza immunization during pregnancy on respiratory illness among pregnant women and their infants and reproductive outcomes in rural Nepal. BMC Pregnancy and Childbirth [Internet]. 2015;15(1). Available from: https://www.scopus.com/inward/record.uri?eid=2-s2.0-84924159320&doi=10.1186%2fs12884-015-0470-y&partnerID=40&md5=21b36f281ff82966d2dc6a302fcc6d21

385. Yang Y, Halloran ME, Sugimoto JD, Longini IMJ. Detecting human-to-human transmission of avian influenza A (H5N1). Emerg Infect Dis. 2007;13(9):1348–53.

386. Millman AJ, Havers F, Iuliano AD, Davis CT, Sar B, Sovann L, et al. Detecting spread of avian influenza A(H7N9) virus beyond China. Emerging Infectious Diseases. 2015;21(5):741–9.

387. Chi XS, Hu A, Bolar TV, Al-Rimawi W, Zhao P, Tam JS, et al. Detection and characterization of new influenza B virus variants in 2002. J Clin Microbiol. 2005;43(5):2345–9.

388. Islam A, Islam S, Rahman MK, Flora MS, Hossain ME, Rahman MZ, et al. Detection and genetic characterization of avian influenza A (H5N6) virus clade 2.3.4.4 in isolates from house crow and poultry in Bangladesh, 2017. Int J Infect Dis. 2020;101:339–40.

389. Thounaojam AD, Balakrishnan A, Mun AB. Detection and molecular typing of human adenoviruses associated with respiratory illnesses in Kerala. Japanese Journal of Infectious Diseases. 2016;69(6):500–4.

390. Ilyushina NA, Govorkova EA, Webster RG. Detection of amantadine-resistant variants among avian influenza viruses isolated in North America and Asia. Virology. 2005;341(1):102–6.

391. Jones-Engel L, Babo R, Froehlich J, Schillaci MA, Engel GA. Detection of antibodies to selected human pathogens among wild and pet macaques (Macaca tonkeana) in Sulawesi, Indonesia. Am J Primatol. 2001;54(3):171–8.

392. Luangsri N, Barbazan P, Dubot A, Gonzalez JP, Thitithanyanont A, Misse D, et al. Detection of H5N1 avian influenza virus from mosquitoes collected in an infected poultry farm in Thailand. Vector Borne Zoonotic Dis. 2008;8(1):105–9.

393. Yang G, Chowdury S, Hodges E, Rahman MZ, Jang Y, Hossain ME, et al. Detection of highly pathogenic avian influenza A(H5N6) viruses in waterfowl in Bangladesh. Virology. 2019;534:36–44.

394. Chowdhury MNU, Islam S, Hossain ME, Rahman MZ, Zulkar Nine HSM, Doe MK, et al. Detection of Influenza A and Adenovirus in captive wild birds in Bangladesh. Int J Infect Dis. 2020;101:229.

395. Sangkakam A, Hemachudha P, Saraya AW, Thaweethee-Sukjai B, Wacharapluesadee S, Cheun-Arom T, et al. Detection of influenza virus in rectal swabs of patients admitted in hospital for febrile illnesses in Thailand. SAGE Open Med [Internet]. 2021;9. Available from: http://smo.sagepub.com/

396. Wasito R, Wuryastuti H, Sutrisno B. Detection of mixed infection of avian influenza and newcastle disease viruses in chickens in Indonesia by immunopathologic immunohistochemistry double staining. Pakistan Veterinary Journal. 2018;38(4):442–5.

397. Pawestri HA, Nugraha AA, Hariastuti NI, Setiawaty V. Detection of neuraminidase inhibitor-resistant influenza A (H1N1)pdm09 viruses obtained from influenza surveillance in Indonesia. SAGE Open Med [Internet]. 2018;6. Available from: http://smo.sagepub.com/

398. Payungporn S, Poomipak W, Makkoch J, Rianthavorn P, Theamboonlers A, Poovorawan Y. Detection of oseltamivir sensitive/resistant strains of pandemic influenza A virus (H1N1) from patients admitted to hospitals in Thailand. Journal of Virological Methods. 2011;177(2):133–9.

399. Makkoch J, Payungporn S, Prachayangprecha S, Tantilertcharoen R, Poovorawan Y. Determination of antibody response to the human pandemic influenza H1N1 2009 among patients with influenza-like illness and high risk groups. Asian Pacific Journal of Allergy and Immunology. 2010;28(1):69–77.

400. Arunorat J, Charoenvisal N, Woonwong Y, Kedkovid R, Thanawongnuwech R. Determination of current reference viruses for serological study of swine influenza viruses after the introduction of pandemic 2009 H1N1 (pdmH1N1) in Thailand. Journal of Virological Methods. 2016;236:5–9.

401. Yuyun I, Wibawa H, Setiaji G, Kusumastuti TA, Nugroho WS. Determining highly pathogenic H5 avian influenza clade 2.3.2.1c seroprevalence in ducks, Purbalingga, Central Java, Indonesia. Veterinary World. 2020;13(6):1138–44.

402. Giduthuri JG, Maire N, Schaetti C, Sundaram N, Schindler C, Weiss MG, et al. Developing and validating a tablet version of an illness explanatory model interview for a public health survey in pune, india. PLoS ONE. 2014;9(9):5.

403. Durr PA, Indriani R, Selleck P, Adjid ARM, Syafriati T, Ignjatovic J. Developing farm-level post-vaccination sero-monitoring systems for H5N1 highly pathogenic avian influenza in an endemically infected country. Frontiers in Veterinary Science [Internet]. 2019;5. Available from: https://www.scopus.com/inward/record.uri?eid=2-s2.0-85060246717&doi=10.3389%2ffvets.2018.00324&partnerID=40&md5=e190b5e09ba387be9dde2cf944b32cc9

404. Arora R, Prabha S, Bagai P, Mandal P, Chandra J, Moulik N. Developing immunization solutions for children with cancer in India: A quality improvement initiative. Pediatr Blood Cancer. 2015;62:S375.

405. Rudenko L, Yeolekar L, Kiseleva I, Isakova-Sivak I. Development and approval of live attenuated influenza vaccines based on Russian master donor viruses: Process challenges and success stories. Vaccine. 2016;34(45):5436–41.

406. Hegde NR, Kumar D, Rao PP, Kumari PK, Kaushik Y, Ravikrishnan R, et al. Development and preclinical testing of HNVAC, a cell culture-based H1N1 pandemic influenza vaccine from India. Vaccine. 2014;32(29):3636–43.

407. Dong J, Matsuoka Y, Maines TR, Swayne DE, O’Neill E, Davis CT, et al. Development of a new candidate H5N1 avian influenza virus for pre-pandemic vaccine production. Influenza Other Respir Viruses. 2009;3(6):287–95.

408. Hoelscher MA, Garg S, Bangari DS, Belser JA, Lu X, Stephenson I, et al. Development of adenoviral-vector-based pandemic influenza vaccine against antigenically distinct human H5N1 strains in mice. Lancet. 2006;367(9509):475–81.

409. Surichan S, Wirachwong P, Supachaturas W, Utid K, Theerasurakarn S, Langsanam P, et al. Development of influenza vaccine production capacity by the Government Pharmaceutical Organization of Thailand: Addressing the threat of an influenza pandemic. Vaccine. 2011;29:A29–33.

410. Hasegawa H, Ichinohe T, Ainai A, Tamura SI, Kurata T. Development of mucosal adjuvants for intranasal vaccine for H5N1 influenza viruses. Ther Clin Risk Manag. 2009;5(1):125–32.

411. Broor S, Chahar HS, Kaushik S. Diagnosis of influenza viruses with special reference to novel H1N1 2009 influenza virus. Indian Journal of Microbiology. 2009;49(4):301–7.

412. Robyn M, Priyono WB, Kim LM, Brum E. Diagnostic sensitivity and specificity of a participatory disease surveillance method for highly pathogenic avian influenza in household chicken flocks in Indonesia. Avian Diseases. 2012;56(2):377–80.

413. Newman KL, Gustafson K, Chu HY, Englund JA, Khatry S, Le Clerq S, et al. Diarrheal Illness as a risk factor for subsequent respiratory infection among women and infants in Nepal. Open Forum Infect Dis. 2019;6:S755–6.

414. Sakabe S, Takano R, Nagamura-Inoue T, Yamashita N, Nidom CA, Quynh Le MT, et al. Differences in cytokine production in human macrophages and in virulence in mice are attributable to the acidic polymerase protein of highly pathogenic influenza A virus subtype H5N1. Journal of Infectious Diseases. 2013;207(2):262–71.

415. Koul PA, Broor S, Saha S, Barnes J, Smith C, Shaw M, et al. Differences in influenza seasonality by latitude, Northern India. Emerging Infectious Diseases. 2014;20(10):1723–6.

416. Thube MM, Shil P, Kasbe R, Patil AA, Pawar SD, Mullick J. Differences in Type I interferon response in human lung epithelial cells infected by highly pathogenic H5N1 and low pathogenic H11N1 avian influenza viruses. Virus Genes. 2018;54(3):414–23.

417. Hayashi T, Chaichoune K, Patchimasiri T, Hiromoto Y, Kawasaki Y, Wiriyarat W, et al. Differential host gene responses in mice infected with two highly pathogenic avian influenza viruses of subtype H5N1 isolated from wild birds in Thailand. Virology. 2011;412(1):9–18.

418. Basu A, Shelke V, Chadha M, Kadam D, Sangle S, Gangodkar S, et al. Direct imaging of pH1N1 2009 influenza virus replication in alveolar pneumocytes in fatal cases by transmission electron microscopy. J Electron Microsc (Tokyo). 2011;60(1):89–93.

419. Kshirsagar DP, Savalia CV, Kalyani IH, Kumar R, Nayak DN. Disease alerts and forecasting of zoonotic diseases: An overview. Veterinary World. 2013;6(11):889–96.

420. Adiga A, Chu S, Eubank S, Kuhlman CJ, Lewis B, Marathe A, et al. Disparities in spread and control of influenza in slums of Delhi: findings from an agent-based modelling study. BMJ Open. 2018;8(1):e017353.

421. Kanai Y, Chittaganpitch M, Nakamura I, Li GM, Bai GR, Li YG, et al. Distinct propagation efficiencies of H5N1 influenza virus Thai isolates in newly established murine respiratory region-derived cell clones. Virus Research. 2010;153(2):218–25.

422. Cheung CL, Rayner JM, Smith GJD, Wang P, Naipospos TSP, Zhang J, et al. Distribution of amantadine-resistant H5N1 avian influenza variants in Asia. J Infect Dis. 2006;193(12):1626–9.

423. Fernandez S, Klungthong C, Thaisomboonsuk B, Rodpradit P, Mongkolsirichaikul D, Yoon IK. Distribution of influenza anti-viral resistance in Southeast Asia in 2012-13. Am J Trop Med Hyg. 2014;91(5):261.

424. Caini S, Spreeuwenberg P, Kusznierz GF, Rudi JM, Owen R, Pennington K, et al. Distribution of influenza virus types by age using case-based global surveillance data from twenty-nine countries, 1999-2014. BMC Infectious Diseases [Internet]. 2018;18(1). Available from: https://www.scopus.com/inward/record.uri?eid=2-s2.0-85048291114&doi=10.1186%2fs12879-018-3181-y&partnerID=40&md5=abca8acde91a56c0f0c84089b7aec56d

425. Sherchand JB, Joshi AR, Gauchan P, Amatya J. Distribution of serotypes and antimicrobial resistance of Streptococcus pneumoniae in a children’s hospital in Nepal. Journal of Nepal Paediatric Society. 2008;28(2):45–8.

426. Saha S, Chadha M, Shu Y, Lijie W, Chittaganpitch M, Waicharoen S, et al. Divergent seasonal patterns of influenza types A and B across latitude gradient in Tropical Asia. Influenza and other Respiratory Viruses. 2016;10(3):176–84.

427. Wangdi K, Kasturiaratchi K, Nery SV, Lau CL, Gray DJ, Clements ACA. Diversity of infectious aetiologies of acute undifferentiated febrile illnesses in south and Southeast Asia: A systematic review. BMC Infectious Diseases [Internet]. 2019;19(1). Available from: https://www.scopus.com/inward/record.uri?eid=2-s2.0-85068577306&doi=10.1186%2fs12879-019-4185-y&partnerID=40&md5=1ae4197af9b8ffd92da1fcbae09832aa

428. Praphasiri P, Ditsungneon D, Greenbaum A, Dawood FS, Olsen SJ, Lindblade KA, et al. Do Thai physicians recommend seasonal influenza vaccines to pregnant women? A cross-sectional survey of physicians’perspectives and practices in Thailand. PLoS ONE. 2017;12(1):e0169221.

429. Assanangkornchai N, Bhurayanontachai R. Does treatment with ECMO improve the outcome of patients with severe acute respiratory distress syndrome (ARDS)? A case control study in a southern Thailand hospital. Perfusion. 2019;34(1):135–6.

430. Jam-On R, Sae-Heng N, Meemak N, Hulse-Post DJ, Sturm-Ramirez KM, Songserm T, et al. Domestic ducks and H5N1 influenza epidemic, Thailand. Emerg Infect Dis. 2006;12(4):575–81.

431. Keitel W, Groth N, Lattanzi M, Praus M, Hilbert AK, Borkowski A, et al. Dose ranging of adjuvant and antigen in a cell culture H5N1 influenza vaccine: safety and immunogenicity of a phase 1/2 clinical trial. Vaccine. 2010;28(3):840–8.

432. Kumar A, Sharma DK, Mohan A, Srivastava S, Srivastava R, Rawat AK. Drug design for influenza a pandemic (H1N1) 2009 virus isolates from India. Trends Bioinformatics. 2012;5(1):1–13.

433. Gohil DJ, Kothari ST, Shinde PS, Chintakrindi AS, Meharunkar R, Warke RV, et al. Drug susceptibility of influenza A/H3N2 strains co-circulating during 2009 influenza pandemic: First report from Mumbai. Infection, Genetics and Evolution. 2015;29:75–81.

434. Gunardi H, Rusmil K, Fadlyana E, Dhamayanti M, Sekartini R, Tarigan R, et al. DTwP-HB-Hib: Antibody persistence after a primary series, immune response and safety after a booster dose in children 18-24 months old. BMC Pediatrics [Internet]. 2018;18(1). Available from: https://www.scopus.com/inward/record.uri?eid=2-s2.0-85047512227&doi=10.1186%2fs12887-018-1143-6&partnerID=40&md5=fcd9c557356521863b1e5ff27d290aee

435. Park AW, Glass K. Dynamic patterns of avian and human influenza in east and southeast Asia. Lancet Infect Dis. 2007;7(8):543–8.

436. Broor S, Krishnan A, Dhakad S, Kaushik S, Mir MA, Singh Y, et al. Dynamic patterns of circulating seasonal and pandemic A(H1N1)pdm09 influenza viruses from 2007-2010 in and around Delhi, India. PLoS ONE. 2012;7(1):e29129.

437. Ratre YK, Vishvakarma NK, Bhaskar LVKS, Verma HK. Dynamic Propagation and Impact of Pandemic Influenza A (2009 H1N1) in Children: A Detailed Review. Current Microbiology. 2020;77(12):3809–20.

438. Mathur KS, Narayan P. Dynamics of an SVEIRS Epidemic Model with Vaccination and Saturated Incidence Rate. Int J Appl Comput Math. 2018;4(5):118.

439. Chadha MS, Potdar VA, Mishra AC, Saha S, Lal RB, Koul PA, et al. Dynamics of influenza seasonality at sub-regional levels in India and implications for vaccination timing. PLoS ONE. 2015;10(5):e0124122.

440. Retkute R, Jewell CP, Van Boeckel TP, Zhang G, Xiao X, Thanapongtharm W, et al. Dynamics of the 2004 avian influenza H5N1 outbreak in Thailand: The role of duck farming, sequential model fitting and control. Preventive Veterinary Medicine. 2018;159:171–81.

441. Kiruba R, Suresh Babu BV, Sheriff AK, Gunasekaran P, Anupama CP, Saran N, et al. Dynamics of the occurrence of influenza in relation to seasonal variation in Chennai, Tamil Nadu: A 7 -year cumulative study. Indian Journal of Medical Microbiology. 2019;37(3):401–5.

442. Hanvoravongchai P, Coker R. Early reporting of pandemic flu and the challenge of global surveillance: A lesson for Southeast Asia. Southeast Asian Journal of Tropical Medicine and Public Health. 2011;42(5):1093–9.

443. Newman SH, Hill NJ, Spragens KA, Janies D, Voronkin IO, Prosser DJ, et al. Eco-virological approach for assessing the role of wild birds in the spread of avian influenza H5N1 along the Central Asian Flyway. PLoS One. 2012;7(2):e30636.

444. Toft N, Ahmed SSU, Ersboll AK, Biswas PK, Christensen JP, Hannan ASMA. Ecological determinants of highly pathogenic avian influenza (H5N1) outbreaks in Bangladesh. PLoS ONE. 2012;7(3):e33938.

445. Ren H, Jin Y, Hu M, Zhou J, Song T, Huang Z, et al. Ecological dynamics of influenza A viruses: cross-species transmission and global migration. Sci Rep. 2016;6:36839.

446. Slingenbergh JI, Gilbert M, de Balogh KI, Wint W. Ecological sources of zoonotic diseases. Rev Sci Tech. 2004;23(2):467–84.

447. Stegeman A, Nielen M, Tiensin T, Chanachai K, Thanapongtham W, Kalpravidh W, et al. Ecologie risk factor investigation of clusters of avian influenza a (H5N1) virus infection in thailand. J Infect Dis. 2009;199(12):1736–43.

448. Kiertiburanakul S, Phongsamart W, Tantawichien T, Manosuthi W, Kulchaitanaroaj P. Economic Burden of Influenza in Thailand: A Systematic Review. Inquiry (United States) [Internet]. 2020;57. Available from: https://www.scopus.com/inward/record.uri?eid=2-s2.0-85098012020&doi=10.1177%2f0046958020982925&partnerID=40&md5=7b156a29e91687b0827d78090f4c1aa0

449. Bhuiyan MU, Luby SP, Alamgir NI, Homaira N, Mamun AA, Khan JAM, et al. Economic burden of influenza-associated hospitalizations and outpatient visits in Bangladesh during 2010. Influenza and other Respiratory Viruses. 2014;8(4):406–13.

450. Wongsurakiat P, Lertakyamanee J, Maranetra KN, Jongriratanakul S, Sangkaew S. Economic evaluation of influenza vaccination in Thai chronic obstructive pulmonary disease patients. Journal of the Medical Association of Thailand. 2003;86(6):497–508.

451. Govindaraj G, Sridevi R, Nandakumar SN, Vineet R, Rajeev P, Binu MK, et al. Economic impacts of avian influenza outbreaks in Kerala, India. Transboundary and Emerging Diseases. 2018;65(2):e361–72.

452. Hughes MM, Praphasiri P, Dawood FS, Sornwong K, Ditsungnoen D, Mott JA, et al. Effect of acute respiratory illness on short-term frailty status of older adults in Nakhon Phanom, Thailand—June 2015 to June 2016: A prospective matched cohort study. Influenza and other Respiratory Viruses. 2019;13(4):391–7.

453. Sachan N, Singh VP. Effect of climatic changes on the prevalence of zoonotic diseases. Veterinary World. 2010;3(11):519–22.

454. Newman KL, Gustafson K, Englund JA, Magaret A, Khatry S, LeClerq SC, et al. Effect of diarrheal illness during pregnancy on adverse birth outcomes in Nepal. Open Forum Infectious Diseases [Internet]. 2019;6(2). Available from: https://www.scopus.com/inward/record.uri?eid=2-s2.0-85066488904&doi=10.1093%2fofid%2fofz011&partnerID=40&md5=1c21969a35220fb8af7bc9f8d6dc27a1

455. Sedyaningsih ER, Malik MS, Setiawaty V, Trihono T, Burhan E, Aditama TY, et al. Effect of double dose oseltamivir on clinical and virological outcomes in children and adults admitted to hospital with severe influenza: Double blind randomised controlled trial. BMJ (Online) [Internet]. 2013;346(7911). Available from: https://www.scopus.com/inward/record.uri?eid=2-s2.0-84878821350&doi=10.1136%2fbmj.f3039&partnerID=40&md5=76a5f6e53bcddeac5443f210be340484

456. Kaewchana S, Simmerman M, Somrongthong R, Suntarattiwong P, Lertmaharit S, Chotipitayasunondh T. Effect of intensive hand washing education on hand washing behaviors in Thai households with an influenza-positive child in urban Thailand. Asia-Pacific Journal of Public Health. 2012;24(4):577–85.

457. Chen J, Chu S, Chungbaek Y, Khan M, Kuhlman C, Marathe A, et al. Effect of modelling slum populations on influenza spread in Delhi. BMJ Open. 2016;6(9):e011699.

458. Murray A, Englund J, Kuypers J, Tielsch J, Katz J, Shrestha L, et al. Effect of nasopharyngeal pneumococcal carriage on RSV and HMPV illness severity in infants in Nepal. Open Forum Infect Dis. 2018;5:S4.

459. Beigel JH, Manosuthi W, Beeler J, Bao Y, Hoppers M, Ruxrungtham K, et al. Effect of oral oseltamivir on virological outcomes in low-risk adults with influenza: A randomized clinical trial. Clinical Infectious Diseases. 2020;70(11):2317–24.

460. van der Goot JA, van Boven M, de Jong MCM, Koch G. Effect of vaccination on transmission of HPAI H5N1: the effect of a single vaccination dose on transmission of highly pathogenic avian influenza H5N1 in Peking ducks. Avian Dis. 2007;51(1):323–4.

461. Biswas D, Ahmed M, Roguski K, Ghosh PK, Parveen S, Nizame FA, et al. Effectiveness of a behavior change intervention with hand sanitizer use and respiratory hygiene in reducing laboratory-confirmed influenza among schoolchildren in Bangladesh: A cluster randomized controlled trial. American Journal of Tropical Medicine and Hygiene. 2019;101(6):1446–55.

462. Kulkarni PS, Agarkhedkar S, Lalwani S, Bavdekar AR, Jog S, Raut SK, et al. Effectiveness of an Indian-made attenuated influenza A(H1N1)pdm 2009 vaccine: A case control study. Human Vaccines and Immunotherapeutics. 2014;10(3):566–71.

463. Adisasmito W, Chan PKS, Lee N, Oner AF, Gasimov V, Aghayev F, et al. Effectiveness of antiviral treatment in human influenza A(H5N1) infections: Analysis of a global patient registry. Journal of Infectious Diseases. 2010;202(8):1154–60.

464. Biswas D, Chowdhury F, Nizame FA, Parveen S, Ghosh PK, Khan SH, et al. Effectiveness of hand sanitizer with hand and respiratory hygiene education in reducing influenza-like illness and laboratory confirmed influenza among school children in Bangladesh, 2015. Am J Trop Med Hyg. 2017;97(5):212–3.

465. Mangklakeree N, Pinitsoontorn S, Srisaenpang S. Effectiveness of influenza control using nonpharmaceutical interventions at primary schools in Nakhon Phanom Province, Northeast Thailand. Asian Biomed. 2014;8(3):405–10.

466. Zaman K, Roy E, Arifeen SE, Rahman M, Raqib R, Wilson E, et al. Effectiveness of maternal influenza immunization in mothers and infants. New England Journal of Medicine. 2008;359(15):1555–64.

467. Sonthichai C, Iamsirithaworn S, Cummings D, Shokekird P, Niramitsantipong A, Khumket S, et al. Effectiveness of Non-pharmaceutical Interventions in Controlling an Influenza A Outbreak in a School, Thailand, November 2007. Outbreak Surveill Investig Rep. 2011;4(2):611.

468. Dawood FS, Prapasiri P, Areerat P, Ruayajin A, Chittaganpitch M, Muangchana C, et al. Effectiveness of the 2010 and 2011 Southern Hemisphere trivalent inactivated influenza vaccines against hospitalization with influenza-associated acute respiratory infection among Thai adults aged ≥50 years. Influenza and other Respiratory Viruses. 2014;8(4):463–8.

469. Kittikraisak W, Suntarattiwong P, Ditsungnoen D, Klungthong C, Fernandez S, Yoon IK, et al. Effectiveness of the 2013 and 2014 Southern Hemisphere influenza vaccines against laboratory-confrmed influenza in young children using a test-negative design, Bangkok, Thailand. Pediatric Infectious Disease Journal. 2016;35(10):e318–25.

470. Prasert K, Patumanond J, Praphasiri P, Siriluk S, Ditsungnoen D, Chittaganpich M, et al. Effectiveness of trivalent inactivated influenza vaccine among community-dwelling older adults in Thailand: A two-year prospective cohort study. Vaccine. 2019;37(6):783–91.

471. Mangklakeeree N, Pinitsoontorn S, Srisaenpang S. Effects of an influenza prevention program using non-pharmaceutical prevention measures to improve the knowledge, attitudes and practices of elementary school students in Nakhon Phanom province, Thailand. Southeast Asian J Trop Med Public Health. 2013;44(4):630–5.

472. Jaiwong C, Ngamphaiboon J. Effects of inactivated influenza vaccine on respiratory illnesses and asthma-related events in children with mild persistent asthma in Asia. Asian Pacific Journal of Allergy and Immunology. 2015;33(1):3–7.

473. Jaiwong C, Siripipattanamongkol N, Ngamphaiboon J. Effects of influenza vaccine in children with moderate to severe allergic rhinitis. Journal of the Medical Association of Thailand. 2017;100(11):1189–95.

474. Fry AM, Goswami D, Nahar K, Sharmin AT, Rahman M, Gubareva L, et al. Effects of oseltamivir treatment of index patients with influenza on secondary household illness in an urban setting in Bangladesh: Secondary analysis of a randomised, placebo-controlled trial. The Lancet Infectious Diseases. 2015;15(6):654–62.

475. Zaman K, Goswami D, Sharmeen AT, Nahar K, Rahman M, Rahman MZ, et al. Efficacy of a Russian-backbone live attenuated influenza vaccine among young children in Bangladesh: a randomised, double-blind, placebo-controlled trial. Lancet Global Health. 2016;4(12):e946–54.

476. Block SL, Toback SL, Yi T, Ambrose CS. Efficacy of a single dose of live attenuated influenza vaccine in previously unvaccinated children: a post hoc analysis of three studies of children aged 2 to 6 years. Clin Ther. 2009;31(10):2140–7.

477. De Vleeschauwer AR, Baras B, Kyriakis CS, Jacob V, Planty C, Giannini SL, et al. Efficacy of an AS03A-adjuvanted split H5N1 influenza vaccine against an antigenically distinct low pathogenic H5N1 virus in pigs. Vaccine. 2012;30(37):5557–63.

478. Sullender WM, Fowler KB, Gupta V, Krishnan A, Ram Purakayastha D, Srungaram VLN R, et al. Efficacy of inactivated trivalent influenza vaccine in rural India: a 3-year cluster-randomised controlled trial. Lancet Global Health. 2019;7(7):e940–50.

479. Krishnan A, Dar L, Saha S, Narayan VV, Kumar R, Kumar R, et al. Efficacy of live attenuated and inactivated influenza vaccines among children in rural India: A 2-year, randomized, triple-blind, placebo-controlled trial. PLoS Medicine [Internet]. 2021;18(4). Available from: https://www.scopus.com/inward/record.uri?eid=2-s2.0-85105548100&doi=10.1371%2fjournal.pmed.1003609&partnerID=40&md5=d7a3237040a8c7cd54f410e44765fb3c

480. Fry AM, Gubareva L, Bresee J, Goswami D, Nahar K, Sharmin AT, et al. Efficacy of oseltamivir treatment started within 5 days of symptom onset to reduce influenza illness duration and virus shedding in an urban setting in Bangladesh: A randomised placebo-controlled trial. Lancet Infect Dis. 2014;14(2):109–18.

481. Krairittichai U, Chittaganpitch M. Efficacy of the trivalent influenza vaccination in thai patients with hemodialysis or kidney transplant compared with healthy volunteers. Journal of the Medical Association of Thailand. 2013;96:1–7.

482. Bublot M, Le Gros FX, Nieddu D, Pritchard N, Mickle TR, Swayne DE. Efficacy of two H5N9-inactivated vaccines against challenge with a recent H5N1 highly pathogenic avian influenza isolate from a chicken in Thailand. Avian Dis. 2007;51:332–7.

483. Kwon JH, Criado MF, Killmaster L, Ali MZ, Giasuddin M, Samad MA, et al. Efficacy of two vaccines against recent emergent antigenic variants of clade 2.3.2.1a highly pathogenic avian influenza viruses in Bangladesh. Vaccine. 2021;39(21):2824–32.

484. Omer SB, Clark DR, Madhi SA, Tapia MD, Nunes MC, Cutland CL, et al. Efficacy, duration of protection, birth outcomes, and infant growth associated with influenza vaccination in pregnancy: a pooled analysis of three randomised controlled trials. The Lancet Respiratory Medicine. 2020;8(6):597–608.

485. Pandey A, Singh N, Sambhara S, Mittal SK. Egg-independent vaccine strategies for highly pathogenic H5N1 influenza viruses. Hum Vaccin. 2010;6(2):178–88.

486. Levy JW, Bhoomiboonchoo P, Simasathien S, Salje H, Huang A, Rangsin R, et al. Elevated transmission of upper respiratory illness among new recruits in military barracks in Thailand. Influenza and other Respiratory Viruses. 2015;9(6):308–14.

487. Hurt AC, Ernest J, Deng YM, Iannello P, Besselaar TG, Birch C, et al. Emergence and spread of oseltamivir-resistant A(H1N1) influenza viruses in Oceania, South East Asia and South Africa. Antiviral Research. 2009;83(1):90–3.

488. Broor S, Gupta S, Mohapatra S, Kaushik S, Mir MA, Jain P, et al. Emergence of 2009A/H1N1 cases in a tertiary care hospital in New Delhi, India. Influenza and other Respiratory Viruses. 2011;5(6):e552–7.

489. Tosh C, Murugkar HV, Nagarajan S, Tripathi S, Katare M, Jain R, et al. Emergence of amantadine-resistant avian influenza H5N1 virus in India. Virus Genes. 2011;42(1):10–5.

490. Parida M, Dash PK, Kumar JS, Joshi G, Tandel K, Sharma S, et al. Emergence of influenza A(H1N1)pdm09 genogroup 6B and drug resistant virus, India, January to May 2015. Eurosurveillance [Internet]. 2016;21(5). Available from: https://www.scopus.com/inward/record.uri?eid=2-s2.0-84957556702&doi=10.2807%2f1560-7917.ES.2016.21.5.30124&partnerID=40&md5=4e61d25da7c77cc47c791bbb09a7db34

491. Kumar M, Nagarajan S, Murugkar HV, Saikia B, Singh B, Mishra A, et al. Emergence of novel reassortant H6N2 avian influenza viruses in ducks in India. Infection, Genetics and Evolution. 2018;61:20–3.

492. Choudhry A, Singh S, Khare S, Rai A, Rawat DS, Aggarwal RK, et al. Emergence of pandemic 2009 influenza A H1N1, India. Indian J Med Res. 2012;135(4):534–7.

493. Venkataramana M, Vindal V, Kondapi AK. Emergence of Swine flu in Andhra Pradesh: Facts and future. Indian Journal of Microbiology. 2009;49(4):320–3.

494. Tenzin T, Tenzin S, Tshering D, Lhamo K, Rai PB, Dahal N, et al. Emergency surveillance for novel influenza A(H7N9) virus in domestic poultry, feral pigeons and other wild birds in Bhutan. OIE Revue Scientifique et Technique. 2015;34(3):829–36.

495. Mohapatra S, Dar L. Emerging and remerging viral infections in India. Journal International Medical Sciences Academy. 2010;23(1):37–40.

496. Krilov LR. Emerging infectious disease issues in international adoptions: severe acute respiratory syndrome (SARS), avian influenza and measles. Curr Opin Infect Dis. 2004;17(5):391–5.

497. Coker RJ, Hunter BM, Rudge JW, Liverani M, Hanvoravongchai P. Emerging infectious diseases in southeast Asia: Regional challenges to control. Lancet. 2011;377(9765):599–609.

498. Mani RS, Ravi V, Desai A, Madhusudana SN. Emerging Viral Infections in India. Proc Natl Acad Sci India Sect B Biol Sci. 2012;82(1):5–21.

499. Tun Win Y, Gardner E, Hadrill D, Su Mon CC, Kyin MM, Maw MT, et al. Emerging Zoonotic Influenza A Virus Detection in Myanmar: Surveillance Practices and Findings. Health Security. 2017;15(5):483–93.

500. Kulkarni R, Kinikar A. Encephalitis in a child with H1N1 infection: First case report from India. Journal of Pediatric Neurosciences. 2010;5(2):157–9.

501. Lokida D, Martini T, Trihono T, Setiawaty V, Mahoney F. Enhanced surveillance for patients with novel H1N1 (nH1N1) in Tangerang District Indonesia. Eur Respir J [Internet]. 2011;38. Available from: http://erj.ersjournals.com/content/38/Suppl_55/p4147

502. Indriani R, Samaan G, Gultom A, Loth L, Irianti S, Adjid R, et al. Environmental sampling for avian influenza virus a (H5N1) in live-bird markets, Indonesia. Emerging Infectious Diseases. 2010;16(12):1889–95.

503. Win SMK, Win NC, Lasham DJ, Saito R, Chon I, Kyaw Y, et al. Epidemic of influenza A(H1N1)pdm09 analyzed by full genome sequences and the first case of oseltamivir-resistant strain in Myanmar 2017. PLoS ONE. 2020;15(3):e0229601.

504. Dubal ZB, Barbuddhe SB, Singh NP. Epidemics of emerging and re-emerging viral Zoonotic diseases in India - An overview. Journal of Communicable Diseases. 2013;45(3):105–22.

505. Malhotra B, Singh R, Sharma P, Meena D, Gupta J, Atreya A, et al. Epidemiological & clinical profile of influenza A (H1N1) 2009 virus infections during 2015 epidemic in Rajasthan. Indian J Med Res. 2016;144(6):918–23.

506. Wangchuk S, Thapa B, Zangmo S, Jarman R, Gibbons RV, Bhoomiboonchoo P. Epidemiological analysis of the pandemic influenza A (H1N1) virus in Bhutan. Int J Infect Dis. 2012;16:e118.

507. Prachayangprecha S, Makkoch J, Vuthitanachot C, Vuthitanachot V, Payungporn S, Chieochansin T, et al. Epidemiological and serological surveillance of human pandemic influenza a virus infections during 2009-2010 in Thailand. Japanese Journal of Infectious Diseases. 2011;64(5):377–81.

508. Magazine R, Rao S, Chogtu B, Venkateswaran R, Shahul HA, Goneppanavar U. Epidemiological profile of acute respiratory distress syndrome patients: A tertiary care experience. Lung India. 2017;34(1):38–42.

509. Mahendra S, Suman B, Afzal H, Savitri S. Epidemiological profile of H1N1 cases in western Rajasthan from January 2012 to December 2012. Indian Journal of Public Health Research and Development. 2015;6(2):118–22.

510. Mavadiya SV, Raval SK, Mehta SA, Kanani AN, Vagh AA, Tank PH, et al. Epidemiological survey of equine influenza in horses in India. Rev Sci Tech. 2012;31(3):871–5.

511. Dubey SC, Venkatesh G, Kulkarni DD. Epidemiological update on swine influenza (H1N1) in pigs. Indian Journal of Microbiology. 2009;49(4):324–31.

512. Horthongkham N, Athipanyasilp N, Pattama A, Kaewnapan B, Sornprasert S, Srisurapanont S, et al. Epidemiological, Clinical and Virological Characteristics of Influenza B Virus from Patients at the Hospital Tertiary Care Units in Bangkok during 2011-2014. PLoS One. 2016;11(7):e0158244.

513. Koul PA, Bhavsar A, Mir H, Simmerman M, Khanna H. Epidemiology and costs of severe acute respiratory infection and influenza hospitalizations in adults with diabetes in India. Journal of Infection in Developing Countries. 2019;13(3):204–11.

514. Lubroth J, Slingenbergh J, Domenech J, Sims L, Pfeiffer D, Martin V. Epidemiology and ecology of highly pathogenic avian influenza with particular emphasis on South East Asia. Dev Biol. 2006;124:23–36.

515. Palani N, Sistla S. Epidemiology and phylogenetic analysis of respiratory viruses from 2012 to 2015 – A sentinel surveillance report from union territory of Puducherry, India. Clinical Epidemiology and Global Health. 2020;8(4):1225–35.

516. El Guerche-Séblain C, Caini S, Paget J, Vanhems P, Schellevis F. Epidemiology and timing of seasonal influenza epidemics in the Asia-Pacific region, 2010-2017: implications for influenza vaccination programs. BMC Public Health. 2019;19(1):331.

517. Tam CC, Anderson KB, Offeddu V, Weg A, Macareo LR, Ellison DW, et al. Epidemiology and transmission of respiratory infections in Thai Army recruits: A prospective cohort study. American Journal of Tropical Medicine and Hygiene. 2018;99(4):1089–95.

518. Isfandari S, Setiawaty V, Rifati L, Harun S, Soendoro T, Purba W, et al. Epidemiology of cases of H5N1 virus infection in Indonesia, July 2005-June 2006. J Infect Dis. 2007;196(4):522–7.

519. Arunkumar G, Robin S, Sushama A, Sabeena SP, Devadiga S, Nair S, et al. Epidemiology of dengue in India-outcome of a facility based acute febrile illness (AFI) surveillance. Am J Trop Med Hyg. 2018;99(4):285–6.

520. Das B, Pany A, Das A, Chheda P, Singh H, Mahida M, et al. Epidemiology of H1N1 pandemic in metro cities of India. J Mol Diagn. 2010;12(6):875–6.

521. Adisasmito W. Epidemiology of human avian influenza in Indonesia, 2005-2009: A descriptive analysis. Medical Journal of Indonesia. 2010;19(1):64–70.

522. Dapat C, Saito R, Kyaw Y, Naito M, Hasegawa G, Suzuki Y, et al. Epidemiology of human influenza A and B viruses in myanmar from 2005 to 2007. Intervirology. 2009;52(6):310–20.

523. Nandhini G, Sujatha S. Epidemiology of influenza viruses from 2009 to 2013 - A sentinel surveillance report from Union territory of Puducherry, India. Asian Pacific Journal of Tropical Medicine. 2015;8(9):718–23.

524. Shrikhande S, Bhoyar SK, Tenpe SH, Deogade NG. Epidemiology of pandemic H1N1 strains in a tertiary hospital of Maharashtra. Indian journal of public health. 2012;56(3):242–4.

525. Prachayangprecha S, Makkoch J, Suwannakarn K, Vichaiwattana P, Korkong S, Theamboonlers A, et al. Epidemiology of seasonal influenza in Bangkok between 2009 and 2012. Journal of Infection in Developing Countries. 2013;7(10):734–40.

526. Krishnan A, Kumar R, Broor S, Gopal G, Saha S, Amarchand R, et al. Epidemiology of viral acute lower respiratory infections in a community-based cohort of rural north Indian children. Journal of Global Health [Internet]. 2019;9(1). Available from: https://www.scopus.com/inward/record.uri?eid=2-s2.0-85066876578&doi=10.7189%2fjogh.09.010433&partnerID=40&md5=382ce1570d78562e7c5b75e515369c37

527. Sabat J, Subhadra S, Dwibedi B, Palo SK, Pati S. Epidemiology of viral respiratory infection in Odisha, India. VirusDisease. 2019;30(1):141–2.

528. Virmani N, Bera BC, Singh BK, Shanmugasundaram K, Gulati BR, Barua S, et al. Equine influenza outbreak in India (2008-09): Virus isolation, sero-epidemiology and phylogenetic analysis of HA gene. Veterinary Microbiology. 2010;143(2):224–37.

529. Waghmare SP, Mode SG, Kolte AY, Babhulkar N, Vyavahare SH, Patel A. Equine influenza: An overview. Veterinary World. 2010;3(4):194–7.

530. Setiawaty V, Pangesti KNA, Sampurno OD. Establishing a laboratory network of influenza diagnosis in Indonesia: An experience from the avian flu (H5N1) outbreak. Clinical Epidemiology. 2012;4(1):209–12.

531. Chen H, Smith GJD, Li KS, Wang J, Fan XH, Rayner JM, et al. Establishment of multiple sublineages of H5N1 influenza virus in Asia: implications for pandemic control. Proc Natl Acad Sci U S A. 2006;103(8):2845–50.

532. Suhardono M, Ugiyadi D, Nurnaeni I, Emelia I. Establishment of pandemic influenza vaccine production capacity at Bio Farma, Indonesia. Vaccine. 2011;29:A22–5.

533. Dawood FS, Iuliano AD, Reed C, Meltzer MI, Shay DK, Cheng PY, et al. Estimated global mortality associated with the first 12 months of 2009 pandemic influenza A H1N1 virus circulation: A modelling study. The Lancet Infectious Diseases. 2012;12(9):687–95.

534. Susilarini NK, Haryanto E, Praptiningsih CY, Mangiri A, Kipuw N, Tarya I, et al. Estimated incidence of influenza-associated severe acute respiratory infections in Indonesia, 2013-2016. Influenza and other Respiratory Viruses. 2018;12(1):81–7.

535. Iuliano AD, Roguski KM, Chang HH, Muscatello DJ, Palekar R, Tempia S, et al. Estimates of global seasonal influenza-associated respiratory mortality: a modelling study. The Lancet. 2018;391(10127):1285–300.

536. Ahmed M, Aleem MA, Roguski K, Abedin J, Islam A, Alam KF, et al. Estimates of seasonal influenza-associated mortality in Bangladesh, 2010-2012. Influenza and other Respiratory Viruses. 2018;12(1):65–71.

537. Van Kerkhove MD, Hirve S, Koukounari A, Mounts AW, Allwinn R, Bandaranayake, et al. Estimating age-specific cumulative incidence for the 2009 influenza pandemic: A meta-analysis of A(H1N1)pdm09 serological studies from 19 countries. Influenza and other Respiratory Viruses. 2013;7(5):872–86.

538. Marquetoux N, Paul M, Wongnarkpet S, Poolkhet C, Thanapongtharm W, Roger F, et al. Estimating spatial and temporal variations of the reproduction number for highly pathogenic avian influenza H5N1 epidemic in Thailand. Preventive Veterinary Medicine. 2012;106(2):143–51.

539. Ssematimba A, Okike I, Ahmed GM, Yamage M, Boender GJ, Hagenaars TJ, et al. Estimating the between-farm transmission rates for highly pathogenic avian influenza subtype H5N1 epidemics in Bangladesh between 2007 and 2013. Transboundary and Emerging Diseases. 2018;65(1):e127–34.

540. Walker PGT, Jost C, Ghani AC, Cauchemez S, Bett B, Azhar M, et al. Estimating the transmissibility of H5N1 and the effect of vaccination in Indonesia. Transboundary and Emerging Diseases. 2015;62(2):200–8.

541. Ram Purakayastha D, Vishnubhatla S, Rai SK, Broor S, Krishnan A. Estimation of burden of influenza among under-five children in India: A meta-analysis. Journal of Tropical Pediatrics. 2018;64(5):441–53.

542. Saha S, Gupta V, Rai SK, Krishnan A, Dawood FS, Lafond KE, et al. Estimation of community-level influenza-associated illness in a low resource rural setting in India. PLoS ONE. 2018;13(4):e0196495.

543. Moorthy M, Vijayakumar S, Sekhar D, Abraham AM, Samuel P, Verghese VP, et al. Estimation of the Burden of Pandemic(H1N1)2009 in Developing Countries: Experience from a Tertiary Care Center in South India. PLoS ONE. 2012;7(9):e41507.

544. Chuengsatiansup K. Ethnography of epidemiologic transition: Avian flu, global health politics and agro-industrial capitalism in Thailand. Anthropology and Medicine. 2008;15(1):53–9.

545. Das R, Singh M, Agarwal A, Ray P. Etiologic profile of micro-organisms causing severe pneumonia in children under five in India. Int J Infect Dis. 2014;21:293.

546. Luvira V, Silachamroon U, Piyaphanee W, Lawpoolsri S, Chierakul W, Leaungwutiwong P, et al. Etiologies of acute undifferentiated febrile illness in Bangkok, Thailand. American Journal of Tropical Medicine and Hygiene. 2019;100(3):622–9.

547. Aman AT, Wibawa T, Kosasih H, Asdie RH, Safitri I, Intansari US, et al. Etiologies of severe acute respiratory infection (SARI) and misdiagnosis of influenza in Indonesia, 2013-2016. Influenza and other Respiratory Viruses. 2021;15(1):34–44.

548. Robinson M, Gupta A, Balasubramanian U, Raichur P, Marbaniang I, Kanade S, et al. Etiology of acute febrile illness among hospitalized adults and children at a public tertiary-care center in Pune, India. Open Forum Infect Dis. 2016;3.

549. Upadhyay BP, Banjara MR, Shrestha RK, Tashiro M, Ghimire P. Etiology of coinfections in children with influenza during 2015/16 winter season in Nepal. International Journal of Microbiology [Internet]. 2018;2018. Available from: https://www.scopus.com/inward/record.uri?eid=2-s2.0-85062588357&doi=10.1155%2f2018%2f8945142&partnerID=40&md5=9a65592f5fae590287572b1eb0dfd8b6

550. Rahaman MR, Alroy KA, van Beneden CA, Friedman MS, Kennedy ED, Rahman M, et al. Etiology of severe acute respiratory infections, Bangladesh, 2017. Emerging Infectious Diseases. 2021;27(1):324–6.

551. Wawegama NK, Tarigan S, Indriani R, Selleck P, Adjid RMA, Syafriati T, et al. Evaluation of a conserved HA274–288 epitope to detect antibodies to highly pathogenic avian influenza virus H5N1 in Indonesian commercial poultry. Avian Pathology. 2016;45(4):478–92.

552. Sawatwong P, Whistler T, Chittaganpitch M, Paveenkittiporn W, Baggett HC, Olsen SJ. Evaluation of a molecular diagnostic platform for simultaneous detection of multiple respiratory pathogens in Thailand. Am J Trop Med Hyg. 2013;89(5):124.

553. Vangara H, Kalawat U, Dakshinamurthy S, Kumar BS, Badur M, Madhusudan M, et al. Evaluation of a new rapid antigen detection test for the diagnosis of influenza virus infection in a tertiary care Hospital of Southern India. J Clin Diagn Res. 2021;15(4):DC24–9.

554. Saha P, Chawla-Sarkar M, Mitra S, Malbari K, Kanyalkar M. Evaluation of anti-viral activity of synthesized molecules on Influenza A pandemic H1N1 strains circulating in eastern India during 2017-2019. Int J Infect Dis. 2020;101:514–5.

555. Ghoke SS, Sood R, Kumar N, Pateriya AK, Bhatia S, Mishra A, et al. Evaluation of antiviral activity of Ocimum sanctum and Acacia arabica leaves extracts against H9N2 virus using embryonated chicken egg model. BMC Complementary and Alternative Medicine [Internet]. 2018;18(1). Available from: https://www.scopus.com/inward/record.uri?eid=2-s2.0-85048040800&doi=10.1186%2fs12906-018-2238-1&partnerID=40&md5=fa48842144fcbca96255ce56bfc75fe7

556. Rahman MS, Malek MA, Islam MA, Uddin MJ, Ahasan MS, Chakrabartty A, et al. Evaluation of avian influenza and Newcastle disease virus detection kit using field samples from domestic and semi-domestic birds. Journal of Veterinary Clinics. 2012;29(4):309–14.

557. Saha S, Pandey BG, Choudekar A, Krishnan A, Gerber SI, Rai SK, et al. Evaluation of case definitions for estimation of respiratory syncytial virus associated hospitalizations among children in a rural community of northern India. Journal of Global Health [Internet]. 2015;5(2). Available from: https://www.scopus.com/inward/record.uri?eid=2-s2.0-84976313641&doi=10.7189%2fjogh.05.020419&partnerID=40&md5=a532c09f8f093e649c70e8ed23165efe

558. Ranjan P, Kumari A, Das R, Gupta L, Singh S, Yadav M. Evaluation of clinical features scoring system as screening tool for influenza A (H1N1) in epidemic situations. Journal of Postgraduate Medicine. 2012;58(4):265–9.

559. Narayan VV, Iuliano AD, Roguski K, Haldar P, Saha S, Sreenivas V, et al. Evaluation of data sources and approaches for estimation of influenza-associated mortality in India. Influenza and other Respiratory Viruses. 2018;12(1):72–80.

560. Chinnawirotpisan P, Manasatienkij W, Joonlasak K, Klunhthong C, Huang A, Macareo LR, et al. Evaluation of next generation sequencing and bioinformatics pipeline for pathogen identification used at the department of virology, armed forces research institute of medical sciences (AFRIMS), Bangkok, Thailand. Am J Trop Med Hyg. 2018;99(4):281.

561. Rahman M, Mangtani P, Uyeki TM, Cardwell JM, Torremorell M, Islam A, et al. Evaluation of potential risk of transmission of avian influenza A viruses at live bird markets in response to unusual crow die-offs in Bangladesh. Influenza and other Respiratory Viruses. 2020;14(3):349–52.

562. Makkoch J, Prachayangprecha S, Vichaiwattana P, Suwannakarn K, Theamboonlers A, Poovorawan Y. Evaluation of rapid influenza virus tests in patients with influenza-like illness in Thailand. Clin Lab. 2012;58(9):905–10.

563. Bassat Q, Jullien S, Sharma R, Tshering K, Munoz C. Evaluation of the epidemiology, aetiology and clinical presentation of acute lower respiratory infections among children under five years of age admitted to the Jigme Dorji Wangchuck national referral hospital in Thimphu, Bhutan. Am J Trop Med Hyg. 2018;99(4):184.

564. Loth L, Prijono WB, Wibawa H, Usman TB. Evaluation of two avian influenza type A rapid antigen tests under Indonesian field conditions. Journal of Veterinary Diagnostic Investigation. 2008;20(5):642–4.

565. Muraduzzaman AKM, Khan MH, Sultana S, Alam AN, Akram A, Shirin T, et al. Event based surveillance of middle east respiratory syndrome coronavirus (MERS-CoV) in Bangladesh among pilgrims and travelers from the middle east: An update for the period 2013-2016. PLoS ONE. 2018;13(1):e0189914.

566. Cattoli G, Fusaro A, Monne I, Coven F, Joannis T, El-Hamid HSA, et al. Evidence for differing evolutionary dynamics of A/H5N1 viruses among countries applying or not applying avian influenza vaccination in poultry. Vaccine. 2011;29(50):9368–75.

567. Tewawong N, Suntronwong N, Korkong S, Theamboonlers A, Vongpunsawad S, Poovorawan Y. Evidence for influenza B virus lineage shifts and reassortants circulating in Thailand in 2014–2016. Infection, Genetics and Evolution. 2017;47:35–40.

568. Khuntirat BP, Yoon IK, Blair PJ, Krueger WS, Chittaganpitch M, Putnam SD, et al. Evidence for subclinical avian influenza virus infections among rural thai villagers. Clinical Infectious Diseases. 2011;53(8):e107–16.

569. Kuanprasert S, Apichartpikul N, Chuenkitmongkol S, Wiangosot W, Topaiboon P, Sukonthasarn A. Evidence of influenza or influenza-like-illness preceding acute coronary syndrome. Southeast Asian J Trop Med Public Health. 2008;39(6):1040–4.

570. Tangwangvivat R, Chanvatik S, Charoenkul K, Chaiyawong S, Janethanakit T, Tuanudom R, et al. Evidence of pandemic H1N1 influenza exposure in dogs and cats, Thailand: A serological survey. Zoonoses and Public Health. 2019;66(3):349–53.

571. Leung YHC, Poon LLM, Smith GJD, Vijaykrishna D, Cheung CL, Rayner JM, et al. Evolution and adaptation of H5N1 influenza virus in avian and human hosts in Indonesia and Vietnam. Virology. 2006;350(2):258–68.

572. Evolution of H5N1 avian influenza viruses in Asia. Emerg Infect Dis. 2005;11(10):1515–21.

573. Neumann G, Green MA, Macken CA. Evolution of highly pathogenic avian H5N1 influenza viruses and the emergence of dominant variants. J Gen Virol. 2010;91:1984–95.

574. Tewawong N, Vichiwattana P, Korkong S, Klinfueng S, Suntronwong N, Thongmee T, et al. Evolution of the neuraminidase gene of seasonal influenza A and B viruses in Thailand between 2010 and 2015. PLoS ONE. 2017;12(4):e0175655.

575. Lam TTY, Hon CC, Wong RTY, Yip CW, Zeng F, Leung FCC, et al. Evolutionary and transmission dynamics of reassortant H5N1 influenza virus in Indonesia. PLoS Pathog. 2008;4(8):e1000130.

576. Mullick J, Cherian SS, Potdar VA, Chadha MS, Mishra AC. Evolutionary dynamics of the influenza A pandemic (H1N1) 2009 virus with emphasis on Indian isolates: Evidence for adaptive evolution in the HA gene. Infection, Genetics and Evolution. 2011;11(5):997–1005.

577. Mathur R, Adlakha N. Evolutionary network to predict the reassortment of avian-human A/H5N1 influenza virus in India. Trends Bioinformatics. 2013;6(1):1–9.

578. Agustiningsih A, Trimarsanto H, Restuadi R, Made Artika I, Hellard M, Muljono DH. Evolutionary study and phylodynamic pattern of human influenza A/H3N2 virus in Indonesia from 2008 to 2010. PLoS ONE. 2018;13(8):e0201427.

579. Jones S, Prasad R, Chirundodh DV, Pillai RM, Nelson-Sathi S, Rayen S, et al. Evolutionary, genetic, structural characterization and its functional implications for the influenza A (H1N1) infection outbreak in India from 2009 to 2017. Sci Rep. 2019;9(1):14690.

580. Siswoyo H, Permana M, Larasati RP, Farid J, Suryadi A, Sedyaningsih ER. EWORS: using a syndromic-based surveillance tool for disease outbreak detection in Indonesia. BMC Proc. 2008;2:S3.

581. Kumar S, Quinn SC. Existing health inequalities in India: informing preparedness planning for an influenza pandemic. Health Policy Plan. 2012;27(6):516–26.

582. Ruscio BA, Hotez P. Expanding global and national influenza vaccine systems to match the COVID-19 pandemic response. Vaccine. 2020;38(50):7880–2.

583. Islam QT, Kahhar A, Arif SM, Ahasan HN, Siddiqui MMR, Mahbub MS, et al. Experience of pandemic influenza a (H1N1) 2009 at Dhaka Medical College Hospital. Journal of Medicine. 2010;11(2):119–23.

584. Townsend MB, Dawson ED, Mehlmann M, Smagala JA, Dankbar DM, Moore CL, et al. Experimental evaluation of the FluChip diagnostic microarray for influenza virus surveillance. J Clin Microbiol. 2006;44(8):2863–71.

585. Charoenvisal N, Keawcharoen J, Sreta D, Tantawet S, Jittimanee S, Arunorat J, et al. Experimental infection with a Thai reassortant swine influenza virus of pandemic H1N1 origin induced disease. Virology Journal [Internet]. 2013;10. Available from: https://www.scopus.com/inward/record.uri?eid=2-s2.0-84874936696&doi=10.1186%2f1743-422X-10-88&partnerID=40&md5=55776c3f42a0cee4e82cdc6504ecd98b

586. Das BR, Kumar M, Murugkar HV, Nagarajan S, Senthil Kumar D, Kalaiyarasu S, et al. Experimental inoculation of a crow derived influenza A (H5N1) virus in chickens and its pathological and genetic characterization. Indian Journal of Animal Sciences. 2016;86(3):238–42.

587. Wibawa H, Bingham J, Nuradji H, Lowther S, Payne J, Harper J, et al. Experimentally infected domestic ducks show efficient transmission of Indonesian H5N1 highly pathogenic avian influenza virus, but lack persistent viral shedding. PLoS One. 2014;9(1):e83417.

588. Wen F, Bedford T, Cobey S. Explaining the geographical origins of seasonal influenza A (H3N2). Proc Biol Sci. 2016;283(1838).

589. Wibawa H, Karo-Karo D, Pribadi ES, Bouma A, Bodewes R, Vernooij H, et al. Exploring contacts facilitating transmission of influenza A(H5N1) virus between poultry farms in West Java, Indonesia: A major role for backyard farms? Preventive Veterinary Medicine. 2018;156:8–15.

590. Poolkhet C, Makita K, Thongratsakul S, Leelehapongsathon K. Exponential random graph models to evaluate the movement of backyard chickens after the avian influenza crisis in 2004–2005, Thailand. Preventive Veterinary Medicine. 2018;158:71–7.

591. Aggarwal N, Dwarakanathan V, Gautam N, Ray A. Facemasks for prevention of viral respiratory infections in community settings: A systematic review and meta-analysis. Indian journal of public health. 2020;64:S192–200.

592. Davis BM, Black D. Facilitators and barriers to adult vaccination in south east asia and Latin America. Value Health. 2017;20(9):A934.

593. Visitsunthorn N, Lilitwat W, Jirapongsananuruk O, Vichyanond P. Factors affecting readmission for acute asthmatic attacks in children. Asian Pacific Journal of Allergy and Immunology. 2013;31(2):138–41.

594. Suresh PS, Thejaswini V, Rajan T. Factors associated with 2009 pandemic influenza A (H1N1) vaccination acceptance among university students from India during the post-pandemic phase. BMC Infectious Diseases [Internet]. 2011;11. Available from: https://www.scopus.com/inward/record.uri?eid=2-s2.0-79960882845&doi=10.1186%2f1471-2334-11-205&partnerID=40&md5=9ee886133524411dc8c712d2839f86a9

595. Kandun IN, Tresnaningsih E, Purba WH, Soni E, Septiawati C, Setiawati T, et al. Factors associated with case fatality of human H5N1 virus infections in Indonesia: a case series. Lancet. 2008;372(9640):744–9.

596. Gupta SD, Fournié G, Hoque MA, Henning J. Factors influencing chicken farmers’ decisions to implement prevention and control measures to reduce avian influenza virus spread under endemic conditions. Transboundary and Emerging Diseases. 2021;68(1):194–207.

597. Songserm T, Amonsin A, Jam-on R, Sae-Heng N, Pariyothorn N, Payungporn S, et al. Fatal avian influenza A H5N1 in a dog. Emerg Infect Dis. 2006;12(11):1744–7.

598. Heldens JGM, Glansbeek HL, Hilgers LAT, Haenen B, Stittelaar KJ, Osterhaus ADME, et al. Feasibility of single-shot H5N1 influenza vaccine in ferrets, macaques and rabbits. Vaccine. 2010;28(51):8125–31.

599. Philpott EK, Englund JA, Katz J, Tielsch J, Khatry S, LeClerq SC, et al. Febrile rhinovirus illness during pregnancy is associated with low birth weight in Nepal. Open Forum Infectious Diseases [Internet]. 2017;4(2). Available from: https://www.scopus.com/inward/record.uri?eid=2-s2.0-85031902864&doi=10.1093%2fofid%2fofx073&partnerID=40&md5=9a7d509307567a6c1ddc182c42cd1f18

600. Tarigan S, Indriani R, Sumarningsih S, Wibowo MH, Artanto S, Asmara W, et al. Field effectiveness of highly pathogenic avian influenza H5N1 vaccination in commercial layers in Indonesia. PLoS ONE. 2018;13(1):e0190947.

601. Simmerman JM, Chittaganpitch M, Erdman D, Sawatwong P, Uyeki TM, Dowell SF. Field performance and new uses of rapid influenza testing in Thailand. Int J Infect Dis. 2007;11(2):166–71.

602. Bouma A, Muljono AT, Jatikusumah A, Nell AJ, Mudjiartiningsih S, Dharmayanti I, et al. Field trial for assessment of avian influenza vaccination effectiveness in Indonesia. OIE Revue Scientifique et Technique. 2008;27(3):633–42.

603. Simmerman JM, Suntarattiwong P, Levy J, Jarman RG, Kaewchana S, Gibbons RV, et al. Findings from a household randomized controlled trial of hand washing and face masks to reduce influenza transmission in Bangkok, Thailand. Influenza and other Respiratory Viruses. 2011;5(4):256–67.

604. Jongcherdchootrakul K, Henderson AK, Iamsirithaworn S, Modchang C, Siriarayapon P. First pandemic A (H1N1) pdm09 outbreak in a private school, Bangkok, Thailand, June 2009. Journal of the Medical Association of Thailand. 2014;97:S145–52.

605. Lekcharoensuk P, Nanakorn J, Wajjwalku W, Webby R, Chumsing W. First whole genome characterization of swine influenza virus subtype H3N2 in Thailand. Veterinary Microbiology. 2010;145(3):230–44.

606. Gilbert M, Newman SH, Takekawa JY, Loth L, Subba Rao MV, Slingenbergh J, et al. Flying over an infected landscape: Distribution of highly pathogenic avian influenza H5N1 risk in South Asia and satellite tracking of wild waterfowl. EcoHealth. 2010;7(4):448–58.

607. Rangarajan P, Mody SK, Marathe M. Forecasting dengue and influenza incidences using a sparse representation of Google trends, electronic health records, and time series data. PLoS Comput Biol. 2019;15(11):e1007518.

608. Paul S, Sahoo J. Four new vaccines for routine immunization in India: what about hemophilus influenza B and pneumococcal vaccine? J Family Med Prim Care. 2015;4(1):9–12.

609. Seetoh T, Liverani M, Coker R. Framing risk in pandemic influenza policy and control. Global Public Health. 2012;7(7):717–30.

610. Chaitaweesub P, Parakamawongsa T, Premashthira S, Tiensin T, Kalpravidh W, Wagner H, et al. Free-grazing ducks and highly pathogenic avian influenza, Thailand. Emerg Infect Dis. 2006;12(2):227–34.

611. Roy Mukherjee T, Mukherjee A, Mullick S, Chawla-Sarkar M. Full genome analysis and characterization of influenza C virus identified in Eastern India. Infection, Genetics and Evolution. 2013;16:419–25.

612. Zaraket H, Kondo H, Hibino A, Yagami R, Odagiri T, Takemae N, et al. Full genome characterization of human influenza A/H3N2 isolates from asian countries reveals a rare amantadine resistance-conferring mutation and novel PB1-F2 polymorphisms. Frontiers in Microbiology [Internet]. 2016;7. Available from: https://www.scopus.com/inward/record.uri?eid=2-s2.0-84964317865&doi=10.3389%2ffmicb.2016.00262&partnerID=40&md5=13a9f978c1276df821a9a59a68e22977

613. Mukherjee TR, Agrawal AS, Chakrabarti S, Chawla-Sarkar M. Full genomic analysis of an influenza A (H1N2) virus identified during 2009 pandemic in Eastern India: Evidence of reassortment event between co-circulating A(H1N1)pdm09 and A/Brisbane/10/2007-like H3N2 strains. Virology Journal [Internet]. 2012;9. Available from: https://www.scopus.com/inward/record.uri?eid=2-s2.0-84874108210&doi=10.1186%2f1743-422X-9-233&partnerID=40&md5=fe8adb387ea9b850427aeb925f9ae24e

614. Parvin R, Heenemann K, Halami MY, Chowdhury EH, Islam MR, Vahlenkamp TW. Full-genome analysis of avian influenza virus H9N2 from Bangladesh reveals internal gene reassortments with two distinct highly pathogenic avian influenza viruses. Archives of Virology. 2014;159(7):1651–61.

615. DeFraites RF, Chambers WC. Gaining experience with military medical situational awareness and geographic information systems in a simulated influenza epidemic. Mil Med. 2007;172(10):1071–6.

616. Chamlagain D, Aryal A, Pokhrel KN, Poudel KN, Kattel B, Kakkar M, et al. Gaps in the control of zoonoses in Nepal. Trop Med Int Health. 2017;22:146–7.

617. Kumar D, Gupta G, Jhamb U. Gastrointestinal symptoms among hospitalized children admitted with H1N1 infection: A report from a tertiary hospital in north india. Gut. 2019;68:A61.

618. Poonsuk S, Sangthong P, Petcharat N, Lekcharoensuk P. Genesis and genetic constellations of swine influenza viruses in Thailand. Veterinary Microbiology. 2013;167(3):314–26.

619. Li KS, Guan Y, Wang J, Smith GJD, Xu KM, Duan L, et al. Genesis of a highly pathogenic and potentially pandemic H5N1 influenza virus in eastern Asia. Nature. 2004;430(6996):209–13.

620. Shanmuganatham K, Feeroz MM, Jones-Engel L, Walker D, Alam SMR, Hasan MK, et al. Genesis of avian influenza H9N2 in Bangladesh. Emerging Microbes and Infections [Internet]. 2014;3(12). Available from: https://www.scopus.com/inward/record.uri?eid=2-s2.0-84927919568&doi=10.1038%2femi.2014.88&partnerID=40&md5=d84039febbd54e12031c96484b751bda

621. El-Shesheny R, Barman S, Feeroz MM, Hasan MK, Jones-Engel L, Franks J, et al. Genesis of influenza A(H5N8) viruses. Emerging Infectious Diseases. 2017;23(8):1368–71.

622. Sanjay RE, Sabeena S, Robin S, Shaji JT, Jayakrishnan MP, Suresh EKK, et al. Genetic analysis of Enterovirus D68 associated with pneumonia in children from South India. Journal of Medical Microbiology [Internet]. 2021;70(5). Available from: https://www.scopus.com/inward/record.uri?eid=2-s2.0-85106639446&doi=10.1099%2fJMM.0.001356&partnerID=40&md5=94b16767c84f7092052a8378196a0a28

623. Tosh C, Nagarajan S, Behera P, Rajukumar K, Purohit K, Kamal RP, et al. Genetic analysis of H9N2 avian influenza viruses isolated from India. Archives of Virology. 2008;153(8):1433–9.

624. Gunasekaran P, Krishnasamy K, Arunagiri K, Sambasivam M, Lakshmipathy M, Arunpon, et al. Genetic analysis of HA gene of pandemic H1N1 2009 influenza viruses circulating in India. Indian Journal of Medical Microbiology. 2012;30(3):346–9.

625. Amonsin A, Pariyothorn N, Songserm T, Jam-on R, Sae-Heng N, Chutinimitkul S, et al. Genetic analysis of influenza a virus (H5N1) derived from domestic cat and dog in Thailand. Arch Virol. 2007;152(10):1925–33.

626. Jagadesh A, Salam AAA, Zadeh VR, Arunkumar G. Genetic analysis of neuraminidase gene of influenza A(H1N1)pdm09 virus circulating in Southwest India from 2009 to 2012. Journal of Medical Virology. 2017;89(2):202–12.

627. Kumar S, Gadhoke I, Khare S, Rawat DS, Chauhan LS, Rai A. Genetic analysis of the complete NS gene of novel pandemic influenza A H1N1 2009 virus strains circulating in India during 2010-11. Int J Infect Dis. 2012;16:e92.

628. Virmani N, Bera BC, Shanumugasundaram K, Singh BK, Gulati BR, Singh RK, et al. Genetic analysis of the matrix and non-structural genes of equine influenza virus (H3N8) from epizootic of 2008-2009 in India. Veterinary Microbiology. 2011;152(1):169–75.

629. Bera BC, Virmani N, Shanmugasundaram K, Vaid RK, Singh BK, Gulati BR, et al. Genetic analysis of the neuraminidase (NA) gene of equine influenza virus (H3N8) from epizootic of 2008-2009 in India. Indian Journal of Virology. 2013;24(2):256–64.

630. Komadina N, Iannello P, Roque V, Rimando-Magalong J, Bomasang E, Rivera M, et al. Genetic analysis of two influenza A (H1) swine viruses isolated from humans in Thailand and the Philippines. Virus Genes. 2007;35(2):161–5.

631. Bhat S, Bhatia S, Pillai AS, Sood R, Singh VK, Shrivas OP, et al. Genetic and antigenic characterization of H5N1 viruses of clade 2.3.2.1 isolated in India. Microbial Pathogenesis. 2015;88:87–93.

632. Tewawong N, Suntronwong N, Vichiwattana P, Vongpunsawad S, Theamboonlers A, Poovorawan Y. Genetic and antigenic characterization of hemagglutinin of influenza A/H3N2 virus from the 2015 season in Thailand. Virus Genes. 2016;52(5):711–5.

633. Pawestri HA, Nugraha AA, Han AX, Pratiwi E, Parker E, Richard M, et al. Genetic and antigenic characterization of influenza A/H5N1 viruses isolated from patients in Indonesia, 2008–2015. Virus Genes. 2020;56(4):417–29.

634. Suntronwong N, Klinfueng S, Vichiwattana P, Korkong S, Thongmee T, Vongpunsawad S, et al. Genetic and antigenic divergence in the influenza A(H3N2) virus circulating between 2016 and 2017 in Thailand. PLoS ONE. 2017;12(12):e0189511.

635. Mine J, Abe H, Parchariyanon S, Boonpornprasert P, Ubonyaem N, Nuansrichay B, et al. Genetic and antigenic dynamics of influenza A viruses of swine on pig farms in Thailand. Archives of Virology. 2019;164(2):457–72.

636. Jain A, Dangi T, Jain B, Singh AK, Singh JV, Kumar R. Genetic changes in influenza A(H3N2) viruses circulating during 2011 to 2013 in northern India (Lucknow). Journal of Medical Virology. 2015;87(8):1268–75.

637. Biswas D, Dutta M, Sarmah K, Yadav K, Buragohain M, Sarma K, et al. Genetic characterisation of influenza A(H1N1)pdm09 viruses circulating in Assam, Northeast India during 2009-2015. Indian Journal of Medical Microbiology. 2019;37(1):42–9.

638. Franks J, Marathe BM, Krauss S, McKenzie P, Webby RJ, Webster RG, et al. Genetic characterization and pathogenic potential of H10 avian influenza viruses isolated from live poultry markets in Bangladesh. Sci Rep. 2018;8(1):10693.

639. Lapkuntod J, Tantilertcharoen R, Boonyapisitsopa S, Bunpapong N, Wisedchanwet T, Amonsin A, et al. Genetic characterization of 2008 reassortant influenza A virus (H5N1), Thailand. Virol J. 2010;7:233.

640. Kim JK, Nam JH, Lyoo KS, Moon H, Na W, Song EJ, et al. Genetic Characterization of an Ancestral Strain of the Avian-Origin H3N2 Canine Influenza Virus Currently Circulating in East Asia. J Microbiol Biotechnol. 2016;26(6):1109–14.

641. Wisedchanwet T, Wongphatcharachai M, Boonyapisitsopa S, Bunpapong N, Kitikoon P, Amonsin A. Genetic characterization of avian influenza subtype H4N6 and H4N9 from live bird market, Thailand. Virology Journal [Internet]. 2011;8. Available from: https://www.scopus.com/inward/record.uri?eid=2-s2.0-79953107380&doi=10.1186%2f1743-422X-8-131&partnerID=40&md5=bb61d079aeb9b0bb35913683cb6ae74d

642. Bunpapong N, Nonthabenjawan N, Chaiwong S, Tangwangvivat R, Boonyapisitsopa S, Jairak W, et al. Genetic characterization of canine influenza A virus (H3N2) in Thailand. Virus Genes. 2014;48(1):56–63.

643. Mukherjee A, Nayak MK, Chawla-Sarkar M, Dutta S, Panda S, Satpathi BR. Genetic characterization of circulating 2015 A(H1N1)pdm09 influenza viruses from Eastern India. PLoS ONE. 2016;11(12):e0168464.

644. Agrawal AS, Sarkar M, Ghosh S, Roy T, Chakrabarti S, Lal R, et al. Genetic characterization of circulating seasonal Influenza A viruses (2005-2009) revealed introduction of oseltamivir resistant H1N1 strains during 2009 in eastern India. Infection, Genetics and Evolution. 2010;10(8):1188–98.

645. Dharmayanti NLPI, Hartawan R, Pudjiatmoko, Wibawa H, Hardiman, Balish A, et al. Genetic characterization of clade 2.3.2.1 avian influenza A(H5N1) viruses, Indonesia, 2012. Emerging Infectious Diseases. 2014;20(4):671–4.

646. Chutinimitkul S, Thippamom N, Damrongwatanapokin S, Payungporn S, Thanawongnuwech R, Amonsin A, et al. Genetic characterization of H1N1, H1N2 and H3N2 swine influenza virus in Thailand. Archives of Virology. 2008;153(6):1049–56.

647. Amonsin A, Thanawongnuwech R, Suradhat S, Pariyothorn N, Tantilertcharoen R, Payungporn S, et al. Genetic characterization of H5N1 influenza A viruses isolated from zoo tigers in Thailand. Virology. 2006;344(2):480–91.

648. Kholik, Indrasari S, Hayati RS, Nidom RV, Alamudi MY, Nidom CA, et al. Genetic characterization of H5N1 influenza viruses isolated from chickens in Indonesia in 2010. Virus Genes. 2012;44(3):459–65.

649. Jagadesh A, Krishnan A, Nair S, Sivadas S, Arunkumar G. Genetic characterization of hemagglutinin (HA) gene of influenza A viruses circulating in Southwest India during 2017 season. Virus Genes. 2019;55(4):458–64.

650. Parvin R, Kamal AHM, Haque ME, Chowdhury EH, Giasuddin M, Islam MR, et al. Genetic characterization of highly pathogenic H5N1 avian influenza virus from live migratory birds in Bangladesh. Virus Genes. 2014;49(3):438–48.

651. Gohil D, Kothari S, Shinde P, Meharunkar R, Warke R, Chowdhary A, et al. Genetic Characterization of Influenza A (H1N1) Pandemic 2009 Virus Isolates from Mumbai. Curr Microbiol. 2017;74(8):899–907.

652. Jairak W, Boonyapisitsopa S, Chaiyawong S, Nonthabenjawan N, Tangwangvivat R, Bunpapong N, et al. Genetic characterization of influenza A (H7N6) virus isolated from a live-bird market in Thailand. Arch Virol. 2016;161(5):1315–22.

653. Wisedchanwet T, Bunpapong N, Wongphatcharachai M, Nonthabenjawan N, Jairak W, Chaiyawong S, et al. Genetic characterization of influenza A virus subtype H7N1 isolated from quail, Thailand. Virus Genes. 2014;49(3):428–37.

654. Wongphatcharachai M, Wisedchanwet T, Lapkuntod J, Nonthabenjawan N, Jairak W, Amonsin A. Genetic characterization of influenza A virus subtype H12N1 isolated from a watercock and lesser whistling ducks in Thailand. Archives of Virology. 2012;157(6):1123–30.

655. Chaiyawong S, Boonyapisitsopa S, Jairak W, Nonthabenjawan N, Tangwangvivat R, Bunpapong N, et al. Genetic characterization of influenza A virus subtypes H1N3 and H1N9 isolated from free-grazing ducks in Thailand. Archives of Virology. 2016;161(10):2819–24.

656. Amonsin A, Pariyothorn N, Puranaveja S, Tantilertcharoen R, Suradhat S, Thanawongnuwech R, et al. Genetic characterization of influenza A viruses (H5N1) isolated from 3rd wave of Thailand AI outbreaks. Virus Res. 2006;122(1):194–9.

657. Manasatienkij W, Chinnawirotpaisan P, Kittichotirat W, Macareo LR, Ellison DW, Cheevadhanarak S, et al. Genetic characterization of influenza a(H3n2) viruses from vaccinated and unvaccinated children during thailand 2013 and 2014 influenza seasons. Southeast Asian Journal of Tropical Medicine and Public Health. 2019;50(1):101–19.

658. Suwannakhon N, Pookorn S, Sanguansermsri D, Chamnanpood C, Chamnanpood P, Wongvilairat R, et al. Genetic characterization of nonstructural genes of H5N1 avian influenza viruses isolated in Thailand in 2004-2005. Southeast Asian Journal of Tropical Medicine and Public Health. 2008;39(5):837–47.

659. Charoenvisal N, Keawcharoen J, Sreta D, Chaiyawong S, Nonthabenjawan N, Tantawet S, et al. Genetic characterization of Thai swine influenza viruses after the introduction of pandemic H1N1 2009. Virus Genes. 2013;47(1):75–85.

660. Potdar VA, Chadha MS, Jadhav SM, Mullick J, Cherian SS, Mishra AC. Genetic characterization of the influenza A pandemic (H1N1) 2009 virus isolates from India. PLoS ONE. 2010;5(3):e9693.

661. Welkers MRA, Pawestri HA, Fonville JM, Sampurno OD, Pater M, Holwerda M, et al. Genetic diversity and host adaptation of avian H5N1 influenza viruses during human infection. Emerging Microbes and Infections. 2019;8(1):262–71.

662. Mondal SP, Balasuriya UBR, Yamage M. Genetic Diversity and Phylogenetic Analysis of Highly Pathogenic Avian Influenza (HPAI) H5N1 Viruses Circulating in Bangladesh from 2007-2011. Transboundary and Emerging Diseases. 2013;60(6):481–91.

663. Mir MA, Lal RB, Sullender W, Singh Y, Garten R, Krishnan A, et al. Genetic diversity of HA1 domain of hemagglutinin gene of pandemic influenza H1N1pdm09 viruses in New Delhi, India. Journal of Medical Virology. 2012;84(3):386–93.

664. Nonthabenjawan N, Chanvatik S, Chaiyawong S, Jairak W, Boonyapisusopha S, Tuanudom R, et al. Genetic diversity of swine influenza viruses in Thai swine farms, 2011–2014. Virus Genes. 2015;50(2):221–30.

665. Takemae N, Parchariyanon S, Damrongwatanapokin S, Uchida Y, Ruttanapumma R, Watanabe C, et al. Genetic diversity of swine influenza viruses isolated from pigs during 2000 to 2005 in Thailand. Influenza and other Respiratory Viruses. 2008;2(5):181–9.

666. Dharmayanti NLPI, Hewajuli DA, Ratnawati A, Hartawan R. Genetic diversity of the H5N1 viruses in live bird markets, Indonesia. Journal of Veterinary Science [Internet]. 2020;21(4). Available from: https://www.scopus.com/inward/record.uri?eid=2-s2.0-85088885454&doi=10.4142%2fJVS.2020.21.E56&partnerID=40&md5=3f7e40966b47cb55ec18b012dccf183b

667. Kwon JH, Lee DH, Criado MF, Killmaster L, Ali MZ, Giasuddin M, et al. Genetic evolution and transmission dynamics of clade 2.3.2.1a highly pathogenic avian influenza A/H5N1 viruses in Bangladesh. Virus Evolution [Internet]. 2020;6(2). Available from: https://www.scopus.com/inward/record.uri?eid=2-s2.0-85096991160&doi=10.1093%2fve%2fveaa046&partnerID=40&md5=e0365a5ab1d5f9d9702d74ab4d1154e9

668. Nandhini P, Sistla S. Genetic sequencing of influenza A (H1N1) pdm09 isolates from South India, collected between 2011 and 2015 to detect mutations affecting virulence and resistance to oseltamivir. Indian Journal of Medical Microbiology. 2020;38(3):324–37.

669. Choudhary ML, Anand SP, Wadhwa BS, Chadha MS. Genetic variability of human respiratory syncytial virus in Pune, Western India. Infection, Genetics and Evolution. 2013;20:369–77.

670. Thippamom N, Sreta D, Kitikoon P, Thanawongnuwech R, Poovorawan Y, Theamboonlers A, et al. Genetic variations of nucleoprotein gene of influenza A viruses isolated from swine in Thailand. Virology Journal [Internet]. 2010;7. Available from: https://www.scopus.com/inward/record.uri?eid=2-s2.0-77955296783&doi=10.1186%2f1743-422X-7-185&partnerID=40&md5=6e3d52c5e46ff4c3489dbc27a079d126

671. Sarmah K, Borkakoty B, Sarma K, Hazarika R, Das PK, Jakharia A, et al. Genetic variations of the Hemagglutinin gene of Pandemic Influenza A (H1N1) viruses in Assam, India during 2016. 3 Biotech. 2018;8(10):408.

672. Gerloff NA, Zanders N, Balish A, Wentworth DE, Donis RO, Davis CT, et al. Genetically diverse low pathogenicity avian influenza A virus subtypes co-circulate among poultry in Bangladesh. PLoS ONE. 2016;11(3):e0152131.

673. Ranaware PB, Mishra A, Vijayakumar P, Gandhale PN, Kumar H, Kulkarni DD, et al. Genome Wide Host Gene Expression Analysis in Chicken Lungs Infected with Avian Influenza Viruses. PLoS One. 2016;11(4):e0153671.

674. Kumar A, Vijayakumar P, Gandhale PN, Ranaware PB, Kumar H, Kulkarni DD, et al. Genome-wide gene expression pattern underlying differential host response to high or low pathogenic H5N1 avian influenza virus in ducks. Acta Virologica. 2017;61(1):66–76.

675. Chaitaweesub P, Chotiprasatintara S, Chanachai K, Thanapongtham W, Tiensin T, Nielen M, et al. Geographic and temporal distribution of highly pathogenic avian influenza a virus (H5N1) in Thailand, 2004-2005: An overview. Avian Dis. 2007;51:182–8.

676. Martin V, De Simone L, Lubroth J. Geographic information systems applied to the international surveillance and control of transboundary animal diseases, a focus on highly pathogenic avian influenza. Vet Ital. 2007;43(3):437–50.

677. Pradana AA, Nurhayati N, Aziz MH, Kosasih H, Fahmi A, Karyana M. Geographical assessment of six most common infectious diseases in seven large cities in indonesia using geographical information systems. Am J Trop Med Hyg. 2018;99(4):479.

678. Li X, Zhang Z, Yu A, Ho SYW, Carr MJ, Zheng W, et al. Global and local persistence of influenza A(H5N1) virus. Emerg Infect Dis. 2014;20(8):1287–95.

679. Lafond KE, Porter RM, Whaley MJ, Suizan Z, Ran Z, Aleem MA, et al. Global burden of influenza-associated lower respiratory tract infections and hospitalizations among adults: A systematic review and meta-analysis. PLoS Medicine [Internet]. 2021;18(3). Available from: https://www.scopus.com/inward/record.uri?eid=2-s2.0-85102964049&doi=10.1371%2fJOURNAL.PMED.1003550&partnerID=40&md5=5c20b7b43bcc7fb66fbc668fb880a474

680. Bedford T, Riley S, Barr IG, Broor S, Chadha M, Cox NJ, et al. Global circulation patterns of seasonal influenza viruses vary with antigenic drift. Nature. 2015;523(7559):217–20.

681. Xu ZW, Li ZJ, Hu WB. Global dynamic spatiotemporal pattern of seasonal influenza since 2009 influenza pandemic. Infect Dis Poverty. 2020;9(1):2.

682. Lai S, Qin Y, Cowling BJ, Ren X, Wardrop NA, Gilbert M, et al. Global epidemiology of avian influenza A H5N1 virus infection in humans, 1997-2015: a systematic review of individual case data. Lancet Infect Dis. 2016;16(7):e108–18.

683. Wang X, Li Y, Mei X, Bushe E, Campbell H, Nair H. Global hospital admissions and in-hospital mortality associated with all-cause and virus-specific acute lower respiratory infections in children and adolescents aged 5-19 years between 1995 and 2019: a systematic review and modelling study. BMJ Glob Health. 2021;6(7).

684. Bedford T, Cobey S, Beerli P, Pascual M. Global migration dynamics underlie evolution and persistence of human influenza A (H3N2). PLoS Pathog. 2010;6(5):e1000918.

685. Lafond KE, Nair H, Rasooly MH, Valente F, Booy R, Rahman M, et al. Global Role and Burden of Influenza in Pediatric Respiratory Hospitalizations, 1982–2012: A Systematic Analysis. PLoS Medicine [Internet]. 2016;13(3). Available from: https://www.scopus.com/inward/record.uri?eid=2-s2.0-84962069655&doi=10.1371%2fjournal.pmed.1001977&partnerID=40&md5=5e441b9d3358825d464674f3d698c7f6

686. Cozza V, Campbell H, Chang HH, Iuliano AD, Paget J, Patel NN, et al. Global Seasonal Influenza Mortality Estimates: A Comparison of 3 Different Approaches. Am J Epidemiol. 2021;190(5):718–27.

687. Sun L, Ward MP, Li R, Xia C, Lynn H, Hu Y, et al. Global spatial risk pattern of highly pathogenic avian influenza H5N1 virus in wild birds: A knowledge-fusion based approach. Prev Vet Med. 2018;152:32–9.

688. Li R, Jiang Z, Xu B. Global spatiotemporal and genetic footprint of the H5N1 avian influenza virus. Int J Health Geogr. 2014;13:14.

689. Duggal A, Pinto R, Rubenfeld G, Fowler RA. Global Variability in Reported Mortality for Critical Illness during the 2009-10 Influenza A(H1N1) Pandemic: A Systematic Review and Meta-Regression to Guide Reporting of Outcomes during Disease Outbreaks. PLoS One. 2016;11(5):e0155044.

690. Mohammad F, Paul R, Nutankalva L, Narreddy S. H1N1 influenza pandemic from June 2010 to November 2010 at a single nodal centre in Hyderabad, India. Clin Microbiol Infect. 2011;17:S263.

691. Nadkar MY, Subramanian S, Ingole N. H1N1 influenza: An update. Journal of Association of Physicians of India. 2009;57(6):454–8.

692. Singh I, Munjal S, Kumar M, Jha M, Gambhir RS, Talukdar B. H1N1 Influenza: Assessment of knowledge and awareness of private dental health professionals of a Tricity. J Family Med Prim Care. 2019;8(7):2229–33.

693. Kadam P, Joshi V, Joshi P. H1N1 influenza: Experience of a level III pediatric intensive care unit in India. Pediatr Crit Care Med. 2011;12(3):A38.

694. Naik JD, Patel KA, Rajderkar SS, Bhoye KR. H1N1 Swine flu: An experience in a district of Western Maharashtra, India. International Journal of Collaborative Research on Internal Medicine and Public Health. 2012;4(12):1876–83.

695. Daniels P, Wiyono A, Sawitri E, Poermadjaja B, Sims LD, Mackenzie J.S., et al. H5N1 highly pathogenic avian influenza in Indonesia: Retrospective considerations. Curr Top Microbiol Immunol. 2013;365:171–84.

696. Eagles D, Siregar ES, Dung DH, Weaver J, Wong F, Daniels P. H5N1 highly pathogenic avian influenza in Southeast Asia. OIE Revue Scientifique et Technique. 2009;28(1):341–8.

697. Neumann G, Chen H, Gao GF, Shu Y, Kawaoka Y. H5N1 influenza viruses: outbreaks and biological properties. Cell Res. 2010;20(1):51–61.

698. Seiler P, Kercher L, Feeroz MM, Shanmuganatham K, Jones-Engel L, Turner J, et al. H9N2 influenza viruses from Bangladesh: Transmission in chicken and New World quail. Influenza and other Respiratory Viruses. 2018;12(6):814–7.

699. Vashishtha VM, Dogra V, Choudhury P, Thacker N, Gupta SG, Gupta SK. Haemophilus influenza type b disease and vaccination in India: knowledge, attitude and practices of paediatricians. WHO South East Asia J Public Health. 2013;2(2):101–5.

700. Samra T, Pawar M. Health care personnel and risk of H1N1-chemoprophylaxis with oseltamivir. Indian Journal of Pharmacology. 2012;44(6):754–8.

701. Worasathit R, Wattana W, Okanurak K, Songthap A, Dhitavat J, Pitisuttithum P. Health education and factors influencing acceptance of and willingness to pay for influenza vaccination among older adults. BMC Geriatrics [Internet]. 2015;15(1). Available from: https://www.scopus.com/inward/record.uri?eid=2-s2.0-84945254017&doi=10.1186%2fs12877-015-0137-6&partnerID=40&md5=f01021be378a97adbd502947d3702007

702. Krishnaprasad K, Manshani P, Karankumar J. Health outcome and safety assessment of a fixed dose combination of Amantadine, Paracetamol, Chlorpheniramine maleate, and Phenylephrine introduction in India: A prescription event monitoring study. Perspect Clin Res. 2012;3(2):62–5.

703. Pandey P, Lee K, Amatya B, Angelo KM, Shlim DR, Murphy H. Health problems in travellers to Nepal visiting CIWEC clinic in Kathmandu — A GeoSentinel analysis. Travel Medicine and Infectious Disease [Internet]. 2021;40. Available from: https://www.scopus.com/inward/record.uri?eid=2-s2.0-85101875905&doi=10.1016%2fj.tmaid.2021.101999&partnerID=40&md5=9bc9ceb75666c3acc1af7bdbc17ded46

704. Bhandari D, Pandey P. Health problems while working as a volunteer or humanitarian aid worker in post-earthquake Nepal. Journal of the Nepal Medical Association. 2018;56(211):691–5.

705. Kalki P, Thavorncharoensap M, Riewpaiboon A. Health seeking behavior and its determinants in Jaffna Sri Lanka. Pharmaceutical Sciences Asia. 2017;44(3):134–41.

706. Krumkamp R, Kretzschmar M, Rudge JW, Ahmad A, Hanvoravongchai P, Westenhoefer J, et al. Health service resource needs for pandemic influenza in developing countries: A linked transmission dynamics, interventions and resource demand model. Epidemiology and Infection. 2011;139(1):59–67.

707. Yasobant S, Bruchhausen W, Memon FZ, Saxena D, Falkenberg T. Health system contact and awareness of zoonotic diseases: Can it serve as one health entry point in the urban community of Ahmedabad, India? Yale J Biol Med. 2021;94(2):259–69.

708. Rudge JW, Hanvoravongchai P, Krumkamp R, Chavez I, Adisasmito W, Chau PN, et al. Health system resource gaps and associated mortality from pandemic influenza across six Asian territories. PLoS One. 2012;7(2):e31800.

709. Wahyuningrum Y. Healthcare utilization survey in East Jakarta and West Java province, Indonesia. Int J Infect Dis. 2012;16:e275.

710. Alladi CSH, Jagadesh A, Prabhu SG, Arunkumar G. Hemagglutination Inhibition Antibody Response Following Influenza A(H1N1)pdm09 Virus Natural Infection: A Cross-Sectional Study from Thirthahalli, Karnataka, India. Viral Immunology. 2019;32(5):230–3.

711. Oehadian A, Jusuf H, Pranggono E, Parwati I, Setiabudi D. Hematologic manifestation of avian influenza patients in hasan sadikin hospital. Acta medica Indonesiana. 2009;41(3):126–9.

712. Haura L, Warachit B, Makkoch J, Poovorawan Y. Hemoptysis in children with pandemic influenza H1N1 2009 infection. Southeast Asian Journal of Tropical Medicine and Public Health. 2009;40(6):1259–63.

713. Nurhayati, Wibawa H, Mahawan T, Zenal FC, Schoonman L, Pfeiffer CN, et al. Herd-Level Risk Factors for Swine Influenza (H1N1) Seropositivity in West Java and Banten Provinces of Indonesia (2016–2017). Frontiers in Veterinary Science [Internet]. 2020;7. Available from: https://www.scopus.com/inward/record.uri?eid=2-s2.0-85096698976&doi=10.3389%2ffvets.2020.544279&partnerID=40&md5=e02835d6334c4e07da1e8ca4abd847ba

714. Kiertiburanakul S, Malathum K, Watcharananan SP, Bunupuradah P, Piebpien P, Rujiraviroj U, et al. High coverage and safety of influenza A (H1N1) 2009 monovalent vaccination among health care personnel in Thailand. American Journal of Infection Control. 2011;39(6):525–8.

715. Bublot M, Manvell RJ, Shell W, Brown IH. High level of protection induced by two fowlpox vector vaccines against a highly pathogenic avian influenza H5N1 challenge in specific-pathogen-free chickens. Avian Dis. 2010;54(1):257–61.

716. Jain B, Singh AK, Dangi T, Agarwal A, Verma AK, Dwivedi M, et al. High prevalence of human metapneumovirus subtype B in cases presenting as severe acute respiratory illness: An experience at tertiary care hospital. Clinical Respiratory Journal. 2014;8(2):225–33.

717. Khuntirat B, Yoon IK, Chittaganpitch M, Krueger WS, Supawat K, Blair PJ, et al. High rate of A(H1N1)pdm09 infections among rural Thai villagers, 2009-2010. PLoS One. 2014;9(9):e106751.

718. Lipatov AS, Webster RG. Highly lethal H5N1 influenza virus in asia: Genesis and options for control. Discov Med. 2004;4(24):378–83.

719. Mon PP, Lapkuntod J, Maw MT, Nuansrichay B, Parchariyanon S, Tiensin T, et al. Highly pathogenic avian influenza (H5N1) in Myanmar, 2006-2010. Archives of Virology. 2012;157(11):2113–23.

720. Walsh MG, Mor SM, Hossain S. Highly pathogenic avian influenza (H5n1) landscape suitability varies by wetland habitats and the degree of interface between wild waterfowl and poultry in india. Viruses [Internet]. 2020;12(11). Available from: https://www.scopus.com/inward/record.uri?eid=2-s2.0-85096153384&doi=10.3390%2fv12111290&partnerID=40&md5=6f4f6af8230c4350bf33b57717a2cfb1

721. Nuradji H, Bingham J, Payne J, Harper J, Lowther S, Wibawa H, et al. Highly Pathogenic Avian Influenza (H5N1) Virus in Feathers: Tropism and Pathology of Virus-Infected Feathers of Infected Ducks and Chickens. Veterinary Pathology. 2017;54(2):226–33.

722. Karo-Karo D, Diyantoro, Pribadi ES, Sudirman FX, Kurniasih SW, Sukirman, et al. Highly pathogenic avian influenza a(H5n1) outbreaks in West Java Indonesia 2015–2016: Clinical manifestation and associated risk factors. Microorganisms [Internet]. 2019;7(9). Available from: https://www.scopus.com/inward/record.uri?eid=2-s2.0-85074275671&doi=10.3390%2fmicroorganisms7090327&partnerID=40&md5=c22e4e53c8838cd4687c64d0f406340a

723. Nasreen S, Khan SU, Luby SP, Gurley ES, Abedin J, Zaman RU, et al. Highly pathogenic avian influenza a(H5n1) virus infection among workers at live bird markets, Bangladesh, 2009–2010. Emerging Infectious Diseases. 2015;21(4):629–37.

724. Thiry E, Zicola A, Addie D, Egberink H, Hartmann K, Lutz H, et al. Highly pathogenic avian influenza H5N1 virus in cats and other carnivores. Vet Microbiol. 2007;122(1):25–31.

725. Chaitaweesub P, Hoonsuwan W, Buranathai C, Parakamawongsa T, Premashthira S, Nielen M, et al. Highly pathogenic avian influenza H5N1, Thailand, 2004. Emerg Infect Dis. 2005;11(11):1664–72.

726. Marinova-Petkova A, Franks J, Tenzin S, Dahal N, Dukpa K, Dorjee J, et al. Highly pathogenic reassortant avian influenza A(H5N1) virus clade 2.3.2.1a in Poultry, Bhutan. Emerging Infectious Diseases. 2016;22(12):2137–41.

727. Rafeek RAM, Divarathna MVM, Noordeen F. History and current trends in influenza virus infections with special reference to Sri Lanka. VirusDisease. 2017;28(3):225–32.

728. Mathie RT, Baitson ES, Frye J, Nayak C, Manchanda RK, Fisher P. Homeopathic treatment of patients with influenza-like illness during the 2009 A/H1N1 influenza pandemic in India. Homeopathy. 2013;102(3):187–92.

729. Singh AK, Jain B, Verma AK, Kumar A, Dangi T, Dwivedi M, et al. Hospital outbreak of human respiratory syncytial virus (HRSV) illness in immunocompromised hospitalized children during summer. Clinical Respiratory Journal. 2015;9(2):180–4.

730. Chudasama RK, Patel UV, Verma PB. Hospitalizations associated with 2009 influenza A (H1N1) and seasonal influenza in Saurashtra region, India. Journal of Infection in Developing Countries. 2010;4(12):834–41.

731. Guo F, Roy A, Wang R, Yang J, Zhang Z, Luo W, et al. Host Adaptive Evolution of Avian-Origin H3N2 Canine Influenza Virus. Frontiers in Microbiology [Internet]. 2021;12. Available from: https://www.scopus.com/inward/record.uri?eid=2-s2.0-85108963290&doi=10.3389%2ffmicb.2021.655228&partnerID=40&md5=60583440ef216820b618fb4fc1d64694

732. Hayashi T, Hiromoto Y, Chaichoune K, Patchimasiri T, Chakritbudsabong W, Prayoonwong N, et al. Host cytokine responses of pigeons infected with highly pathogenic Thai avian influenza viruses of subtype H5N1 isolated from wild birds. PLoS One. 2011;6(8):e23103.

733. Chatterjee P, Seth B, Biswas T. Hotspots of H1N1 influenza in India: analysis of reported cases and deaths (2010-2017). Trop Doct. 2020;50(2):166–9.

734. Weaver AM, Islam M, Khatun-e-Jannat K, Munir Sohel B, Ahmed M, Rahman AM, et al. Household-level risk factors for secondary influenza-like illness in a rural area of Bangladesh. Am J Trop Med Hyg. 2015;93(4):563.

735. Assi TM, Rookkapan K, Rajgopal J, Sornsrivichai V, Brown ST, Welling JS, et al. How influenza vaccination policy may affect vaccine logistics. Vaccine. 2012;30(30):4517–23.

736. Theamboonlers A, Duang-In A, Vichaiwattana P, Thongmee T, Poovorawan Y. Human adenovirus in patients with influenza-like illness and/or acute gastroenteritis in thailand, 2016. Southeast Asian Journal of Tropical Medicine and Public Health. 2019;50(2):229–39.

737. Eyanoer PC, Singhasivanon P, Kaewkungwal J, Apisarnthanarak A. Human avian influenza in Indonesia: Are they really clustered? Southeast Asian Journal of Tropical Medicine and Public Health. 2011;42(3):583–95.

738. Lu X, Peret T, Erdman D, Anderson LJ, Chittaganpitch M, Fischer J, et al. Human bocavirus: A novel parvovirus epidemiologically associated with pneumonia requiring hospitalization in Thailand. J Infect Dis. 2007;195(7):1038–45.

739. Chotpitayasunondh T, Lochindarat S, Srisan P, Ungchusak K, Hanshaoworakul W, Chunsuthiwat S, et al. Human disease from influenza A (H5N1), Thailand, 2004. Emerg Infect Dis. 2005;11(2):201–9.

740. Acharya KP, Acharya N, Phuyal S, Subramanya SH. Human infection with Avian influenza A virus in Nepal: requisite for timely management and preparedness. VirusDisease. 2020;31(3):244–8.

741. Adisasmito W, Aisyah DN, Aditama TY, Kusriastuti R, Trihono, Suwandono A, et al. Human influenza A H5N1 in Indonesia: Health care service-associated delays in treatment initiation. BMC Public Health [Internet]. 2013;13(1). Available from: https://www.scopus.com/inward/record.uri?eid=2-s2.0-84878710967&doi=10.1186%2f1471-2458-13-571&partnerID=40&md5=fc6ac0b8f282dd8fd8287d93ac758665

742. Lenahan JL, Englund JA, Katz J, Kuypers J, Wald A, Magaret A, et al. Human metapneumovirus and other respiratory viral infections during pregnancy and birth, Nepal. Emerging Infectious Diseases. 2017;23(8):1341–9.

743. Principi N, Bosis S, Esposito S. Human metapneumovirus in paediatric patients. Clin Microbiol Infect. 2006;12(4):301–8.

744. Ascione A, Capecchi B, Campitelli L, Imperiale V, Flego M, Zamboni S, et al. Human monoclonal antibodies in single chain fragment variable format with potent neutralization activity against influenza virus H5N1. Antiviral Res. 2009;83(3):238–44.

745. Thathaisong U, Maneewatch S, Kulkeaw K, Thueng-In K, Poungpair O, Srimanote P, et al. Human monoclonal single chain antibodies (HuScFv) that bind to the polymerase proteins of influenza A virus. Asian Pacific Journal of Allergy and Immunology. 2008;26(1):23–35.

746. Chadha M, Hirve S, Bancej C, Barr I, Baumeister E, Caetano B, et al. Human respiratory syncytial virus and influenza seasonality patterns—Early findings from the WHO global respiratory syncytial virus surveillance. Influenza and other Respiratory Viruses. 2020;14(6):638–46.

747. Sood R, Kumar N, Pateriya AK, Bhatia S, Panickan S, Mishra A, et al. Identification and molecular characterization of H9N2 viruses carrying multiple mammalian adaptation markers in resident birds in central-western wetlands in India. Infec Genet Evol. 2021;94:105005.

748. Shankar BP, Gowda RNS, Pattnaik B, Prabhu BHM, Sreenivas BK, Vinuthan MK, et al. Identification and subtyping of avian influenza viruses by reverse transcription polymerase chain reaction (rt-pcr) and agarose gel electrophoresis. International Journal of Poultry Science. 2009;8(5):465–9.

749. Dawson ED, Moore CL, Dankbar DM, Mehlmann M, Townsend MB, Smagala JA, et al. Identification of A/H5N1 influenza viruses using a single gene diagnostic microarray. Anal Chem. 2007;79(1):378–84.

750. Jonas M, Sahesti A, Murwijati T, Lestariningsih CL, Irine I, Ayesda CS, et al. Identification of avian influenza virus subtype H9N2 in chicken farms in Indonesia. Preventive Veterinary Medicine. 2018;159:99–105.

751. McKimm-Breschkin JL, Barrett S, Wong FYK, Pudjiatmoko, Azhar M, Selleck P, et al. Identification of Indonesian clade 2.1 highly pathogenic influenza A(H5N1) viruses with N294S and S246N neuraminidase substitutions which further reduce oseltamivir susceptibility. Antiviral Research. 2018;153:95–100.

752. Wang J, Vijaykrishna D, Duan L, Bahl J, Zhang JX, Webster RG, et al. Identification of the progenitors of Indonesian and Vietnamese avian influenza A (H5N1) viruses from southern China. J Virol. 2008;82(7):3405–14.

753. Widhidewi NW, Wiyatno A, Dewantari AK, Paramasatiari L, Aryastuti SA, Artika IN, et al. Identification of viral etiology of acute respiratory tract infections in children and adults in Tabanan, Bali, Indonesia. Access Microbiol. 2020;2(6):acmi000120.

754. Loth L, Gilbert M, Wu J, Czarnecki C, Hidayat M, Xiao X. Identifying risk factors of highly pathogenic avian influenza (H5N1 subtype) in Indonesia. Preventive Veterinary Medicine. 2011;102(1):50–8.

755. Laroia ST, Gupta E, Kumar S, Kumar G, Sarin SK. Imaging spectrum of H1N1 influenza from a tertiary liver hospital in India: First ever experience. Journal of Association of Physicians of India. 2019;67:37–41.

756. Dutta M, Dutta P, Medhi S, Borkakoty B, Biswas D. Immune response during influenza virus infection among the population of Assam, Northeast India. Indian Journal of Medical Microbiology. 2019;37(4):549–56.

757. Chotirosniramit N, Sugandhavesa P, Aurpibul L, Thetket S, Kosashunhanan N, Supindham T, et al. Immune response to 2009 H1N1 vaccine in HIV-infected adults in Northern Thailand. Human Vaccines and Immunotherapeutics. 2012;8(12):1854–9.

758. Moulik NR, Mandal P, Chandra J, Bansal S, Jog P, Sanjay S, et al. Immunization of Children with Cancer in India Treated with Chemotherapy — Consensus Guideline from the Pediatric Hematology-Oncology Chapter and the Advisory Committee on Vaccination and Immunization Practices of the Indian Academy of Pediatrics. Indian Pediatrics. 2019;56(12):1041–8.

759. Khan A, Ashher F, Karim T, Fatema A, Jahan I, Muhit M, et al. Immunization of mothers of children with cerebral palsy in rural bangladesh. Infectious Disorders - Drug Targets. 2020;20(3):303–8.

760. Sintusek P, Poovorawan Y. Immunization status and hospitalization for vaccine-preventable non-vaccine-preventable infections in liver-transplanted children. World Journal of Hepatology. 2021;13(1):120–31.

761. Soedjatmiko S, Medise BE, Gunardi H, Sekartini R, Satari HI, Hadinegoro SR, et al. Immunogenicity and safety of a Trivalent Influenza HA vaccine in Indonesian infants and children. Vaccine. 2018;36(16):2126–32.

762. Izurieta P, Kim WJ, Wie SH, Lee J, Lee JS, Dramé M, et al. Immunogenicity and safety of an AS03-adjuvanted H5N1 pandemic influenza vaccine in Korean adults: a phase IV, randomized, open-label, controlled study. Vaccine. 2015;33(24):2800–7.

763. Kankawinpong O, Sangsajja C, Cholapand A, Manosuthi W, Thientong V, Nuntapanich N, et al. Immunogenicity and safety of an inactivated pandemic H1N1 vaccine provided by the Thai ministry of public health as a routine public health service. Southeast Asian Journal of Tropical Medicine and Public Health. 2012;43(3):680–6.

764. Agarkhedkar S, Chhatwal J, Kompithra RZ, Lalwani SK, Narayan A, Muninarayanaswam V, et al. Immunogenicity and safety of an intramuscular split-virion quadrivalent inactivated influenza vaccine in individuals aged ≥ 6 months in India. Human Vaccines and Immunotherapeutics. 2019;15(4):973–7.

765. Phongsamart W, Sirisanthana V, Wittawatmongkol O, Maleesatharn A, Sudjaritruk T, Chearskul P, et al. Immunogenicity and safety of monovalent influenza A (H1N1) 2009 in HIV-infected Thai children. Vaccine. 2011;29(47):8705–11.

766. Dhamayanti M, Tarigan R, Fadlyana E, Prasetyo D, Amalia N, Rusmil VK, et al. Immunogenicity and safety of Quadrivalent Influenza HA vaccine in Indonesian children: An open-labeled, bridging, clinical study. Vaccine. 2020;38(5):993–1000.

767. Sharma S, Singh VB, Kumar S, Prajapati V, Patel J, Vukkala R, et al. Immunogenicity and safety of the first indigenously developed Indian tetravalent influenza vaccine (split virion) in healthy adults ≥ 18 years of age: A randomized, multicenter, phase II / III clinical trial. Human Vaccines and Immunotherapeutics. 2018;14(6):1362–9.

768. Sarkar S, Bokade C, Garg K, Kumar R, Sanmukhani J, Mittal R. Immunogenicity and safety of the first indigenously developed Indian tetravalent influenza vaccine (split virion) in healthy children (6 months to 17 years of age): a randomized, multicenter, phase III clinical trial. Human Vaccines and Immunotherapeutics. 2021;17(3):681–9.

769. Basu I, Agarwal M, Shah V, Shukla V, Naik S, Supe PD, et al. Immunogenicity and safety of two quadrivalent influenza vaccines in healthy adult and elderly participants in India - A phase III, active-controlled, randomized clinical study. Human Vaccines and Immunotherapeutics [Internet]. 2021; Available from: https://www.scopus.com/inward/record.uri?eid=2-s2.0-85105955103&doi=10.1080%2f21645515.2021.1885278&partnerID=40&md5=fc207c025f835a84173457720e472366

770. Chu DWS, Hwang SJ, Lim FS, Oh HML, Thongcharoen P, Yang PC, et al. Immunogenicity and tolerability of an AS03A-adjuvanted prepandemic influenza vaccine: A phase III study in a large population of Asian adults. Vaccine. 2009;27(52):7428–35.

771. Lewis KDC, Ortiz JR, Rahman MZ, Levine MZ, Rudenko L, Wright PF, et al. Immunogenicity and Viral Shedding of Russian-Backbone, Seasonal, Trivalent, Live, Attenuated Influenza Vaccine in a Phase II, Randomized, Placebo-Controlled Trial among Preschool-Aged Children in Urban Bangladesh. Clinical Infectious Diseases. 2019;69(5):777–85.

772. Wu J, Fang HH, Chen JT, Zhou JC, Feng ZJ, Li CG, et al. Immunogenicity, safety, and cross-reactivity of an inactivated, adjuvanted, prototype pandemic influenza (H5N1) vaccine: a phase II, double-blind, randomized trial. Clin Infect Dis. 2009;48(8):1087–95.

773. He F, Du Q, Ho Y, Kwang J. Immunohistochemical detection of Influenza virus infection in formalin-fixed tissues with anti-H5 monoclonal antibody recognizing FFWTILKP. J Virol Methods. 2009;155(1):25–33.

774. Zulfikhar Z, Wasito R, Wuryastuti H. Immunopathological immunohistochemical study of low pathogenic avian influenza virus H5N1 infection in lovebirds (Agapornis spp.) in Indonesia. Veterinary World. 2019;12(9):1472–7.

775. Boongird C, Thamakaison S, Krairit O. Impact of a geriatric assessment clinic on organizational interventions in primary health-care facilities at a university hospital. Geriatrics and Gerontology International. 2011;11(2):204–10.

776. Suntronwong N, Thongpan I, Chuchaona W, Budi Lestari F, Vichaiwattana P, Yorsaeng R, et al. Impact of COVID-19 public health interventions on influenza incidence in Thailand. Pathog Global Health. 2020;114(5):225–7.

777. Tricco AC, Lillie E, Soobiah C, Perrier L, Straus SE. Impact of H1N1 on socially disadvantaged populations: summary of a systematic review. Influenza Other Respir Viruses. 2013;7:54–8.

778. Ram PK, DiVita MA, Krytus K, Cercone E, Khatun-e-Jannat K, Islam M, et al. Impact of intensive handwashing promotion on secondary household influenza-like illness in rural Bangladesh: Findings from a randomized controlled trial. PLoS ONE. 2015;10(6):e0125200.

779. Kozuki N, Katz J, Englund JA, Steinhoff MC, Khatry SK, Shrestha L, et al. Impact of maternal vaccination timing and influenza virus circulation on birth outcomes in rural Nepal. International Journal of Gynecology and Obstetrics. 2018;140(1):65–72.

780. Silaporn P, Jiamsiri S. Impact of national influenza vaccine campaign on respiratory illness in thailand, 2010-2011. Southeast Asian Journal of Tropical Medicine and Public Health. 2018;49(2):266–75.

781. Champunot R, Tanjatham S, Kerdsin A, Puangpatra P, Wangsai S, Treebuphachatsakul P, et al. Impact of pandemic influenza (H1N1) virus-associated community-acquired pneumonia among adults in a tertiary hospital in Thailand. Japanese Journal of Infectious Diseases. 2010;63(4):251–6.

782. Katz J, Englund JA, Steinhoff MC, Khatry SK, Shrestha L, Kuypers J, et al. Impact of timing of influenza vaccination in pregnancy on transplacental antibody transfer, influenza incidence, and birth outcomes: A randomized trial in Rural Nepal. Clinical Infectious Diseases. 2018;67(3):334–40.

783. Swayne DE. Impact of vaccines and vaccination on global control of avian influenza. Avian Dis. 2012;56(4):818–28.

784. Pfeiffer DU, Otte MJ, Roland-Holst D, Inui K, Nguyen T, Zilberman D. Implications of global and regional patterns of highly pathogenic avian influenza virus H5N1 clades for risk management. Vet J. 2011;190(3):309–16.

785. van Boeckel TP, Gilbert M, Thanapongtharm W, Robinson T, Biradar CM, Xiao X. Improving Risk Models for Avian Influenza: The Role of Intensive Poultry Farming and Flooded Land during the 2004 Thailand Epidemic. PLoS ONE. 2012;7(11):e49528.

786. Siddiqui A, Chowdhary R, Maan HS, Goel SK, Tripathi N, Prakash A. In silico analysis and molecular characterization of influenza a (H1N1) PDM09 virus circulating and causing major outbreaks in Central India, 2009-2019. Iranian Journal of Microbiology. 2020;12(5):483–94.

787. Tambunan USF, Limanto A, Parikesit AA. In silico analysis of hemagglutinin, neuraminidase, and matrix2 of h5n1 virus indonesian strain related to its high pathogenicity. IIOAB Journal. 2010;1(3):17–24.

788. Behera DK, Behera PM, Acharya L, Dixit A, Padhi P. In silico biology of H1N1: molecular modelling of novel receptors and docking studies of inhibitors to reveal new insight in flu treatment. J Biomed Biotechnol. 2012;2012:714623.

789. Jain B, Jain A, Prakash O, Singh AK, Dangi T, Singh M, et al. In silico designing of siRNA targeting PB 1 gene of Influenza A virus and in vitro validation. Journal of Applied Pharmaceutical Science. 2014;4(8):42–7.

790. Tambunan USF, Witanto DF, Parikesit AA. In silico genetic variation pathogenicity analysis of hemagglutinin, matrix 1, and non structural 1 protein of human H5N1 Indonesian strain. IIOAB Journal. 2012;3(3):5–14.

791. Singh KD, Muthusamy K. In silico genome analysis and drug efficacy test of influenza A virus (H1N1) 2009. Indian Journal of Microbiology. 2009;49(4):358–64.

792. Mandal RS, Panda S, Das S. In silico prediction of drug resistance due to S247R mutation of Influenza H1N1 neuraminidase protein. J Biomol Struct Dyn. 2018;36(4):966–80.

793. Pallavi S, Shruti S, Anismita S, Tanisha U, Sumathra M, Manjunatha Reddy AH. In silico study of the therapeutic role of natural compounds in influenza A. Research Journal of Biotechnology. 2020;15(6):135–45.

794. Chutinimitkul S, van Riel D, Munster VJ, van den Brand JMA, Rimmelzwaan GF, Kuiken T, et al. In vitro assessment of attachment pattern and replication efficiency of H5N1 influenza A viruses with altered receptor specificity. J Virol. 2010;84(13):6825–33.

795. Jain B, Jain A, Prakash O, Singh AK, Dangi T, Singh M, et al. In vitro validation of self designed “universal human Influenza A siRNA”. Indian J Exp Biol. 2015;53(8):514–21.

796. Seniya C, Khan GJ, Misra R, Vyas V, Kaushik S. In-silico modelling and identification of a possible inhibitor of H1N1 virus. Asian Pacific Journal of Tropical Disease. 2014;4:S467–76.

797. Asaf VNM, Kumar A, Raut AA, Bhatia S, Mishra A. In-silico search of virus-specific host microRNAs regulating avian influenza virus NS1 expression. Theory Biosci. 2015;134(1):65–73.

798. Swayne DE, Lee CW, Spackman E. Inactivated North American and European H5N2 avian influenza virus vaccines protect chickens from Asian H5N1 high pathogenicity avian influenza virus. Avian Pathol. 2006;35(2):141–6.

799. Murtadak VB, Mishra AC, Pawar SD. Inactivation of avian influenza (AI) H9N2 virus isolated from india for its potential use as a candidate vaccine. Indian J Virol. 2013;24(1):107.

800. Isbarn S, Buckow R, Himmelreich A, Lehmacher A, Heinz V. Inactivation of avian influenza virus by heat and high hydrostatic pressure. J Food Prot. 2007;70(3):667–73.

801. Sirivichayakul C, Sabcharoen A, Chanthavanich P, Chokejindachai W, Thawatsupha P, Suthisarnsunthorn U, et al. Incidence and clinical manifestations of influenza in nurse assistant students. Southeast Asian Journal of Tropical Medicine and Public Health. 2000;31(1):57–61.

802. Baggett HC, Chittaganpitch M, Thamthitiwat S, Prapasiri P, Naorat S, Sawatwong P, et al. Incidence and epidemiology of hospitalized influenza cases in rural Thailand during the influenza A (H1N1)pdm09 pandemic, 2009-2010. PLoS ONE. 2012;7(11):e48609.

803. Hasan R, Rhodes J, Thamthitiwat S, Olsen SJ, Prapasiri P, Naorat S, et al. Incidence and etiology of acute lower respiratory tract infections in hospitalized children younger than 5 years in rural Thailand. Pediatric Infectious Disease Journal. 2014;33(2):e45–52.

804. Tuladhar S, Das P, Samaddar S, Bhattacharyya S, Bhattacharyya A. Incidence and outcome of Influenza in children with cancer and impact of Influenza vaccination: report from a tertiary cancer centre in Eastern India. Pediatr Hematol Oncol J. 2019;4(2):S18.

805. Henning J, Morton JM, Wibawa H, Yulianto D, Usman TB, Prijono W, et al. Incidence and risk factors for H5 highly pathogenic avian influenza infection in flocks of apparently clinically healthy ducks. Epidemiology and Infection. 2013;141(2):390–401.

806. Sindhu T. Incidence and severity of H1N1 influenza ARDS [2018-2019] amongst suspected viral pneumonia cases in a tertiary care centre from South India. Indian J Crit Care Med. 2020;24:S31–2.

807. Biswas PK, Giasuddin M, Chowdhury P, Barua H, Debnath NC, Yamage M. Incidence of contamination of live bird markets in Bangladesh with influenza A virus and subtypes H5, H7 and H9. Transboundary and Emerging Diseases. 2018;65(3):687–95.

808. Dawood FS, Kittikraisak W, Patel A, Rentz Hunt D, Suntarattiwong P, Wesley MG, et al. Incidence of influenza during pregnancy and association with pregnancy and perinatal outcomes in three middle-income countries: a multisite prospective longitudinal cohort study. The Lancet Infectious Diseases. 2021;21(1):97–106.

809. Steinhoff MC, McNeal M, Henkle E, Moss WJ, Omer SB, Arifeen SE, et al. Incidence Of Influenza Infection In Early Infancy In South Asia. Am J Trop Med Hyg. 2010;83(5):356.

810. Hirve S, Krishnan A, Dawood FS, Lele P, Saha S, Rai S, et al. Incidence of influenza-associated hospitalization in rural communities in western and northern India, 2010-2012: A multi-site population-based study. Journal of Infection. 2015;70(2):160–70.

811. Azziz-Baumgartner E, Alamgir ASM, Rahman M, Homaira N, Sohel BM, Yushuf Sharker MA, et al. Incidence of influenza-like illness and severe acute respiratory infection during three influenza seasons in Bangladesh, 2008-2010. Bulletin of the World Health Organization. 2012;90(1):12–9.

812. Piralam B, Tomczyk SM, Rhodes JC, Thamthitiwat S, Gregory CJ, Olsen SJ, et al. Incidence of pneumococcal pneumonia among adults in rural Thailand, 2006-2011: Implications for pneumococcal vaccine considerations. American Journal of Tropical Medicine and Hygiene. 2015;93(6):1140–7.

813. Olsen SJ, Thamthitiwat S, Chantra S, Chittaganpitch M, Fry AM, Simmerman JM, et al. Incidence of respiratory pathogens in persons hospitalized with pneumonia in two provinces in Thailand. Epidemiology and Infection. 2010;138(12):1811–22.

814. Homaira N, Rahman M, Hossain K, Zesmin F, Alam M, Gurley ES, et al. Incidence of respiratory virus-associated pneumonia in urban poor young children of dhaka, bangladesh, 2009-2011. PLoS ONE. 2012;7(2):e32056.

815. Fowler KB, Gupta V, Sullender W, Broor S, Widdowson MA, Lal RB, et al. Incidence of symptomatic A(H1N1)pdm09 influenza during the pandemic and post-pandemic periods in a rural Indian community. International Journal of Infectious Diseases. 2013;17(12):e1182–5.

816. Kumar R, Dar L, Amarchand R, Saha S, Lafond KE, Purakayastha DR, et al. Incidence, risk factors, and viral etiology of community-acquired acute lower respiratory tract infection among older adults in rural north India. Journal of global health. 2021;11:04027.

817. Simmerman JM, Levy J, Maloney S, Thamthitiwat S, Baggett HC, Uyeki T, et al. Incidence, seasonality and mortality associated with influenza pneumonia in Thailand: 2005-2008. PLoS ONE. 2009;4(11):e7776.

818. Levy JW, Suntarattiwong P, Simmerman JM, Jarman RG, Johnson K, Olsen SJ, et al. Increased hand washing reduces influenza virus surface contamination in Bangkok households, 2009-2010. Influenza and other Respiratory Viruses. 2014;8(1):13–6.

819. Sawani A, Anderson K, Suwanpakdee D, Watanaveeradej V, Kerdpanich P, Phiboonbanakit D, et al. Increasing elective haemophilus influenza type B vaccine coverage in Thailand. Am J Trop Med Hyg. 2018;99(4):148.

820. Guha S. India in the pandemic age. Indian Econ Rev. 2020;1–18.

821. Nambiar P. India to Envision One Health Movement for Confronting Emerging Health Threats: From Concept to Approach Toward Institutionalization. International Journal of One Health. 2020;6(2):165–76.

822. Chaichoune K, Wiriyarat W, Thitithanyanont A, Phonarknguen R, Sariya L, Suwanpakdee S, et al. Indigenous sources of 2007-2008 H5N1 avian influenza outbreaks in Thailand. Journal of General Virology. 2009;90(1):216–22.

823. Ram PK, DiVita M, Cercone E, Rook K, Yu J, Islam M, et al. Individual level risk factors for secondary transmission of influenza-like illness: Secondary data analysis from the bangladesh interruption of secondary transmission of influenza study (bistis). Am J Trop Med Hyg. 2012;87(5):285.

824. Ichinohe T, Ainai A, Nakamura T, Akiyama Y, Maeyama JI, Odagiri T, et al. Induction of cross-protective immunity against influenza A virus H5N1 by an intranasal vaccine with extracts of mushroom mycelia. J Med Virol. 2010;82(1):128–37.

825. Murray AF, Englund JA, Kuypers J, Tielsch JM, Katz J, Khatry SK, et al. Infant Pneumococcal Carriage during Influenza, RSV, and hMPV Respiratory Illness Within a Maternal Influenza Immunization Trial. Journal of Infectious Diseases. 2019;220(6):956–60.

826. Hughes MM, Katz J, Englund JA, Khatry SK, Shrestha L, LeClerq SC, et al. Infant vaccination timing: Beyond traditional coverage metrics for maximizing impact of vaccine programs, an example from southern Nepal. Vaccine. 2016;34(7):933–41.

827. Michaelis M, Geiler J, Klassert D, Doerr HW, Cinatl JJ. Infection of human retinal pigment epithelial cells with influenza A viruses. Invest Ophthalmol Vis Sci. 2009;50(11):5419–25.

828. Kreijtz JHCM, Bodewes R, van den Brand JMA, de Mutsert G, Baas C, van Amerongen G, et al. Infection of mice with a human influenza A/H3N2 virus induces protective immunity against lethal infection with influenza A/H5N1 virus. Vaccine. 2009;27(36):4983–9.

829. Bunpapong N, Boonyapisitsopa S, Suwannakarn K, Tantilertchareon R, Thanawongnuwech R, Amonsin A. Infection of Thai influenza A viruses subtype H5N1 using tracheal culture. Thai Journal of Veterinary Medicine. 2015;45(2):181–8.

830. Bingham J, Green DJ, Lowther S, Klippel J, Burggraaf S, Anderson DE, et al. Infection studies with two highly pathogenic avian influenza strains (Vietnamese and Indonesian) in Pekin ducks (Anas platyrhynchos), with particular reference to clinical disease, tissue tropism and viral shedding. Avian Pathology. 2009;38(4):267–78.

831. Nasreen S, Rahman M, Hancock K, Katz JM, Goswami D, Sturm-Ramirez K, et al. Infection with influenza A(H1N1)pdm09 during the first wave of the 2009 pandemic: Evidence from a longitudinal seroepidemiologic study in Dhaka, Bangladesh. Influenza and other Respiratory Viruses. 2017;11(5):394–8.

832. Budwong A, Auephanwiriyakul S, Theera-Umpon N. Infectious disease relational data analysis using string grammar non-euclidean relational fuzzy c-means. International Journal of Environmental Research and Public Health [Internet]. 2021;18(15). Available from: https://www.scopus.com/inward/record.uri?eid=2-s2.0-85111466913&doi=10.3390%2fijerph18158153&partnerID=40&md5=075ceab84817d615b95cb4109d2ab0b1

833. Luby SP, Brooks WA, Zaman K, Hossain S, Ahmed T. Infectious diseases and vaccine sciences: Strategic directions. Journal of Health, Population and Nutrition. 2008;26(3):295–310.

834. Khanna V, Bairy I, Mukhopadayay C, Khanna R, Kumar M. Influence of H1N1 pandemic on attitude and intended behaviour of university students: A cross sectional study from south India. Internet Journal of Infectious Diseases [Internet]. 2011;9(2). Available from: https://www.scopus.com/inward/record.uri?eid=2-s2.0-80054762050&partnerID=40&md5=9b5842e9e0727d505d4374946f7658f8

835. Ramakrishna K, Peter JV, Karthik G, Abraham AM, Surekha V, Karthik R, et al. Influenza A (H1N1) 2009 pandemic: Was there a difference in the two waves in patients requiring admission to the intensive-care unit? Clinical Microbiology and Infection. 2011;17(9):1355–8.

836. Trakulsrichai S, Watcharananan SP, Chantratita W. Influenza A (H1N1) 2009 reinfection in Thailand. Journal of Infection and Public Health. 2012;5(2):211–4.

837. Kulkarni SV, Narain JP, Gupta S, Dhariwal AC, Singh SK, Macintyre CR. Influenza a (H1N1) in India: Changing epidemiology and its implications. National Medical Journal of India. 2019;32(2):107–8.

838. Chawla R, Sharma RK, Bhardwaj JR. Influenza A (H1N1) outbreak and challenges for pharmacotherapy. Indian Journal of Physiology and Pharmacology. 2009;53(2):113–26.

839. Indhumathi E, Krishna Makkena V, Mamidi V, Jayaprakash V, Jayakumar M. Influenza A (H1N1) Virus Infection Associated Acute Kidney Injury - A Study from a Tertiary Care Center in South India. Saudi Journal of Kidney Diseases and Transplantation. 2020;31(4):759–66.

840. Sangle SA, Vadgaonkar G, Kadam DB, Chadha M. Influenza a (H3N2) associated acute necrotising encephalopathy. Journal of Association of Physicians of India [Internet]. 2011;59(1). Available from: https://www.scopus.com/inward/record.uri?eid=2-s2.0-79960957254&partnerID=40&md5=54a074006f29553818ae3e2bd3e3e311

841. Canas LC, Macias EA, Niemeyer D, Gould P, Chambers JP, Renthal R, et al. Influenza A (H3N2) outbreak, Nepal. Emerg Infect Dis. 2005;11(8):1186–91.

842. Nidom CA, Takano R, Yamada S, Sakai-Tagawa Y, Daulay S, Aswadi D, et al. Influenza a (H5N1) viruses from pigs, Indonesia. Emerging Infectious Diseases. 2010;16(10):1515–23.

843. Rutvisuttinunt W, Pollett SD, Berry IM, Jarman RG, Huang A, Manasatienkij W, et al. Influenza A and B virus epidemics in Bhutan, Cambodia, Nepal, Philippines and Thailand are characterized by repeated introductions and limited persistence of circulating strains. Am J Trop Med Hyg. 2018;99(4):302–3.

844. Nagarajan K, Saikumar G, Arya RS, Gupta A, Somvanshi R, Pattnaik B. Influenza A H1N1 virus in Indian pigs & its genetic relatedness with pandemic human influenza A 2009 H1N1. Indian J Med Res. 2010;132:160–7.

845. Wallace RG, Fitch WM. Influenza A H5N1 immigration is filtered out at some international borders. PLoS One. 2008;3(2):e1697.

846. Tiwari N, Verma S, Dhole TN. Influenza a pandemic (P-H1N1) flu and its circulation trend in northern India: A review. Intl J Pharm Sci Rev Res. 2014;25(2):252–8.

847. Wisedchanwet T, Wongpatcharachai M, Boonyapisitsopa S, Bunpapong N, Jairak W, Kitikoon P, et al. Influenza A virus surveillance in live-bird markets: First report of influenza A virus subtype H4N6, H4N9, and H10N3 in Thailand. Avian Diseases. 2011;55(4):593–602.

848. Guleria R, Kumar J, Mohan A, Wig N. Influenza A: From highly pathogenic H5N1 to pandemic 2009 H1N1. Epidemiology and clinical features. Indian Journal of Microbiology. 2009;49(4):315–9.

849. Sharma P, Gupta S, Singh D, Verma S, Kanga A. Influenza A(H1N1)pdm09 cases in sub-Himalayan region, 2014-2015 India. Indian Journal of Pathology and Microbiology. 2016;59(1):63–5.

850. Gurav YK, Chadha MS, Tandale BV, Potdar VA, Pawar SD, Shil P, et al. Influenza A(H1N1)pdm09 outbreak detected in inter-seasonal months during the surveillance of influenza-like illness in Pune, India, 2012-2015. Epidemiology and Infection. 2017;145(9):1898–909.

851. Bunthi C, Thamthitiwat S, Baggett HC, Maloney SA, Akarasewi P, Ungchusak K, et al. Influenza A(H1N1)pdm09-Associated Pneumonia Deaths in Thailand. PLoS ONE. 2013;8(2):e54946.

852. Nonthabenjawan N, Chaiyawong S, Bunpapong N, Boonyapisitsopa S, Janetanakit T, Amonsin A, et al. Influenza A(H9N2) virus, Myanmar, 2014-2015. Emerg Infect Dis. 2017;23(6):1041–3.

853. Sareen S, Singh P, Miglani U. Influenza a/H1N1 (2009) infection in pregnancy in a tertiary care hospital in India. Journal of the Indian Medical Association. 2016;114(1):226–30.

854. Setiawaty V, Roselinda R, Sampurno OD. Influenza activities in Indonesia in 2010-2011. Int J Infect Dis. 2012;16:e144.

855. Prachayangprecha S, Vichaiwattana P, Korkong S, Felber JA, Poovorawan Y. Influenza activity in Thailand and occurrence in different climates. Springerplus. 2015;4:356.

856. Rotrosen E, Zaman K, Feser J, Ortiz JR, Goswami D, Sharmeen AT, et al. Influenza among Young Children in Bangladesh: Clinical Characteristics and Outcomes from a Randomized Clinical Trial. Clinical Infectious Diseases. 2017;65(11):1914–20.

857. Wiwanitkit V. Influenza and diabetes mellitus. Diabetes and Metabolic Syndrome: Clinical Research and Reviews. 2010;4(2):99–100.

858. Bhalerao-Gandhi A, Chhabra P, Arya S, Simmerman JM. Influenza and pregnancy: A review of the literature from India. Infectious Diseases in Obstetrics and Gynecology [Internet]. 2015;2015. Available from: https://www.scopus.com/inward/record.uri?eid=2-s2.0-84924351868&doi=10.1155%2f2015%2f867587&partnerID=40&md5=f539c6efdbba07e29831b528dbb734aa

859. Koul PA, Potdar V, Showkat M, Mir H, Chadha MS. Influenza B in a temperate region of northern India 2010–2016: co-circulation of the two lineages with northern hemispherical seasonality. VirusDisease. 2018;29(4):553–9.

860. Haque F, Sturm-Ramirez K, Homaira N, Gurley ES, Hossain MJ, Hasan SMM, et al. Influenza B virus outbreak at a religious residential school for boys in Northern Bangladesh, 2011. Influenza and other Respiratory Viruses. 2017;11(2):165–9.

861. Puig-Barberà J, Mira-Iglesias A, Burtseva E, Cowling BJ, Serhat U, Ruiz-Palacios GM, et al. Influenza epidemiology and influenza vaccine effectiveness during the 2015-2016 season: Results from the Global Influenza Hospital Surveillance Network. BMC Infectious Diseases [Internet]. 2019;19(1). Available from: https://www.scopus.com/inward/record.uri?eid=2-s2.0-85065739242&doi=10.1186%2fs12879-019-4017-0&partnerID=40&md5=e3acac9a80f352e059a45103516f7084

862. Koul PA, Bali NK, Mir H, Jabeen F, Ahmad A. Influenza Illness in Pregnant Indian Women: A Cross-Sectional Study. Infectious Diseases in Obstetrics and Gynecology [Internet]. 2016;2016. Available from: https://www.scopus.com/inward/record.uri?eid=2-s2.0-84958580920&doi=10.1155%2f2016%2f1248470&partnerID=40&md5=42ab10658700f5ea8d2d973cf8941550

863. Influenza in outpatient ILI case-patients in national hospital-based surveillance, Bangladesh, 2007-2008. PLoS ONE. 2009;4(12):e8452.

864. Watthanaworawit W, Carrara VI, Turner P, Turner CL, Nosten FH, Kapella BK, et al. Influenza in refugees on the Thailand-Myanmar border, May-October 2009. Emerg Infect Dis. 2010;16(9):1366–72.

865. Simmerman JM, Thawatsupha P, Kingnate D, Fukuda K, Chaising A, Dowell SF. Influenza in Thailand: A case study for middle income countries. Vaccine. 2004;23(2):182–7.

866. Cowling BJ, Caini S, Chotpitayasunondh T, Djauzi S, Gatchalian SR, Huang QS, et al. Influenza in the Asia-Pacific region: Findings and recommendations from the Global Influenza Initiative. Vaccine. 2017;35(6):856–64.

867. Koul PA, Mir H, Saha S, Chadha MS, Potdar V, Widdowson MA, et al. Influenza not MERS CoV among returning Hajj and Umrah pilgrims with respiratory illness, Kashmir, north India, 2014–15. Travel Medicine and Infectious Disease. 2017;15:45–7.

868. Kakkar M, Hazarika S, Zodpey S, Reddy KS. Influenza pandemic preparedness and response: A review of legal frameworks in India. Indian journal of public health. 2010;54(1):11–7.

869. Kamigaki T, Oshitani H. Influenza pandemic preparedness and severity assessment of pandemic (H1N1) 2009 in South-east Asia. Public Health. 2010;124(1):5–9.

870. Fedson DS. Influenza pandemic preparedness: A special challenge for india. Indian J Med Res Suppl. 2019;150(3):217–20.

871. Saha S, Chadha M, Al Mamun A, Rahman M, Sturm-Ramirez K, Chittaganpitch M, et al. Influenza seasonality and vaccination timing in tropical and subtropical areas of southern and south-eastern Asia. Bulletin of the World Health Organization. 2014;92(5):318–30.

872. Rungrojcharoenkit K, Klungthong C, Yoon IK, Fernandez S, Rungorjcharoenkit K, Kittikraisak W, et al. Influenza seroconversion rates in a cohort of young children, Bangkok, Thailand. Am J Trop Med Hyg. 2015;93(4):53.

873. Wangchuk S, Thapa B, Zangmo S, Jarman RG, Bhoomiboonchoo P, Gibbons RV. Influenza surveillance from November 2008 to 2011; including pandemic influenza A(H1N1)pdm09 in Bhutan. Influenza and other Respiratory Viruses. 2013;7(3):426–30.

874. Kosasih H, Ma’roef C, Listiyaningsih E, Elyazar IRF, Wuryadi S, McArdle JL, et al. Influenza surveillance in Indonesia: 1999-2003. Clin Infect Dis. 2004;39(4):443–9.

875. Yeolekar LR, Kadam SS, Pawar MS, Kulkarni PB, More BA, Khude MR, et al. Influenza surveillance in Pune, India, 2003. Southeast Asian J Trop Med Public Health. 2005;36(4):906–9.

876. Kanchana S, Kanchana S, Prachayangprecha S, Makkoch J, Chantrakul C, Poovorawan Y. Influenza surveillance in Southern Thailand during 2009-2010. Southeast Asian Journal of Tropical Medicine and Public Health. 2012;43(4):871–6.

877. Tharakaraman K, Sasisekharan R. Influenza surveillance: 2014-2015 H1N1 “swine”-derived influenza viruses from India. Cell Host and Microbe. 2015;17(3):279–82.

878. Chotpitayasunondh T, Sawanpanyalert N, Bumrungsak R, Chunthitiwong P, Chainatraporn P. Influenza vaccination among health care workers in Thailand. BMC Proc. 2011;5.

879. Kittikraisak W, Suntarattiwong P, Levy J, Fernandez S, Dawood FS, Olsen SJ, et al. Influenza vaccination coverage and effectiveness in young children in Thailand, 2011-2013. Influenza and other Respiratory Viruses. 2015;9(2):85–93.

880. Mathew JL. Influenza vaccination for children in India. Indian Pediatrics. 2009;46(4):304–7.

881. Gupta V, Dawood FS, Kapella BK, Kitsutani P, Corwin A, Olsen SJ, et al. Influenza Vaccination Guidelines and Vaccine Sales in Southeast Asia: 2008-2011. PLoS ONE. 2012;7(12):e52842.

882. Sribhutorn A, Phrommintikul A, Wongcharoen W, Chaikledkaew U, Eakanunkul S, Sukonthasarn A. Influenza vaccination in acute coronary syndromes patients in Thailand: The cost-effectiveness analysis of the prevention for cardiovascular events and pneumonia. Journal of Geriatric Cardiology. 2018;15(6):413–21.

883. Vashishtha VM, Kalra A, Choudhury P. Influenza vaccination in India: Position paper of Indian Academy of Pediatrics, 2013. Indian Pediatr. 2013;50(9):867–74.

884. Koul PA, Ali S, Mir H, Ahmad SJ, Bhat SA, Bhat MA. Influenza vaccination in north Indian patients with heart failure. Indian Heart Journal. 2017;69(1):28–31.

885. Giduthuri JG, Purohit V, Maire N, Kudale A, Utzinger J, Schindler C, et al. Influenza vaccination of pregnant women: Engaging clinicians to reduce missed opportunities for vaccination. Vaccine. 2019;37(14):1910–7.

886. Vijayasaratha K, Basumani K, Sasank A. Influenza vaccination uptake, awareness, and barriers among healthcare workers (HCWs) at tertiary care setting in India. Eur Respir J [Internet]. 2019;54. Available from: https://erj.ersjournals.com/content/54/suppl_63/PA4548

887. Syarif R, Syarif S, Wahyono TY. Influenza vaccine and the frequency of acute respiratory tract infection of West Java’s pilgrims-indonesia: Is there any correlations between? (an analysis of siskohatkes indonesian hajj data 2018). Indian J Public Health Res Dev. 2020;11(3):1065–71.

888. Alfelali M, Barasheed O, Koul P, Badahdah AM, Bokhary H, Tashani M, et al. Influenza vaccine effectiveness among Hajj pilgrims: a test-negative case-control analysis of data from different Hajj years. Expert Review of Vaccines. 2019;18(10):1103–14.

889. Levy JW, Simasathien S, Watanaveeradej V, Bhoomiboonchoo P, Fernandez S, Jarman RG, et al. Influenza vaccine effectiveness in the tropics: moderate protection in a case test-negative analysis of a hospital-based surveillance population in Bangkok between August 2009 and January 2013. PLoS One. 2015;10(8):e0134318.

890. Kopsaftis Z, Wood-Baker R, Poole P. Influenza vaccine for chronic obstructive pulmonary disease (COPD). Cochrane Database Syst Rev. 2018;6(6):CD002733.

891. Ortiz JR, Englund JA, Neuzil KM. Influenza vaccine for pregnant women in resource-constrained countries: a review of the evidence to inform policy decisions. Vaccine. 2011;29(27):4439–52.

892. Jadhav S, Dhere R, Yeolekar L, Gautam M. Influenza vaccine production capacity building in developing countries: Example of the Serum Institute of India. Procedia Vaccinology. 2010;2(2):166–71.

893. Nayak S. Influenza Vaccine Requirements in Pregnant Women. Journal of Obstetrics and Gynecology of India. 2016;66(2):76–80.

894. Singh M, Tanvir T, Nagoji D, Madan A, Gattem S, Singh H. Influenza vaccine: A viable option to protect pregnant women and infants from seasonal flu: A retrospective hospital-based study in India. International Journal of Clinical Practice [Internet]. 2019;73(7). Available from: https://www.scopus.com/inward/record.uri?eid=2-s2.0-85068872947&doi=10.1111%2fijcp.13361&partnerID=40&md5=42619d4a54081b23e69528ec78c6b507

895. Amonsin A, Choatrakol C, Lapkuntod J, Tantilertcharoen R, Thanawongnuwech R, Suradhat S, et al. Influenza virus (H5N1) in live bird markets and food markets, Thailand. Emerging Infectious Diseases. 2008;14(11):1739–42.

896. Babakir-Mina M, Balestra E, Perno CF, Aquaro S. Influenza virus A (H5N1): a pandemic risk? New Microbiol. 2007;30(2):65–78.

897. Hindupur A, Dhandapani P, Menon T. Influenza Virus Among Children with Acute Respiratory Infections in Chennai, India. Indian Pediatr. 2019;56(1):74–5.

898. Simmerman JM, Suntarattiwong P, Levy J, Gibbons RV, Cruz C, Shaman J, et al. Influenza virus contamination of common household surfaces during the 2009 influenza A (H1N1) Pandemic in Bangkok, Thailand: Implications for contact transmission. Clinical Infectious Diseases. 2010;51(9):1053–61.

899. Dangi T, Jain B, Singh AK, Mohan M, Dwivedi M, Singh KP, et al. Influenza virus genotypes circulating in and around Lucknow, Uttar Pradesh, India, during post pandemic period, august 2010 - September 2012. Indian J Med Res. 2014;139:418–26.

900. Dilantika C, Sedyaningsih ER, Kasper MR, Agtini M, Listiyaningsih E, Uyeki TM, et al. Influenza virus infection among pediatric patients reporting diarrhea and influenza-like illness. BMC Infectious Diseases [Internet]. 2010;10. Available from: https://www.scopus.com/inward/record.uri?eid=2-s2.0-76849117365&doi=10.1186%2f1471-2334-10-3&partnerID=40&md5=ea054dea9bc1a6dc96879a569d965028

901. Perera KV, Chan KH, Ma E, Peiris JS. Influenza virus infections among a sample of hospital attendees in Ragama, Sri Lanka. The Ceylon medical journal. 2010;55(2):40–4.

902. Rungrojcharoenkit K, Kittikraisak W, Ditsungnoen D, Olsen SJ, Suntarattiwong P, Chotpitayasunondh T, et al. Influenza virus seroincidence in a cohort of healthy and high-risk children enrolled in infancy, Bangkok, Thailand. International Journal of Infectious Diseases. 2019;89:21–6.

903. Mungaomklang A, Chomcheoy J, Wacharapluesadee S, Joyjinda Y, Jittmittraphap A, Rodpan A, et al. Influenza virus-associated fatal acute necrotizing encephalopathy: Role of nonpermissive viral infection? Clinical Medicine Insights: Case Reports. 2016;9:99–102.

904. Kollerova E, Betáková T. Influenza viruses and their ion channels. Acta Virol. 2006;50(1):7–16.

905. Waicharoen S, Thawatsupha P, Chittaganpitch M, Maneewong P, Thanadachakul T, Sawanpanyalert P. Influenza viruses circulating in Thailand in 2004 and 2005. Japanese Journal of Infectious Diseases. 2008;61(4):321–3.

906. Chittaganpitch M, Supawat K, Olsen SJ, Waicharoen S, Patthamadilok S, Yingyong T, et al. Influenza viruses in Thailand: 7years of sentinel surveillance data, 2004-2010. Influenza and other Respiratory Viruses. 2012;6(4):276–83.

907. Suntarattiwong P, Sian-nork C, Thongtipa P, Thawatsupha P, Kitphati R, Chotpitayasunondh T. Influenza-associated hospitalization in urban Thai children. Influenza Other Respir Viruses. 2007;1(5):177–82.

908. Rolfes MA, Olsen SJ, Kittikraisak W, Suntarattiwong P, Klungthong C, Ellison D, et al. Influenza-Associated Medical Visits Prevented by Influenza Vaccination in Young Children in Thailand, 2012-2014. Journal of the Pediatric Infectious Diseases Society. 2021;10(3):349–51.

909. Homaira N, Luby SP, Alamgir ASM, Islam K, Paul R, Abedin J, et al. Influenza-associated mortality in 2009 in four sentinel sites in Bangladesh. Bulletin of the World Health Organization. 2012;90(4):272–8.

910. Aungkulanon S, Cheng PY, Kusreesakul K, Bundhamcharoen K, Chittaganpitch M, Margaret M, et al. Influenza-associated mortality in Thailand, 2006-2011. Influenza and other Respiratory Viruses. 2015;9(6):298–304.

911. Zambon M. Influenza, respiratory syncytial virus and SARS. Medicine (Abingdon). 2005;33(5):130–4.

912. Millar J, Abdurrahman M, Toribio JA, Ambarawati A, Yusuf RP, Suadnya W. Informal inter-island poultry movement in Indonesia: does it pose a risk to HPAI H5N1 transmission? Tropical Animal Health and Production. 2015;47(7):1261–9.

913. Lambrou AS, Luitel H, Bhattarai RK, Basnet HB, Heaney CD. Informing influenza pandemic preparedness using commercial poultry farmer knowledge, attitudes, and practices (KAP) surrounding biosecurity and self-reported avian influenza outbreaks in Nepal. One Health [Internet]. 2020;11. Available from: https://www.scopus.com/inward/record.uri?eid=2-s2.0-85097183225&doi=10.1016%2fj.onehlt.2020.100189&partnerID=40&md5=0f3b4bf4bb58f2157a4b51282bf720ba

914. Shah B, Kaushik S. Innovative use of social media platform WhatsApp during influenza outbreak in Gujarat, India. WHO South East Asia J Public Health. 2015;4(2):213–4.

915. Turner JCM, Feeroz MM, Hasan MK, Akhtar S, Walker D, Seiler P, et al. Insight into live bird markets of Bangladesh: An overview of the dynamics of transmission of H5N1 and H9N2 avian influenza viruses. Emerging Microbes and Infections [Internet]. 2017;6(3). Available from: https://www.scopus.com/inward/record.uri?eid=2-s2.0-85014817245&doi=10.1038%2femi.2016.142&partnerID=40&md5=d54a1a3ed4d4c65b56130975d0786a3b

916. Auewarakul P, Hanchaoworakul W, Ungchusak K. Institutional responses to avian influenza in Thailand: Control of outbreaks in poultry and preparedness in the case of human-to-human transmission. Anthropology and Medicine. 2008;15(1):61–7.

917. Ansaldi F, Canepa P, Ceravolo A, Valle L, de Florentiis D, Oomen R, et al. Intanza(®) 15 mcg intradermal influenza vaccine elicits cross-reactive antibody responses against heterologous A(H3N2) influenza viruses. Vaccine. 2012;30(18):2908–13.

918. Xu X, Smith CB, Mungall BA, Lindstrom SE, Hall HE, Subbarao K, et al. Intercontinental circulation of human influenza A(H1N2) reassortant viruses during the 2001-2002 influenza season. J Infect Dis. 2002;186(10):1490–3.

919. Perez Arredondo AM, Bender K, Yasobant S, Bruchhausen W, Falkenberg T. Intersectoral collaboration shaping One Health in the policy agenda: A comparative analysis of Ghana and India. One Health. 2021;13:100272.

920. Apisarnthanarak A, Apisarnthanarak P, Cheevakumjorn B, Mundy LM. Intervention with an infection control bundle to reduce transmission of influenza-like illnesses in a thai preschool. Infection Control and Hospital Epidemiology. 2009;30(9):817–22.

921. Bahamondez-Canas TF, Cui Z. Intranasal immunization with dry powder vaccines. Eur J Pharm Biopharm. 2018;122:167–75.

922. Ichinohe T, Kawaguchi A, Tamura S ichi, Takahashi H, Sawa H, Ninomiya A, et al. Intranasal immunization with H5N1 vaccine plus Poly I:Poly C12U, a Toll-like receptor agonist, protects mice against homologous and heterologous virus challenge. Microbes Infect. 2007;9(11):1333–40.

923. Major D, Chichester JA, Pathirana RD, Guilfoyle K, Shoji Y, Guzman CA, et al. Intranasal vaccination with a plant-derived H5 HA vaccine protects mice and ferrets against highly pathogenic avian influenza virus challenge. Hum Vaccin Immunother. 2015;11(5):1235–43.

924. Khan SU, Berman LS, Haider N, Gerloff N, Rahman MZ, Shu B, et al. Investigating a crow die-off in January-February 2011 during the introduction of a new clade of highly pathogenic avian influenza virus H5N1 into Bangladesh. Archives of Virology. 2014;159(3):509–18.

925. Sakeena MHF, Bennett AA, Jamshed S, Mohamed F, Herath DR, Gawarammana I, et al. Investigating knowledge regarding antibiotics and antimicrobial resistance among pharmacy students in Sri Lankan universities. BMC Infectious Diseases [Internet]. 2018;18(1). Available from: https://www.scopus.com/inward/record.uri?eid=2-s2.0-85046687114&doi=10.1186%2fs12879-018-3107-8&partnerID=40&md5=96eaf5ba9a963bd7f3b7f061bdf386bd

926. Areechokchai D, Jiraphongsa C, Laosiritaworn Y, Hanshaoworakul W, O’Reilly M. Investigation of avian influenza (H5N1) outbreak in humans--Thailand, 2004. MMWR Morb Mortal Wkly Rep. 2006;55:3–6.

927. Setiawaty V, Isfandari S, Susilarini NK, Pangesti KA, Sedyaningsih ER. Investigations of close contacts of Patients with Laboratory-confirmed H5N1 Infection in Indonesia, in 2007. Am J Trop Med Hyg. 2009;81(5):213.

928. Kumar R, Nayak M, Sahoo GC, Pandey K, Sarkar MC, Ansari Y, et al. Iron oxide nanoparticles based antiviral activity of H1N1 influenza A virus. Journal of Infection and Chemotherapy. 2019;25(5):325–9.

929. Dhuria M, Ayub A, Ahmad S, Kumar P, Kumar A. Is india ready to address covid-19 like pandemics: A perspective from existing public health acts. Indian J Public Health Res Dev. 2020;11(11):119–25.

930. Javed D, Dixit AK. Is Trikatu; an ayurvedic formulation effective for the management of flu-like illness? A narrative review. J Complement Integr Med. 2021;

931. Indumathi CP, Gunanasekaran P, Kaveri K, Arunagiri K, Mohana S, Khaleefathullah Sheriff A, et al. Isolation & Molecular characterization of human parainfluenza virus in Chennai, India. Indian J Med Res. 2015;142:583–90.

932. Jakhesara SJ, Bhatt VD, Patel NV, Prajapati KS, Joshi CG. Isolation and characterization of H9N2 influenza virus isolates from poultry respiratory disease outbreak. Springerplus. 2014;3:196.

933. Dubey SC, Dahal N, Nagarajan S, Tosh C, Murugkar HV, Rinzin K, et al. Isolation and characterization of influenza A virus (subtype H5N1) that caused the first highly pathogenic avian influenza outbreak in chicken in Bhutan. Veterinary Microbiology. 2012;155(1):100–5.

934. Thawatsupha P, Waicharoen S, Maneewong P, Prasittikhet K, Chittaganapitch M, Sawanpanyalert P. Isolation and identification of influenza virus strains circulating in Thailand in 2001. Southeast Asian Journal of Tropical Medicine and Public Health. 2003;34(1):94–7.

935. Nagarajan S, Tosh C, Murugkar HV, Venkatesh G, Katare M, Jain R, et al. Isolation and molecular characterization of a H5N1 virus isolated from a Jungle crow (Corvus macrohynchos) in India. Virus Genes. 2010;41(1):30–6.

936. Nagarajan S, Rajukumar K, Tosh C, Ramaswamy V, Purohit K, Saxena G, et al. Isolation and pathotyping of H9N2 avian influenza viruses in Indian poultry. Veterinary Microbiology. 2009;133(1):154–63.

937. Gowthaman V, Singh SD, Dhama K, Srinivasan P, Saravanan S, Murthy TRGK, et al. Isolation and phylogenetic characterization of haemagglutinin and neuraminidase genes of H9N2 low pathogenicity avian influenza virus isolated from commercial layers in India. VirusDisease. 2016;27(4):382–6.

938. Promkuntod N, Antarasena C, Prommuang P, Prommuang P. Isolation of avian influenza virus A subtype H5N1 from internal contents (albumen and allantoic fluid) of Japanese quail (Coturnix coturnix japonica) eggs and oviduct during a natural outbreak. Ann N Y Acad Sci. 2006;1081:171–3.

939. Kadam SS, Rao BL. Isolation of recent variant influenza types A (H3N2) and B strains in Pune, India during 1998. Indian J Med Res. 2000;111:3–5.

940. Rao BL, Kadam SS, Pawar MS. Isolation of recent variant influenza types A(H3N2), A (H1N1) & B strains in Pune, India. Indian J Med Res. 2001;114:157–9.

941. Hiromoto Y, Parchariyanon S, Ketusing N, Netrabukkana P, Hayashi T, Kobayashi T, et al. Isolation of the Pandemic (H1N1) 2009 virus and its reassortant with an H3N2 swine influenza virus from healthy weaning pigs in Thailand in 2011. Virus Research. 2012;169(1):175–81.

942. Gupta YK, Padhy BM. Issues in pharmacotherapy of 2009 H1N1 influenza infection. Journal of Postgraduate Medicine. 2010;56(4):321–7.

943. Wheelock A, Miraldo M, Parand A, Vincent C, Sevdalis N. Journey to vaccination: a protocol for a multinational qualitative study. BMJ Open. 2014;4(1):e004279.

944. Le Menach A, Vergu E, Grais RF, Smith DL, Flahault A. Key strategies for reducing spread of avian influenza among commercial poultry holdings: lessons for transmission to humans. Proc Biol Sci. 2006;273(1600):2467–75.

945. Kitphati R, Pooruk P, Lerdsamran H, Poosuwan S, Louisirirotchanakul S, Auewarakul P, et al. Kinetics and Longevity of Antibody Response to Influenza a H5N1 Virus Infection in Humans. Clinical and Vaccine Immunology. 2009;16(7):978–81.

946. Sood N, Dharsandia M, Patankar M, Vegad M. Kinetics of pandemic influenza virus 2009 virus in Gujarat, Western India: An investigation of signature features. Annals of Tropical Medicine and Public Health. 2012;5(4):321–6.

947. Kositanont U, Assantachai P, Wasi C, Puthavathana P, Praditsuwan R. Kinetics of the antibody response to seasonal influenza vaccination among the elderly. Viral Immunology. 2012;25(6):471–6.

948. Hickey JE, Gagnon AJ, Jitthai N. Knowledge about pandemic influenza preparedness among vulnerable migrants in Thailand. Health Promotion International. 2016;31(1):124–32.

949. Gambhir RS, Pannu PR, Nanda T, Arora G, Kaur A. Knowledge and awareness regarding swine-influenza a (H1n1) virus infection among dental professionals in India - A systematic review. J Clin Diagn Res. 2016;10(9):ZE10–3.

950. Kurscheid J, Millar J, Abdurrahman M, Suadnya W, Ambarawati IGAA, Yusuf RP, et al. Knowledge and perceptions of Highly Pathogenic Avian Influenza (HPAI) among poultry traders in live bird markets in Bali and Lombok, Indonesia. PLoS ONE. 2015;10(10):e0139917.

951. Shrestha R, Shrestha KB, Ghimire S, Shrestha N. Knowledge and Preventive Practices related to Avian Influenza among Poultry Workers of Kamalamai Municipality, Sindhuli, Nepal. Journal of Nepal Health Research Council. 2016;14(32):7–12.

952. Kumar N, Sood S, Singh M, Kumar M, Makkar B, Singh M. Knowledge of swine flu among health care workers and general population of Haryana, India during 2009 pandemic. Australasian Medical Journal. 2010;3(9):614–7.

953. Nath B, Midha T, Kumari R, Gupta S. Knowledge, Attitude and practice regarding influenza a (H1N1) among senior secondary school students of Kanpur city in north India. Indian Journal of Community Health. 2014;26(3):303–7.

954. Puri S, Singh A, Koushal V, Thakare M, Singhal A. Knowledge, attitude and practice regarding the H1N1 pandemic amongst healthcare providers, and preparedness in a multispeciality teaching hospital in North India. Public Health. 2011;125(11):795–8.

955. Datta SS, Kuppuraman D, Boratne AV, Abraham SB, Singh Z. Knowledge, attitude and practices regarding Swine flu among para-medical workers in a tertiary care hospital in Pondicherry. Journal of Communicable Diseases. 2011;43(1):1–9.

956. Hossain SM, Eusufzai SZ, Elahi MM, Jamayet NB. Knowledge, attitude and practices related to AI among poultry workers of Bangladesh. Bangladesh Journal of Medical Science. 2015;14(1):26–31.

957. Suresh A, Sruthi N, Krishnan K, Hariharan V, Karthikeyan LV, Anitha S. Knowledge, attitude and practices towards swine flu among school students of Nilgiris, Tamil Nadu. Journal of Communicable Diseases. 2019;51(4):41–6.

958. Koul PA, Bali NK, Sonawane S. Knowledge, attitude, and behavioural response of corporate employees in India towards influenza: A questionnaire based study. Journal of Association of Physicians of India. 2016;64:44–50.

959. Bali NK, Ashraf M, Ahmad F, Khan UH, Widdowson M. A, Lal RB, et al. Knowledge, attitude, and practices about the seasonal influenza vaccination among healthcare workers in Srinagar, India. Influenza and other Respiratory Viruses. 2013;7(4):540–5.

960. Parathasarathy CR, Chitra Rajalaksmi P, Jeyaseelan Senthinath T, Revathi P, Uma A, Ismail M, et al. Knowledge, attitude, behavior and practices on H1N1 among the heterogenous population of Tamil Nadu, India. BMC Proc. 2011;5.

961. Singh K, Bhat N, Chaudhary H, Asawa K, Sharda A, Agrawal A. Knowledge, attitude, behavioural response and use of preventive measures regarding pandemic H1N1 influenza outbreak among dental students in Udaipur city, India. Oral Health Prev Dent. 2012;10(4):339–44.

962. Thanee C, Kittikraisak W, Sinthuwattanawibool C, Roekworachai K, Klinklom A, Kornsitthikul K, et al. Knowledge, attitude/perception, and practice related to seasonal influenza vaccination among caregivers of young Thai children: A cross-sectional study. PLoS One. 2021;16(6):e0253561.

963. Ditsungnoen D, Greenbaum A, Praphasiri P, Dawood FS, Thompson MG, Yoocharoen P, et al. Knowledge, attitudes and beliefs related to seasonal influenza vaccine among pregnant women in Thailand. Vaccine. 2016;34(18):2141–6.

964. Rukmanee N, Yimsamran S, Rukmanee P, Thanyavanich N, Maneeboonyang W, Puangsa-art S, et al. Knowledge, attitudes and practices (kap) regarding influenza A (H1N1) among a population living along Thai-Myanmar border, Ratchaburi Province, Thailand. Southeast Asian J Trop Med Public Health. 2014;45(4):825–33.

965. Azizur Rahman M, Karim R. Knowledge, attitudes and practices regarding highly pathogenic avian influenza among adult population of Bangladesh. Indian Journal of Public Health Research and Development. 2013;4(3):159–63.

966. Neupane D, Khanal V, Ghimire K, Aro AR, Leppin A. Knowledge, attitudes and practices related to avian influenza among poultry workers in Nepal: A cross sectional study. BMC Infectious Diseases [Internet]. 2012;12. Available from: https://www.scopus.com/inward/record.uri?eid=2-s2.0-84859085052&doi=10.1186%2f1471-2334-12-76&partnerID=40&md5=43a9bc87b9d54ff866adbac190263278

967. Kumar SC, Ramesh N, Sreevatsan S, Joseph B, Alle P, Belani KG, et al. Knowledge, attitudes, and poultry-handling practices of poultry workers in relation to avian influenza in India. Indian Journal of Occupational and Environmental Medicine. 2013;17(1):16–21.

968. Sharma R, Kaur S, Sodhi A. Knowledge, behaviour change, and anticipated compliance regarding non-pharmaceutical interventions during pandemic of influenza A H1N1 in Delhi. Lung India. 2012;29(4):341–6.

969. Mishra P, Bhadauria US, Dasar PL, N S, Kumar S, Lalani A, et al. Knowledge,attitude and anxiety towards pandemic flu a potential bio weapon among health professionals in Indore City. Przegla̧d epidemiologiczny. 2016;70(1):41–5, 125–7.

970. Wertheim HFL, Puthavathana P, Nghiem NM, Rogier van Doorn H, Nguyen TV, Pham HV, et al. Laboratory capacity building in Asia for infectious disease research: Experiences from the South East Asia Infectious Disease Clinical Research Network (SEAICRN). PLoS Medicine [Internet]. 2010;7(4). Available from: https://www.scopus.com/inward/record.uri?eid=2-s2.0-77951733997&doi=10.1371%2fjournal.pmed.1000231&partnerID=40&md5=39496801c3338c37a62e5efc4b62539e

971. Potdar V, Hinge D, Satav A, Simões EF, Yadav PD, Chadha MS. Laboratory-confirmed avian influenza A(H9N2) virus infection, India, 2019. Emerging Infectious Diseases. 2019;25(12):2328–30.

972. Bloom-Feshbach K, Alonso WJ, Charu V, Tamerius J, Simonsen L, Miller MA, et al. Latitudinal variations in seasonal activity of influenza and respiratory syncytial virus (RSV): a global comparative review. PLoS One. 2013;8(2):e54445.

973. Isaac BTJ, Kirupakaran H, Barney AM, Christopher DJ. Lessons from healthcare personnel screening and management during H1N1 pandemic in preparation for the impending COVID-19 pandemic in a tertiary care hospital in India. Indian Journal of Tuberculosis. 2020;67(4):S122–7.

974. Chakraborti C. Lessons from the response to A H1N1 influenza, 2009, India: ethics in pandemic planning. Indian journal of medical ethics. 2010;7(4):216–9.

975. Tallis G. Lessons learned from emerging infectious diseases in Indonesia. Intern Med J. 2010;40:54.

976. Ungchusak K, Sawanpanyalert P, Hanchoworakul W, Sawanpanyalert N, Maloney SA, Brown RC, et al. Lessons learned from influenza A(H1N1)pdm09 pandemic response in Thailand. Emerging Infectious Diseases. 2012;18(7):1058–64.

977. Kshatriya RM, Khara NV, Ganjiwale J, Lote SD, Patel SN, Paliwal RP. Lessons learnt from the Indian H1N1 (swine flu) epidemic: Predictors of outcome based on epidemiological and clinical profile. J Family Med Prim Care. 2018;7(6):1506–9.

978. Steensels M, Van Borm S, Boschmans M, Van Den Berg T. Lethality and molecular characterization of an HPAI H5N1 virus isolated from eagles smuggled from Thailand into Europe. Avian Dis. 2007;51:401–7.

979. Govorkova EA, Rehg JE, Krauss S, Yen HL, Guan Y, Peiris M, et al. Lethality to ferrets of H5N1 influenza viruses isolated from humans and poultry in 2004. J Virol. 2005;79(4):2191–8.

980. Wiwanitkit V. Leucocyte and lymphocyte count in cases of bird flu infection in Thailand. Journal of the Indian Medical Association [Internet]. 2008;106(3). Available from: https://www.scopus.com/inward/record.uri?eid=2-s2.0-49149094593&partnerID=40&md5=75cc8b3f52110b9ff934d4d384e59ea9

981. Indrawan D, Rich KM, van Horne P, Daryanto A, Hogeveen H. Linking supply chain governance and biosecurity in the context of HPAI control in western java: A value chain perspective. Frontiers in Veterinary Science [Internet]. 2018;5. Available from: https://www.scopus.com/inward/record.uri?eid=2-s2.0-85046845226&doi=10.3389%2ffvets.2018.00094&partnerID=40&md5=f07eb37a625f1bcff4a514ee4332f46d

982. Hassan MM, Hoque MA, Ujvari B, Klaassen M. Live bird markets in Bangladesh as a potentially important source for Avian Influenza Virus transmission. Preventive Veterinary Medicine. 2018;156:22–7.

983. Negovetich NJ, Walker D, Seiler P, Ferguson A, Friedman K, Barman S, et al. Live bird markets of bangladesh: H9n2 viruses and the near absence of highly pathogenic h5n1 influenza. PLoS ONE. 2011;6(4):e19311.

984. Poudel U, Dahal U, Upadhyaya N, Chaudhari S, Dhakal S. Livestock and poultry production in Nepal and current status of vaccine development. Vaccines. 2020;8(2):1–9.

985. Gillard P, Chu DWS, Hwang SJ, Yang PC, Thongcharoen P, Lim FS, et al. Long-term booster schedules with AS03A-adjuvanted heterologous H5N1 vaccines induces rapid and broad immune responses in Asian adults. BMC Infectious Diseases [Internet]. 2014;14(1). Available from: https://www.scopus.com/inward/record.uri?eid=2-s2.0-84899098483&doi=10.1186%2f1471-2334-14-142&partnerID=40&md5=d3385c07e6134e59925d4264974acb5e

986. Moolasart V, Manosuthi W, Ausavapipit J, Chottanapund S, Likanonsakul S, Uttayamakul S, et al. Long-term seroprotective response of trivalent seasonal influenza vaccine in HIV-infected children, regardless of immunogenicity before immunisation. International Journal of STD and AIDS. 2016;27(9):761–8.

987. Hinjoy S, Puthavathana P, Laosiritaworn Y, Limpakarnjanarat K, Pooruk P, Chuxnum T, et al. Low frequency of infection with avian influenza virus (H5N1) among poultry farmers, Thailand, 2004. Emerging Infectious Diseases. 2008;14(3):499–501.

988. Kode SS, Pawar SD, Tandale BV, Parkhi SS, Barde TD, Mishra AC. Low level of cross-reactive antibodies to pandemic influenza (H1N1) 2009 virus in humans in pre-pandemic period in Maharashtra, India. Indian Journal of Virology. 2012;23(1):36–8.

989. Sukprasert S, Pansuksan K, Sriyakul K. Lysiphyllum strychnifolium (Craib) A. Schmitz extract, a novel neuraminidase inhibitor of avian influenza virus subtype H5N1. Journal of Herbal Medicine [Internet]. 2020;20. Available from: https://www.scopus.com/inward/record.uri?eid=2-s2.0-85077920026&doi=10.1016%2fj.hermed.2020.100330&partnerID=40&md5=27e83ed9367be2484e474ad0ebdec71b

990. Smith RD, Keogh-Brown MR. Macroeconomic impact of a mild influenza pandemic and associated policies in Thailand, South Africa and Uganda: A computable general equilibrium analysis. Influ Other Respir Viruses. 2013;7(6):1400–8.

991. Smith RD, Keogh-Brown MR. Macroeconomic impact of pandemic influenza and associated policies in Thailand, South Africa and Uganda. Influ Other Respir Viruses. 2013;7:64–71.

992. Dauphin G, Hamilton K, Kim LM, Choudhury B, Capua I, Edwards S. Main achievements of the World Organisation for Animal Health/United Nations Food and Agriculture Organization network on animal influenza. Avian Dis. 2010;54(1):380–3.

993. Hunt HD, Jadhao S, Swayne DE. Major histocompatibility complex and background genes in chickens influence susceptibility to high pathogenicity avian influenza virus. Avian Dis. 2010;54(1):572–5.

994. Chowdhury S, Aleem MA, Khan MSI, Hossain ME, Ghosh S, Rahman MZ. Major zoonotic diseases of public health importance in Bangladesh. Veterinary Medicine and Science. 2021;7(4):1199–210.

995. Anand R, Gupta A, Gupta A, Wadhawan S, Bhadoria P. Management of swine-flu patients in the intensive care unit: Our experience. Journal of Anaesthesiology Clinical Pharmacology. 2012;28(1):51–5.

996. Kosasih H, Bratasena A, Pangesti K, Laras K, Samaan G. Managing seasonal influenza: oseltamivir treatment policy in indonesia? Acta medica Indonesiana. 2014;46(1):58–65.

997. Gilbert M, Xiao X, Boles S, Czarnecki C, Pfeiffer DU, Epprecht M, et al. Mapping H5N1 highly pathogenic avian influenza risk in Southeast Asia. Proc Natl Acad Sci U S A. 2008;105(12):4769–74.

998. Marchand DK, Argáez C. Masks for Prevention of Influenza Transmission in Acute and Long-Term Care Settings: A Review of Clinical Effectiveness, Cost-Effectiveness and Guidelines. Ottawa (ON): Canadian Agency for Drugs and Technologies in Health; 2020.

999. Desheva Y, Mamontov A, Petkova N, Karev V, Nazarov P. Mast cell degranulation and histamine release during A/H5N1 influenza infection in influenza-sensitized mice. Life Sci. 2020;258:118230.

1000. Aqil AR, Clark DR, Omer SB, Tapia MD, Nunes MC, Kozuki N, et al. Maternal Influenza Immunization and Prevention of Severe Clinical Pneumonia in Young Infants: Analysis of Randomized Controlled Trials Conducted in Nepal, Mali and South Africa. Pediatr Infect Dis J. 2018;37(5):436–40.

1001. Sharma R, Karad AB, Dash B, Dhariwal AC, Chauhan LS, Lal S. Media scanning and verification system as a supplemental tool to disease outbreak detection & reporting at National Centre for Disease Control, Delhi. Journal of Communicable Diseases. 2012;44(1):9–14.

1002. Vimalanathan S, Ignacimuthu S, Hudson JB. Medicinal plants of Tamil Nadu (Southern India) are a rich source of antiviral activities. Pharmaceutical Biology. 2009;47(5):422–9.

1003. Kala CP. Medicinal plants used for the treatment of respiratory diseases in Uttarakhand state of India. Studies on Ethno-Medicine. 2020;14(1):1–8.

1004. Farnsworth ML, Fitchett S, Hidayat MM, Lockhart C, Hamilton-West C, Brum E, et al. Metapopulation dynamics and determinants of H5N1 highly pathogenic avian influenza outbreaks in Indonesian poultry. Preventive Veterinary Medicine. 2011;102(3):206–17.

1005. Chakraborty A, Rahman M, Hossain MJ, Khan SU, Haider MS, Sultana R, et al. Mild respiratory illness among young children caused by highly pathogenic avian influenza a (h5n1) virus infection in Dhaka, Bangladesh, 2011. Journal of Infectious Diseases. 2017;216:S520–8.

1006. Chawla R, Sharma RK, Madaan D, Dubey N, Arora R, Goel R, et al. Mitigation approaches to combat the flu pandemic. J Glob Infect Dis. 2009;1(2):117–30.

1007. Biswas PK, Islam MZ, Debnath NC, Yamage M. Modeling and roles of meteorological factors in outbreaks of highly pathogenic avian influenza H5N1. PLoS One. 2014;9(6):e98471.

1008. Stevens KB, Gilbert M, Pfeiffer DU. Modeling habitat suitability for occurrence of highly pathogenic avian influenza virus H5N1 in domestic poultry in Asia: a spatial multicriteria decision analysis approach. Spat Spatiotemporal Epidemiol. 2013;4:1–14.

1009. Pandit PS, Bunn DA, Pande SA, Aly SS. Modeling highly pathogenic avian influenza transmission in wild birds and poultry in West Bengal, India. Sci Rep. 2013;3:2175.

1010. Chadsuthi S, Iamsirithaworn S, Triampo W, Modchang C. Modeling Seasonal Influenza Transmission and Its Association with Climate Factors in Thailand Using Time-Series and ARIMAX Analyses. Computational and Mathematical Methods in Medicine [Internet]. 2015;2015. Available from: https://www.scopus.com/inward/record.uri?eid=2-s2.0-84948799649&doi=10.1155%2f2015%2f436495&partnerID=40&md5=03aee5818a46dc72bc6cd2faf65fef2c

1011. Wiratsudakul A, Paul MC, Bicout DJ, Tiensin T, Triampo W, Chalvet-Monfray K. Modeling the dynamics of backyard chicken flows in traditional trade networks in Thailand: Implications for surveillance and control of avian influenza. Tropical Animal Health and Production. 2014;46(5):845–53.

1012. Rao ASRS. Modeling the rapid spread of avian influenza (H5N1) in India. Math Biosci Eng. 2008;5(3):523–37.

1013. Upadhyay RK, Kumari N, Rao VSH. Modeling the spread of bird flu and predicting outbreak diversity. Nonlinear Anal Real World Appl. 2008;9(4):1638–48.

1014. Colizza V, Barrat A, Barthelemy M, Valleron AJ, Vespignani A. Modeling the worldwide spread of pandemic influenza: baseline case and containment interventions. PLoS Med. 2007;4(1):e13.

1015. Hill EM, House T, Dhingra MS, Kalpravidh W, Morzaria S, Osmani MG, et al. Modelling H5N1 in Bangladesh across spatial scales: Model complexity and zoonotic transmission risk. Epidemics. 2017;20:37–55.

1016. Rahman M, Hoque SA, Islam MA, Rahman SR. Molecular analysis of amantadine-resistant influenza A (H1N1 pdm09) virus isolated from slum dwellers of Dhaka, Bangladesh. Virus Genes. 2017;53(3):377–85.

1017. Mahardika GN, Jonas M, Murwijati T, Fitria N, Suartha IN, Suartini IGAA, et al. Molecular analysis of hemagglutinin-1 fragment of avian influenza H5N1 viruses isolated from chicken farms in Indonesia from 2008 to 2010. Veterinary Microbiology. 2016;186:52–8.

1018. Jain M, Islam S, Rahman ASMZ, Akhtar S, Hasan KN, Ahsan GU, et al. Molecular analysis of hemagglutinin, neuraminidase, matrix genes provide insight into the genetic diversity of seasonal H3N2 human influenza a viruses in Bangladesh during July–August, 2012. VirusDisease. 2018;29(1):54–60.

1019. Pandey S, Sahu M, Potdar V, Barde P. Molecular analysis of influenza A H1N1pdm09 virus circulating in Madhya Pradesh, India in the year 2017. VirusDisease. 2018;29(3):380–4.

1020. Canas LC, Arulanandam BP, Chambers JP, Klimov AI, Shaw MW, Gibbons RV, et al. Molecular analysis of isolates from influenza B outbreaks in the U.S. and Nepal, 2005. Arch Virol. 2006;151(9):1863–74.

1021. Sahu M, Singh N, Shukla MK, Potdar VA, Sharma RK, Sahare LK, et al. Molecular and epidemiological analysis of pandemic and post-pandemic influenza A(H1N1)pdm09 virus from central India. Journal of Medical Virology. 2018;90(3):447–55.

1022. Aras S, Aiyar A, Amedee AM, Gallaher WR. Molecular character of influenza A/H1N1 2009: Implications for spread and control. Indian J Microbiol. 2009;49(4):339–47.

1023. Chutinimitkul S, Chieochansin T, Payungporn S, Samransamruajkit R, Hiranras T, Theamboonlers A, et al. Molecular characterization and phylogenetic analysis of H1N1 and H3N2 human influenza A viruses among infants and children in Thailand. Virus Research. 2008;132(1):122–31.

1024. Dangi T, Jain B, Singh AK, Singh JV, Kumar R, Dwivedi M, et al. Molecular characterization of circulating pandemic strains of influenza A virus during 2012 to 2013 in Lucknow (India). Journal of Medical Virology. 2014;86(12):2134–41.

1025. Qureshi M, Tosh C, Nagarajan S, Murugkar HV, Jain R, Kulkarni DD. Molecular characterization of H5N1 avian influenza virus isolated in Tripura, 2011. Indian Journal of Animal Sciences. 2013;83(5):521–4.

1026. Sahu M, Shukla MK, Barde PV. Molecular characterization of human respiratory syncytial virus detected from central India. Journal of Medical Virology. 2017;89(10):1871–4.

1027. Biswas D, Yadav K, Borkakoty B, Mahanta J. Molecular characterization of human respiratory syncytial virus NA1 and GA5 genotypes detected in Assam in northeast India, 2009-2012. Journal of Medical Virology. 2013;85(9):1639–44.

1028. Saha P, Biswas M, Gupta R, Majumdar A, Mitra S, Banerjee A, et al. Molecular characterization of Influenza A pandemic H1N1 viruses circulating in eastern India during 2017–19: Antigenic diversity in comparison to the vaccine strains. Infection, Genetics and Evolution [Internet]. 2020;81. Available from: https://www.scopus.com/inward/record.uri?eid=2-s2.0-85081018610&doi=10.1016%2fj.meegid.2020.104270&partnerID=40&md5=8517bba1b1db3422d51f3e789876009b

1029. Potdar V, Hinge D, Chadha M, Vijay N, Kaur H, Gupta N, et al. Molecular characterization of influenza A(H1N1)pdm09 viruses circulating at various geographical locations in India, 2017. Indian J Med Res. 2019;149(6):783–9.

1030. Kuypers J, Chu HY, Gaydos CA, Katz J, Khatry SK, LeClerq SC, et al. Molecular characterization of influenza viruses from women and infants in Sarlahi, Nepal. Diagnostic Microbiology and Infectious Disease. 2019;93(4):305–10.

1031. Jagadesh A, Salam AAA, Zadeh VR, Krishnan A, Arunkumar G. Molecular characterization of neuraminidase genes of influenza A(H3N2) viruses circulating in Southwest India from 2009 to 2013. Archives of Virology. 2017;162(7):1887–902.

1032. Boonnak K, Mansanguan C, Schuerch D, Boonyuen U, Lerdsamran H, Jiamsomboon K, et al. Molecular characterization of seasonal influenza a and b from hospitalized patients in thailand in 2018–2019. Viruses [Internet]. 2021;13(6). Available from: https://www.scopus.com/inward/record.uri?eid=2-s2.0-85107353458&doi=10.3390%2fv13060977&partnerID=40&md5=9c1d427518268b61fcadf1b797f6f29e

1033. Puthavathana P, Auewarakul P, Charoenying PC, Sangsiriwut K, Pooruk P, Boonnak K, et al. Molecular characterization of the complete genome of human influenza H5N1 virus isolates from Thailand. J Gen Virol. 2005;86(2):423–33.

1034. Potdar VA, Hinge DD, Dakhave MR, Manchanda A, Jadhav N, Kulkarni PB, et al. Molecular detection and characterization of Influenza ‘C’ viruses from western India. Infection, Genetics and Evolution. 2017;54:466–77.

1035. Parida M, Shukla J, Sharma S, Rao PVL. Molecular detection and characterization of novel pandemic Swine flu H1N1 virus from India during 2009-2010 epidemics. J Antivirals Antiretrovirals [Internet]. 2011;2011. Available from: http://www.omicsonline.org/1948-5964/1948-5964.S1.8-12.pdf

1036. Gaur P, Srivastava N, Awasthi S, Katiyar R, Srivastava NN, Singh DV, et al. Molecular detection of human rhinovirus in respiratory samples of swine flu negative north Indian children with flu-like illness. Asian Journal of Pharmaceutical and Clinical Research. 2016;9(1):254–6.

1037. Uchida Y, Chaichoune K, Wiriyarat W, Watanabe C, Hayashi T, Patchimasiri T, et al. Molecular epidemiological analysis of highly pathogenic avian influenza H5N1 subtype isolated from poultry and wild bird in Thailand. Virus Research. 2008;138(1):70–80.

1038. Upadhyay BP, Ghimire P, Tashiro M, Banjara MR. Molecular Epidemiology and Antigenic Characterization of Seasonal Influenza Viruses Circulating in Nepal. Journal of Nepal Health Research Council. 2017;15(35):44–50.

1039. Sharma S, Joshi G, Dash PK, Athmaram TN, Kumar JS, Rao PVL, et al. Molecular Epidemiology and Complete Genome Characterization of H1N1pdm Virus from India. PLoS ONE. 2013;8(2):e56364.

1040. Tewawong N, Suwannakarn K, Prachayangprecha S, Korkong S, Vichiwattana P, Vongpunsawad S, et al. Molecular epidemiology and phylogenetic analyses of influenza B virus in thailand during 2010 to 2014. PLoS ONE. 2015;10(1):e0116302.

1041. Prabu D, Karunya D, Sree Nethra B, Kiruthika K, Vidhya NM, Anusha H, et al. Molecular epidemiology of acute respiratory viral infections among children in Chennai, South India. BMC Infect Dis. 2020;20.

1042. Ahmed SSU, Themudo GE, Christensen JP, Biswas PK, Giasuddin M, Samad MA, et al. Molecular epidemiology of circulating highly pathogenic avian influenza (H5N1) virus in chickens, in Bangladesh, 2007-2010. Vaccine. 2012;30(51):7381–90.

1043. Guan Y, Smith GJD, Webby R, Webster RG. Molecular epidemiology of H5N1 avian influenza. Rev Sci Tech. 2009;28(1):39–47.

1044. Hoque MA, Tun HM, Hassan MM, Khan SA, Islam SKMA, Islam MN, et al. Molecular epidemiology of influenza A (H5N1) viruses, Bangladesh, 2007-2011. Preventive Veterinary Medicine. 2013;111(3):314–8.

1045. Cheung CL, Yeung MF, Poon LLM, Guan Y, Peiris M, Perera HKK, et al. Molecular epidemiology of influenza A(H1N1) pdm09 virus among humans and swine, Sri Lanka. Emerg Infect Dis. 2014;20(12):2080–4.

1046. Sharma S, Parida M, Shukla J, Rao PVL. Molecular epidemiology of novel swine origin influenza virus (S-OIV) from Gwalior, India, 2009. Virology Journal [Internet]. 2011;8. Available from: https://www.scopus.com/inward/record.uri?eid=2-s2.0-79957939715&doi=10.1186%2f1743-422X-8-280&partnerID=40&md5=4186e7ebee229eb2692eb578eb762bb7

1047. Sharma S, Mayank A, Lal SK. Molecular events leading to the creation of a pandemic influenza virus. Indian Journal of Microbiology. 2009;49(4):332–8.

1048. Haque ME, Giasuddin M, Chowdhury EH, Islam MR. Molecular evolution of H5N1 highly pathogenic avian influenza viruses in Bangladesh between 2007 and 2012. Avian Pathology. 2014;43(2):183–94.

1049. Suwannakarn K, Amonsin A, Sasipreeyajan J, Kitikoon P, Tantilertcharoen R, Parchariyanon S, et al. Molecular evolution of H5N1 in Thailand between 2004 and 2008. Infection, Genetics and Evolution. 2009;9(5):896–902.

1050. Suwannakarn K, Chieochansin T, Thongmee C, Makkoch J, Praianantathavorn K, Theamboonlers A, et al. Molecular evolution of human H1N1 and H3N2 influenza A virus in Thailand, 2006-2009. PLoS ONE. 2010;5(3):e9717.

1051. Neumann G, Shinya K, Kawaoka Y. Molecular pathogenesis of H5N1 influenza virus infections. Antivir Ther. 2007;12(4):617–26.

1052. Hindupur A, Menon T, Dhandapani P. Molecular surveillance of respiratory viruses in children with acute respiratory infections in Chennai, South India. Int J Infect Dis. 2020;101:515.

1053. Biswas D, Yadav K, Lahon M, Borkakoty B, Mahanta J. Monitoring of human influenza virus activity in northeast India with a focus on Pandemic H1N1/2009. Trop Med Int Health. 2011;16:79.

1054. Modi M, Madhavani K, Patel N, Javadekar T. Morbidity by Influenza A (Novel H1N1) virus infection in relation to age and gender from january 01, 2015 to october 30, 2015 in Baroda, a city in western India. Int J Infect Dis. 2016;45:232.

1055. Cooper BS, Kotirum S, Kulpeng W, Praditsitthikorn N, Chittaganpitch M, Limmathurotsakul D, et al. Mortality attributable to seasonal influenza A and B infections in Thailand, 2005-2009: A longitudinal study. American Journal of Epidemiology. 2015;181(11):898–907.

1056. Sharma V, Verma PK, Gupta S, Sharma A. Mortality from influenza A/H1N1 in a tertiary care teaching institution in North India. Journal of Infection in Developing Countries. 2010;4(8):468–71.

1057. Chandra S. Mortality from the influenza pandemic of 1918-19 in Indonesia. Popul Stud. 2013;67(2):185–93.

1058. Chandra S, Kuljanin G, Wray J. Mortality From the Influenza Pandemic of 1918-1919: The Case of India. Demography. 2012;49(3):857–65.

1059. Biswas PK, Barua H, Rahman MH, Debnath NC, Christensen JP, Ahmed SSU, et al. Mortality rate and clinical features of highly pathogenic avian influenza in naturally infected chickens in Bangladesh. OIE Rev Sci Tech. 2011;30(3):871–8.

1060. Gulati P, Saini L, Jawa A, Das CJ. MRI in H1N1 encephalitis. Indian Journal of Pediatrics. 2013;80(2):157–9.

1061. Yasobant S, Saxena D, Memon FZ, Bruchhausen W, Falkenberg T. Multi-sectoral prioritization of zoonotic diseases: One health perspective from Ahmedabad, India. PLoS ONE. 2019;14(7):e0220152.

1062. Hadifar F, Ignjatovic J, Tarigan S, Indriani R, Ebrahimie E, Hasan NH, et al. Multimeric recombinant M2e protein-based ELISA: a significant improvement in differentiating avian influenza infected chickens from vaccinated ones. PLoS One. 2014;9(10):e108420.

1063. Maines TR, Chen LM, Belser JA, Van Hoeven N, Smith E, Donis RO, et al. Multiple genes contribute to the virulent phenotype observed in ferrets of an H5N1 influenza virus isolated from Thailand in 2004. Virology. 2011;413(2):226–30.

1064. Tosh C, Nagarajan S, Kumar M, Murugkar HV, Venkatesh G, Shukla S, et al. Multiple introductions of a reassortant H5N1 avian influenza virus of clade 2.3.2.1c with PB2 gene of H9N2 subtype into Indian poultry. Infection, Genetics and Evolution. 2016;43:173–8.

1065. Marinova-Petkova A, Feeroz MM, Rabiul Alam S, Kamrul Hasan M, Akhtar S, Jones-Engel L, et al. Multiple introductions of highly pathogenic avian influenza H5N1 viruses into Bangladesh. Emerging Microbes and Infections [Internet]. 2014;3. Available from: https://www.scopus.com/inward/record.uri?eid=2-s2.0-84894120559&doi=10.1038%2femi.2014.11&partnerID=40&md5=cb8febbd211a2f4bc5bec9883705fc4d

1066. Gerloff NA, Khan SU, Balish A, Shanta IS, Simpson N, Berman L, et al. Multiple reassortment events among highly pathogenic avian influenza A(H5N1) viruses detected in Bangladesh. Virology. 2014;450:297–307.

1067. Adam K, Pangesti KNA, Setiawaty V. Multiple Viral Infection Detected from Influenza-Like Illness Cases in Indonesia. BioMed Research International [Internet]. 2017;2017. Available from: https://www.scopus.com/inward/record.uri?eid=2-s2.0-85012191621&doi=10.1155%2f2017%2f9541619&partnerID=40&md5=4ee93e106c2f0f7ff6b9bbfe0476e824

1068. Mahony JB, Hatchette T, Ojkic D, Drews SJ, Gubbay J, Low DE, et al. Multiplex PCR tests sentinel the appearance of pandemic influenza viruses including H1N1 swine influenza. J Clin Virol. 2009;45(3):200–2.

1069. Chadha MS, Broor S, Gunasekaran P, Potdar VA, Krishnan A, Chawla-Sarkar M, et al. Multisite virological influenza surveillance in India: 2004-2008. Influenza and other Respiratory Viruses. 2012;6(3):196–203.

1070. Kreijtz JHCM, Suezer Y, de Mutsert G, van Amerongen G, Schwantes A, van den Brand JMA, et al. MVA-based H5N1 vaccine affords cross-clade protection in mice against influenza A/H5N1 viruses at low doses and after single immunization. PLoS One. 2009;4(11):e7790.

1071. Tariyo S, Paengta S, Wongsaen R, Thanompan S. Mycoplasma pneumoniae outbreak in a medical record department at a tertiary care hospital, northern thailand. Antimicrob Resist Infect Control. 2017;6.

1072. Li Y, Johnson EK, Shi T, Campbell H, Chaves SS, Commaille-Chapus C, et al. National burden estimates of hospitalisations for acute lower respiratory infections due to respiratory syncytial virus in young children in 2019 among 58 countries: a modelling study. Lancet Respir Med. 2021;9(2):175–85.

1073. National consensus on geriatric immunization 2011. Acta Med Indones. 2012;44(1):78–91.

1074. Lindblade KA, Praphasiri P, Ditsungnoen D, Chittaganpitch M, Sirilak S, Jaichuang S, et al. Neglected tropical populations: The burden of influenza in the elderly, Thailand. Am J Trop Med Hyg. 2016;95(5):393–4.

1075. Steinhoff MC, Omer SB, Roy E, Arifeen SE, Raqib R, Dodd C, et al. Neonatal outcomes after influenza immunization during pregnancy: A randomized controlled trial. CMAJ. 2012;184(6):645–53.

1076. Chan J, Holmes A, Rabadan R. Network analysis of global influenza spread. PLoS Comput Biol. 2010;6(11):e1001005.

1077. Gopinath SCB, Awazu K, Fujimaki M, Shimizu K. Neu5Acα2,6Gal and Neu5Acα2,3Gal receptor specificities on influenza viruses determined by a waveguide-mode sensor. Acta Biomater. 2013;9(2):5080–7.

1078. Hibino A, Massaad E, Kondo H, Saito R, Odagiri T, Takemae N, et al. Neuraminidase inhibitor susceptibility and evolutionary analysis of human influenza B isolates from three Asian countries during 2012–2015. Infection, Genetics and Evolution. 2018;62:27–33.

1079. Tewawong N, Poovorawan Y, Vongpunsawad S, Marathe BM, Webby RJ, Govorkova EA. Neuraminidase inhibitor susceptibility and neuraminidase enzyme kinetics of human influenza A and B viruses circulating in Thailand in 2010-2015. PLoS ONE. 2018;13(1):e0190877.

1080. Sood R, Kumar N, Bhatia S, Chanu KV, Gupta CL, Pateriya AK, et al. Neuraminidase inhibitors susceptibility profiles of highly pathogenic influenza A (H5N1) viruses isolated from avian species in India (2006–2015). Antiviral Research. 2018;158:143–6.

1081. Potdar V, Dakhave M, Chadha M, Mishra A. Neuraminidase sub typing and drug resistance among influenza A viruses circulating in western India. Int J Infect Dis. 2010;14:e86.

1082. Takia L, Saini L, Keshavan S, Angurana SK, Nallasamy K, Suthar R, et al. Neurological Manifestations of Influenza A (H1N1): Clinical Features, Intensive Care Needs, and Outcome. Indian Journal of Pediatrics. 2020;87(10):803–9.

1083. Boonsathorn N, Kanai Y, Punjumpa J, Bai G, Chittaganpitch M, Petphuwadee U, et al. Neutralization titers against influenza A (H3N2) and influenza B viruses among a non-vaccinated population from Thailand. Southeast Asian Journal of Tropical Medicine and Public Health. 2012;43(3):674–9.

1084. Oh S, Selleck P, Temperton NJ, Chan PKS, Capecchi B, Manavis J, et al. Neutralizing monoclonal antibodies to different clades of Influenza A H5N1 viruses. J Virol Methods. 2009;157(2):161–7.

1085. Jadhao SJ, Suarez DL. New approach to delist highly pathogenic avian influenza viruses from BSL3+ Select Agents to BSL2 non-select status for diagnostics and vaccines. Avian Dis. 2010;54(1):302–6.

1086. Erdem H, Ünal S. New global viral threats. Saudi Med J. 2015;36(4):393–8.

1087. Islam MR, Haque ME, Giasuddin M, Chowdhury EH, Samad MA, Parvin R, et al. New introduction of clade 2.3.2.1 avian influenza virus (H5N1) into bangladesh. Transboundary and Emerging Diseases. 2012;59(5):460–3.

1088. Hoelscher MA, Jayashankar L, Garg S, Veguilla V, Lu X, Singh N, et al. New pre-pandemic influenza vaccines: an egg- and adjuvant-independent human adenoviral vector strategy induces long-lasting protective immune responses in mice. Clin Pharmacol Ther. 2007;82(6):665–71.

1089. Mukherjee S, Majumdar S, Vipat VC, Mishra AC, Chakrabarti AK. Non structural protein of avian influenza A (H11N1) virus is a weaker suppressor of immune responses but capable of inducing apoptosis in host cells. Virology Journal [Internet]. 2012;9. Available from: https://www.scopus.com/inward/record.uri?eid=2-s2.0-84864528172&doi=10.1186%2f1743-422X-9-149&partnerID=40&md5=e815608b5039eaf69bbf57e91cc76dd7

1090. Watanabe T, Watanabe S, Kim JH, Hatta M, Kawaoka Y. Novel approach to the development of effective H5N1 influenza A virus vaccines: use of M2 cytoplasmic tail mutants. J Virol. 2008;82(5):2486–92.

1091. Gilbert CR, Vipul K, Baram M. Novel H1N1 influenza A viral infection complicated by alveolar hemorrhage. Respir Care. 2010;55(5):623–5.

1092. Rashid M, Ara R, Akhter N. Novel influenza H1N1 in pregnancy: Report of a diagnosed case in Bangladesh. Bangladesh Journal of Obstetrics and Gynecology. 2009;24(2):75–8.

1093. Li ZN, Trost JF, Weber KM, LeMasters EH, Nasreen S, Esfandiari J, et al. Novel multiplex assay platforms to detect influenza A hemagglutinin subtype-specific antibody responses for high-throughput and in-field applications. Influenza and other Respiratory Viruses. 2017;11(3):289–97.

1094. Nagarajan S, Kumar M, Murugkar HV, Tripathi S, Shukla S, Agarwal S, et al. Novel reassortant highly pathogenic avian influenza (H5N8) virus in zoos, India. Emerg Infect Dis. 2017;23(4):717–9.

1095. Khadka M. Novel swine influenza A/H1N1 and the phase six pandemic. Journal of the Nepal Medical Association. 2010;49(3):255–8.

1096. Ramadhani BP, Soeroto AY, Suryadinata H, Rakhmilla LE. Nursing knowledge, attitude, and practice to influenza vaccination at suburban hospital in West Java, Indonesia. Journal of Preventive Medicine and Hygiene. 2020;61(1):E15–20.

1097. Katz J, Englund JA, Steinhoff MC, Khatry SK, Shrestha L, Kuypers J, et al. Nutritional status of infants at six months of age following maternal influenza immunization: A randomized placebo-controlled trial in rural Nepal. Vaccine. 2017;35(48):6743–50.

1098. Phyo AP, Swe MMM, Soe K, Warrell CE, Lin HN, Ko CK, et al. Observational study of adult respiratory infections in primary care clinics in Myanmar: understanding the burden of melioidosis, tuberculosis and other infections not covered by empirical treatment regimes. Trans R Soc Trop Med Hyg. 2021;

1099. Naysmith S. Observations from a live bird market in Indonesia following a contained outbreak of avian influenza A (H5N1). Ecohealth. 2014;11(1):50–2.

1100. Nakharuthai C, Boonsoongnern A, Poolperm P, Wajjwalku W, Urairong K, Chumsing W, et al. Occurrence of swine influenza virus infection in swine with porcine respiratory disease complex. Southeast Asian Journal of Tropical Medicine and Public Health. 2008;39(6):1045–53.

1101. Aruksakunwong O, Malaisree M, Decha P, Sompornpisut P, Parasuk V, Pianwanit S, et al. On the lower susceptibility of oseltamivir to influenza neuraminidase subtype N1 than those in N2 and N9. Biophys J. 2007;92(3):798–807.

1102. Acharya KP, Karki S, Shrestha K, Kaphle K. One health approach in Nepal: Scope, opportunities and challenges. One Health [Internet]. 2019;8. Available from: https://www.scopus.com/inward/record.uri?eid=2-s2.0-85070667297&doi=10.1016%2fj.onehlt.2019.100101&partnerID=40&md5=feedd873b0c6a8071d4c88b4f43f7bda

1103. Gongal G. One health approach in the south east asia region: Opportunities and challenges. Curr Top Microbiol Immunol. 2013;366:113–22.

1104. Sukumaran A, Pradeepkumar AS. One Health approach: A platform for intervention in emerging public health challenges of Kerala state. International Journal of One Health. 2015;1:14–25.

1105. Stein ML, Kretzschmar MEE, Van Steenbergen JE, Chanyasanha C, Tipayamongkholgul M, Buskens V, et al. Online respondent-driven sampling for studying contact patterns relevant for the spread of close-contact pathogens: A pilot study in Thailand. PLoS ONE. 2014;9(1):e85256.

1106. Matrajt L, Halloran ME, Longini IMJ. Optimal vaccine allocation for the early mitigation of pandemic influenza. PLoS Comput Biol. 2013;9(3):e1002964.

1107. Roger F, Goutard FL, Paul M, Chanachai K, Thanapongtharm W, Tavornpanich S, et al. Optimizing early detection of avian influenza H5N1 in backyard and free-range poultry production systems in Thailand. Prev Vet Med. 2012;105(3):223–34.

1108. Anekthananon T, Pukritayakamee S, Ratanasuwan W, Jittamala P, Werarak P, Charunwatthana P, et al. Oseltamivir and inhaled zanamivir as influenza prophylaxis in Thai health workers: A randomized, double-blind, placebo-controlled safety trial over 16 weeks. Journal of Antimicrobial Chemotherapy. 2013;68(3):697–707.

1109. Potdar VA, Padbidri VV, Chadha MS. Oseltamivir-resistant influenza A(H1N1) pdm09 virus: first reported case from India. WHO South East Asia J Public Health. 2013;2(3):181–3.

1110. Tandel K, Sharma S, Dash PK, Parida M. Oseltamivir-resistant influenza A(H1N1)pdm09 virus associated with high case fatality, India 2015. Journal of Medical Virology. 2018;90(5):836–43.

1111. Hurt AC, Deng YM, Ernest J, Caldwell N, Leang L, Iannello P, et al. Oseltamivir-resistant influenza viruses circulating during the first year of the influenza A(H1N1) 2009 pandemic in the Asia-Pacific region, March 2009 to March 2010. Euro Surveill. 2011;16(3).

1112. Beigel JH, Bao Y, Beeler J, Manosuthi W, Slandzicki A, Dar SM, et al. Oseltamivir, amantadine, and ribavirin combination antiviral therapy versus oseltamivir monotherapy for the treatment of influenza: a multicentre, double-blind, randomised phase 2 trial. The Lancet Infectious Diseases. 2017;17(12):1255–65.

1113. Shakya G, Marasini B, Karki KB, Upadhaya BP, Acharya J, Adhikari S, et al. Outbreak Investigation Following the 2015 Earthquake Disaster in Nepal. Journal of Nepal Health Research Council. 2018;16(1):61–5.

1114. Upadhyay SK, Singh Pradhan PM, Mahato RK, Marasini B, Upadhyaya B, Shakya G, et al. Outbreak Investigation of Influenza in Pajaru VDC of Jajarkot District of Nepal. Journal of Nepal Health Research Council. 2016;14(34):186–91.

1115. Pokharel S, Karki M, Acharya B, Marasini B, Arjyal A. Outbreak of acute undifferentiated febrile illness in Kathmandu, Nepal: clinical and epidemiological investigation. BMC Infectious Diseases [Internet]. 2020;20(1). Available from: https://www.scopus.com/inward/record.uri?eid=2-s2.0-85078711228&doi=10.1186%2fs12879-020-4803-8&partnerID=40&md5=0a07be9f3e81b51d9b4ec17ebd2aa519

1116. Kushwaha AS, Teli P, Mahen A. Outbreak of influenza (H1N1) amongst children in a residential school. Medical Journal Armed Forces India. 2014;70(3):274–6.

1117. Biswas D, Buragohain M, Yadav K, Dutta M, Sarmah K, Baruah PJ, et al. Outbreak of influenza-like illness investigated during August 2013 near Indo-China border of Arunachal Pradesh, Northeast India. Journal of Medical Virology. 2016;88(11):1999–2003.

1118. Adhikari BR, Shakya G, Upadhyay BP, Prakash Kc K, Shrestha SD, Dhungana GR. Outbreak of pandemic influenza A/H1N1 2009 in Nepal. Virology Journal [Internet]. 2011;8. Available from: https://www.scopus.com/inward/record.uri?eid=2-s2.0-79953069552&doi=10.1186%2f1743-422X-8-133&partnerID=40&md5=d9723c222b8d3db7a32aa8a10193dafe

1119. Vanderburg S, Wijayaratne G, Danthanarayana N, Jayamaha J, Piyasiri B, Halloluwa C, et al. Outbreak of severe acute respiratory infection in Southern Province, Sri Lanka in 2018: A cross-sectional study. BMJ Open [Internet]. 2020;10(11). Available from: https://www.scopus.com/inward/record.uri?eid=2-s2.0-85095801313&doi=10.1136%2fbmjopen-2020-040612&partnerID=40&md5=79396c210dff3a5df1dd8a51c4a70e35

1120. Outbreaks of avian influenza A (H5N1) in Asia and interim recommendations for evaluation and reporting of suspected cases--United States, 2004. MMWR Morb Mortal Wkly Rep. 2004;53(5):97–100.

1121. Walker P, Cauchemez S, Ghani AC, Hartemink N, Tiensin T. Outbreaks of H5N1 in poultry in Thailand: The relative role of poultry production types in sustaining transmission and the impact of active surveillance in control. J R Soc Interface. 2012;9(73):1836–45.

1122. Apisarnthanarak A, Puthavathana P, Kitphati R, Auewarakul P, Mundy LM. Outbreaks of influenza A among nonvaccinated healthcare workers: Implications for resource-limited settings. Infection Control and Hospital Epidemiology. 2008;29(8):777–80.

1123. Varghese YE, Anandabhavan Sukumara Menon AK, Kalaiselvan MS, Arun Kumar AS. Outcome of H1N1 pneumonia in a tertiary care ICU from South India. Intensive Care Med. 2014;40(1):S163–4.

1124. Todkar SS, Gaikwad LA. Outcome of suspected H1N1 influenza cases admitted in tertiary care Govt. Hospital Solapur, Maharashtra. Indian Journal of Medical Sciences. 2017;69(1):6–7.

1125. Khare S, Agarwal R, Singh R, Lal S. Overview of avian influenza. J Indian Med Assoc. 2006;104(7):379–80, 382, 384 passim.

1126. Jha BK, Pandit R, Jha R, Manandhar KD. Overview of seasonal influenza and recommended vaccine during the 2016/2017 season in Nepal. Heliyon. 2020;6(1):e03304.

1127. Kandel N, Shrestha JM, Upadhyay B, Shrestha AK, Shakya G. Pandemic (H1N1) 2009 cases in Nepal. Journal of the Nepal Medical Association. 2012;52(4):201–4.

1128. Mukherjee S, Vipat VC, Mishra AC, Pawar SD, Chakrabarti AK. Pandemic (H1N1) 2009 influenza virus induces weaker host immune responses in vitro: A possible mechanism of high transmissibility. Virology Journal [Internet]. 2011;8. Available from: https://www.scopus.com/inward/record.uri?eid=2-s2.0-79953125413&doi=10.1186%2f1743-422X-8-140&partnerID=40&md5=3543f73067c65dad90db0f3ddab60497

1129. Sahoo JN, Poddar B, Azim A, Singh RK, Gurjar M, Baronia AK. Pandemic (H1N1) 2009 influenza: Experience from a critical care unit in India. Indian Journal of Critical Care Medicine. 2010;14(3):156–9.

1130. Pramanick A, Rathore S, Peter JV, Moorthy M, Lionel J. Pandemic (H1N1) 2009 virus infection during pregnancy in South India. International Journal of Gynecology and Obstetrics. 2011;113(1):32–5.

1131. Sreta D, Tantawet S, Na Ayudhya SN, Thontiravong A, Wongphatcharachai M, Lapkuntod J, et al. Pandemic (H1N1) 2009 virus on commercial swine farm, Thailand. Emerging Infectious Diseases. 2010;16(10):1587–90.

1132. Narain JP, Kumar R, Bhatia R. Pandemic (H1N1) 2009: Epidemiological, clinical and prevention aspects. National Medical Journal of India. 2009;22(5):242–7.

1133. Koul PA, Mir MA, Bali NK, Chawla-Sarkar M, Sarkar M, Kaushik S, et al. Pandemic and seasonal influenza viruses among patients with acute respiratory illness in Kashmir (India). Influenza and other Respiratory Viruses. 2011;5(6):e521–7.

1134. Kant L, Guleria R. Pandemic flu, 1918: After hundred years, India is as vulnerable. Indian J Med Res. 2018;147:221–4.

1135. Gunasekaran P, Mohana S, Kaveri K, Arunagiri K, Kiruba R, Suresh Babu BV, et al. Pandemic H1N1 2009 among pediatric age group in Tamilnadu, June 2009 - Aug 2010. Indian Journal of Public Health Research and Development. 2014;5(3):226–30.

1136. Mishra AC, Chadha MS, Choudhary ML, Potdar VA. Pandemic Influenza (H1N1) 2009 is associated with severe disease in India. PLoS ONE. 2010;5(5):e10540.

1137. Gandhoke I, Rawat D, Rai A, Khare S, Ichhpujani R. Pandemic Influenza A (H1N1) 2009 in India: Duration of virus shedding in patients under antiviral treatment. Indian Journal of Medical Microbiology. 2011;29(1):37–41.

1138. Mahato RK, Bhandari GP, Shrestha JM, Basnet P. Pandemic influenza A (H1N1) 2009 outbreak investigation in Nepal. Journal of Nepal Health Research Council. 2010;8(2):75–7.

1139. Goel M, Goel M, Khanna P, Mittal K. Pandemic influenza A (H1N1) 2009 vaccine: An update. Indian Journal of Medical Microbiology. 2011;29(1):13–8.

1140. Khuntirat B, Yoon IK, Gibbons RV, Krueger W, Heil GL, Friary JA, et al. Pandemic influenza A (H1N1) virus infections among villagers living in rural Thailand. Int J Infect Dis. 2012;16:e348.

1141. Gurav YK, Pawar SD, Chadha MS, Potdar VA, Koratkar SS, Mishra AC, et al. Pandemic influenza A(H1N1) 2009 outbreak in a residential school at Panchgani, Maharashtra, India. Indian J Med Res. 2010;132(7):67–71.

1142. Adisasmito W, Hunter BM, Krumkamp R, Latief K, Rudge JW, Hanvoravongchai P, et al. Pandemic influenza and health system resource gaps in bali: An analysis through a resource transmission dynamics model. Asia-Pacific Journal of Public Health. 2015;27(2):NP713–33.

1143. Meeyai A, Cooper B, Coker R, Pan-Ngum W, Akarasewi P, Iamsirithaworn S. Pandemic influenza H1N1 2009 in Thailand. WHO South East Asia J Public Health. 2012;1(1):59–68.

1144. Hanvoravongchai P, Adisasmito W, Chau PN, Conseil A, De Sa J, Krumkamp R, et al. Pandemic influenza preparedness and health systems challenges in Asia: Results from rapid analyses in 6 Asian countries. BMC Public Health [Internet]. 2010;10. Available from: https://www.scopus.com/inward/record.uri?eid=2-s2.0-77953125535&doi=10.1186%2f1471-2458-10-322&partnerID=40&md5=b1599112624dda8e98f7d1001509e268

1145. Coker R, Mounier-Jack S. Pandemic influenza preparedness in the Asia-Pacific region. Lancet. 2006;368(9538):886–9.

1146. Wijesinghe PR, Ofrin RH, Bhola AK, Inbanathan FY, Bezbaruah S. Pandemic influenza preparedness in the WHO South-East Asia Region: a model for planning regional preparedness for other priority high-threat pathogens. WHO South-East Asia journal of public health. 2020;9(1):43–9.

1147. Cinti S. Pandemic influenza: are we ready? Disaster Manag Response. 2005;3(3):61–7.

1148. Mahesh SH, Kushwaha AS, Kotwal A. Pandemic influenza: Experience in a flu OPD of a tertiary care hospital. Medical Journal Armed Forces India. 2014;70(1):39–42.

1149. Chaturvedi S. Pandemic influenza: Imminent threat, preparedness and the divided globe. Indian Pediatrics. 2009;46(2):115–21.

1150. Chakraborti C. Pandemic management and developing world bioethics: Bird flu in west Bengal. Developing World Bioethics. 2009;9(3):161–6.

1151. Hickey J, Gagnon AJ, Jitthai N. Pandemic preparedness: Perceptions of vulnerable migrants in Thailand towards WHO-recommended non-pharmaceutical interventions: A cross-sectional study. BMC Public Health [Internet]. 2014;14(1). Available from: https://www.scopus.com/inward/record.uri?eid=2-s2.0-84903244133&doi=10.1186%2f1471-2458-14-665&partnerID=40&md5=b4aa4e5d7a82259ba622be37e6b50a94

1152. Ramprasad C, Zachariah R, Steinhoff M, Simon A. Parental attitudes towards influenza vaccination for children in South India. World Journal of Pediatrics. 2017;13(1):84–90.

1153. Dash SK, Kumar M, Kataria JM, Nagarajan S, Tosh C, Murugkar HV, et al. Partial heterologous protection by low pathogenic H9N2 virus against natural H9N2-PB1 gene reassortant highly pathogenic H5N1 virus in chickens. Microbial Pathogenesis. 2016;95:157–65.

1154. Azhar M, Lubis AS, Siregar ES, McGrane J, Morgan I, Alders RG, et al. Participatory disease surveillance and response in Indonesia: Strengthening veterinary services and empowering communities to prevent and control highly pathogenic avian influenza. Avian Dis. 2010;54:749–53.

1155. Bhatnagar N, Gangadharan N, Garg S. Participatory disease surveillance in India: A critical interpretative synthesis! Int J Infect Dis. 2020;101:376.

1156. Pantin-Jackwood MJ, Swayne DE. Pathobiology of Asian highly pathogenic avian influenza H5N1 virus infections in ducks. Avian Dis. 2007;51(1):250–9.

1157. Sreta D, Kedkovid R, Tuamsang S, Kitikoon P, Thanawongnuwech R. Pathogenesis of swine influenza virus (Thai isolates) in weanling pigs: An experimental trial. Virology Journal [Internet]. 2009;6. Available from: https://www.scopus.com/inward/record.uri?eid=2-s2.0-65549126837&doi=10.1186%2f1743-422X-6-34&partnerID=40&md5=adc92900d6c560e51f918614a63d8266

1158. Shankar BP, Gowda RNS, Manjunath Prabhu BH, Pattnaik B, Nagarajan S, Patil SS, et al. Pathogenicity for Chickens of Avian Influenza Virus Strain H9N1 Isolated from Water Coot in India. International Journal of Poultry Science. 2009;8(3):252–5.

1159. Suzuki H, Okamatsu M, Yamaguchi S, Saito T, Watanabe C, Takemae N, et al. Pathogenicity of highly pathogenic avian influenza viruses of H5N1 subtype isolated in Thailand for different poultry species. Vet Microbiol. 2009;133(1):65–74.

1160. Shelke VN, Kolhapure RM, Kadam D, Sangle S, Chadha MC, Basu A, et al. Pathologic study of pandemic influenza A (H1N1) 2009 cases from India. Pathology International. 2012;62(1):36–42.

1161. Bal A, Suri V, Mishra B, Bhalla A, Agarwal R, Abrol A, et al. Pathology and virology findings in cases of fatal influenza A H1N1 virus infection in 2009-2010. Histopathology. 2012;60(2):326–35.

1162. Suba S, Nagarajan S, Saxena VK, Kumar M, Vanamayya PR, Rajukumar K, et al. Pathology of a H5N1, highly pathogenic avian influenza virus, in two Indian native chicken breeds and a synthetic broiler line. Indian J Exp Biol. 2015;53(4):202–7.

1163. Mumu TT, Nooruzzaman M, Hasnat A, Parvin R, Chowdhury EH, Bari ASM, et al. Pathology of an outbreak of highly pathogenic avian influenza A(H5N1) virus of clade 2.3.2.1a in turkeys in Bangladesh. Journal of Veterinary Diagnostic Investigation. 2021;33(1):124–8.

1164. Nooruzzaman M, Haque ME, Chowdhury EH, Islam MR. Pathology of clade 2.3.2.1 avian influenza virus (H5N1) infection in quails and ducks in Bangladesh. Avian Pathology. 2019;48(1):73–9.

1165. Gupta SD, Hoque MA, Fournié G, Henning J. Patterns of Avian Influenza A (H5) and A (H9) virus infection in backyard, commercial broiler and layer chicken farms in Bangladesh. Transboundary and Emerging Diseases. 2021;68(1):137–51.

1166. Prateepko T, Chongsuvivatwong V. Patterns of perception toward influenza pandemic among the front-line responsible health personnel in southern Thailand: A Q methodology approach. BMC Public Health [Internet]. 2009;9. Available from: https://www.scopus.com/inward/record.uri?eid=2-s2.0-67651153043&doi=10.1186%2f1471-2458-9-161&partnerID=40&md5=f0d0f2fc3875d94211ccc2aafbdd24ef

1167. Parakh A, Kumar A, Kumar V, Kumar Dutta A, Khare S. Pediatric hospitalizations associated with 2009 pandemic influenza a (H1N1): An experience from a tertiary care center in North India. Indian Journal of Pediatrics. 2010;77(9):981–5.

1168. Gargano LM, Thacker N, Choudhury P, Weiss PS, Russ RM, Pazol K, et al. Pediatricians’ perceptions of vaccine effectiveness and safety are significant predictors of vaccine administration in India. International Health. 2013;5(3):205–10.

1169. Malik A. Pentavalent Vaccine and Adverse Events Following Immunization—Untangling the Misinterpretations. Indian Journal of Pediatrics. 2014;81(12):1353–7.

1170. Bairwa M, Pilania M, Rajput M, Khanna P, Kumar N, Nagar M, et al. Pentavalent vaccine: a major breakthrough in India’s Universal Immunization Program. Hum Vaccin Immunother. 2012;8(9):1314–6.

1171. Mohanraj U, Chander S, Chavan YG. Peptide based viral detection systems for effective diagnosis of common viral infections in India. Curr Protein Pept Sci [Internet]. 2017;18(2). Available from: http://benthamscience.com/contents-JCode-CPPS-Vol-00000013-Iss-00000001.htm

1172. Hirve S, Chadha M, Lele P, Lafond KE, Deoshatwar A, Sambhudas S, et al. Performance of case definitions used for influenza surveillance among hospitalized patients in a rural area of India. Bulletin of the World Health Organization. 2012;90(11):804–12.

1173. Koul PA, Mir H, Bhat MA, Khan UH, Khan MM, Chadha MS, et al. Performance of rapid influenza diagnostic tests (QuickVue) for Influenza A and B Infection in India. Indian Journal of Medical Microbiology. 2015;33:S26–31.

1174. Wesley MG, Tinoco Y, Patel A, Suntarratiwong P, Hunt D, Sinthuwattanawibool C, et al. Performance of symptom-based case definitions to identify influenza virus infection among pregnant women in middle-income countries: findings from the Pregnancy and Influenza Multinational Epidemiologic (PRIME) Study. Clin Infect Dis. 2020;

1175. Srikanth P, Mani M, Barani R, Sarangan G, Reju S, Annamalai R, et al. Periodicity in the waxing and waning of Influenza A H1N1: A report from a tertiary care center in Chennai India. Int J Infect Dis. 2016;45:192.

1176. Hogerwerf L, Wallace RG, Ottaviani D, Slingenbergh J, Prosser D, Bergmann L, et al. Persistence of highly pathogenic avian influenza H5N1 virus defined by agro-ecological niche. Ecohealth. 2010;7(2):213–25.

1177. Nasamran C, Janetanakit T, Chiyawong S, Boonyapisitsopa S, Bunpapong N, Amonsin A, et al. Persistence of pdm2009-H1N1 internal genes of swine influenza in pigs, Thailand. Sci Rep. 2020;10(1):19847.

1178. Odo NU, Raynor PC, Beaudoin A, Somrongthong R, Scheftel JM, Donahue JG, et al. Personal protective equipment use and handwashing among animal farmers: A multi-site assessment. Journal of Occupational and Environmental Hygiene. 2015;12(6):363–8.

1179. Jittamala P, Pukrittayakamee S, Tarning J, Lindegardh N, Hanpithakpong W, Taylor WRJ, et al. Pharmacokinetics of orally administered oseltamivir in healthy obese and nonobese thai subjects. Antimicrobial Agents and Chemotherapy. 2014;58(3):1615–21.

1180. Hon CC, Shi M, Tun HM, Li J, Jiang J, Leung FCC, et al. Phylodynamics of H5N1 avian influenza virus in Indonesia. Mol Ecol. 2012;21(12):3062–77.

1181. Adam DC, Scotch M, Macintyre CRaina. Phylodynamics of influenza A/H1N1pdm09 in India reveals circulation patterns and increased selection for clade 6b residues and other high mortality mutants. Viruses. 2019;11(9):791.

1182. Flavia GBA, Natarajaseenivasan K. Phylogenetic analysis of H1N1 sequences from pandemic infections during 2009 in India. Bioinformation. 2011;5(10):416–21.

1183. Prakash N, Devangi P, Madhuuri K, Khushbu P, Deepali P. Phylogenetic analysis of H1N1 swine flu virus isolated in India. Journal of Antivirals and Antiretrovirals. 2011;3(1):011–3.

1184. Yan HX, Xu HF, He WJ, Xie Y, Dong GY. Phylogenetic analysis of HA and NA genes of influenza H1N1 viruses from 1918 to 2017. Acta Virol. 2019;63(2):195–202.

1185. Mutisari D, Muflihanah M, Wibawa H, Hendrawati F, Putra HH, Sulistyo KP, et al. Phylogenetic Analysis of HPAI H5N1 Virus from Duck Swab Specimens in Indonesia. Journal of Advanced Veterinary and Animal Research. 2021;8(2):346–54.

1186. Sharma V, Sharma M, Dhull D, Kaushik S, Kaushik S. Phylogenetic analysis of the hemagglutinin gene of influenza A(H1N1)pdm09 and A(H3N2) virus isolates from Haryana, India. VirusDisease. 2019;30(3):336–43.

1187. Danishuddin M, Khan AU. Phylogenetic Analysis of the Neuraminidase Gene Reveals that the H5N1 Strains Prevalent in Chickens During 2006 Bird Flu Outbreaks in Two Regions of Maharashtra, India Are Genetically Different. Genomics Proteomics Bioinform. 2009;7(1):57–61.

1188. Babakir-Mina M, Ciccozzi M, Ciotti M, Marcuccilli F, Balestra E, Dimonte S, et al. Phylogenetic analysis of the surface proteins of influenza A (H5N1) viruses isolated in Asian and African populations. New Microbiol. 2009;32(4):397–403.

1189. Adisasmito W, Budayanti SN, Aisyah DN, Gallo Cassarino T, Rudge JW, Watson SJ, et al. Phylogenetic characterisation of circulating, clinical influenza isolates from Bali, Indonesia: Preliminary report from the BaliMEI project. BMC Infectious Diseases [Internet]. 2017;17(1). Available from: https://www.scopus.com/inward/record.uri?eid=2-s2.0-85027970891&doi=10.1186%2fs12879-017-2684-2&partnerID=40&md5=844899934b05ccec8d6b6478c04bc05f

1190. Takano R, Nidom CA, Kiso M, Muramoto Y, Yamada S, Sakai-Tagawa Y, et al. Phylogenetic characterization of H5N1 avian influenza viruses isolated in Indonesia from 2003-2007. Virology. 2009;390(1):13–21.

1191. Kuypers J, Perchetti GA, Chu HY, Newman KL, Katz J, Khatry SK, et al. Phylogenetic characterization of rhinoviruses from infants in Sarlahi, Nepal. Journal of Medical Virology. 2019;91(12):2108–16.

1192. Tosh C, Nagarajan S, Murugkar HV, Jain R, Behera P, Katare M, et al. Phylogenetic evidence of multiple introduction of H5N1 virus in Malda district of West Bengal, India in 2008. Veterinary Microbiology. 2011;148(2):132–9.

1193. Zar Htwe KT, Shobugawa Y, Odagiri T, Hibino A, Kondo H, Yagami R, et al. Phylogeographic analysis of human influenza A and B viruses in Myanmar, 2010-2015. PLoS ONE. 2019;14(1):e0210550.

1194. Njoto EN, Bui CM, Adam DC, Chughtai AA, Scotch M, MacIntyre CR. Phylogeography of H5N1 avian influenza virus in Indonesia. Transboundary Emer Dis. 2018;65(5):1339–47.

1195. Mangiri A, Iuliano AD, Wahyuningrum Y, Praptiningsih CY, Lafond KE, Storms AD, et al. Physician’s knowledge, attitudes, and practices regarding seasonal influenza, pandemic influenza, and highly pathogenic avian influenza A (H5N1) virus infections of humans in Indonesia. Influenza and other Respiratory Viruses. 2017;11(1):93–9.

1196. Rimi NA, Fahad MdH, Mortaza SM, Mahmud AA, Islam A, Hassan Z, et al. Piloting workstations to improve hygiene practices among poultry workers during poultry processing in a live bird market in Bangladesh. Am J Trop Med Hyg. 2017;97(5):573.

1197. Devnani M, Gupta AK, Devnani B. Planning and response to the influenza A (H1N1) pandemic: ethics, equity and justice. Indian journal of medical ethics. 2011;8(4):237–40.

1198. Kost GJ, Hale KN, Brock TK, Louie RF, Gentile NL, Kitano TK, et al. Point-of-care testing for disasters: needs assessment, strategic planning, and future design. Clin Lab Med. 2009;29(3):583–605.

1199. Ichinohe T, Ainai A, Tashiro M, Sata T, Hasegawa H. PolyI:polyC12U adjuvant-combined intranasal vaccine protects mice against highly pathogenic H5N1 influenza virus variants. Vaccine. 2009;27(45):6276–9.

1200. Dutta M, Dutta P, Medhi S, Borkakoty B, Biswas D. Polymorphism of HLA class I and class II alleles in influenza A(H1N1)pdm09 virus infected population of Assam, Northeast India. Journal of Medical Virology. 2018;90(5):854–60.

1201. Pagala MA, Saili T, Nafiu LO, Sandiah N, Baa LO, Aku AS, et al. Polymorphism of Mx|Hpy81 genes in native chickens observed using the PCR-RFLP technique. International Journal of Poultry Science. 2017;16(9):364–8.

1202. Koul PA, Bali NK, Ali S, Ahmad SJ, Bhat MA, Mir H, et al. Poor uptake of influenza vaccination in pregnancy in northern India. International Journal of Gynecology and Obstetrics. 2014;127(3):234–7.

1203. Farzin A, Saha SK, Baqui AH, Choi Y, Ahmed NU, Simoes EAF, et al. Population-based incidence and etiology of community-acquired neonatal viral infections in Bangladesh: A community-based and hospital-based surveillance study. Pediatric Infectious Disease Journal. 2015;34(7):706–11.

1204. Havers FP, Fry AM, Goswami D, Nahar K, Sharmin AT, Rahman M, et al. Population-based Incidence of Childhood Pneumonia Associated with Viral Infections in Bangladesh. Pediatric Infectious Disease Journal. 2019;38(4):344–50.

1205. Nasreen S, Brooks WA, Homaira N, Mamun AA, Bhuiyan MU, Rahman M, et al. Population-based incidence of severe acute respiratory virus infections among children aged <5 years in rural Bangladesh, June-October 2010. PLoS ONE. 2014;9(2):e89978.

1206. Hughes MM, Englund JA, Kuypers J, Tielsch JM, Khatry SK, Shrestha L, et al. Population-based pertussis incidence and risk factors in infants less than 6 months in Nepal. Journal of the Pediatric Infectious Diseases Society. 2017;6(1):33–9.

1207. Khan MSI, Akbar SMF, Hossain ST, Mahatab M, Hossain MM, Idrus Z. Possible route of transmission of highly pathogenic avian influenza virus type H5N1 in family poultry at rural Bangladesh. Pakistan Veterinary Journal. 2012;32(1):112–6.

1208. Ganju SA, Singh D, Mehta V, Bhagra S, Sharma P, Kanga AK. Post pandemic scenario of pandemic 2009 H1N1 and seasonal influenza a virus infection in Himachal Pradesh, India. Journal of Communicable Diseases. 2015;47(1):8–13.

1209. Ali Z, Hasan M, Giasuddin. Potential risk factors of avian influenza virus infection in asymptomatic commercialchicken flocks in selected areas of Bangladesh during 2019. Journal of Advanced Veterinary and Animal Research. 2021;8(1):51–7.

1210. Souris M, Selenic D, Khaklang S, Ninphanomchai S, Minet G, Gonzalez JP, et al. Poultry farm vulnerability and risk of avian influenza re-emergence in Thailand. International Journal of Environmental Research and Public Health. 2014;11(1):934–51.

1211. Delabouglise A, Nguyen-Van-Yen B, Thanh NTL, Xuyen HTA, Tuyet PN, Lam HM, et al. Poultry population dynamics and mortality risks in smallholder farms of the Mekong river delta region. BMC Vet Res. 2019;15(1):205.

1212. Aengwanich W, Intarakhamhaeng M, Wandee J, Nongbua T, Chaiyasak S, Srikot P, et al. Poultry production clusters (PPCs) after ai outbreaks in Thailand: Past, present and future direction. International Journal of Poultry Science. 2012;11(8):541–50.

1213. Chantong W, Kaneene B. Poultry raising systems and highly pathogenic avian influenza outbreaks in thailand: The situation, associations,and impacts. Southeast Asian Journal of Tropical Medicine and Public Health. 2011;42(3):596–608.

1214. Rimi NA li, Sultana R, Ishtiak-Ahmed K, Khan SU ddin, Sharker MAY, Uz Zaman R, et al. Poultry slaughtering practices in rural communities of Bangladesh and risk of avian influenza transmission: a qualitative study. Ecohealth. 2014;11(1):83–93.

1215. Fournie G, Hoque A, Biswas P, Barnett T, Mangtani P, Giasuddin M, et al. Poultry value chains shaping avian influenza viral transmission in Bangladesh. Trans R Soc Trop Med Hyg. 2019;113:S44–5.

1216. Prapasiri P, Dowell SF, Laosiritaworn Y, Pattanasin S, Olsen SJ. Poultry-handling practices during avian influenza outbreak, Thailand. Emerg Infect Dis. 2005;11(10):1601–3.

1217. Lye DCB, Nguyen DH, Giriputro S, Anekthananon T, Eraksoy H, Tambyah PA. Practical management of avian influenza in humans. Singapore Med J. 2006;47(6):471–5.

1218. Paul M, Baritaux V, Wongnarkpet S, Poolkhet C, Thanapongtharm W, Roger F, et al. Practices associated with Highly Pathogenic Avian Influenza spread in traditional poultry marketing chains: Social and economic perspectives. Acta Tropica. 2013;126(1):43–53.

1219. Reechaipichitkul W. Precipitating causes and outcomes of chronic obstructive pulmonary disease exacerbation at a tertiary care center in northeast Thailand. Asian Biomed. 2014;8(2):229–36.

1220. Landry N, Ward BJ, Trépanier S, Montomoli E, Dargis M, Lapini G, et al. Preclinical and clinical development of plant-made virus-like particle vaccine against avian H5N1 influenza. PLoS One. 2010;5(12):e15559.

1221. Van Boeckel TP, Thanapongtharm W, Robinson T, D’Aietti L, Gilbert M. Predicting the distribution of intensive poultry farming in Thailand. Agric Ecosyst Environ. 2012;149:144–53.

1222. Jongkon N, Mokmak W, Chuakheaw D, Shaw PJ, Tongsima S, Sangma C. Prediction of avian influenza A binding preference to human receptor using conformational analysis of receptor bound to hemagglutinin. BMC Genomics. 2009;10:S24.

1223. Kaoiean S, Kittikraisak W, Suntarattiwong P, Ditsungnoen D, Phadungkiatwatana P, Srisantiroj N, et al. Predictors for influenza vaccination among Thai pregnant woman: The role of physicians in increasing vaccine uptake. Influenza and other Respiratory Viruses. 2019;13(6):582–92.

1224. Murray A, Englund J, Tielsch J, Katz J, Shrestha L, Khatry S, et al. Predictors of measles and rubella serostatus in mother-infant pairs in Rural Nepal. Open Forum Infect Dis. 2017;4:S118.

1225. Chawla R, Kansal S, Chauhan M, Jain A, Jibhkate B. Predictors of mortality and length of stay in hospitalized cases of 2009 influenza A (H1N1): Experiences of a tertiary care center. Indian Journal of Critical Care Medicine. 2013;17(5):275–82.

1226. Kinikar AA, Kulkarni RK, Valvi CT, Mave V, Gupte N, Khadse S, et al. Predictors of mortality in hospitalized children with pandemic H1N1 influenza 2009 in Pune, India. Indian Journal of Pediatrics. 2012;79(4):459–66.

1227. Praphasiri P, Ditsungnoen D, Sirilak S, Rattanayot J, Areerat P, Dawood FS, et al. Predictors of seasonal influenza vaccination among older adults in Thailand. PLoS ONE. 2017;12(11):e0188422.

1228. Carter NJ, Plosker GL. Prepandemic influenza vaccine H5N1 (split virion, inactivated, adjuvanted) [Prepandrix]: a review of its use as an active immunization against influenza A subtype H5N1 virus. BioDrugs. 2008;22(5):279–92.

1229. Goel S, Singh A, Lenka SR, Gupta AK. Preparations and limitations for prevention of severe acute respiratory syndrome in a tertiary care centre of India. J Hosp Infect. 2007;66(2):142–7.

1230. Prateepko T, Chongsuvivatwong V. Preparedness against an influenza pandemic of the frontline health facilities in Southern Thailand: Factor and cluster analyses. Asia-Pacific Journal of Public Health. 2012;24(1):28–38.

1231. Srivanichakorn W, Asavathitanonta K, Washirasaksiri C, Chaisathaphon T, Chouriyagune C, Phisalprapa P, et al. Prescribing rate of influenza vaccine among internal medicine residents for outpatient continuum care. Journal of the Medical Association of Thailand. 2014;97(12):1281–9.

1232. Hassan MM, Islam A, Hasan RB, Rahman MK, Webby RJ, Hoque MA, et al. Prevalence and distribution of avian influenza viruses in domestic ducks at the waterfowl-chicken interface in Wetlands. Pathogens. 2020;9(11):1–18.

1233. Hassan MM, El Zowalaty ME, Islam A, Khan SA, Rahman MK, Järhult JD, et al. Prevalence and diversity of avian influenza virus hemagglutinin sero-subtypes in poultry and wild birds in Bangladesh. Veterinary Sciences [Internet]. 2020;7(2). Available from: https://www.scopus.com/inward/record.uri?eid=2-s2.0-85087442818&doi=10.3390%2fVETSCI7020073&partnerID=40&md5=62a4889fd40823d42427a539786c32ec

1234. Serrao E, Meers J, Pym R, Henning J, Copland R, Eagles D. Prevalence and incidence of Newcastle disease and prevalence of Avian Influenza infection of scavenging village chickens in Timor-Leste. Prev Vet Med. 2012;104(3):301–8.

1235. Nolan T, Borja-Tabora C, Lopez P, Weckx L, Ulloa-Gutierrez R, Lazcano-Ponce E, et al. Prevalence and incidence of respiratory syncytial virus and other respiratory viral infections in children aged 6 months to 10 years with influenza-like illness enrolled in a randomized trial. Clinical Infectious Diseases. 2015;60(11):e80–9.

1236. Gunasekaran P, Mohana S, Kiruba R, Ruban K, Magesh S, Indhumathi CP, et al. Prevalence and molecular characterization of circulating respiratory syncytial virus (RSV) in Chennai, South India during 2011-2014. Biosci Biotechnol Res Asia. 2016;13(2):1055–62.

1237. Horthongkham N, Athipanyasilp N, Sirijatuphat R, Assanasen S, Sutthent R. Prevalence and molecular characterization of human metapneumovirus in influenza a negative sample in Thailand. Journal of Clinical Laboratory Analysis. 2014;28(5):398–404.

1238. Sapkota A, Upadhyay BP. Prevalence and seasonality of influenza virus among pediatric population in Nepal, 2018. Int J Infect Dis. 2020;101:526.

1239. Kim Y, Biswas PK, Giasuddin M, Hasan M, Mahmud R, Chang YM, et al. Prevalence of avian influenza A(H5) and A(H9) viruses in live bird markets, Bangladesh. Emerging Infectious Diseases. 2018;24(12):2309–16.

1240. Rahman MS, Rabbani MG, Uddin MJ, Chakrabartty A, Her M. Prevalence of Avian Influenza and Newcastle Disease Viruses in poultry in selected areas of Bangladesh using rapid antigen detection kit. Archives of Clinical Microbiology [Internet]. 2012;3(1). Available from: https://www.scopus.com/inward/record.uri?eid=2-s2.0-84872035062&doi=10.3823%2f248&partnerID=40&md5=c1c9e500fd8b77d0fc8eb499b6ff41e1

1241. Kumrul Hassan M, Nahat FW, Bhattacharjee PK, Rahman MS, Anisur Rahman AKM, Islam MA, et al. Prevalence of canine influenza infection in pet dogs and canine parvovirus infection in street dogs of bangladesh. Journal of Veterinary Clinics. 2017;34(3):165–71.

1242. Lockhart C, Brum E, Barrios PR, Wuryaninggsih E. Prevalence of HPAI in live-bird markets in the Jabodatabek region of west Java, Indonesia in 2009. Int J Infect Dis. 2010;14:e167.

1243. Nandhini G, Sujatha S, Jain N, Dhodapkar R, Tamilarasu K, Krishnamurthy S, et al. Prevalence of Human metapneumovirus infection among patients with influenza-like illness: Report from a Tertiary Care Centre, Southern India. Indian Journal of Medical Microbiology. 2016;34(1):27–32.

1244. Tamang JM, Shrestha RK, Maharjan U, Upadhyay BP. Prevalence of influenza and viral co-pathogens with severe acute respiratory infection visiting at tertiary care hospital in Nepal. Int J Infect Dis. 2020;101:198.

1245. Roy S, Patil D, Dahake R, Mukherjee S, Athlekar SV, Deshmukh RA, et al. Prevalence of influenza virus among the paediatric population in Mumbai during 2007-2009. Indian Journal of Medical Microbiology. 2012;30(2):155–8.

1246. Kini S, Kalal BS, Chandy S, Shamsundar R, Shet A. Prevalence of respiratory syncytial virus infection among children hospitalized with acute lower respiratory tract infections in Southern India. World J Clin Pediatr. 2019;8(2):33–42.

1247. Posuwan N, Payungporn S, Poovorawan Y, Thontiravong A, Kitikoon P, Amonsin A. Prevalence of respiratory viruses isolated from dogs in Thailand during 2008-2009. Asian Biomed. 2010;4(4):563–9.

1248. Samransamruajkit R, Hiranrat T, Chieochansin T, Sritippayawan S, Decrojanawong J, Prapphal N, et al. Prevalence, clinical presentations and complications among hospitalized children with influenza pneumonia. Japanese Journal of Infectious Diseases. 2008;61(6):446–9.

1249. Kusala MKJ, Ansori ANM, Nidom RV, Indrasari S, Astutik AF, Normalina I, et al. Primary cell culture of zebrafish (Danio rerio) as a material for developing H5N1 avian influenza vaccines. Research Journal of Pharmacy and Technology. 2020;13(12):6140–6.

1250. Agustiningsih A, Trimarsanto H, Setiawaty V, Artika IM, Muljono DH. Primer development to obtain complete coding sequence of HA and NA genes of influenza A/H3N2 virus. BMC Res Notes. 2016;9(1):423.

1251. Samaan G, Indriani R, Carrasco LR, Lokuge K, Cook AR, Kelly PM, et al. Prioritizing live bird markets at risk of avian influenza H5N1 virus contamination for intervention: A simple tool for low resource settings. Preventive Veterinary Medicine. 2012;107(3):280–5.

1252. Campbell Z, Coleman P, Guest A, Kushwaha P, Ramuthivheli T, Osebe T, et al. Prioritizing smallholder animal health needs in East Africa, West Africa, and South Asia using three approaches: Literature review, expert workshops, and practitioner surveys. Prev Vet Med. 2021;189:105279.

1253. Ungchusak K, Auewarakul P, Dowell SF, Kitphati R, Auwanit W, Puthavathana P, et al. Probable person-to-person transmission of avian influenza A (H5N1). N Engl J Med. 2005;352(4):333–40.

1254. Thanawongnuwech R, Amonsin A, Tantilertcharoen R, Damrongwatanapokin S, Theamboonlers A, Payungporn S, et al. Probable tiger-to-tiger transmission of avian influenza H5N1. Emerg Infect Dis. 2005;11(5):699–701.

1255. Suri V, Bhalla A, Sagar V, Abrol A, Lakshmi PM, Singh MP, et al. Profiles of H1N1 positive patients: A study in a tertiary care hospital In north India. Int J Infect Dis. 2014;21:315.

1256. Sharma R, Agarwal S, Mehta S, Nawal CL, Bhandari S, Rathore M, et al. Profiling the mortality due to influenza a (H1N1) pdm09 at a tertiary care hospital in jaipur during the current season - January & February 2015. Journal of Association of Physicians of India. 2015;63:36–9.

1257. Thomas M, Mani RS, Philip M, Adhikary R, Joshi S, Revadi SS, et al. Proinflammatory chemokines are major mediators of exuberant immune response associated with Influenza A (H1N1) pdm09 virus infection. Journal of Medical Virology. 2017;89(8):1373–81.

1258. Chan MCW, Cheung CY, Chui WH, Tsao SW, Nicholls JM, Chan YO, et al. Proinflammatory cytokine responses induced by influenza A (H5N1) viruses in primary human alveolar and bronchial epithelial cells. Respir Res. 2005;6(1):135.

1259. Ramadhany R, Setiawaty V, Wibowo HA, Lokida D. Proportion of influenza cases in severe acute respiratory illness in Indonesia during 2008-2009. Medical Journal of Indonesia. 2010;19(4):264–7.

1260. Matsui S. Protecting human and ecological health under viral threats in Asia. Water Sci Technol. 2005;51(8):91–7.

1261. Kiertiburanakul S, Phongsamart W, Tantawichien T, Manosuthi W, Kulchaitanaroaj P. PRS27 ECONOMIC BURDEN OF INFLUENZA IN THAILAND: A SYSTEMATIC REVIEW. Value Health. 2019;22:S354.

1262. Purohit V, Kudale A, Sundaram N, Joseph S, Schaetti C, Weiss MG. Public health policy and experience of the 2009 H1N1 influenza pandemic in Pune, India. International Journal of Health Policy and Management. 2018;7(2):154–66.

1263. Kamate SK, Agrawal A, Chaudhary H, Singh K, Mishra P, Asawa K. Public knowledge, attitude and behavioural changes in an Indian population during the Influenza A (H1N1) outbreak. Journal of Infection in Developing Countries. 2010;4(1):007–14.

1264. Chantratita W, Sukasem C, Kaewpongsri S, Srichunrusami C, Pairoj W, Thitithanyanont A, et al. Qualitative detection of avian influenza A (H5N1) viruses: a comparative evaluation of four real-time nucleic acid amplification methods. Mol Cell Probes. 2008;22(5):287–93.

1265. Islam SKS, Akwar H, Hossain MM, Sufian MA, Hasan MZ, Chakma S, et al. Qualitative risk assessment of transmission pathways of highly pathogenic avian influenza (HPAI) virus at live poultry markets in Dhaka city, Bangladesh. Zoonoses and Public Health. 2020;67(6):658–72.

1266. Roulleau F, Paul MC, Goutard FL, Roger FL, Holl D, Thanapongtharm W, et al. Quantitative assessment of a spatial multicriteria model for highly pathogenic avian influenza H5N1 in Thailand, and application in Cambodia. Sci Rep. 2016;6:31096.

1267. Liao CM, Chio CP, Cheng YH, Hsieh NH, Chen WY, Chen SC. Quantitative links between arsenic exposure and influenza A (H1N1) infection-associated lung function exacerbations risk. Risk Anal. 2011;31(8):1281–94.

1268. Ramaswamy S, Chakraborty A, Akshata JS, Raghu BP, Nagaraja C. Radiological presentation of H1N1 influenza in the 2017 outbreak in India and correlation with patient outcome. Curr Respir Med Rev. 2018;14(4):237–44.

1269. Shanta IS, Hasnat MA, Zeidner N, Gurley ES, Azziz-Baumgartner E, Sharker MAY, et al. Raising Backyard Poultry in Rural Bangladesh: Financial and Nutritional Benefits, but Persistent Risky Practices. Transbound Emerg Dis. 2017;64(5):1454–64.

1270. Garg S, Thongcharoen P, Praphasiri P, Chitwarakorn A, Sathirapanya P, Fernandez S, et al. Randomized controlled trial to compare immunogenicity of standard-dose intramuscular versus intradermal trivalent inactivated influenza vaccine in HIV-infected men who have sex with men in Bangkok, Thailand. Clinical Infectious Diseases. 2016;62(3):383–91.

1271. Kurian A, Dandapat P, Jacob S, Francis J. Ranking of zoonotic diseases using composite index method: An illustration in Indian context. Indian Journal of Animal Sciences. 2014;84(4):357–63.

1272. Wei HL, Bai GR, Mweene AS, Zhou YC, Cong YL, Pu J, et al. Rapid detection of avian influenza virus a and subtype H5N1 by single step multiplex reverse transcription-polymerase chain reaction. Virus Genes. 2006;32(3):261–7.

1273. Shankar BP, Gowda RNS, Pattnaik B, Manjunath Prabhu BH, Patil SS, Pradhan HK. Rapid Detection of Highly Pathogenic Avian Influenza H5N1 Virus by TaqMan Reverse Transcriptase-Polymerase Chain Reaction. International Journal of Poultry Science. 2009;8(3):260–3.

1274. Maitreyi RS, Broor S, Kabra SK, Ghosh M, Seth P, Dar L, et al. Rapid detection of respiratory viruses by centrifugation enhanced cultures from children with acute lower respiratory tract infections. Journal of Clinical Virology. 2000;16(1):41–7.

1275. Imai M, Ninomiya A, Minekawa H, Notomi T, Ishizaki T, Van Tu P, et al. Rapid diagnosis of H5N1 avian influenza virus infection by newly developed influenza H5 hemagglutinin gene-specific loop-mediated isothermal amplification method. J Virol Methods. 2007;141(2):173–80.

1276. Sampath R, Hall TA, Massire C, Li F, Blyn LB, Eshoo MW, et al. Rapid identification of emerging infectious agents using PCR and electrospray ionization mass spectrometry. Ann N Y Acad Sci. 2007;1102(1):109–20.

1277. Dapat C, Suzuki Y, Saito R, Kyaw Y, Myint YY, Lin N, et al. Rare influenza A (H3N2) variants with reduced sensitivity to antiviral drugs. Emerging Infectious Diseases. 2010;16(3):493–6.

1278. Broor S, Dawood FS, Pandey BG, Saha S, Gupta V, Krishnan A, et al. Rates of respiratory virus-associated hospitalization in children aged <5 years in rural northern India. Journal of Infection. 2014;68(3):281–9.

1279. Spreeuwenberg P, Kroneman M, Paget J. Reassessing the Global Mortality Burden of the 1918 Influenza Pandemic. Am J Epidemiol. 2018;187(12):2561–7.

1280. Monne I, Yamage M, Dauphin G, Claes F, Ahmed G, Giasuddin M, et al. Reassortant avian influenza A(H5N1) viruses with H9N2-PB1 gene in poultry, Bangladesh. Emerging Infectious Diseases. 2013;19(10):1630–4.

1281. Jackson S, Van Hoeven N, Chen LM, Maines TR, Cox NJ, Katz JM, et al. Reassortment between avian H5N1 and human H3N2 influenza viruses in ferrets: a public health risk assessment. J Virol. 2009;83(16):8131–40.

1282. Karo-Karo D, Bodewes R, Wibawa H, Artika IM, Pribadi ES, Diyantoro D, et al. Reassortments among avian influenza A(H5N1) viruses circulating in Indonesia, 2015-2016. Emerging Infectious Diseases. 2019;25(3):465–72.

1283. Wibowo MH, Anggoro D, Amanu S, Wahyuni A, Untari T, Artanto S, et al. Receptor binding and antigenic site analysis of hemagglutinin gene fragments of avian influenza virus serotype H5N1 isolated from Indonesia. Pakistan Veterinary Journal. 2017;37(2):123–8.

1284. Pawar SD, Parkhi SS, Koratkar SS, Mishra AC. Receptor specificity and erythrocyte binding preferences of avian influenza viruses isolated from India. Virology Journal [Internet]. 2012;9. Available from: https://www.scopus.com/inward/record.uri?eid=2-s2.0-84867901193&doi=10.1186%2f1743-422X-9-251&partnerID=40&md5=2cba0e0f1185680557f0be592148f1ae

1285. Zaman M, Gasimov V, Oner AF, Dogan N, Adisasmito W, Coker R, et al. Recognizing true H5N1 infections in humans during confirmed outbreaks. Journal of Infection in Developing Countries. 2014;8(2):202–7.

1286. Kreijtz JHCM, Suezer Y, de Mutsert G, van den Brand JMA, van Amerongen G, Schnierle BS, et al. Recombinant modified vaccinia virus Ankara expressing the hemagglutinin gene confers protection against homologous and heterologous H5N1 influenza virus infections in macaques. J Infect Dis. 2009;199(3):405–13.

1287. Lange A. Reconstruction of disease transmission rates: Applications to measles, dengue, and influenza. J Theor Biol. 2016;400:138–53.

1288. Dash PK, Sharma S, Kumar JS, Krishna S, Siddappa S, Pattabiraman C, et al. Recovery of five complete influenza A(H1N1)pdm09 genome sequences from the 2015 influenza outbreak in India by metagenomic sequencing. Genome Announce. 2018;6(26):e00511-18.

1289. Koul P, Khan U, Bhat K, Saha S, Lal R, Broor S, et al. Recrudescent wave of A/H1N1pdm09 influenza viruses in winter 2012-2013 in Kashmir, India. PLoS Currents [Internet]. 2013;5. Available from: https://www.scopus.com/inward/record.uri?eid=2-s2.0-84916202410&doi=10.1371%2fcurrents.outbreaks.f1241c3a2625fc7a81bf25eea81f66e6&partnerID=40&md5=881794dccc20e36d67772def83d2b400

1290. Apisarnthanarak A, Uyeki TM, Puthavathana P, Kitphati R, Mundy LM. Reduction of seasonal influenza transmission among healthcare workers in an intensive care unit: a 4-year intervention study in Thailand. Infect Control Hosp Epidemiol. 2010;31(10):996–1003.

1291. Wiwanitkit V. Renal failure in swine flu: an appraisal from Thailand’s story. Ren Fail. 2010;32(1):150.

1292. El-Shesheny R, Feeroz MM, Krauss S, Vogel P, McKenzie P, Webby RJ, et al. Replication and pathogenic potential of influenza A virus subtypes H3, H7, and H15 from free-range ducks in Bangladesh in mammals article. Emerging Microbes and Infections [Internet]. 2018;7(1). Available from: https://www.scopus.com/inward/record.uri?eid=2-s2.0-85045925616&doi=10.1038%2fs41426-018-0072-7&partnerID=40&md5=1723bcf5c72dc5bd307a0414b157cfef

1293. Lenny BJ, Shanmuganatham K, Sonnberg S, Feeroz MM, Alam SMR, Hasan MK, et al. Replication capacity of avian influenza a(H9n2) virus in pet birds and mammals, Bangladesh. Emerging Infectious Diseases. 2015;21(12):2174–7.

1294. Jennings LC, Smith DW, Chan PKS. Report of the first Asia-Pacific Forum on antiviral treatment of influenza, Asia-Pacific Alliance for the Control of Influenza, Bangkok, 14 June 2012. Influenza Other Respir Viruses. 2013;7(6):987–90.

1295. Jennings LC, Smith DW, Chan PKS. Report of the first Asia-Pacific influenza summit, Asia-Pacific Alliance for the Control of Influenza (APACI), Bangkok, 12-13 June 2012. Influenza Other Respir Viruses. 2013;7(6):991–5.

1296. Li YG, Chittaganpitch M, Waicharoen S, Kanai Y, Bai GR, Kameoka M, et al. Research Characterization of H5N1 influenza viruses isolated from humans in vitro. Virology Journal [Internet]. 2010;7(1). Available from: https://www.scopus.com/inward/record.uri?eid=2-s2.0-77952852819&doi=10.1186%2f1743-422X-7-112&partnerID=40&md5=75702af95f892f87add818566981c16c

1297. Astrahan P, Arkin IT. Resistance characteristics of influenza to amino-adamantyls. Biochim Biophys Acta. 2011;1808(2):547–53.

1298. Rahman MZ, Islam M, Alam S, Sumiya MK, Goswami DR, Rahman M, et al. Respiratory and febrile illnesses in children due to human parainfluenza virus type 4 (HPIV4) and human coronavirus (HCOV) OC43 in Dhaka, Bangladesh. Am J Trop Med Hyg. 2019;101(5):260.

1299. Chierakul N, Rittayamai N, Nana A. Respiratory complications of adult patients with novel influenza A (H1N1) virus infection in Thailand. Respirology. 2009;14:A135.

1300. Sonawane AA, Shastri J, Bavdekar SB. Respiratory Pathogens in Infants Diagnosed with Acute Lower Respiratory Tract Infection in a Tertiary Care Hospital of Western India Using Multiplex Real Time PCR. Indian Journal of Pediatrics. 2019;86(5):433–8.

1301. Thongpan I, Vongpunsawad S, Poovorawan Y. Respiratory syncytial virus infection trend is associated with meteorological factors. Sci Rep. 2020;10(1):10931.

1302. Thongpan I, Suntronwong N, Vichaiwattana P, Wanlapakorn N, Vongpunsawad S, Poovorawan Y. Respiratory syncytial virus, human metapneumovirus, and influenza virus infection in Bangkok, 2016-2017. PeerJ. 2019;7:e6748.

1303. Emanuels A, Hawes SE, Newman KL, Martin ET, Englund JA, Tielsch JM, et al. Respiratory viral coinfection in a birth cohort of infants in rural Nepal. Influenza Other Respir Viruses. 2020;14(6):739–46.

1304. Tillekeratne LG, Bodinayake CK, Simmons R, Nagahawatte A, Devasiri V, Arachchi WK, et al. Respiratory viral infection: An underappreciated cause of acute febrile illness admissions in southern Sri Lanka. American Journal of Tropical Medicine and Hygiene. 2019;100(3):672–80.

1305. Bharaj P, Sullender WM, Kabra SK, Mani K, Cherian J, Tyagi V, et al. Respiratory viral infections detected by multiplex PCR among pediatric patients with lower respiratory tract infections seen at an urban hospital in Delhi from 2005 to 2007. Virology Journal [Internet]. 2009;6. Available from: https://www.scopus.com/inward/record.uri?eid=2-s2.0-67949108097&doi=10.1186%2f1743-422X-6-89&partnerID=40&md5=55c0b03f98dd1f2e80b5f6fa17dfed04

1306. Turner P, Carrara V, Turner C, Cicelia N, Watthanaworawit W, Nosten F, et al. Respiratory virus surveillance in hospitalized pneumonia patients on the Thailand-Myanmar border. Int J Infect Dis. 2012;16:e147.

1307. Taylor S, Lopez P, Weckx L, Borja-Tabora C, Ulloa-Gutierrez R, Lazcano-Ponce E, et al. Respiratory viruses and influenza-like illness: Epidemiology and outcomes in children aged 6 months to 10 years in a multi-country population sample. Journal of Infection. 2017;74(1):29–41.

1308. Prasetyo AA, Desyardi MN, Tanamas J, Suradi, Reviono, Harsini, et al. Respiratory viruses and torque teno virus in adults with acute respiratory infections. Intervirology. 2015;58(1):57–68.

1309. Homaira N, Luby SP, Hossain K, Islam K, Ahmed M, Rahman M, et al. Respiratory Viruses Associated Hospitalization among Children Aged <5 Years in Bangladesh: 2010-2014. PLoS One. 2016;11(2):e0147982.

1310. Wannachai T, Kamalaporn H, Preutthipan A. Respiratory viruses detection using multiplex PCR in children with severe community acquired pneumonia at Ramathibodi Hospital, Thailand. Paediatr Respir Rev. 2012;13:S49–50.

1311. Yeolekar LR, Damle RG, Kamat AN, Khude MR, Simha V, Pandit AN. Respiratory viruses in acute respiratory tract infections in Western India. Indian Journal of Pediatrics. 2008;75(4):341–5.

1312. Mathisen M, Strand TA, Valentiner-Branth P, Chandyo RK, Basnet S, Sharma BN, et al. Respiratory viruses in nepalese children with and without pneumonia: A case-control study. Pediatric Infectious Disease Journal. 2010;29(8):731–5.

1313. Koul PA, Mir H, Saha S, Chadha MS, Potdar V, Widdowson MA, et al. Respiratory viruses in returning Hajj & Umrah pilgrims with acute respiratory illness in 2014-2015. Indian J Med Res. 2018;148(3):329–33.

1314. Chunsuttiwat S. Response to avian influenza and preparedness for pandemic influenza: Thailand’s experience. Respirology. 2008;13:S36-40.

1315. Moore M, Dausey DJ. Response to the 2009-H1N1 influenza pandemic in the Mekong Basin: surveys of country health leaders. BMC Res Notes. 2011;4:361.

1316. Retrospective analysis of Oseltamivir and Zanamivir in patients of H1N1 influenza in a tertiary care hospital in Western India. Int J Pharma Bio Sci. 2015;6(1):P672–8.

1317. Souris M, Gonzalez JP, Shanmugasundaram J, Corvest V, Kittayapong P. Retrospective space-time analysis of H5N1 Avian Influenza emergence in Thailand. International Journal of Health Geographics [Internet]. 2010;9. Available from: https://www.scopus.com/inward/record.uri?eid=2-s2.0-77649279212&doi=10.1186%2f1476-072X-9-3&partnerID=40&md5=9505cc595db52ce2794e55b6ab81f391

1318. Sreta D, Jittimanee S, Charoenvisal N, Amonsin A, Kitikoon P, Thanawongnuwech R. Retrospective swine influenza serological surveillance in the four highest pig density provinces of Thailand before the introduction of the 2009 pandemic Influenza A virus subtype H1N1 using various antibody detection assays. Journal of Veterinary Diagnostic Investigation. 2013;25(1):45–53.

1319. Rutvisuttinunt W, Klungthong C, Thaisomboonsuk B, Chinnawirotpisan P, Ajariyakhajorn C, Manasatienkij W, et al. Retrospective use of next-generation sequencing reveals the presence of Enteroviruses in acute influenza-like illness respiratory samples collected in South/South-East Asia during 2010–2013. Journal of Clinical Virology. 2017;94:91–9.

1320. Jung EJ, Lee KH, Seong BL. Reverse genetic platform for inactivated and live-attenuated influenza vaccine. Exp Mol Med. 2010;42(2):116–21.

1321. Parvin R, Begum JA, Nooruzzaman M, Chowdhury EH, Islam MR, Vahlenkamp TW. Review analysis and impact of co-circulating H5N1 and H9N2 avian influenza viruses in Bangladesh. Epidemiol Infect. 2018;146(10):1259–66.

1322. Lye DCB, Ang BSP, Leo YS. Review of human infections with avian influenza H5N1 and proposed local clinical management guideline. Ann Acad Med Singap. 2007;36(4):285–92.

1323. Rao R, Vttaldas RB, Dsouza JP. Rising threat of H1N1 cases in Dakshina Kannada, India: An epidemiological assessment from January 2011 to August 2017. Indian Journal of Community Health. 2019;31(3):413–6.

1324. Gilbert M, Pfeiffer DU. Risk factor modelling of the spatio-temporal patterns of highly pathogenic avian influenza (HPAIV) H5N1: a review. Spat Spatiotemporal Epidemiol. 2012;3(3):173–83.

1325. Loth L, Gilbert M, Osmani MG, Kalam AM, Xiao X. Risk factors and clusters of highly pathogenic avian influenza H5N1 outbreaks in Bangladesh. Preventive Veterinary Medicine. 2010;96(1):104–13.

1326. Scott EM, Kuypers J, Englund JA, Chu HY, Stewart L, Katz J, et al. Risk factors and dynamics of household transmission of respiratory syncytial virus and other respiratory viruses in rural Nepal. Lancet Global Health. 2016;4:28.

1327. Scott EM, Magaret A, Kuypers J, Tielsch JM, Katz J, Khatry SK, et al. Risk factors and patterns of household clusters of respiratory viruses in rural Nepal. Epidemiology and infection. 2019;147:e288.

1328. Gompo TR, Shah BR, Karki S, Koirala P, Maharjan M, Bhatt DD. Risk factors associated with Avian Influenza subtype H9 outbreaks in poultry farms in Kathmandu valley, Nepal. PLoS One. 2020;15(4):e0223550.

1329. Balaganesakumar SR, Murhekar MV, Swamy KK, Kumar MR, Manickam P, Pandian P. Risk factors associated with death among influenza A (H1N1) patients, Tamil Nadu, India, 2010. J Postgrad Med. 2013;59(1):9–14.

1330. Aditama TY, Samaan G, Kusriastuti R, Purba WH, Misriyah, Santoso H, et al. Risk factors for cluster outbreaks of avian influenza A H5N1 Infection, Indonesia. Clinical Infectious Diseases. 2011;53(12):1237–44.

1331. Kumar T, Bhatia D, Aggarwal S, Kumar R, Lakshmi P, Dikid T, et al. Risk factors for death among hospitalized influenza A (H1N1) patients, Punjab, India-2013. Int J Infect Dis. 2014;21:228.

1332. Kumar T, Bhatia D, Maha Lakshmi PV, Laserson KF, Narain JP, Kumar R. Risk factors for death during a resurgence of influenza-A (H1N1) pdm09 in Punjab State in 2013. Indian J Public Health. 2017;61(1):9–13.

1333. Beaudoin AL, Kitikoon P, Schreiner PJ, Singer RS, Sasipreeyajan J, Amonsin A, et al. Risk factors for exposure to influenza a viruses, including subtype H5 viruses, in Thai free-grazing ducks. Transboundary and Emerging Diseases. 2014;61(4):362–74.

1334. Paul M, Wongnarkpet S, Gasqui P, Poolkhet C, Thongratsakul S, Ducrot C, et al. Risk factors for highly pathogenic avian influenza (HPAI) H5N1 infection in backyard chicken farms, Thailand. Acta Tropica. 2011;118(3):209–16.

1335. Osmani MG, Thornton RN, Dhand NK, Hoque MA, Milon SMA, Kalam MA, et al. Risk Factors for Highly Pathogenic Avian Influenza in Commercial Layer Chicken Farms in Bangladesh During 2011. Transboundary and Emerging Diseases. 2014;61(6):e44–51.

1336. Moolasart V, Manosuthi W. Risk factors for hospitalization among children with influenza B infection. Southeast Asian J Trop Med Public Health. 2014;45(3):622–9.

1337. van Kerkhove MD, Vandemaele KAH, Shinde V, Jaramillo-Gutierrez G, Koukounari A, Donnelly CA, et al. Risk factors for severe outcomes following 2009 influenza a (H1N1) infection: A global pooled analysis. PLoS Medicine [Internet]. 2011;8(7). Available from: https://www.scopus.com/inward/record.uri?eid=2-s2.0-79960936455&doi=10.1371%2fjournal.pmed.1001053&partnerID=40&md5=820c9132025816c0c971aa0a1e95cfea

1338. Yupiana Y, de Vlas SJ, Adnan NM, Richardus JH. Risk factors of poultry outbreaks and human cases of H5N1 avian influenza virus infection in West Java Province, Indonesia. International Journal of Infectious Diseases. 2010;14(9):e800–5.

1339. Biswas PK, Rahman MH, Das A, Ahmed SSU, Giasuddin M, Christensen JP. Risk for highly pathogenic avian influenza H5N1 virus infection in chickens in small-scale commercial farms, in a high-risk area, Bangladesh, 2008. Transboundary and Emerging Diseases. 2011;58(6):519–25.

1340. Biswas PK, Christensen JP, Ahmed SSU, Das A, Rahman MH, Barua H, et al. Risk for infection with highly pathogenic avian influenza virus (H5N1) in backyard chickens, Bangladesh. Emerging Infectious Diseases. 2009;15(12):1931–6.

1341. Netrabukkana P, Robertson I, Fenwick S, Kasemsuwan S, Wongsathapornchai K. Risk of influenza A transmission at the pig-human interface in small pig farms in rural Thailand. Int J Antimicrob Agents. 2013;42:S105.

1342. Newman KL, Gustafson K, Englund JA, Khatry SK, LeClerq SC, Tielsch JM, et al. Risk of respiratory infection following diarrhea among adult women and infants in Nepal. American Journal of Tropical Medicine and Hygiene. 2020;102(1):28–30.

1343. Zhang Z, Chen D, Chen Y, Davies TM, Vaillancourt JP, Liu W. Risk signals of an influenza pandemic caused by highly pathogenic avian influenza subtype H5N1: spatio-temporal perspectives. Vet J. 2012;192(3):417–21.

1344. Thanapongtharm W, Van Boeckel TP, Biradar C, Xiao XM, Gilbert M. Rivers and flooded areas identified by medium-resolution remote sensing improve risk prediction of the highly pathogenic avian influenza H5N1 in Thailand. Geospat Health. 2013;8(1):193–201.

1345. Mathisen M, Strand TA, Sharma BN, Chandyo RK, Valentiner-Branth P, Basnet S, et al. RNA viruses in community-acquired childhood pneumonia in semi-urban Nepal; a cross-sectional study. BMC Medicine [Internet]. 2009;7. Available from: https://www.scopus.com/inward/record.uri?eid=2-s2.0-68949219603&doi=10.1186%2f1741-7015-7-35&partnerID=40&md5=4ddb498b5bd5ba42d56d9ce194a8eae8

1346. Mathisen M, Basnet S, Sharma A, Shrestha PS, Sharma BN, Valentiner-Branth P, et al. RNA viruses in young Nepalese children hospitalized with severe pneumonia. Pediatric Infectious Disease Journal. 2011;30(12):1032–6.

1347. Barman S, Marinova-Petkova A, Hasan MK, Akhtar S, El-Shesheny R, Turner JC, et al. Role of domestic ducks in the emergence of a new genotype of highly pathogenic H5N1 avian influenza A viruses in Bangladesh. Emerg Microbes Infect. 2017;6(8):e72.

1348. Samina M, Tabish SA, Mufti SA, Ajaz M, Rehana K, Panditha K, et al. Role of Hospital in pandemic: Our experience. Journal International Medical Sciences Academy. 2012;25(3):201–4.

1349. Arankalle VA, Lole KS, Arya RP, Tripathy AS, Ramdasi AY, Chadha MS, et al. Role of host immune response and viral load in the differential outcome of pandemic H1N1 (2009) influenza virus infection in Indian patients. PLoS One. 2010;5(10).

1350. Singh BB, Gajadhar AA. Role of India’s wildlife in the emergence and re-emergence of zoonotic pathogens, risk factors and public health implications. Acta Tropica. 2014;138:67–77.

1351. Sharma R, Karad A, Dash B, Chauhan L. Role of media scanning and verification system as a supplemental tool to disease outbreak detection & reporting under integrated disease surveillance project (IDSP)-India. Int J Infect Dis. 2012;16:e10.

1352. Phonrat B, Pitisuttithum P, Chamnanchanunt S, Puthavathana P, Ngaosuwankul N, Louisirirotchanakul S, et al. Safety and immune responses following administration of H1N1 live attenuated influenza vaccine in Thais. Vaccine. 2013;31(11):1503–9.

1353. Pitisuttithum P, Boonnak K, Chamnanchanunt S, Puthavathana P, Luvira V, Lerdsamran H, et al. Safety and immunogenicity of a live attenuated influenza H5 candidate vaccine strain A/17/turkey/Turkey/05/133 H5N2 and its priming effects for potential pre-pandemic use: a randomised, double-blind, placebo-controlled trial. The Lancet Infectious Diseases. 2017;17(8):833–42.

1354. Kulkarni PS, Manjunath K, Agarkhedkar S. Safety and immunogenicity of an adjuvanted whole virion, inactivated A (H1N1) 2009 influenza vaccine in young and elderly adults, and children. Vaccine. 2012;31(1):20–2.

1355. Rudenko L, Desheva J, Korovkin S, Mironov A, Rekstin A, Grigorieva E, et al. Safety and immunogenicity of live attenuated influenza reassortant H5 vaccine (phase I-II clinical trials). Influenza Other Respir Viruses. 2008;2(6):203–9.

1356. Ortiz JR, Goswami D, Lewis KDC, Sharmeen AT, Ahmed M, Rahman M, et al. Safety of Russian-backbone seasonal trivalent, live-attenuated influenza vaccine in a phase II randomized placebo-controlled clinical trial among children in urban Bangladesh. Vaccine. 2015;33(29):3415–21.

1357. Nigwekar PV, Kumar A, Padbidri VV, Choudhury A, Chaudhari AB, Kulkarni PS. Safety of Russian-Backbone Trivalent, Live Attenuated Seasonal Influenza Vaccine in Healthy Subjects: Open-Label, Non-randomized Phase 4 Study. Drug Saf. 2018;41(2):171–7.

1358. Kyriakis CS, De Vleeschauwer A, Barbé F, Bublot M, Van Reeth K. Safety, immunogenicity and efficacy of poxvirus-based vector vaccines expressing the haemagglutinin gene of a highly pathogenic H5N1 avian influenza virus in pigs. Vaccine. 2009;27(16):2258–64.

1359. Ratanakorn P, Suwanpakdee S, Wiriyarat W, Eiamampai K, Chaichoune K, Wiratsudakul A, et al. Satellite telemetry tracks flyways of Asian Openbill storks in relation to H5N1 avian influenza spread and ecological change. BMC Vet Res. 2018;14(1):349.

1360. Ratanakorn P, Wiratsudakul A, Wiriyarat W, Eiamampai K, Farmer AH, Webster RG, et al. Satellite tracking on the flyways of brown-headed gulls and their potential role in the spread of highly pathogenic avian influenza H5N1 virus. PLoS One. 2012;7(11):e49939.

1361. Henning J, Wibawa H, Morton J, Usman TB, Junaidi A, Meers J. Scavenging ducks and transmission of highly pathogenic avian influenza, Java, Indonesia. Emerging Infectious Diseases. 2010;16(8):1244–50.

1362. Ganju SA, Gautam N, Singh DV, Walia S, Kanga A. Scenario of pandemic 2009 H1N1 in Himachal Pradesh, India in 2015: Is it resurgence? Am J Respir Crit Care Med [Internet]. 2017;195. Available from: http://www.atsjournals.org/doi/pdf/10.1164/ajrccm-conference.2017.195.1_MeetingAbstracts.A6056

1363. Cauchemez S, Van Kerkhove MD, Archer BN, Cetron M, Cowling BJ, Grove P, et al. School closures during the 2009 influenza pandemic: national and local experiences. BMC Infect Dis. 2014;14:207.

1364. Paudel S. Scope and challenges of One Health approach in Nepal. Int J Infect Dis. 2020;101:404.

1365. Sharma J, Bhattacharyya D, Poddar K, Pavithran TC, Thakur H. Scoping review of non-pharmacological interventions to control H1N1 in India. Clin Epidemiol Global Health. 2019;7(3):504–8.

1366. Maria John KM, Enkhtaivan G, Ayyanar M, Jin K, Yeon JB, Kim DH. Screening of ethnic medicinal plants of South India against influenza (H1N1) and their antioxidant activity. Saudi J Biol Sci. 2015;22(2):191–7.

1367. Supawat K, Chittaganpitch M, Waicharoen S, Pattamadilok S, Sriwantana B, Sawanpanyalert P, et al. Seasonal and avian influenza surveillance in Thailand 2004-2008. Influ Other Respir Viruses. 2010;4:12.

1368. Anand M, Nimmala P. Seasonal incidence of respiratory viral infections in Telangana, India: utility of a multiplex PCR assay to bridge the knowledge gap. Tropical Medicine and International Health. 2020;25(12):1503–9.

1369. Lafond KE, Praptiningsih CY, Mangiri A, Syarif M, Triada R, Mulyadi E, et al. Seasonal influenza and avian influenza A(H5N1) virus surveillance among inpatients and outpatients, East Jakarta, Indonesia, 2011-2014. Emerging Infectious Diseases. 2019;25(11):2031–9.

1370. Akhtar Z, Chowdhury F, Rahman M, Ghosh PK, Ahmmed MK, Islam MA, et al. Seasonal influenza during the COVID-19 pandemic in Bangladesh. PLoS One. 2021;16(8):e0255646.

1371. Seasonal influenza surveillance (2009-2017) for pandemic preparedness in the WHO South-East Asia Region. WHO South East Asia J Public Health. 2020;9(1):55–65.

1372. Meeyai A, Praditsitthikorn N, Kotirum S, Kulpeng W, Putthasri W, Cooper BS, et al. Seasonal Influenza Vaccination for Children in Thailand: A Cost-Effectiveness Analysis. PLoS Medicine [Internet]. 2015;12(5). Available from: https://www.scopus.com/inward/record.uri?eid=2-s2.0-84930532938&doi=10.1371%2fjournal.pmed.1001829&partnerID=40&md5=46d2b80e86c270ad8cd1f1308f3a5d56

1373. Mantel C, Chu SY, Hyde TB, Lambach P. Seasonal influenza vaccination in middle-income countries: Assessment of immunization practices in Belarus, Morocco, and Thailand. Vaccine. 2020;38(2):212–9.

1374. Owusu JT, Prapasiri P, Ditsungnoen D, Leetongin G, Yoocharoen P, Rattanayot J, et al. Seasonal influenza vaccine coverage among high-risk populations in Thailand, 2010-2012. Vaccine. 2015;33(5):742–7.

1375. Palache A. Seasonal influenza vaccine provision in 157 countries (2004-2009) and the potential influence of national public health policies. Vaccine. 2011;29(51):9459–66.

1376. Morse SS, Murray EJ. Seasonal oscillation of human infection with influenza A/H5N1 in Egypt and Indonesia. PLoS ONE. 2011;6(9):e24042.

1377. Mathur MB, Patel RB, Gould M, Uyeki TM, Bhattacharya J, Xiao Y, et al. Seasonal patterns in human A (H5N1) virus infection: analysis of global cases. PLoS One. 2014;9(9):e106171.

1378. Farrar DS, Awasthi S, Fadel SA, Kumar R, Sinha A, Fu SH, et al. Seasonal variation and etiologic inferences of childhood pneumonia and diarrhea mortality in India. Elife. 2019;8.

1379. Sartika T, Sulandari S, Zein MSA. Selection of Mx gene genotype as genetic marker for Avian Influenza resistance in Indonesian native chicken. BMC Proc. 2011;5:S37.

1380. Anup J, Jazeel A, Robin S, Santhosh D, Rao C, Aswathyraj S, et al. Sentinel influenza surveillance among inpatient cases in a district level hospital in Kerala, India from 2015 to 2017. VirusDisease. 2018;29(2):234.

1381. Boonyapisitsopa S, Chaiyawong S, Nonthabenjawan N, Jairak W, Prakairungnamthip D, Bunpapong N, et al. Sentinel model for influenza A virus monitoring in free-grazing ducks in Thailand. Veterinary Microbiology. 2016;182:35–43.

1382. Jarman RG, Bhoomiboonchoo P, Yoon IK, Gibbons RV, Simasathien S, Watanaveeradej V, et al. Sentinel surveillance for influenza in Phramongkutklao Hospital in Bangkok Thailand. Am J Trop Med Hyg. 2011;85(6):422.

1383. Bharmoria A, Vaish VB, Mani KR. Seroconversion and immunogenicity evaluation of seasonal influenza vaccine in mice model. VirusDisease. 2016;27(3):260–5.

1384. Pawar SD, Jamgaonkar AV, Umarani UB, Kode SS. Seroepidemiology of avian influenza H5N1, H9N2 & Newcastle disease viruses during 1954 to 1981 in India. Indian J Med Res. 2016;144:472–6.

1385. Yeolekar LR, Kulkarni PB, Chadha MS, Rao BL. Seroepidemiology of influenza in Pune, India. Indian J Med Res. 2001;114:121–6.

1386. Tandale BV, Pawar SD, Gurav YK, Chadha MS, Koratkar SS, Shelke VN, et al. Seroepidemiology of pandemic influenza A (H1N1) 2009 virus infections in Pune, India. BMC Infectious Diseases [Internet]. 2010;10. Available from: https://www.scopus.com/inward/record.uri?eid=2-s2.0-77955790217&doi=10.1186%2f1471-2334-10-255&partnerID=40&md5=e5c88b3a1b20d054290b1c381da7ba02

1387. Shimizu K, Wulandari L, Poetranto ED, Setyoningrum RA, Yudhawati R, Sholikhah A, et al. Seroevidence for a high prevalence of subclinical infection with avian influenza a(h5n1) virus among workers in a live-poultry market in Indonesia. Journal of Infectious Diseases. 2016;214(12):1929–36.

1388. Garg S, Olsen SJ, Fernandez S, Muangchana C, Rungrojcharoenkit K, Prapasiri P, et al. Seroincidence of influenza among HIV-infected and HIV-uninfected men during the 2009 H1N1 influenza pandemic, Bangkok, Thailand. Open Forum Infectious Diseases [Internet]. 2014;1(3). Available from: https://www.scopus.com/inward/record.uri?eid=2-s2.0-84978328179&doi=10.1093%2fofid%2fofu082&partnerID=40&md5=eb144686576f6551a4f68b71f4cf11af

1389. Shinde PV, Koratkar SS, Pawar SD, Kale SD, Rawankar AS, Mishra AC. Serologic evidence of avian influenza H9N2 and paramyxovirus type 1 infectionin emus (dromaius novaehollandiae) in India. Avian Diseases. 2012;56(1):257–60.

1390. Prachayangprecha S, Makkoch J, Payungporn S, Chieochansin T, Vuthitanachot C, Vuthitanachot V, et al. Serological analysis of human pandemic influenza (H1N1) in Thailand. Journal of Health, Population and Nutrition. 2010;28(6):537–44.

1391. Helmi TZ, Haryanto A. Serological and molecular analysis of avian influenza virus subtype h5 isolated from aceh province in Indonesia. International Journal of Virology. 2017;13(1):62–7.

1392. Sarker RD, Giasuddin M, Chowdhury EH, Islam MR. Serological and virological surveillance of avian influenza virus in domestic ducks of the north-east region of Bangladesh. BMC Veterinary Research [Internet]. 2017;13(1). Available from: https://www.scopus.com/inward/record.uri?eid=2-s2.0-85027587663&doi=10.1186%2fs12917-017-1104-6&partnerID=40&md5=2003b71f752f68a77cf7cd4192b7548b

1393. Hassan MM, El Zowalaty ME, Islam A, Rahman MM, Chowdhury MNU, Nine HSMZ, et al. Serological evidence of avian influenza in captive wild birds in a zoo and two safari parks in Bangladesh. Veterinary Sciences [Internet]. 2020;7(3). Available from: https://www.scopus.com/inward/record.uri?eid=2-s2.0-85091597416&doi=10.3390%2fVETSCI7030122&partnerID=40&md5=c1b1739fc8d3643477428d505f258be1

1394. Sangkachai N, Thongdee M, Chaiwattanarungruengpaisan S, Buddhirongawatr R, Chamsai T, Poltep K, et al. Serological evidence of influenza virus infection in captive wild felids, Thailand. Journal of Veterinary Medical Science. 2019;81(9):1341–7.

1395. Kitikoon P, Sreta D, Tuanudom R, Amonsin A, Suradhat S, Oraveerakul K, et al. Serological evidence of pig-to-human influenza virus transmission on Thai swine farms. Veterinary Microbiology. 2011;148(2):413–8.

1396. Kosalaraksa P, Srirompotong U, Newman RW, Lumbiganon P, Wood JM. Serological response to trivalent inactive influenza vaccine in HIV-infected children with different immunologic status. Vaccine. 2011;29(16):3055–60.

1397. Uddin MK, Ahasan MS, Islam MR, Islam MMM, Hoque MF. Seroprevalance of Influenza A in swine population of Rangamati and Khagracchari districts. Journal of Advanced Veterinary and Animal Research [Internet]. 2018;5(2). Available from: https://www.scopus.com/inward/record.uri?eid=2-s2.0-85048059816&doi=10.5455%2fjavar.2018.e269&partnerID=40&md5=a38d7502a600d9db996c270de5788099

1398. Monger VR, Stegeman JA, Koop G, Dukpa K, Tenzin T, Loeffen WLA. Seroprevalence and associated risk factors of important pig viral diseases in Bhutan. Preventive Veterinary Medicine. 2014;117(1):222–32.

1399. Chokephaibulkit K, Assanasen S, Apisarnthanarak A, Rongrungruang Y, Kachintorn K, Tuntiwattanapibul Y, et al. Seroprevalence of 2009 H1N1 virus infection and self-reported infection control practices among healthcare professionals following the first outbreak in Bangkok, Thailand. Influenza Other Respir Viruses. 2013;7(3):359–63.

1400. Nasreen S, Uddin Khan S, Azziz-Baumgartner E, Hancock K, Veguilla V, Wang D, et al. Seroprevalence of antibodies against highly pathogenic avian influenza A (H5N1) virus among poultry workers in Bangladesh, 2009. PLoS One. 2013;8(9):e73200.

1401. Dejpichai R, Laosiritaworn Y, Phuthavathana P, Uyeki TM, O’Reilly M, Yampikulsakul N, et al. Seroprevalence of antibodies to avian influenza virus A (H5N1) among residents of villages with human cases, Thailand, 2005. Emerging Infectious Diseases. 2009;15(5):756–60.

1402. Du Ry van Beest Holle MR, Setiawaty V, Pangesti KNA, Sedyaningsih ER. Seroprevalence of avian influenza A/H5N1 among poultry farmers in rural Indonesia, 2007. Southeast Asian Journal of Tropical Medicine and Public Health. 2010;41(5):1095–103.

1403. Novitasari D, Anwar C. Seroprevalence of Avian Influenza Virus Subtype H5 among Poultry Workers of Central Traditional Markets in Indonesia. Journal of World’s Poultry Research. 2020;10(4):643–8.

1404. Chanvatik S, Tangwangvivat R, Chaiyawong S, Prakairungnamthip D, Tuanudom R, Thontiravong A, et al. Seroprevalence of influenza A in domestic dogs in Thailand, 2013. Thai Journal of Veterinary Medicine. 2016;46(1):33–9.

1405. Chumsang S, Na Lampang K, Srikitjakarn L, Pringproa K. Seroprevalence of the viral pig diseases among backyard pigs in Chiang Mai, Thailand. Prev Vet Med. 2021;190:105330.

1406. Paungpin W, Wiriyarat W, Chaichoun K, Tiyanun E, Sangkachai N, Changsom D, et al. Serosurveillance for pandemic influenza A (H1N1) 2009 virus infection in domestic elephants, Thailand. PLoS One. 2017;12(10):e0186962.

1407. Biswas PK, Barua H, Uddin GMN, Biswas D, Ahad A, Debnath NC. Serosurvey of five viruses in chickens on smallholdings in Bangladesh. Preventive Veterinary Medicine. 2009;88(1):67–71.

1408. Apisarnthanarak A, Uyeki TM, Miller ER, Mundy LM. Serum sickness-like reaction associated with inactivated influenza vaccination among Thai Health Care Personnel: Risk factors and outcomes. Clinical Infectious Diseases. 2009;49(1):e18–22.

1409. Hanshaoworakul W, Simmerman JM, Narueponjirakul U, Sanasuttipun W, Shinde V, Kaewchana S, et al. Severe human influenza infections in Thailand: oseltamivir treatment and risk factors for fatal outcome. PLoS One. 2009;4(6):e6051.

1410. Liverani M, Teng S, Le MS, Coker R. Sharing public health data and information across borders: Lessons from Southeast Asia 11 Medical and Health Sciences 1117 Public Health and Health Services. Globalization and Health [Internet]. 2018;14(1). Available from: https://www.scopus.com/inward/record.uri?eid=2-s2.0-85054224078&doi=10.1186%2fs12992-018-0415-0&partnerID=40&md5=c9b4c7c738ac8b715f86216a5876b7f4

1411. Wiedenmayer KA, Weiss S, Chattopadhyay C, Mukherjee A, Kundu R, Ayé R, et al. Simplifying paediatric immunization with a fully liquid DTP-HepB-Hib combination vaccine: Evidence from a comparative time-motion study in India. Vaccine. 2009;27(5):655–9.

1412. Rutvisuttinunt W, Chinnawirotpisan P, Simasathien S, Shrestha SK, Yoon IK, Klungthong C, et al. Simultaneous and complete genome sequencing of influenza A and B with high coverage by Illumina MiSeq Platform. Journal of Virological Methods. 2013;193(2):394–404.

1413. Chantratita W, Sukasem C, Sirinavin S, Sankuntaw N, Srichantaratsamee C, Pasomsub E, et al. Simultaneous detection and subtyping of H274Y-positive influenza a (H1N1) using pyrosequencing. Journal of Infection in Developing Countries. 2011;5(5):348–52.

1414. Thontiravong A, Tantilertcharoen R, Tuanudom R, Sreta D, Thanawongnuwech R, Amonsin A, et al. Single-step multiplex reverse transcription polymerase chain reaction assay for detection and differentiation of the 2009 H1N1 influenza A virus pandemic in Thai swine populations. Journal of Veterinary Diagnostic Investigation. 2011;23(5):1017–21.

1415. Paudel M, Acharya B, Adhikari M. Social determinants that lead to poor knowledge about, and inappropriate precautionary practices towards, avian influenza among butchers in Kathmandu, Nepal. Infectious Diseases of Poverty [Internet]. 2013;2(1). Available from: https://www.scopus.com/inward/record.uri?eid=2-s2.0-84930190163&doi=10.1186%2f2049-9957-2-10&partnerID=40&md5=5eca577687e9028ed469f9e4955d1668

1416. Poolkhet C, Chairatanayuth P, Thongratsakul S, Yatbantoong N, Kasemsuwan S, Damchoey D, et al. Social network analysis for assessment of Avian influenza spread and trading patterns of backyard chickens in Nakhon Pathom, Suphan Buri and Ratchaburi, Thailand. Zoonoses and Public Health. 2013;60(6):448–55.

1417. Kurscheid J, Stevenson M, Durr PA, Toribio JALML, Kurscheid S, Ambarawati IGAA, et al. Social network analysis of the movement of poultry to and from live bird markets in Bali and Lombok, Indonesia. Transboundary and Emerging Diseases. 2017;64(6):2023–33.

1418. Poolkhet C, Chairatanayuth P, Thongratsakul S, Kasemsuwan S, Rukkwamsuk T. Social network analysis used to assess the relationship between the spread of avian influenza and movement patterns of backyard chickens in Ratchaburi, Thailand. Research in Veterinary Science. 2013;95(1):82–6.

1419. Basuno E, Yusdja Y, Ilham N. Socio-economic impacts of avian influenza outbreaks on small-scale producers in Indonesia. Transboundary and Emerging Diseases. 2010;57(1):7–10.

1420. Sundaram N, Schaetti C, Grize L, Purohit V, Joseph S, Schindler C, et al. Sociocultural determinants of anticipated acceptance of pandemic influenza vaccine in Pune, India: a community survey using mixed-methods. Int J Public Health. 2017;62(1):103–15.

1421. Kudale A, Purohit VS, Sundaram N, Schaetti C, Weiss MG. Socioeconomic, cultural and behavioural features of prior and anticipated influenza vaccine uptake in urban and rural Pune district, India: a mixed-methods case study. BMJ Open. 2013;3(2).

1422. Nimbalkar PM, Tripathi NK. Space-time epidemiology and effect of meteorological parameters on influenza-like illness in Phitsanulok, a northern province in Thailand. Geospat Health. 2016;11(3):447.

1423. Lopez D, Gunasekaran M, Murugan BS, Kaur H, Abbas KM. Spatial Big Data Analytics of Influenza Epidemic in Vellore, India. Proc IEEE Int Conf Big Data. 2014;2014:19–24.

1424. Nakapan S, Tripathi NK, Tipdecho T, Souris M. Spatial diffusion of influenza outbreak-related climate factors in Chiang Mai Province, Thailand. International Journal of Environmental Research and Public Health. 2012;9(11):3824–42.

1425. Dhingra MS, Dissanayake R, Negi AB, Oberoi M, Castellan D, Thrusfield M, et al. Spatio-temporal epidemiology of highly pathogenic avian influenza (subtype H5N1) in poultry in eastern India. Spatial and Spatio-temporal Epidemiology. 2014;11:45–57.

1426. Toft N, Ahmed SSU, Ersboll AK, Biswas PK, Christensen JP. Spatio-Temporal magnitude and direction of highly pathogenic avian influenza (H5N1) outbreaks in Bangladesh. PLoS ONE. 2011;6(9):e24324.

1427. Reyes O, Lee EC, Sah P, Viboud C, Chandra S, Bansal S. Spatiotemporal Patterns and Diffusion of the 1918 Influenza Pandemic in British India. Am J Epidemiol. 2018;187(12):2550–60.

1428. Horthongkham N, Srihtrakul T, Athipanyasilp N, Siritantikorn S, Kantakamalakul W, Poovorawan Y, et al. Specific antibody response of mice after immunization with COS-7 cell derived avian influenza virus (H5N1) recombinant proteins. J Immune Based Ther Vaccines. 2007;5:10.

1429. Kini S, Bhat R, Handattu K, Kousika P, Thunga C. Spectrum of influenza B viral infection in indian children: A tertiary centre experience. Journal of Nepal Paediatric Society. 2018;38(3):170–5.

1430. Jony MHK. Spectrum of respiratory pathogens in selected hospitals of Bangladesh. Int J Infect Dis. 2020;101:357.

1431. Roy Mukherjee T, Chanda S, Mullick S, De P, Dey-Sarkar M, Chawla-Sarkar M. Spectrum of respiratory viruses circulating in eastern India: prospective surveillance among patients with influenza-like illness during 2010-2011. J Med Virol. 2013;85(8):1459–65.

1432. Ruenphet S, Punyadarsaniya D, Jantafong T, Takehara K. Stability and virucidal efficacies using powder and liquid forms of fresh charcoal ash and slaked lime against Newcastle disease virus and Avian influenza virus. Vet World. 2019;12(1):1–6.

1433. Amoah S, Mishina M, Praphasiri P, Cao W, Kim JH, Liepkalns JS, et al. Standard-dose intradermal influenza vaccine elicits cellular immune responses similar to those of intramuscular vaccine in men with and those without hiv infection. Journal of Infectious Diseases. 2019;220(5):743–51.

1434. Swayne DE, Kapczynski D. Strategies and challenges for eliciting immunity against avian influenza virus in birds. Immunol Rev. 2008;225:314–31.

1435. Carrasco LR, Lee VJ, Chen MI, Matchar DB, Thompson JP, Cook AR. Strategies for antiviral stockpiling for future influenza pandemics: a global epidemic-economic perspective. J R Soc Interface. 2011;8(62):1307–13.

1436. Verma AK, Dhama K, Chakraborty S, Kumar A, Tiwari R, Rahal A, et al. Strategies for combating and eradicating important infectious diseases of animals with particular reference to India: Present and future perspectives. Asian Journal of Animal and Veterinary Advances. 2014;9(2):77–106.

1437. Ferguson NM, Cummings DAT, Cauchemez S, Fraser C, Riley S, Meeyai A, et al. Strategies for containing an emerging influenza pandemic in Southeast Asia. Nature. 2005;437(7056):209–14.

1438. Rai NK, Rim KI, Wulandari EW, Subrata F, Sugihantono A, Sitohang V. Strengthening emergency preparedness and response systems: experience from Indonesia. WHO South-East Asia journal of public health. 2020;9(1):26–31.

1439. Choi YK, Nguyen TD, Ozaki H, Webby RJ, Puthavathana P, Buranathal C, et al. Studies of H5N1 influenza virus infection of pigs by using viruses isolated in Vietnam and Thailand in 2004. J Virol. 2005;79(16):10821–5.

1440. Kamath SD, Kumar M, Sunder A. Study of Profile of Swine Flu Cases Admitted in Tertiary Care Hospital: Lessons Learned! Infectious Diseases in Clinical Practice. 2021;29(3):E154–9.

1441. Sreedhar S, Antony A, Poulose N. Study on the effectiveness and impact of pentavalent vaccination program in India and other south Asian countries. Human Vaccines and Immunotherapeutics. 2014;10(7):2062–5.

1442. Manasatienkij W, Rutvisuttinunt W, Chinnawirotpaisan P, Bhoomiboonchoo P, Yoon IK, Klungthong C, et al. Study the effects of influenza vaccination on children in thailand by using next generation sequencing. Am J Trop Med Hyg. 2015;93(4):55.

1443. Roy G. Successful containment of an avian influenza outbreak through public health intervention in district Howrah, West Bengal, India, January’08. Int J Infect Dis. 2010;14:e370–1.

1444. Kale SD, Mishra AC, Pawar SD. Suitability of specimen types for isolation of avian influenza viruses from poultry. Indian Journal of Virology. 2013;24(3):391–3.

1445. Alexander DJ. Summary of avian influenza activity in Europe, Asia, Africa, and Australasia, 2002-2006. Avian Dis. 2007;51(1):161–6.

1446. Brown IH. Summary of avian influenza activity in Europe, Asia, and Africa, 2006-2009. Avian Dis. 2010;54(1):187–93.

1447. Buranathai C, Chaisigh A, Amonsin A, Pariyothorn N, Theamboonlers A, Poovorawan Y. Surveillance activities and molecular analysis of H5N1 highly pathogenic avian influenza viruses from Thailand, 2004-2005. Avian Dis. 2007;51:194–200.

1448. Rudge J, Coker R, Budayanti NS, Handayani L, Prashinta M, Adisasmito W, et al. Surveillance and characterisation of influenza among patients with influenza-like illness in Bali, Indonesia. Int J Infect Dis. 2012;16:e14.

1449. Adisasmito W, Budayanti S, Aisyah DN, Coker R, Andayani AR, Smith GJD, et al. Surveillance and characterisation of influenza viruses among patients with influenza-like illness in Bali, Indonesia, July 2010-June 2014. BMC Infectious Diseases [Internet]. 2019;19(1). Available from: https://www.scopus.com/inward/record.uri?eid=2-s2.0-85062641868&doi=10.1186%2fs12879-019-3842-5&partnerID=40&md5=bc8d0003b6566346a071fd2419b21872

1450. Roy T, Agrawal AS, Mukherjee A, Mishra AC, Chadha MS, Kaur H, et al. Surveillance and molecular characterization of human influenza B viruses during 2006-2010 revealed co-circulation of Yamagata-like and Victoria-like strains in eastern India. Infection, Genetics and Evolution. 2011;11(7):1595–601.

1451. Hartaningsih N, Wibawa H, Pudjiatmoko, Rasa FST, Irianingsih SH, Dharmawan R, et al. Surveillance at the molecular level: Developing an integrated network for detecting variation in avian influenza viruses in Indonesia. Preventive Veterinary Medicine. 2015;120(1):96–105.

1452. Pant GR, Selleck PW. Surveillance for avian influenza in Nepal 2004-2005. Avian Dis. 2007;51:352–4.

1453. Fuller J, Hanley K, Schultz R, Lewis M, Freed NE, Ellis M, et al. Surveillance for febrile respiratory infections during Cobra Gold 2003. Mil Med. 2006;171(5):357–9.

1454. Surarith W, Ketchim N, Chaiwattanarungruengpaisan S, Kaewchot S, Lekcharoen P, Mongkolphan C, et al. Surveillance for influenza virus in nonhuman primates (NHPs) in Thailand, 2009-2018. Thai Journal of Veterinary Medicine. 2020;50(4):599–602.

1455. Tantawiwattananon N, Chiangson T, Rattanavibul K, Sakdajiwajaroen V, Iamsirithaworn S, Suanngam L, et al. Surveillance in 2013 of avian influenza virus from live-bird markets in Bangkok, Thailand. Southeast Asian Journal of Tropical Medicine and Public Health. 2017;48(1):37–44.

1456. Sarkar M, Chanda S, Chakrabarti S, Mazumdar J, Ganguly A, Chadha MS, et al. Surveillance in eastern India (2007-2009) revealed reassortment event involving ns and PB1-F2 gene segments among co-circulating influenza a subtypes. Virology Journal [Internet]. 2012;9. Available from: https://www.scopus.com/inward/record.uri?eid=2-s2.0-84855360765&doi=10.1186%2f1743-422X-9-3&partnerID=40&md5=8579b4a12cdebb7e8e1dd35a69489cde

1457. Mahardika GN, Adi AAAM, Besung NK, Dharmawan NS, Kencana GAY, Rompis ALT, et al. Surveillance of avian influenza virus of H5N1 subtype in backyard animals and its introduction in Bali, Indonesia. Pakistan Veterinary Journal. 2018;38(1):7–12.

1458. Khatun A, Giasuddin M, Islam KM, Khanom S, Samad MA, Islam MR, et al. Surveillance of avian influenza virus type A in semi-scavenging ducks in Bangladesh. BMC Veterinary Research [Internet]. 2013;9. Available from: https://www.scopus.com/inward/record.uri?eid=2-s2.0-84884993317&doi=10.1186%2f1746-6148-9-196&partnerID=40&md5=61a4c5ff1ca220a2490169caa8596706

1459. Karmacharya D, Manandhar S, Sharma A, Bhatta T, Adhikari P, Sherchan AM, et al. Surveillance of influenza a virus and its subtypes in migratory wild birds of Nepal. PLoS ONE. 2015;10(7):e0133035.

1460. Thurain K, Mon PP, Nasamran C, Charoenkul K, Boonyapisitsopa S, Tun TN, et al. Surveillance of influenza A virus subtype H5N1 in a live bird market in Yangon, Myanmar: 2017–2018. Transboundary and Emerging Diseases. 2020;67(6):2667–78.

1461. Kosasih H, Roselinda, Nurhayati, Klimov A, Xiyan X, Lindstrom S, et al. Surveillance of Influenza in Indonesia, 2003-2007. Influenza and other Respiratory Viruses. 2013;7(3):312–20.

1462. Rahman MM, Nooruzzaman M, Kabiraj CK, Mumu TT, Das PM, Chowdhury EH, et al. Surveillance on respiratory diseases reveals enzootic circulation of both H5 and H9 avian influenza viruses in small-scale commercial layer farms of Bangladesh. Zoonoses and Public Health [Internet]. 2021; Available from: https://www.scopus.com/inward/record.uri?eid=2-s2.0-85109033290&doi=10.1111%2fzph.12879&partnerID=40&md5=6e9402658222ef5dcec8cdddb28eeb5e

1463. Ansari WK, Parvej MS, El Zowalaty ME, Jackson S, Bustin SA, Ibrahim AK, et al. Surveillance, epidemiological, and virological detection of highly pathogenic H5N1 avian influenza viruses in duck and poultry from Bangladesh. Veterinary Microbiology. 2016;193:49–59.

1464. Arya K, Gupta R, Saxena VL. Survey of highly pathogenic avian influenza virus (H5N1) and its reoccurring threat: A brief review on different quails worldwide. Journal of World’s Poultry Research. 2018;8(4):81–94.

1465. Kurmi B, Murugkar HV, Nagarajan S, Tosh C, Dubey SC, Kumar M. Survivability of highly pathogenic avian influenza H5N1 virus in poultry faeces at different temperatures. Indian Journal of Virology. 2013;24(2):272–7.

1466. Hurt AC, Selleck P, Komadina N, Shaw R, Brown L, Barr IG. Susceptibility of highly pathogenic A(H5N1) avian influenza viruses to the neuraminidase inhibitors and adamantanes. Antiviral Res. 2007;73(3):228–31.

1467. Hurt AC, Barr IG, Hampson AW, Hartel G. Susceptibility of human influenza viruses from Australasia and South East Asia to the neuraminidase inhibitors zanamivir and oseltamivir. Antiviral Res. 2004;62(1):37–45.

1468. Silkavute P, Tung DX, Jongudomsuk P. Sustaining a regional emerging infectious disease research network: A trust-based approach. Emerging Health Threats Journal [Internet]. 2013;6(1). Available from: https://www.scopus.com/inward/record.uri?eid=2-s2.0-84881247143&doi=10.3402%2fehtj.v6i0.19957&partnerID=40&md5=c23e48b877ed76c92f5133d97129bf05

1469. Viveki RG, Halappanavar AB, Patil MS, Joshi AV, Gunagi P, Halki SB. Swine flu (H1N1 influenza): Awareness profile of visitors of swine flu screening booths in Belgaum city, Karnataka. Journal of the Indian Medical Association. 2012;110(6):358–61.

1470. Singh N, Singh S, Sharma BB, Singh V. Swine flu fibrosis: Regressive or progressive? Lung India. 2016;33(2):219–21.

1471. Katyal VK, Katyal A, Arora A, Mohan K, Bala K. Swine flu vaccination. Journal, Indian Academy of Clinical Medicine. 2016;17(3):205–8.

1472. Perera HKK, Wickramasinghe G, Cheung CL, Nishiura H, Smith DK, Poon LLM, et al. Swine influenza in Sri Lanka. Emerging Infectious Diseases. 2013;19(3):481–4.

1473. Trevennec K, Cowling BJ, Peyre M, Baudon E, Martineau GP, Roger F. Swine influenza surveillance in East and Southeast Asia: a systematic review. Anim Health Res Rev. 2011;12(2):213–23.

1474. Takemae N, Parchariyanon S, Ruttanapumma R, Hiromoto Y, Hayashi T, Uchida Y, et al. Swine influenza virus infection in different age groups of pigs in farrow-to-finish farms in Thailand. Virology Journal [Internet]. 2011;8. Available from: https://www.scopus.com/inward/record.uri?eid=2-s2.0-83355164475&doi=10.1186%2f1743-422X-8-537&partnerID=40&md5=fb21719983a6b3246de16a79c77862b5

1475. Mon PP, Thurain K, Janetanakit T, Nasamran C, Bunpapong N, Aye AM, et al. Swine influenza viruses and pandemic H1N1-2009 infection in pigs, Myanmar. Transboundary and Emerging Diseases. 2020;67(6):2653–66.

1476. Saha A, Jha N, Dubey NK, Gupta VK, Kalaivani M. Swine-origin influenza A (H1N1) in Indian children. Annals of Tropical Paediatrics. 2010;30(1):51–5.

1477. Kaulgud RS, Kamath V, Patil V, Desai S. Symmetric peripheral gangrene associated with H1N1 infection. International Journal of Preventive Medicine. 2013;4(10):1206–9.

1478. Xia H, Nagaraj K, Chen J, Marathe MV. Synthesis of a high resolution social contact network for Delhi with application to pandemic planning. Artif Intell Med. 2015;65(2):113–30.

1479. Halton K, Sarna M, Barnett A, Graves N, Leonardo L. Systematic review of community-based strategies to control emerging zoonotic infectious diseases in Southeast Asia. Int J Infect Dis. 2012;16:e260.

1480. Myaing MZ, Sugrue RJ, Taye B, Tan BH, Maurer-Stroh S, Chen H. Systems-based approach to examine the cytokine responses in primary mouse lung macrophages infected with low pathogenic avian Influenza virus circulating in South East Asia. BMC Genomics. 2017;18(1):420.

1481. Noisumdaeng P, Roytrakul T, Prasertsopon J, Pooruk P, Lerdsamran H, Assanasen S, et al. T cell mediated immunity against influenza H5N1 nucleoprotein, matrix and hemagglutinin derived epitopes in H5N1 survivors and non-H5N1 subjects. PeerJ. 2021;9:e11021.

1482. Ventura R, Brunner L, Heriyanto B, De Boer O, O’Hara M, Huynh C, et al. Technology transfer of an oil-in-water vaccine-adjuvant for strengthening pandemic influenza preparedness in Indonesia. Vaccine. 2013;31(12):1641–5.

1483. Bahl J, Nelson MI, Chan KH, Chen R, Vijaykrishna D, Halpin RA, et al. Temporally structured metapopulation dynamics and persistence of influenza A H3N2 virus in humans. Proc Natl Acad Sci U S A. 2011;108(48):19359–64.

1484. Girard MP, Tam JS, Assossou OM, Kieny MP. The 2009 A (H1N1) influenza virus pandemic: A review. Vaccine. 2010;28(31):4895–902.

1485. McLean KA, Goldin S, Nannei C, Sparrow E, Torelli G. The 2015 global production capacity of seasonal and pandemic influenza vaccine. Vaccine. 2016;34(45):5410–3.

1486. Koul P, Potdar V, Mir H, Chadha M. The 2015 Outbreak of Severe Influenza in Kashmir, North India: Emergence of a New Clade of A/H1n1 Influenza Virus. PLoS Curr. 2018;10.

1487. Goyal S, Prasert K, Praphasiri P, Chittaganpitch M, Waicharoen S, Ditsungnoen D, et al. The acceptability and validity of self-collected nasal swabs for detection of influenza virus infection among older adults in Thailand. Influenza Other Respir Viruses. 2017;11(5):412–7.

1488. Prasarnphanich T. The accuracy of clinical diagnosis of influenza in Thai children. Journal of Pediatric Infectious Diseases. 2010;5(2):155–9.

1489. Ciptaningtyas VR, De Mast Q, De Jonge MI. The burden and etiology of lower respiratory tract infections in children under five years of age in Indonesia. J Infect Dev Ctries. 2021;15(5):603–14.

1490. Simmerman JM, Uyeki TM. The burden of influenza in East and South-East Asia: A review of the English language literature. Influenza and other Respiratory Viruses. 2008;2(3):81–92.

1491. Thapa B, Roguski K, Azziz-Baumgartner E, Siener K, Gould P, Jamtsho T, et al. The burden of influenza-associated respiratory hospitalizations in Bhutan, 2015-2016. Influenza Other Respir Viruses. 2019;13(1):28–35.

1492. Udompornwattana S, Srajai K, Suwan P, Tangsathapornpong A, Wittawatmongkol O, Phongsamart W, et al. The clinical features, risk of prolonged hospitalization and household infections of hospitalized children for pandemic 2009 influenza A (H1N1) virus infection in Thailand. Journal of the Medical Association of Thailand. 2012;95(3):403–11.

1493. Marinova-Petkova A, Turner J, Walker D, Seiler P, Franks J, McKenzie P, et al. The Continuing Evolution of H5N1 and H9N2 Influenza Viruses in Bangladesh Between 2013 and 2014. Avian Dis. 2016;60(1):108–17.

1494. Monsilp T, Khudphab T, Doloh S, Wasinrat P, Wutikul Y, Weerakhachon P, et al. The correlation between knowledge of influenza and perceived risk of influenza among clients in outpatient department of Yalasiriratthanarak Hospital. Enfermeria Clinica. 2020;30:61–3.

1495. Peasah SK, Ram Purakayastha D, Krishnan A, Koul P, Dawood F, Widdowson MA, et al. The cost of Hospitalization due to acute respiratory infections in Northern India. Value Health. 2014;17(3):A130–1.

1496. Simmerman JM, Dowell SF, Olsen SJ, Lertiendumrong J, Tangcharoensathien V, Uyeki T, et al. The cost of influenza in Thailand. Vaccine. 2006;24(20):4417–26.

1497. Kosasih H, Gasem MH, Tjitra E, Karyana M, Alisjahbana B, Lokida D, et al. The demography, clinical characteristics and diagnoses of acute febrile illness requiring hospitalization in indonesia. Am J Trop Med Hyg. 2017;97(5):557.

1498. Wilasang C, Wiratsudakul A, Chadsuthi S. The dynamics of avian influenza: Individual-based model with intervention strategies in traditional trade networks in Phitsanulok province, Thailand. Computational and Mathematical Methods in Medicine [Internet]. 2016;2016. Available from: https://www.scopus.com/inward/record.uri?eid=2-s2.0-84964814025&doi=10.1155%2f2016%2f6832573&partnerID=40&md5=89f8b189b3a39ab38bfe58986090942c

1499. Storms AD, Kusriastuti R, Misriyah S, Praptiningsih CY, Amalya M, Lafond KE, et al. The East Jakarta Project: Surveillance for highly pathogenic avian influenza A(H5N1) and seasonal influenza viruses in patients seeking care for respiratory disease, Jakarta, Indonesia, October 2011-September 2012. Epidemiology and Infection. 2015;143(16):3394–404.

1500. Henkle E, Steinhoff MC, Omer SB, Roy E, Arifeen SE, Raqib R, et al. The effect of exclusive breast-feeding on respiratory illness in young infants in a maternal immunization trial in Bangladesh. Pediatric Infectious Disease Journal. 2013;32(5):431–5.

1501. Thontiravong A, Prakairungnamthip D, Chanvatik S, Nonthabenjawan N, Tunterak W, Tangwangvivat R, et al. The effect of various erythrocyte species on the detection of avian, swine and canine influenza a viruses isolated in Thailand. Thai Journal of Veterinary Medicine. 2016;46(1):135–42.

1502. Meeyai A, Coker R, Cooper B, Pan W, Akarasewie P, Iamsirithaworn S. The effective reproduction number of Pandemic 2009 H1N1 influenza in Thailand: A spatiotemporal analysis. Int J Infect Dis. 2012;16:e353–4.

1503. Bett B, Mclaws M, Jost C, Schoonman L, Unger F, Poole J, et al. The effectiveness of preventative mass vaccination regimes against the incidence of highly pathogenic avian influenza on Java Island, Indonesia. Transboundary and Emerging Diseases. 2015;62(2):163–73.

1504. Praditsuwan R, Assantachai P, Wasi C, Puthavatana P, Kositanont U. The efficacy and effectiveness of influenza vaccination among Thai elderly persons living in the community. J Med Assoc Thai. 2005;88(2):256–64.

1505. Bharmoria A, Vaish VB, Chaurasia A, Tahlan AK. The emergence and consistency of influenza strains causing influenza like illness in Himachal Pradesh, India. VirusDisease. 2016;27(2):130–5.

1506. Nguyen-Van-Tam JS, Hampson AW. The epidemiology and clinical impact of pandemic influenza. Vaccine. 2003;21(16):1762–8.

1507. Turner C, Turner P, Cararra V, Tha Ler Htoo S, Watthanaworawit W, Nosten F, et al. The epidemiology of pneumonia in a birth cohort of children living on the Thai-Myanmar border. Int J Infect Dis. 2012;16:e13.

1508. Asdie RH, Lie KC, Katu S, Aziz MH, Nurhayati N, Kosasih H, et al. The etiologies and outcomes of sepsis in patients with acute febrile illness in Indonesia: Recommendations for future directions. Am J Trop Med Hyg. 2018;99(4):607.

1509. Chandra S, Kassens-Noor E. The evolution of pandemic influenza: Evidence from India, 1918-19. BMC Infect Dis. 2014;14(1):510.

1510. Luo T, Shen X, Liu L, Liao M, Shen Y, Irwin DM. The evolutionary dynamics of H1N1/pdm2009 in India. Infec Genet Evol. 2018;65:276–82.

1511. Morales KF, Menning L, Lambach P. The faces of influenza vaccine recommendation: A Literature review of the determinants and barriers to health providers’ recommendation of influenza vaccine in pregnancy. Vaccine. 2020;38(31):4805–15.

1512. Suntarattiwong P, Mott JA, Mohanty S, Sinthuwattanawibool C, Srisantiroj N, Patamasingh Na Ayudhaya O, et al. The feasibility and performance of participant-collected mid-turbinate nasal swabs for detection of influenza virus, respiratory syncytial virus, and human metapneumovirus infections among pregnant women. J Infect Dis. 2021;

1513. Reading PC, Leung VK, Buettner I, Gillespie L, Deng YM, Shaw R, et al. The first external quality assessment of isolation and identification of influenza viruses in cell culture in the Asia Pacific region, 2016. Journal of Clinical Virology. 2017;97:54–8.

1514. Sarmanu, Rachmawati K, Santoso KP, Nidom CA, Indrasari S, Nidom RV, et al. THE FIRST PATHOGENICITY ANALYSIS REPORT IN MICE WITH TWO H9N2 SUBTYPE AVIAN INFLUENZA VIRUSES ISOLATED FROM INDONESIA. Biochem Cell Arch. 2021;21(1):593–8.

1515. Viseshakul N, Thanawongnuwech R, Amonsin A, Suradhat S, Payungporn S, Keawchareon J, et al. The genome sequence analysis of H5N1 avian influenza a virus isolated from the outbreak among poultry populations in Thailand. Virology. 2004;328(2):169–76.

1516. Sharma A, Cooper R, Bhardwaj G, Cannoo DS. The genus Nepeta: Traditional uses, phytochemicals and pharmacological properties. J Ethnopharmacol. 2021;268:113679.

1517. Berger KA, Pigott DM, Tomlinson F, Godding D, Maurer-Stroh S, Taye B, et al. The Geographic Variation of Surveillance and Zoonotic Spillover Potential of Influenza Viruses in Domestic Poultry and Swine. Open Forum Infect Dis. 2018;5(12):ofy318.

1518. Russell CA, Jones TC, Barr IG, Cox NJ, Garten RJ, Gregory V, et al. The global circulation of seasonal influenza A (H3N2) viruses. Science. 2008;320(5874):340–6.

1519. Bhatia P. The H1N1 influenza pandemic: need for solutions to ethical problems. Indian journal of medical ethics. 2013;10(4):259–63.

1520. Barde PV, Sahu M, Shukla MK, Bharti PK, Sharma RK, Sahare LK, et al. The high frequency of non-aspartic acid residues at HA222 in influenza A(H1N1) 2009 pandemic viruses is associated with mortality during the upsurge of 2015: A molecular and epidemiological study from central India. Epidemiology and Infection. 2017;145(13):2656–65.

1521. Rusmil K, Gunardi H, Fadlyana E, Soedjatmiko, Dhamayanti M, Sekartini R, et al. The immunogenicity, safety, and consistency of an Indonesia combined DTP-HB-Hib vaccine in expanded program on immunization schedule. BMC Pediatrics [Internet]. 2015;15(1). Available from: https://www.scopus.com/inward/record.uri?eid=2-s2.0-84951136426&doi=10.1186%2fs12887-015-0525-2&partnerID=40&md5=ef59eb01ae15929a1c260cc286289ec6

1522. Kanai Y, Boonsathorn N, Chittaganpitch M, Bai G, Li Y, Kase T, et al. The impact of antigenic drift of influenza A virus on human herd immunity: Sero-epidemiological study of H1N1 in healthy Thai population in 2009. Vaccine. 2010;28(33):5437–44.

1523. Klungthong C, Chinnawirotpisan P, Hussem K, Phonpakobsin T, Manasatienkij W, Ajariyakhajorn C, et al. The impact of primer and probe-template mismatches on the sensitivity of pandemic influenza A/H1N1/2009 virus detection by real-time RT-PCR. Journal of Clinical Virology. 2010;48(2):91–5.

1524. Adams B, McHardy AC. The impact of seasonal and year-round transmission regimes on the evolution of influenza A virus. Proc Biol Sci. 2011;278(1716):2249–56.

1525. Hill EM, House T, Dhingra MS, Kalpravidh W, Morzaria S, Osmani MG, et al. The impact of surveillance and control on highly pathogenic avian influenza outbreaks in poultry in Dhaka division, Bangladesh. PLoS Comput Biol. 2018;14(9):e1006439.

1526. Wanaratana S, Tantilertcharoen R, Sasipreeyajan J, Pakpinyo S. The inactivation of avian influenza virus subtype H5N1 isolated from chickens in Thailand by chemical and physical treatments. Veterinary Microbiology. 2010;140(1):43–8.

1527. De Paus RA, Van Crevel R, Van Beek R, Sahiratmadja E, Alisjahbana B, Marzuki S, et al. The influence of influenza virus infections on the development of tuberculosis. Tuberculosis. 2013;93(3):338–42.

1528. Bhavnani D, Olsen SJ, Simmerman JM, Phatinawin L, Chantra S. The influence of rapid influenza diagnostic testing on antibiotic prescribing patterns in rural Thailand. Int J Infect Dis. 2007;11(4):355–9.

1529. Hardiman D. The Influenza Epidemic of 1918 and the Adivasis of Western India. Soc Hist Med. 2012;25(3):644–64.

1530. Chandra S, Sarathchandra D. The influenza pandemic of 1918-1919 in Sri Lanka: Its demographic cost, timing, and propagation. Influ Other Respir Viruses. 2014;8(3):267–73.

1531. Kreslake JM, Wahyuningrum Y, Iuliano AD, Storms AD, Lafond KE, Mangiri A, et al. The intersection of care seeking and clinical capacity for patients with highly pathogenic avian influenza a (H5N1) virus in Indonesia: Knowledge and treatment practices of the public and physicians. Disaster Medicine and Public Health Preparedness. 2016;10(6):838–47.

1532. Song W, Wang P, Mok BWY, Lau SY, Huang X, Wu WL, et al. The K526R substitution in viral protein PB2 enhances the effects of E627K on influenza virus replication. Nat Commun. 2014;5:5509.

1533. Wu T, Perrings C. The live poultry trade and the spread of highly pathogenic avian influenza: Regional differences between Europe, West Africa, and Southeast Asia. PLoS ONE. 2018;13(12):e0208197.

1534. Suptawiwat O, Boonarkart C, Chakritbudsabong W, Uiprasertkul M, Puthavathana P, Wiriyarat W, et al. The N-linked glycosylation site at position 158 on the head of hemagglutinin and the virulence of H5N1 avian influenza virus in mice. Archives of Virology. 2015;160(2):409–15.

1535. Ortiz JR, Yu SL, Driscoll AJ, Williams SR, Robertson J, Hsu JS, et al. The operational feasibility of vaccination programs targeting influenza risk groups in the WHO African and South-East Asian Regions. Clin Infect Dis. 2021;

1536. Nelson MI, Simonsen L, Viboud C, Miller MA, Holmes EC. The origin and global emergence of adamantane resistant A/H3N2 influenza viruses. Virology. 2009;388(2):270–8.

1537. Magal P, Webb G. The parameter identification problem for SIR epidemic models: identifying unreported cases. J Math Biol. 2018;77(6):1629–48.

1538. Chowdhury S, Hossain ME, Ghosh PK, Ghosh S, Hossain MB, Beard C, et al. The pattern of highly pathogenic avian influenza H5N1 outbreaks in South Asia. Tropical Medicine and Infectious Disease [Internet]. 2019;4(4). Available from: https://www.scopus.com/inward/record.uri?eid=2-s2.0-85078194264&doi=10.3390%2ftropicalmed4040138&partnerID=40&md5=49ef0c03ffdd7907ecf72e0adec8c606

1539. Wanaratana S, Panyim S, Pakpinyo S. The potential of house flies to act as a vector of avian influenza subtype H5N1 under experimental conditions. Medical and Veterinary Entomology. 2011;25(1):58–63.

1540. Dawood FS, Hunt D, Patel A, Kittikraisak W, Tinoco Y, Kurhe K, et al. The Pregnancy and Influenza Multinational Epidemiologic (PRIME) study: a prospective cohort study of the impact of influenza during pregnancy among women in middle-income countries. Reproductive Health [Internet]. 2018;15(1). Available from: https://www.scopus.com/inward/record.uri?eid=2-s2.0-85053687990&doi=10.1186%2fs12978-018-0600-x&partnerID=40&md5=3b972c1972ff80bc760add2edda2685b

1541. Ramadhany R, Setiawaty V, Wibowo HA, Lokida D. The proportion of influenza infections from severe acute respiratory infection (SARI) cases in Indonesia 2008-2009. Int J Infect Dis. 2010;14:e277.

1542. Qureshi NR, Hien TT, Farrar J, Gleeson FV. The radiologic manifestations of H5N1 avian influenza. J Thorac Imaging. 2006;21(4):259–64.

1543. Baral SD, Rucinski KB, Twahirwa Rwema JO, Rao A, Prata Menezes N, Diouf D, et al. The Relationship Between the Global Burden of Influenza From 2017 to 2019 and COVID-19: Descriptive Epidemiological Assessment. JMIR Public Health Surveill. 2021;7(3):e24696.

1544. Shanmuganatham KK, Jones JC, Marathe BM, Feeroz MM, Jones-Engel L, Walker D, et al. The replication of Bangladeshi H9N2 avian influenza viruses carrying genes from H7N3 in mammals. Emerging Microbes and Infections. 2016;5(1):1–12.

1545. Indrawan D, Cahyadi ER, Daryanto A, Hogeveen H. The role of farm business type on biosecurity practices in West Java broiler farms. Preventive Veterinary Medicine [Internet]. 2020;176. Available from: https://www.scopus.com/inward/record.uri?eid=2-s2.0-85078810236&doi=10.1016%2fj.prevetmed.2020.104910&partnerID=40&md5=566fc0cf39cee7136ad5ab069cd62afe

1546. McHardy AC, Adams B. The role of genomics in tracking the evolution of influenza A virus. PLoS Pathog. 2009;5(10):e1000566.

1547. Salman MD. The role of veterinary epidemiology in combating infectious animal diseases on a global scale: the impact of training and outreach programs. Prev Vet Med. 2009;92(4):284–7.

1548. Levy JW, Cowling BJ, Simmerman JM, Olsen SJ, Fang VJ, Suntarattiwong P, et al. The serial intervals of seasonal and pandemic influenza viruses in households in Bangkok, Thailand. American Journal of Epidemiology. 2013;177(12):1443–51.

1549. Schaduangrat N, Phanich J, Rungrotmongkol T, Lerdsamran H, Puthavathana P, Ubol S. The significance of naturally occurring neuraminidase quasispecies of H5N1 avian influenza virus on resistance to oseltamivir: A point of concern. Journal of General Virology. 2016;97(6):1311–23.

1550. Ahmed SSU, Ersbøll AK, Biswas PK, Christensen JP. The space-time clustering of highly pathogenic avian influenza (HPAI) H5N1 outbreaks in Bangladesh. Epidemiology and Infection. 2010;138(6):843–52.

1551. Osmani MG, Ward MP, Giasuddin M, Islam MR, Kalam A. The spread of highly pathogenic avian influenza (subtype H5N1) clades in Bangladesh, 2010 and 2011. Preventive Veterinary Medicine. 2014;114(1):21–7.

1552. Gupta YK, Meenu M, Mohan P. The Tamiflu fiasco and lessons learnt. Indian Journal of Pharmacology. 2015;47(1):11–6.

1553. Wongsawat J, Chittaganpitch M, Ampornareekul S, Srisophaa S, Likanonsakul S. The validity of clinical practice guidelines for empirical use of oseltamivir for influenza in Thai children. Paediatrics and International Child Health. 2016;36(4):275–81.

1554. Shi W, Gibbs MJ, Zhang Y, Zhuang D, Dun A, Yu G, et al. The variable codons of H5N1 avian influenza A virus haemagglutinin genes. Sci China C Life Sci. 2008;51(11):987–93.

1555. Gabriel G, Dauber B, Wolff T, Planz O, Klenk HD, Stech J. The viral polymerase mediates adaptation of an avian influenza virus to a mammalian host. Proc Natl Acad Sci U S A. 2005;102(51):18590–5.

1556. Kandun IN, Wibisono H, Sedyaningsih ER, Yusharmen, Hadisoedarsuno W, Purba W, et al. Three Indonesian clusters of H5N1 virus infection in 2005. N Engl J Med. 2006;355(21):2186–94.

1557. Omer SB, Richards JL, Madhi SA, Tapia MD, Steinhoff MC, Aqil AR, et al. Three randomized trials of maternal influenza immunization in Mali, Nepal, and South Africa: Methods and expectations. Vaccine. 2015;33(32):3801–12.

1558. Imai C, Armstrong B, Chalabi Z, Mangtani P, Hashizume M. Time series regression model for infectious disease and weather. Environ Res. 2015;142:319–27.

1559. Kar SS, Roy G, Selvaraj K, Ramalingam A. Time trend and predictors of lab positivity among suspected cases in the post pandemic phase of H1N1: An observation from a tertiary care hospital, South India. Australas Med J. 2016;9(5):92–102.

1560. De Blasio BF, Engebretsen S, Engo-Monsen K, Aleem MA, Gurley ES, Frigessi A. Time-aggregated mobile phone mobility data are sufficient for modelling influenza spread: The case of Bangladesh. J R Soc Interface. 2020;17(167):rsif20190809.

1561. Asavapiriyanont S, Kittikraisak W, Suntarattiwong P, Ditsungnoen D, Kaoiean S, Phadungkiatwatana P, et al. Tolerability of trivalent inactivated influenza vaccine among pregnant women, 2015. BMC Pregnancy and Childbirth [Internet]. 2018;18(1). Available from: https://www.scopus.com/inward/record.uri?eid=2-s2.0-85045749711&doi=10.1186%2fs12884-018-1712-6&partnerID=40&md5=9505bda42161b069f0544d7962f30519

1562. Sedyaningsih ER, Isfandari S, Soendoro T, Supari SF. Towards mutual trust, transparency and equity in virus sharing mechanism: The avian influenza case of Indonesia. Annals of the Academy of Medicine Singapore. 2008;37(6):482–8.

1563. Wei K, Lin Y, Li Y, Chen Y. Tracking the Evolution in Phylogeny, Structure and Function of H5N1 Influenza Virus PA Gene. Transbound Emerg Dis. 2016;63(5):548–63.

1564. Peltzer K, Pengpid S. Traditional health practitioners in Indonesia: Their profile, practice and treatment characteristics. Complementary Medicine Research. 2019;26(2):93–100.

1565. Ali ST, Kadi AS, Ferguson NM. Transmission dynamics of the 2009 influenza A (H1N1) pandemic in India: The impact of holiday-related school closure. Epidemics. 2013;5(4):157–63.

1566. Nandi JS, Rathore SS, Mathur BR. Transmission of infectious viruses in the natural setting at human-animal interface. Curr Res Virol Sci. 2021;2:100008.

1567. Chotiprasatintara S, Chanachai K, Thanapongtham W, Kalpravidh W, Chaisingh A, Wongkasemjit S, et al. Transmission of the highly pathogenic avian influenza virus H5N1 within flocks during the 2004 epidemic in Thailand. J Infect Dis. 2007;196(11):1679–84.

1568. Khaw SWS, Vu LT, Yulianto D, Meers J, Henning J. Transport of Moving Duck Flocks in Indonesia and Vietnam: Management Practices That Potentially Impact Avian Pathogen Dissemination. Frontiers in Veterinary Science [Internet]. 2021;8. Available from: https://www.scopus.com/inward/record.uri?eid=2-s2.0-85111135719&doi=10.3389%2ffvets.2021.673624&partnerID=40&md5=c045e74d353944bd90528b31282f7c31

1569. Rewar S, Mirdha D, Rewar P. Treatment and Prevention of Pandemic H1N1 Influenza. Annals of Global Health. 2015;81(5):645–53.

1570. Thewjitcharoen Y, Butadej S, Malidaeng A, Yenseung N, Nakasatien S, Lekpittaya N, et al. Trends in influenza and pneumococcal vaccine coverage in Thai patients with type 2 diabetes mellitus 2010–2018: Experience from a tertiary diabetes center in Bangkok. Journal of Clinical and Translational Endocrinology [Internet]. 2020;20. Available from: https://www.scopus.com/inward/record.uri?eid=2-s2.0-85084427245&doi=10.1016%2fj.jcte.2020.100227&partnerID=40&md5=3ce83395da65406ff341ef945ff8e007

1571. Singhi S, Chaudhary D, Varghese G, Bhalla A, Karthi N, Kalantri S, et al. Tropical fevers: Management guidelines. Indian Journal of Critical Care Medicine. 2014;18(2):62–9.

1572. Imai C, Brooks WA, Chung Y, Goswami D, Anjali BA, Dewan A, et al. Tropical influenza and weather variability among children in an urban low-income population in Bangladesh. Global Health Action [Internet]. 2014;7(1). Available from: https://www.scopus.com/inward/record.uri?eid=2-s2.0-84928764277&doi=10.3402%2fgha.v7.24413&partnerID=40&md5=94fbf9d742aa7fdd54b370a41a77476c

1573. Pringproa K, Rungsiwiwut R, Tantilertcharoen R, Praphet R, Pruksananonda K, Baumgärtner W, et al. Tropism and Induction of Cytokines in Human Embryonic-Stem Cells-Derived Neural Progenitors upon Inoculation with Highly- Pathogenic Avian H5N1 Influenza Virus. PLoS One. 2015;10(8):e0135850.

1574. Villanueva-Cabezas JP, Campbell PT, McCaw JM, Durr PA, McVernon J. Turnover of Village Chickens Undermines Vaccine Coverage to Control HPAI H5N1. Zoonoses Public Health. 2017;64(1):53–62.

1575. Rimi NA, Sultana R, Ishtiak-Ahmed K, Rahman MZ, Hasin M, Islam MS, et al. Understanding the failure of a behavior change intervention to reduce risk behaviors for avian influenza transmission among backyard poultry raisers in rural Bangladesh: A focused ethnography. BMC Public Health [Internet]. 2016;16(1). Available from: https://www.scopus.com/inward/record.uri?eid=2-s2.0-84983404623&doi=10.1186%2fs12889-016-3543-6&partnerID=40&md5=2cee85714df929a73e5f520b3950821e

1576. Payaprom Y, Bennett P, Burnard P, Alabaster E, Tantipong H. Understandings of influenza and influenza vaccination among high-risk urban dwelling Thai adults: A qualitative study. Journal of Public Health. 2010;32(1):26–31.

1577. Lemey P, Rambaut A, Bedford T, Faria N, Bielejec F, Baele G, et al. Unifying viral genetics and human transportation data to predict the global transmission dynamics of human influenza H3N2. PLoS Pathog. 2014;10(2):e1003932.

1578. Khokon MSI, Islam S, Islam A, Fakir MAU, Tasneem M, Billah MM, et al. Unusual crow (Corvus splendens) mortality event in Jessore, Bangladesh 2018. Int J Infect Dis. 2020;101:540.

1579. Haider N, Sturm-Ramirez K, Khan SU, Rahman MZ, Sarkar S, Poh MK, et al. Unusually High Mortality in Waterfowl Caused by Highly Pathogenic Avian Influenza A(H5N1) in Bangladesh. Transboundary and Emerging Diseases. 2017;64(1):144–56.

1580. Update: influenza activity--United States and worldwide, 2003-04 season, and composition of the 2004-05 influenza vaccine. MMWR Morb Mortal Wkly Rep. 2004;53(25):547–52.

1581. Update: Influenza activity--United States and worldwide, 2004-05 season. MMWR Morb Mortal Wkly Rep. 2005;54(25):631–4.

1582. Thongpan I, Vichaiwattana P, Vongpunsawad S, Poovorawan Y. Upsurge of human rhinovirus infection followed by a delayed seasonal respiratory syncytial virus infection in Thai children during the coronavirus pandemic. Influenza Other Respir Viruses. 2021;

1583. Hardhantyo M, Chuang YC. Urban-rural differences in factors associated with incomplete basic immunization among children in Indonesia: A nationwide multilevel study. Pediatrics and Neonatology. 2021;62(1):80–9.

1584. Tillekeratne LG, Bodinayake C, Nagahawatte A, Kurukulasooriya R, Orlando LA, Simmons RA, et al. Use of clinical algorithms and rapid influenza testing to manage influenza-like illness: A cost-effectiveness analysis in Sri Lanka. BMJ Global Health [Internet]. 2019;4(2). Available from: https://www.scopus.com/inward/record.uri?eid=2-s2.0-85063660120&doi=10.1136%2fbmjgh-2018-001291&partnerID=40&md5=0e91bed3f21288ca52688ff41b968280

1585. Tare DS, Pawar SD. Use of embryonated chicken egg as a model to study the susceptibility of avian influenza H9N2 viruses to oseltamivir carboxylate. Journal of Virological Methods. 2015;224:67–72.

1586. Kinikar A, Kulkarni R, Valvi C, Gupte N. Use of indigenous bubble CPAP during swine flu pandemic in Pune, India. Indian Journal of Pediatrics. 2011;78(10):1216–20.

1587. Wibowo MH, Tarigan S, Sumarningsih, Artanto S, Indriani R, Anggoro D, et al. Use of M2e ELISAs for longitudinal surveillance of commercial poultry in Indonesia vaccinated against highly pathogenic avian influenza. Journal of Virological Methods. 2017;249:181–8.

1588. Somrongthong R, Beaudoin A, Bender J, Sasipreeyajan J, Laosee O, Pakinsee S, et al. Use of Personal Protective Measures by Thai Households in Areas with Avian Influenza Outbreaks. Zoonoses and Public Health. 2012;59(5):339–46.

1589. Tillekeratne LG, Bodinayake CK, Nagahawatte A, Vidanagama D, Devasiri V, Arachchi WK, et al. Use of rapid influenza testing to reduce antibiotic prescriptions among outpatients with influenza-like illness in southern Sri Lanka. American Journal of Tropical Medicine and Hygiene. 2015;93(5):1031–7.

1590. Gaur B, Saha S, Iuliano A, Rai S, Krishnan A, Jain S, et al. Use of TaqMan Array card for the detection of respiratory viral pathogens in children under 5 years old hospitalised with acute medical illness in Ballabgarh, Haryana, India. Indian Journal of Medical Microbiology. 2019;37(1):105–8.

1591. Wiwanitkit V. Usefulness of influenza vaccination in different groups of diabetic patients. Diabetes and Metabolic Syndrome: Clinical Research and Reviews. 2011;5(4):216–7.

1592. Mohan V, Kumar M S, Kumar CPG, Yuvaraj J, Krishnan A, Amarchand R, et al. Using global positioning system technology and Google My Maps in follow-up studies—An experience from influenza surveillance study, Chennai, India. Spatial and Spatio-temporal Epidemiology [Internet]. 2020;32. Available from: https://www.scopus.com/inward/record.uri?eid=2-s2.0-85077159899&doi=10.1016%2fj.sste.2019.100321&partnerID=40&md5=b3650ea06f6bbf01b3c23c0a449d7231

1593. Ge E, Haining R, Li CP, Chu KH, Yu Z, Waye MY, et al. Using knowledge fusion to analyze avian influenza H5N1 in east and Southeast Asia. PLoS ONE. 2012;7(5):e29617.

1594. Payaprom Y, Bennett P, Alabaster E, Tantipong H. Using the Health Action Process Approach and Implementation Intentions to Increase Flu Vaccine Uptake in High Risk Thai Individuals: A Controlled Before-After Trial. Health Psychology. 2011;30(4):492–500.

1595. Chawansuntati K, Aurpibul L, Wipasa J. Vaccination for 2009 pandemic H1N1 influenza A did not induce conserved epitope-specific memory CD8 T cell responses in HIV+ northern Thai children. Vaccine. 2015;33(38):4741–4.

1596. Bhaskar E, Thobias S, Anthony S, Kumar V, Navaneethan N. Vaccination rates for pandemic influenza among pregnant women: An early observation from Chennai, South India. Lung India. 2012;29(3):232–5.

1597. Bodewes R, Kreijtz JHCM, Hillaire MLB, Geelhoed-Mieras MM, Fouchier RAM, Osterhaus ADME, et al. Vaccination with whole inactivated virus vaccine affects the induction of heterosubtypic immunity against influenza virus A/H5N1 and immunodominance of virus-specific CD8+ T-cell responses in mice. J Gen Virol. 2010;91:1743–53.

1598. Tambunan USF, Sipahutar FRP, Parikesit AA, Kerami D. Vaccine Design for H5N1 Based on B- and T-cell Epitope Predictions. Bioinform Biol Insights. 2016;10:27–35.

1599. Gupta SS, Nair GB, Arora NK, Ganguly NK. Vaccine development and deployment: Opportunities and challenges in India. Vaccine. 2013;31:B43–53.

1600. Villanueva-Cabezas JP, Coppo MJC, Durr PA, McVernon J. Vaccine efficacy against Indonesian Highly Pathogenic Avian Influenza H5N1: systematic review and meta-analysis. Vaccine. 2017;35(37):4859–69.

1601. Dharmayanti NLPI, Indriani R, Nurjanah D. Vaccine efficacy on the novel reassortant h9n2 virus in indonesia. Vaccines. 2020;8(3):1–17.

1602. Tarbet EB, Dorward JT, Day CW, Rashid KA. Vaccine production training to develop the workforce of foreign institutions supported by the BARDA influenza vaccine capacity building program. Vaccine. 2013;31(12):1646–9.

1603. Gessner BD, Sedyaningsih ER, Griffiths UK, Sutanto A, Linehan M, Mercer D, et al. Vaccine-preventable haemophilus influenza type B disease burden and cost-effectiveness of infant vaccination in Indonesia. Pediatric Infectious Disease Journal. 2008;27(5):438–43.

1604. Martin S, Kilich E, Dada S, Kummervold PE, Denny C, Paterson P, et al. “Vaccines for pregnant women…?! Absurd” - Mapping maternal vaccination discourse and stance on social media over six months. Vaccine. 2020;38(42):6627–37.

1605. Jefferson T, Rivetti A, Di Pietrantonj C, Demicheli V. Vaccines for preventing influenza in healthy children. Cochrane Database Syst Rev. 2018;2(2):CD004879.

1606. Verma R, Khanna P, Chawla S. Vaccines for the elderly need to be introduced into the immunization program in India. Hum Vaccines Immunother. 2014;10(8):2468–70.

1607. Poon LLM, Leung YHC, Nicholls JM, Perera PY, Lichy JH, Yamamoto M, et al. Vaccinia virus-based multivalent H5N1 avian influenza vaccines adjuvanted with IL-15 confer sterile cross-clade protection in mice. J Immunol. 2009;182(5):3063–71.

1608. Gupta V, Dawood FS, Rai SK, Broor S, Wigh R, Mishra AC, et al. Validity of clinical case definitions for influenza surveillance among hospitalized patients: Results from a rural community in North India. Influenza and other Respiratory Viruses. 2013;7(3):321–9.

1609. Singh AK, Jain A, Jain B, Singh KP, Dangi T, Mohan M, et al. Viral aetiology of acute lower respiratory tract illness in hospitalised paediatric patients of a tertiary hospital: One year prospective study. Indian Journal of Medical Microbiology. 2014;32(1):13–8.

1610. Mishra P, Nayak L, Das RR, Dwibedi B, Singh A. Viral Agents Causing Acute Respiratory Infections in Children under Five: A Study from Eastern India. Int J Pediatr. 2016;2016:7235482.

1611. Wertheim HFL, Nadjm B, Thomas S, Agustiningsih, Malik S, Nguyen DNT, et al. Viral and atypical bacterial aetiologies of infection in hospitalised patients admitted with clinical suspicion of influenza in Thailand, Vietnam and Indonesia. Influenza and other Respiratory Viruses. 2015;9(6):315–22.

1612. Agustiningsih A, Herman R, Ramadhany R, Pratiwi E, Puspa KD, Setiawaty V. Viral and bacterial infection among hospitalized-suspected influenza A/H5N1 patients in Indonesia, 2008-2009. Medical Journal of Indonesia. 2012;21(2):77–82.

1613. Wannachai T, Kamalaporn H, Techasaensiri C, Preutthipan A. Viral associated severe community acquired pneumonia in children at ramathibodi hospital, Thailand. Am J Respir Crit Care Med [Internet]. 2012;185. Available from: http://www.atsjournals.org/doi/pdf/10.1164/ajrccm-conference.2012.185.1_MeetingAbstracts.A5479

1614. Jayaweera JAAS, Noordeen F, Morel A, Pitchai N, Kothalawala S, Abeykoon AMSB, et al. Viral burden in acute respiratory tract infections in hospitalized children in the wet and dry zones of Sri Lanka. Int J Infect Dis. 2016;45:463.

1615. Chittaganpitch M, Waicharoen S, Yingyong T, Praphasiri P, Sangkitporn S, Olsen SJ, et al. Viral etiologies of influenza-like illness and severe acute respiratory infections in Thailand. Influenza and other Respiratory Viruses. 2018;12(4):482–9.

1616. Muthulingama A, Noordeena F, Morelb AJ. Viral etiology in hospitalized children with acute respiratory tract infection in the Kegalle area of Sri Lanka. Journal of Pediatric Infectious Diseases. 2014;9(4):167–70.

1617. Pawestri HA, Eggink D, Isfandari S, Thanh TT, Rogier Van Doorn H, Setiawaty V, et al. Viral Factors Associated with the High Mortality Related to Human Infections with Clade 2.1 Influenza A/H5N1 Virus in Indonesia. Clinical Infectious Diseases. 2020;70(6):1139–46.

1618. Siengsanan-Lamont J, Robertson I, Blacksell SD, Ellis T, Fenwick S, Saengchoowong S, et al. Virological and molecular epidemiological investigations into the role of wild birds in the epidemiology of influenza A/H5N1 in central Thailand. Veterinary Microbiology. 2011;148(2):213–8.

1619. Yamaoka M, Palilingan JF, Nidom RV, Alamudi MY, Nidom CA, Wibisono J, et al. Virological surveillance of human influenza in Indonesia, October 2008-March 2010. Microbiol Immunol. 2011;55(7):514–7.

1620. Nasreen S, Homaira N, Al Mamun A, Bhuiyan M, Streatfield PK, Luby SP, et al. Virus-specific incidence rates of hospitalization for severe acute respiratory infections among children aged <5 years in rural Bangladesh, 2010. Int J Infect Dis. 2012;16:e12–3.

1621. Farida H, Gasem MH, Suryanto A, Keuter M, Zulkarnain N, Satoto B, et al. Viruses and Gram-negative bacilli dominate the etiology of community-acquired pneumonia in Indonesia, a cohort study. International Journal of Infectious Diseases. 2015;38:101–7.

1622. Malhotra B, Swamy MA, Janardhan Reddy PV, Gupta ML. Viruses causing severe acute respiratory infections (SARI) in children <=5 years of age at a tertiary care hospital in rajasthan, india. Indian J Med Res. 2016;144:877–85.

1623. Liao QY, Lam WWT, Dang VT, Jiang CQ, Udomprasertgul V, Fielding R. What causes H5N1 avian influenza? Lay perceptions of H5N1 aetiology in South East and East Asia. Journal of Public Health. 2009;31(4):573–81.

1624. Indrawan D, Tacken G, Hogeveen H. What drives the choice of poultry market channel and the change of purchase behavior due to highly pathogenic avian influenza outbreaks? Poult Sci. 2018;97(10):3652–60.

1625. Rimi NA, Nahar N, Sultana R, Ishtiak-Ahmed K, Haider N, Azziz-Baumgartner E, et al. Where backyard poultry raisers seek care for sick poultry: implications for avian influenza prevention in Bangladesh. BMC Public Health. 2018;18(1):969.

1626. Lister P, Dixit D, Diaz J, Shindo N. Who critical care training for the management of severe acute respiratory infection (SARI) deployed during outbreaks in Fiji august 2016 and maldives april 2017. Pediatr Crit Care Med. 2018;19(6):90.

1627. Henning J, Meers J, Pfeiffer DU, Stevenson M, Yulianto D, Priyono W. Who is spreading avian influenza in the moving duck flock farming network of Indonesia? PLoS ONE. 2016;11(3):e0152123.

1628. Makkoch J, Suwannakarn K, Prachayangprecha S, Cheiocharnsin T, Linsuwanon P, Theamboonlers A, et al. Whole Genome Characterization, Phylogenetic and Genome Signature Analysis of Human Pandemic H1N1 Virus in Thailand, 2009-2012. PLoS ONE. 2012;7(12):e51275.

1629. Naksupan N, Sanguansermsri D, Wongvilairat R, Niumsup PR, Pongcharoen S, Chamnanpood P, et al. Whole genome sequences of H5N1 influenza a virus isolated from a little grebe in Thailand. Southeast Asian Journal of Tropical Medicine and Public Health. 2008;39(3):373–82.

1630. Novianti AN, Rahardjo K, Prasetya RR, Nastri AM, Dewantari JR, Rahardjo AP, et al. Whole-genome sequence of an avian influenza A/H9N2 virus isolated from an apparently healthy chicken at a live-poultry market in Indonesia. Microbiology Resource Announcements [Internet]. 2019;8(17). Available from: https://www.scopus.com/inward/record.uri?eid=2-s2.0-85068003622&doi=10.1128%2fMRA.01671-18&partnerID=40&md5=28edbc128e99bacb6e1dc78dfb9cffbe

1631. Jones S, Prasad R, Nair AS, Usha R, Pillai RM, Dharmaseelan S, et al. Whole-genome sequences of influenza A(H1N1)pdm09 virus isolates from Kerala, India. Genome Announce. 2017;5(28):e00598-17.

1632. Wignjadiputro I, Widaningrum C, Setiawaty V, Widuri Wulandari E, Sihombing S, Prasetyo WA, et al. Whole–of–society approach for influenza pandemic epicenter Containment exercise in Indonesia. Journal of Infection and Public Health. 2020;13(7):994–7.

1633. Senthilkumar D, Kulkarni DD, Venkatesh G, Gupta V, Patel P, Dixit M, et al. Widespread Prevalence of Antibodies Against Swine Influenza A (pdm H1N1 09) Virus in Pigs of Eastern Uttar Pradesh, India. Current Microbiology. 2021;78(7):2753–61.

1634. Keawcharoen J, Osterhaus ADME, Van Den Broek J, Bouma A, Tiensin T, Heesterbeek H. Wild birds and increased transmission of highly pathogenic avian influenza (H5N1) among poultry, Thailand. Emerg Infect Dis. 2011;17(6):1016–22.

1635. Aro AR, Vartti AM, Schreck M, Turtiainen P, Uutela A. Willingness to take travel-related health risks--a study among Finnish tourists in Asia during the avian influenza outbreak. Int J Behav Med. 2009;16(1):68–73.

1636. Steinhoff MC, Reedy AM, McNeal M, Katz J, Stewart L, Mullany LC, et al. Year-round influenza immunisation during pregnancy in Nepal: a phase 4, randomised, placebo-controlled trial. Lancet Infect Dis. 2017;17(9):981–9.

1637. Hurt AC, Holien JK, Parker M, Kelso A, Barr IG. Zanamivir-resistant influenza viruses with a novel neuraminidase mutation. J Virol. 2009;83(20):10366–73.

1638. Vergne T, Paul MC, Chaengprachak W, Durand B, Gilbert M, Dufour B, et al. Zero-inflated models for identifying disease risk factors when case detection is imperfect: Application to highly pathogenic avian influenza H5N1 in Thailand. Preventive Veterinary Medicine. 2014;114(1):28–36.

1639. Ratananakorn L, Wilson D. Zoning and compartmentalisation as risk mitigation measures: an example from poultry production. Rev Sci Tech. 2011;30(1):297–307.

1640. Grace D, Gilbert J, Lapar ML, Unger F, Fevre S, Schelling E, et al. Zoonotic emerging infectious disease in selected countries in Southeast Asia: Insights from ecohealth. EcoHealth. 2011;8(1):55–62.

1641. Kumar B, Manuja A, Gulati BR, Virmani N, Tripathi BN. Zoonotic Viral Diseases of Equines and Their Impact on Human and Animal Health. Open Virol J. 2018;12:80–98.
